# Enhancing Electron Donor–Acceptor Complex Photoactivation with a Stable Perylene Diimide Metal–Organic Framework

**DOI:** 10.1021/jacs.4c16021

**Published:** 2025-01-27

**Authors:** Xia Wu, Ming Cui, Kun Wu, Jun Guo, Tianyu Liu, Dongyi Liu, Zekun Li, Puxin Weng, Ri-Qin Xia, Xiao Xiong, Yong-Liang Huang, Dan Li, Jian He

**Affiliations:** † Department of Chemistry, The University of Hong Kong, Hong Kong 999077, P. R. China; ‡ Guangdong Provincial Key Laboratory of Supramolecular Coordination Chemistry, Jinan University, Guangzhou 510632, P. R. China; § State Key Laboratory of Synthetic Chemistry, The University of Hong Kong, Hong Kong 999077, P. R. China; ∥ Department of Medicinal Chemistry, Shantou University Medical College, Shantou 515041, P.R. China; ⊥ Materials Innovation Institute for Life Sciences and Energy (MILES), HKU-SIRI, Shenzhen 518048, P. R. China

## Abstract

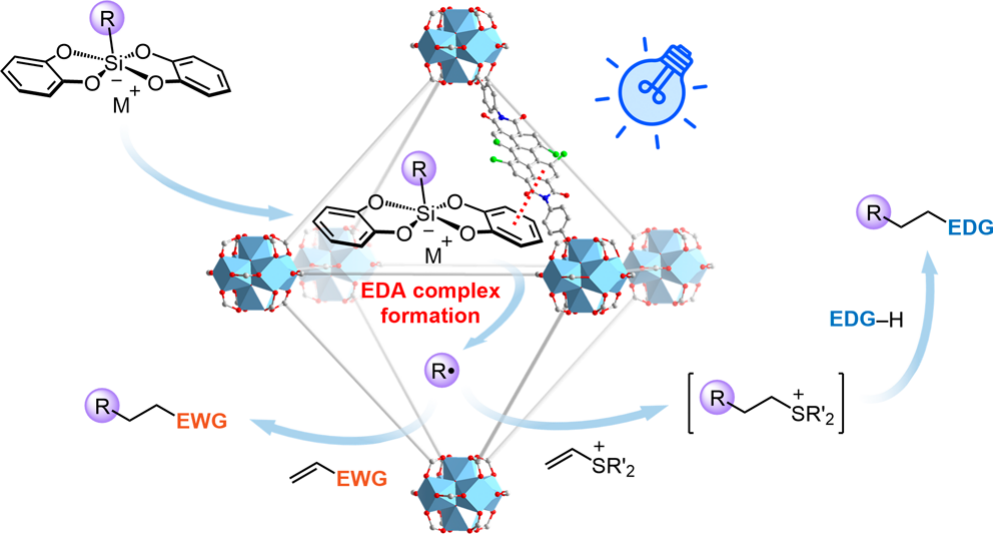

Electron donor–acceptor complexes are commonly employed to facilitate photoinduced radical-mediated organic reactions. However, achieving these photochemical processes with catalytic amounts of donors or acceptors can be challenging, especially when aiming to reduce catalyst loadings. Herein, we have unveiled a framework-based heterogenization approach that significantly enhances the photoredox activity of perylene diimide species in radical addition reactions with alkyl silicates by promoting faster and more efficient electron donor–acceptor complex formation. Besides offering broad substrate scope in alkene hydroalkylation, the newly developed heterogeneous photocatalysis substantially improves the catalyst turnover numbers in comparison to previous homogeneous photocatalytic systems and demonstrates outstanding catalyst recyclability. These research findings pave the way for the advancement of various efficient and practical organic transformations using framework-supported organocatalysts.

## Introduction

Due to their high tunability and expanded photoredox potentials, organo-photocatalysts have recently been widely explored in photoinduced organic transformations.^1−3^ Furthermore, by forming electron donor–acceptor (EDA) complexes with suitable organic substrates,^4−10^ these catalysts can efficiently facilitate single-electron transfer (SET) processes for radical formation under photoexcitation conditions, leading to the development of various radical-mediated bond-forming reactions.^11,12^ For instance, Melchiorre’s pioneering work showcased that tetrachlorophthalimides could engage with a range of electrophiles to catalyze photoinduced radical addition reactions via an EDA complex mechanism.^13^ Despite previous fruitful research into EDA complex photoactivation processes in homogeneous catalytic systems, the corresponding activation mode in heterogeneous photocatalysis remains underdeveloped to date,^14−22^ particularly when the catalysts act as electron acceptors in substrate activation.^13^

**Scheme 1:**
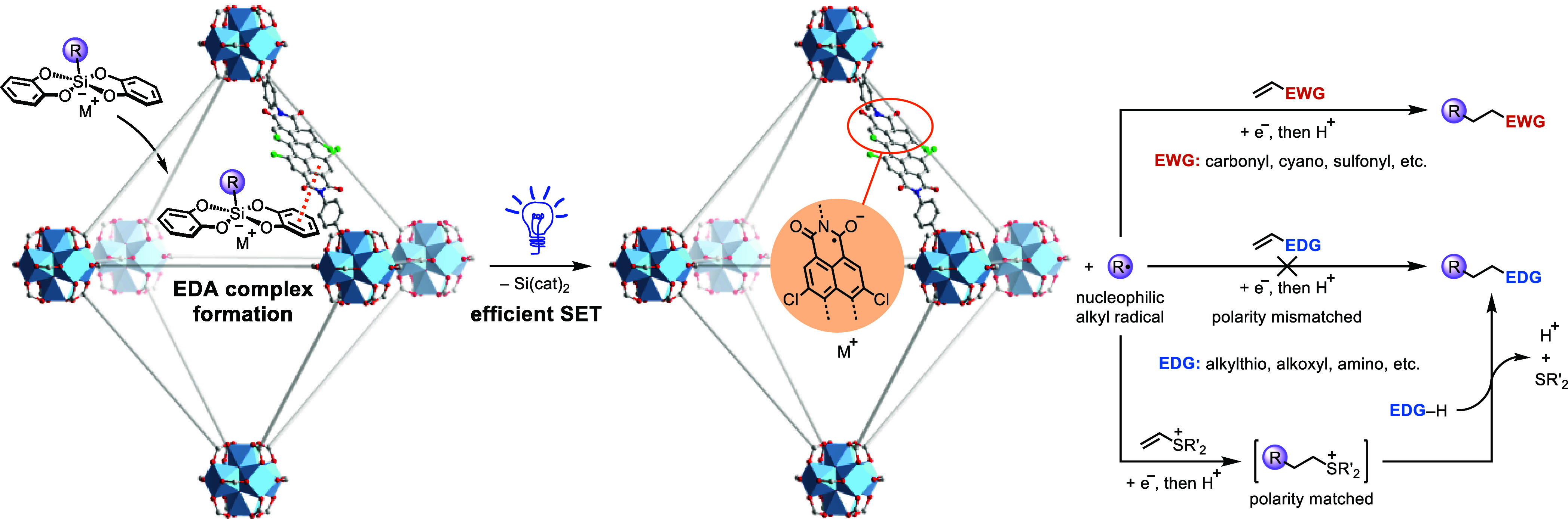
Design of a PDI-Based Framework Photocatalyst for Efficient Radical Additions

Considering the structural similarities between perylene diimide (PDI) derivatives and tetrachlorophthalimides, we envision that framework materials constructed by PDI organic linkers represent an ideal class of heterogeneous photocatalysts for the investigation of EDA complex photoactivation of alkyl silicates.^13,23^ Given the increased local concentration of PDI units^24^ and the confinement effect^25^ provided by the crystalline pores of the framework supports, the EDA interaction between the photoactive linker and the alkyl silicate is expected to be significantly enhanced, resulting in increased SET efficiency and effective radical generation at low catalyst loadings (Scheme 1). In addition, leveraging prior insights into the characterization of reactive intermediates, such as imide radical anions,^26,27^ in photoredox reactions catalyzed by PDIs establishes a solid foundation for understanding the mechanistic pathways in EDA-based heterogeneous photocatalysis.^28^

In this article, we present the synthesis of a stable UiO-type metal–organic framework (MOF)^29−40^ comprising [Zr_6_] inorganic nodes and PDI-containing dicarboxylate linkers designed for the efficient activation of alkyl silicates under visible-light irradiation. Departing from previous photocatalytic systems that were primarily focused on aryl halide dehalogenation^41−45^ and superoxide anion-mediated oxidation,^46,47^ the EDA complex photoactivation strategy employed in this PDI-based heterogeneous photocatalysis allows for the broad-scope hydroalkylation of alkenes bearing electron-withdrawing groups (EWGs). Notably, the substrate scope extends to vinyl sulfonium salts, facilitating the synthesis of polarity-mismatched radical addition products with electron-donating groups (EDGs) through subsequent nucleophilic substitution (Scheme 1).^48,49^ The PDI-based heterogeneous photocatalyst exhibits exceptional recyclability for practical organic synthesis, with photoluminescence studies revealing that the PDI linkers integrated into the MOF supports exhibit a stronger EDA interaction with alkyl silicates compared to their free form in solution, thereby elucidating the superior photocatalytic performance of MOF-supported PDIs in radical addition reactions.

## Results and Discussion

**Figure 1:**
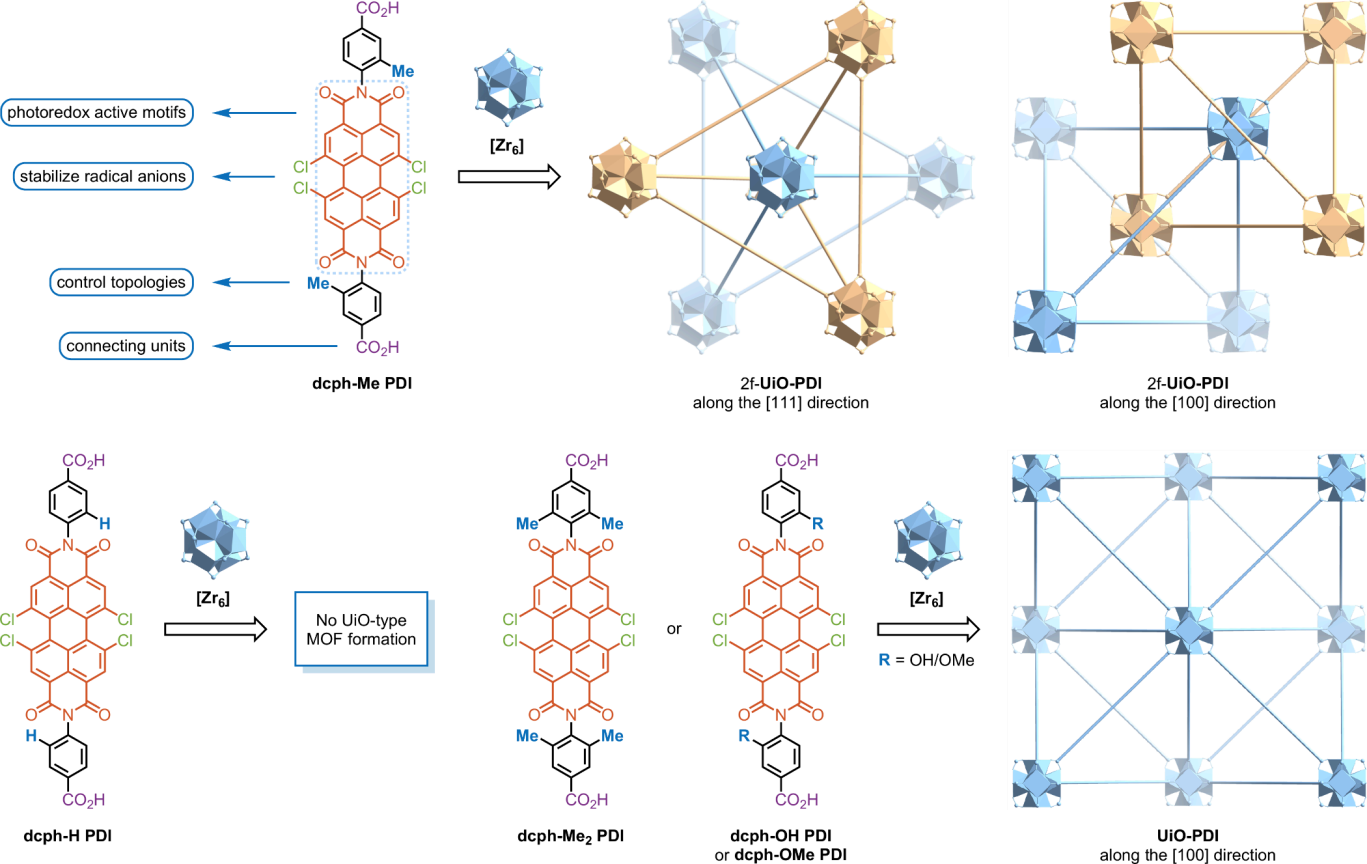
Impact of linker substituents on the formation of [Zr_6_]-based PDI MOFs.

The pronounced tendency of photoactive PDIs to aggregate, stemming from the strong π–π stacking interactions among planar perylene units,^50,51^ pose significant challenges for the development of PDI-based framework materials.^52−54^ To address this issue, we deliberately incorporated four chlorine atoms into the PDI core of the dibenzoic acid linker to induce the formation of a nonplanar structure, thereby enhancing its solubility. Moreover, the strong electron-withdrawing ability of the tetrachloride moiety can facilitate EDA complex formation and stabilize the PDI radical anion (PDI^•–^),^53^ substantially boosting the reactivity of PDI active sites in photoredox catalysis. While UiO-type MOFs are typically stable for catalytic applications,^55−57^ the utilization of dicarboxylic acid linkers with extended lengths often leads to diminished solvent and chemical stability.^58,59^ To produce stable MOF structures for EDA complex photoactivation, we introduced a methyl group at the 3-position of the benzoic acid motif in the linker (**dcph-Me PDI**),^60−65^ resulting in the construction of a 2-fold interpenetrated PDI-based UiO-type framework (2f-**UiO-PDI**) (Figures 1 and S1). The presence of two interpenetrating UiO-type networks in 2f-**UiO-PDI** was clearly identified through single-crystal X-ray diffraction analysis (Tables S1 and S2). For a direct comparison, the organic linker lacking any substituent on the benzoic acid motif failed to yield any UiO-type framework under solvothermal conditions. The introduction of 3,5-dimethyl, 3-hydroxy, or 3-methoxy groups produced conventional UiO-type MOFs without interpenetration, with their crystallinity drastically decreasing within 1 week (Figures S2–S7). This underscores the critical role of the 3-methyl substituent in catalyst preparation.

**Figure 2:**
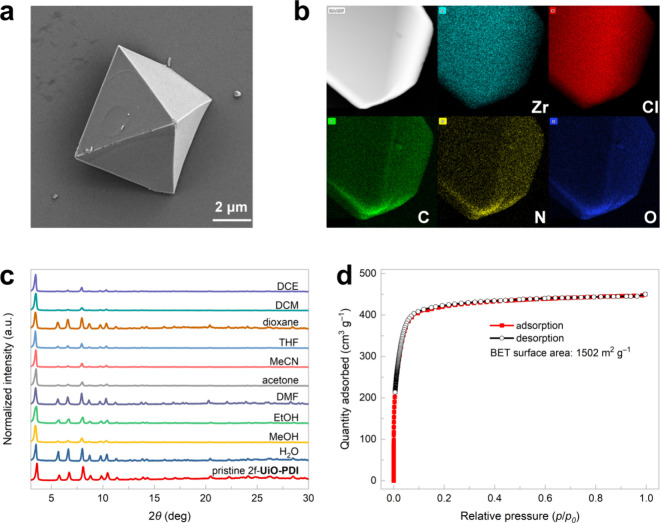
(a) SEM image of the 2f-**UiO-PDI** crystal. Scale bar, 2 μm. (b) TEM image and elemental mapping of 2f-**UiO-PDI**. EDS mapping images of Zr, Cl, C, N, and O are represented in cyan, red, green, yellow, and blue, respectively. (c) PXRD patterns of the pristine 2f-**UiO-PDI** and after the treatment with various solvents. (d) N_2_ adsorption/desorption isotherms of 2f-**UiO-PDI** collected at 77 K.

The addition of ZrCl_4_ to a DMF solution of **dcph-Me PDI**, with acetic acid as a modulator under solvothermal conditions at 90 °C, resulted in the formation of octahedral single crystals of 2f-**UiO-PDI**, measuring approximately 20 μm in length. These crystals displayed a smooth surface, as evidenced by scanning electron microscopy (SEM) and transmission electron microscopy (TEM) images (Figures 2a,b and S8). Energy-dispersive spectroscopy (EDS) mapping confirmed the uniform distribution of Zr, Cl, C, N, and O elements throughout the entire frameworks (Figures 2b and S9). The high phase purity of 2f-**UiO-PDI** was confirmed through powder X-ray diffraction (PXRD) analyses, which closely matched the simulated pattern derived from the single crystal structure (Figure S1).

To assess the solvent stability of the newly synthesized 2f-**UiO-PDI** framework, stability tests were conducted by immersing the pristine 2f-**UiO-PDI** in various organic solvents and water for 24 h. Consistent PXRD patterns were obtained, indicating that the crystallinity and structural integrity of the framework remained unchanged following these treatments (Figure 2c). The Brunauer–Emmett–Teller surface area of 2f-**UiO-PDI** was determined to be 1502 m^2^ g^–1^, with the pore width distribution centered at 1.6 nm (Figure S11), suggesting that 2f-**UiO-PDI** offers sufficient pore sizes to accommodate alkyl silicate substrates. To investigate its catalytic activity in EDA complex photoactivation, the loading of PDI active sites was estimated by extracting the mass percentage of solvent within the MOF pores through thermogravimetric analysis (Figures S12–S16).

While alkyl bis(catecholato)silicates have been widely used in radical-mediated organic transformations in conjunction with precious transition-metal and organo-photocatalysts,^66−74^ progress in EDA complex-enabled hydroalkylation using alkyl silicates as radical sources is still in its early stages,^23,75−78^ with no examples reported in PDI-based photocatalysis. Considering a potentially analogous activation mechanism to tetrachlorophthalimides and the confinement effects of MOF materials, it was envisioned that 2f-**UiO-PDI** could serve as an effective heterogeneous photocatalyst for the hydroalkylation of alkenes with alkyl silicates.

By utilizing cyclopentylsilicate **1a** and vinylsulfone **2a** as starting materials, a thorough exploration of various reaction parameters was conducted to establish a protocol that furnished the desired Giese addition product **3a** in 70% yield (Table 1, entry 1). Control experiments confirmed that the reaction did not proceed in the absence of photocatalysts or light (Table 1, entries 2–4). Even when maintained at 80 °C without light exposure, no radical addition occurred (Table 1, entry 4). The optimal irradiation wavelength for this reaction was determined to be 456 nm (Table 1, entries 5–7). Significantly, the existing **Zr-PDI** MOF,^53^ which has comparable linkers and metal nodes, gave only 28% yield of **3a** (Table 1, entry 8), indicating that the smaller MOF pore size caused by 5-fold interpenetration may impede the effective interaction between silicates and PDI linkers. As a result of inadequate structural stability, the UiO-PDI MOFs without interpenetration (i.e., **UiO-PDI-Me**_**2**_, **UiO-PDI-OH**, and **UiO-PDI-OMe** in Figure 1) exhibited a significant decrease in product yields during the second cycle (Table S3). The lower reactivity of various homogeneous counterparts underlines the importance of incorporating PDI photoactive units into reticular frameworks to develop an effective photoredox catalyst with improved efficiency in electron transfer (Table 1, entries 9–14). Although the photocatalytic performance of **dcph-Me PDI** and catalyst **A** did not match that of 2f-**UiO-PDI**, a further decline in product yields was noted upon removal of the tetrachloride moiety, highlighting the essential role of the electron-deficient nature of the PDI core in facilitating electron-transfer processes during hydroalkylation.^13^ Substituent variations on the terminal aryl groups had minimal impact on reaction efficiency. The similar trends were also observed in the photocatalytic systems involving phthalimides (Table 1, entries 12–14). Among the solvents examined, DMF proved to be the most effective (Table 1, entries 15–17). Decreasing the equivalents of **2a** corresponded to a reduction in yield (Table 1, entry 18). While sensitive to air atmosphere, the reaction is compatible with water (Table 1, entries 19 and 20).

**Table 1:**
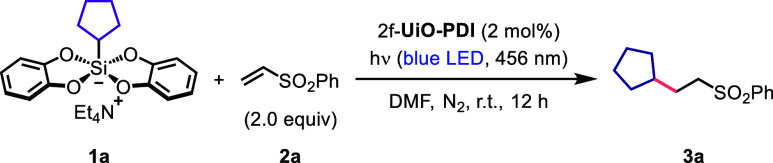
Effects of Various Reaction Parameters on Alkene Hydroalkylation with Silicates^a^

entry	change from the “standard conditions”	yield (%)
1	none	70
2	w/o 2f-**UiO-PDI**	<5
3	w/o light, r.t.	<5
4	w/o light, 80 °C	<5
5	hν (427 nm)	37
6	hν (440 nm)	40
7	hν (467 nm)	35
8	**Zr-PDI**, instead of 2f-**UiO-PDI**	28
9	**dcph-Me PDI**, instead of 2f-**UiO-PDI**	38
10	catalyst **A**, instead of 2f-**UiO-PDI**	34
11	catalyst **B**, instead of 2f-**UiO-PDI**	17
12	catalyst **C**, instead of 2f-**UiO-PDI**	15
13	catalyst **D**, instead of 2f-**UiO-PDI**	13
14	catalyst **E**, instead of 2f-**UiO-PDI**	<5
15	DMSO, instead of DMF	55
16	DCE, instead of DMF	<5
17	MeCN, instead of DMF	7
18	1.5 equiv of **2a**	65
19	under an atmosphere of air, instead of nitrogen	30
20	1.0 equiv H_2_O added	70

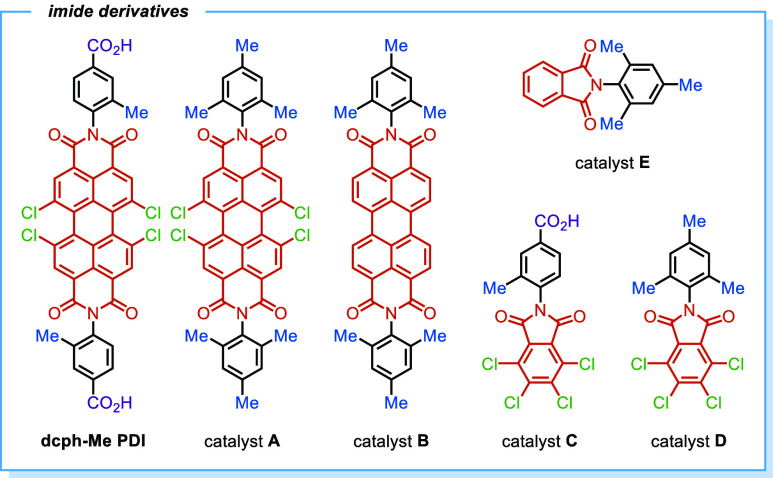

a Reaction conditions: **1a** (0.1 mmol, 1.0 equiv), **2a** (2.0 equiv), photocatalyst (2 mol %) in DMF (1.0 mL) under a nitrogen atmosphere at room temperature with blue LED light irradiation (456 nm) for 12 h. Yield was determined by ^1^H NMR of the crude product using mesitylene as an internal standard.

After identifying the optimal reaction conditions, we utilized *trans*-1,2-dibenzoylethylene (**2b**) as a Michael acceptor to investigate the scope of alkyl bis(catecholato)silicates in the radical addition for the synthesis of 1,4-dicarbonyl compounds (Table 2). In general, cycloalkyl silicates proved to be highly effective alkylation reagents (Table 2, products **3b**–**3e**), although the yield of a tetrahydropyran derivative (**3d**) was relatively modest. Because of the hyperconjugation effect and reduced steric hindrance, cycloalkyl radical intermediates are expected to be highly compatible with atom transfer radical addition processes. The exceptional stability of benzylic radicals enabled the Giese reaction with a benzyl silicate to afford **3f** in an excellent yield of 97%. Encouragingly, other primary alkyl silicates were well-tolerated (Table 2, products **3g**–**3l**); epoxide **3j**, ether **3k**, and alkyl chloride **3l** could all be successfully functionalized with a 1,4-diketone moiety.

**Table 2:**
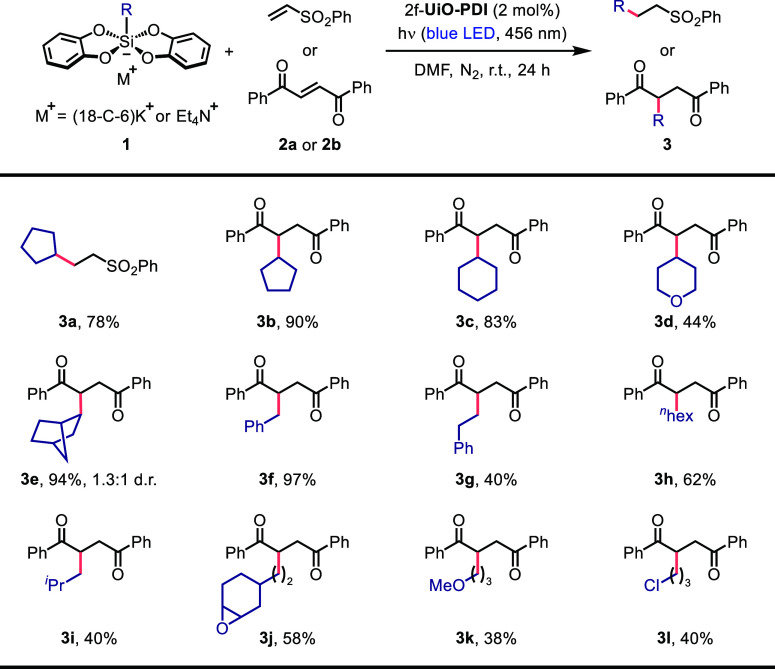
Substrate Scope for Silicates^a^

a Reaction conditions: **1** (0.1 mmol, 1.0 equiv), **2a** or **2b** (2.0 equiv), and 2f-**UiO-PDI** (2 mol %) in DMF (1.0 mL) under a nitrogen atmosphere at room temperature with blue LED light irradiation (456 nm) for 24 h. For each entry number (in bold), the data are presented as isolated yields after column chromatography separation.

Next, we evaluated the suitability of an array of electron-deficient alkenes as radical acceptors, employing *tert*-butyl silicate (**1m**) as the radical precursor (Table 3). Noteworthy outcomes included the successful alkylation of acrylic acids, acrylates, and acrylonitriles with α-aryl substituents, which provided the corresponding products in excellent yields (Table 3, products **3o**–**3q**). Transitioning from aryl groups to benzyl groups did not compromise the efficiency of alkene hydroalkylation (Table 3, products **3r** and **3s**). Furthermore, β-aryl-substituted methylene malonates, such as coumarin derivatives, demonstrated high reactivity as Michael acceptors in EDA complex-enabled radical addition, resulting in the formation of products **3t** and **3u**. Importantly, a 3-methoxycarbonyl-substituted indole derivative exhibited excellent compatibility with heterogeneous photocatalysis, furnishing 2-alkylated indoline **3v** in 70% yield. The addition of a *tert*-butyl radical into the double bond of cyclic substrates^79,80^ demonstrated remarkable diastereoselectivity (Table 3, products **3u** and **3v**). In scenarios where an acetoxymethyl group was present at the α-position of a vinylarylketone or a trifluoromethyl group at the α-position of a styrene, the elimination of an acetate or fluoride anion occurred instead of protonation,^23,69,71,76^ providing a multisubstituted alkene as the sole product (Table 3, products **3w** and **3x**).

**Table 3:**
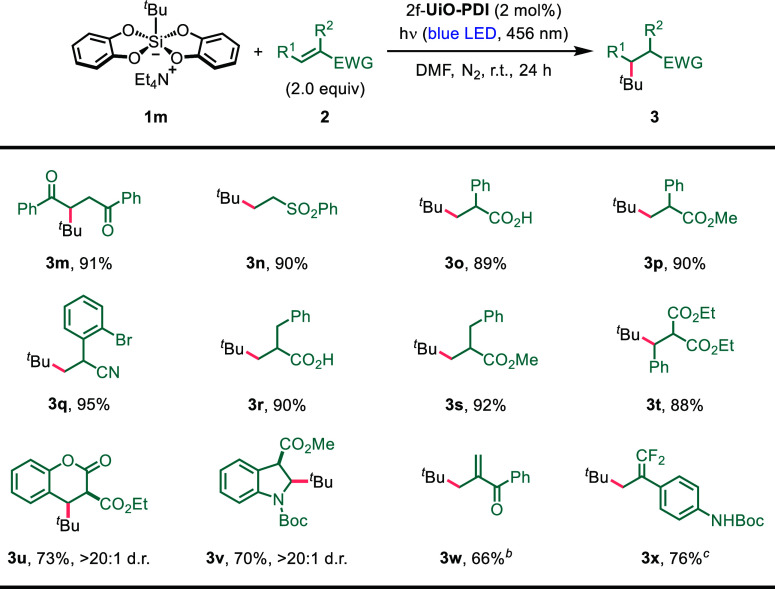
Substrate Scope for Alkenes^a^

a Reaction conditions: **1m** (0.1 mmol, 1.0 equiv), **2** (2.0 equiv), and 2f-**UiO-PDI** (2 mol %) in DMF (1.0 mL) under a nitrogen atmosphere at room temperature with blue LED light irradiation (456 nm) for 24 h. For each entry number (in bold), the data are presented as isolated yields after column chromatography separation.

b Allyl acetate as the alkene partner.

c α-(Trifluoromethyl)styrene as the alkene partner.

Despite the wide applicability of electron-deficient alkenes in radical addition with alkyl silicates,^75,81,82^ the photocatalytic process is not compatible with electron-donating alkenes due to the strong nucleophilicity of alkyl radicals.^83,84^ To address this limitation, we implemented the polarity transduction strategy, originally pioneered by Silvi in alkene hydroalkylation with aliphatic acids,^48^ to broaden the range of alkenes through a radical addition/nucleophilic substitution sequence. Using vinyl sulfonium triflate **4** as the standard alkene substrate, we explored the versatility of nucleophiles in the cascade reaction involving a trialkyl sulfonium intermediate (Table 4). In addition to arylthiols, including 2-mercaptopyridine, both primary and secondary alkyl thiols were effective in producing the desired sulfides in high yields (Table 4, products **5a**–**5g**). Nucleophilic substitution with 2-mercaptoethanol selectively occurred at the *S*-terminal, giving the corresponding β-alkylthio-substituted alcohol **5h** in a 60% yield. Furthermore, phenol and 3-phenylpropionic acid served as suitable nucleophiles for replacing the sulfonium leaving group, resulting in the formation of aryl alkyl ether **5i** and alkyl ester **5j** in 55 and 70% yield, respectively. At elevated temperatures and with increased base loadings, *N*-alkyl anilines and secondary alkylamines could also be obtained in the radical addition/nucleophilic substitution sequence initiated by PDI-based heterogeneous photocatalysis (Table 4, products **5k** and **5l**).

**Table 4:**
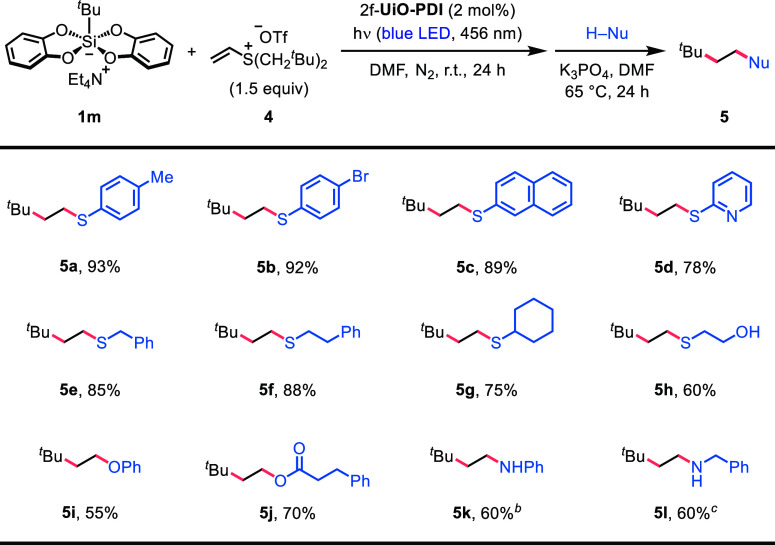
Cascade Radical Addition/Nucleophilic Substitution^a^

a Reaction conditions: **1m** (0.1 mmol, 1.0 equiv), **4** (0.15 mmol, 1.5 equiv), and 2f-**UiO-PDI** (2 mol %) in DMF (1.0 mL) under a nitrogen atmosphere at room temperature with blue LED light irradiation (456 nm) for 24 h. After filtration of 2f-**UiO-PDI** and concentration, nucleophile (0.25 mmol, 2.5 equiv) and K_3_PO_4_ (1.5 or 2.5 equiv) in DMF (0.2 mL) were added, and the reaction mixture was stirred under a nitrogen atmosphere at 65 °C for 24 h. For each entry number (in bold), the data are presented as isolated yields after column chromatography separation.

b 120 °C.

c Benzylamine (4.0 equiv), K_3_PO_4_ (1.5 equiv), MeCN (0.2 mL), 80 °C.

**Figure 3:**
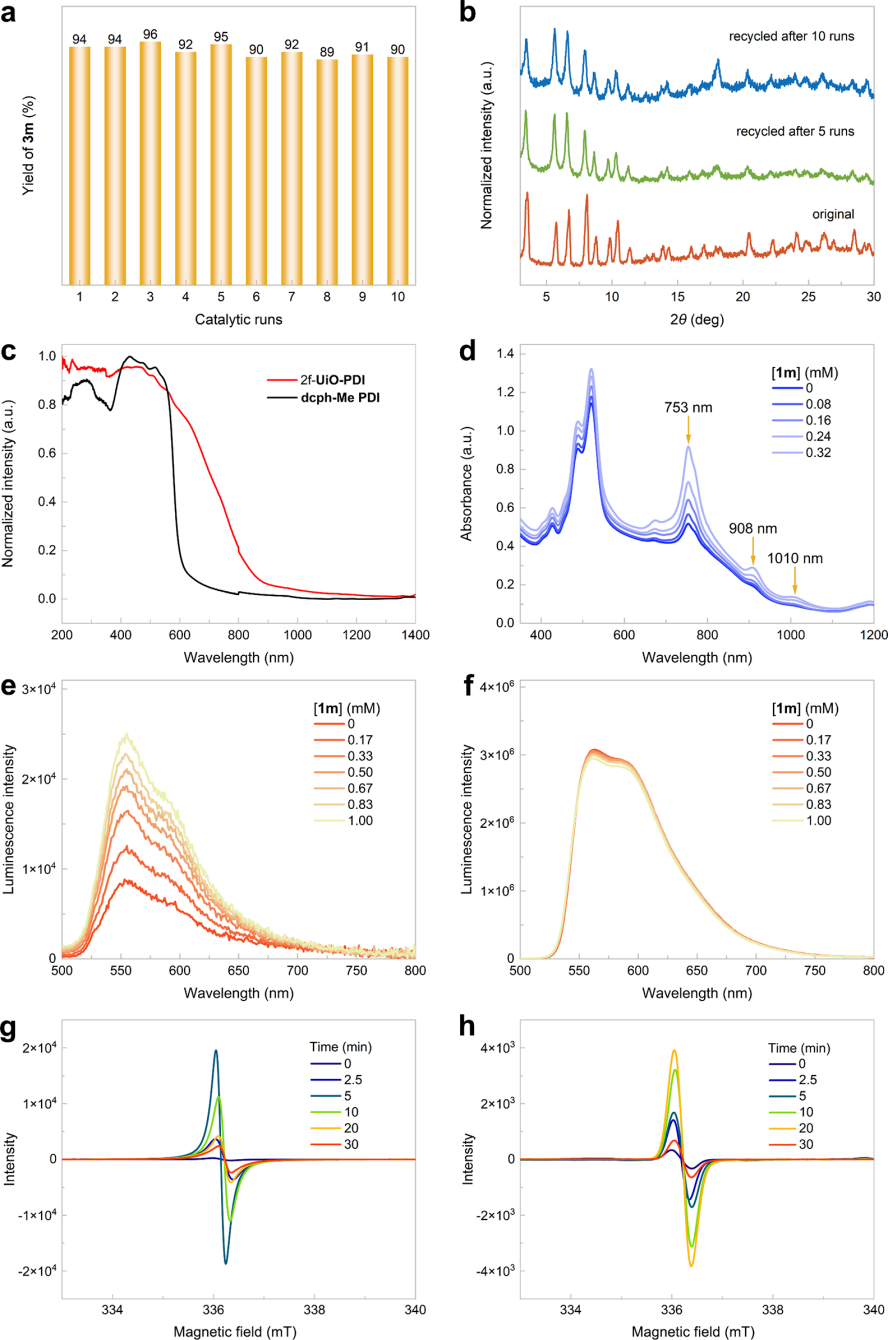
(a) Recycling experiments. (b) PXRD patterns of the original and recycled 2f-**UiO-PDI**. (c) Solid-state absorption spectra of 2f-**UiO-PDI** and **dcph-Me PDI** linker recorded at room temperature. (d) UV–vis–NIR absorption spectra of 2f-**UiO-PDI** and a mixture of 2f-**UiO-PDI** and **1m** in DMF. (e) Luminescence emission spectra of 2f-**UiO-PDI** as a function of the concentration of **1m** upon excitation at 456 nm. (f) Luminescence emission spectra of the **dcph-Me PDI** linker as a function of the concentration of **1m** upon excitation at 456 nm. (g) X-band EPR spectra (9.43 GHz, 298 K) of 2f-**UiO-PDI** and **1m** in DMF under irradiation (456 nm) for 0–30 min. (h) X-band EPR spectra (9.43 GHz, 298 K) of the **dcph-Me PDI** linker and **1m** in DMF under irradiation (456 nm) for 0–30 min.

Impressively, the MOF catalyst used in the photoinduced hydroalkylation to produce **3m** showed excellent recyclability, maintaining full reactivity over ten cycles while preserving consistent PXRD patterns (Figure 3a,b). The SEM images from the recycling experiments revealed the octahedral shape of 2f-**UiO-PDI** after the photocatalytic process (Figure S17). Although both 2f-**UiO-PDI** and its organic linker can absorb blue LED light, the MOF photocatalyst has a wider wavelength range for efficient absorption (Figure 3c). Upon the addition of silicate **1m** to the suspension of 2f-**UiO-PDI** in DMF, significantly enhanced peaks at 753, 908, and 1010 nm were observed, corresponding to the characteristic peaks of PDI^•–^ species generated through SET (Figure 3d).^23,26,27^ In addition to having superior light absorption capabilities compared to tetrachlorophthalimides, photoactive PDI species demonstrate distinctive luminescent properties. While alkyl silicates are typically nonemissive, luminescence steadily increased upon the introduction of silicate **1m** into the MOF suspension, providing compelling evidence of the EDA interaction between 2f-**UiO-PDI** and **1m** (Figure 3e). In contrast, when a **dcph-Me PDI** solution was prepared at the same perylene concentration as the 2f-**UiO-PDI** suspension, no enhanced luminescence was observed following the addition of **1m** (Figure 3f). The immobilization of PDI units on the UiO-type MOF support proves advantageous for facilitating EDA complex formation, likely by mitigating the strong π–π interactions inherent within the PDI units themselves.^50,51^ Based on the ground-state redox potentials determined through cyclic voltammetry measurements (Figures S23 and S24), the photoexcited reduction potential of tetrachloro-functionalized PDI linkers was calculated to be 2.19 V (vs SCE), illustrating their ability to oxidize alkyl silicates and produce PDI^•–^ species along with alkyl radicals upon photoexcitation.^23^ Electron paramagnetic resonance (EPR) spectroscopy was utilized to validate the formation of PDI^•–^ species under blue LED light irradiation (Figure 3g,h).^26,27^ Due to the enhanced EDA interactions between 2f-**UiO-PDI** and silicate **1m**, the EPR signal of PDI^•–^ species exhibited greater intensity and appeared more rapidly compared to that of **dcph-Me PDI** linker, indicating a more efficient SET process within the heterogeneous PDI-based photocatalytic system.^44^

**Figure 4:**
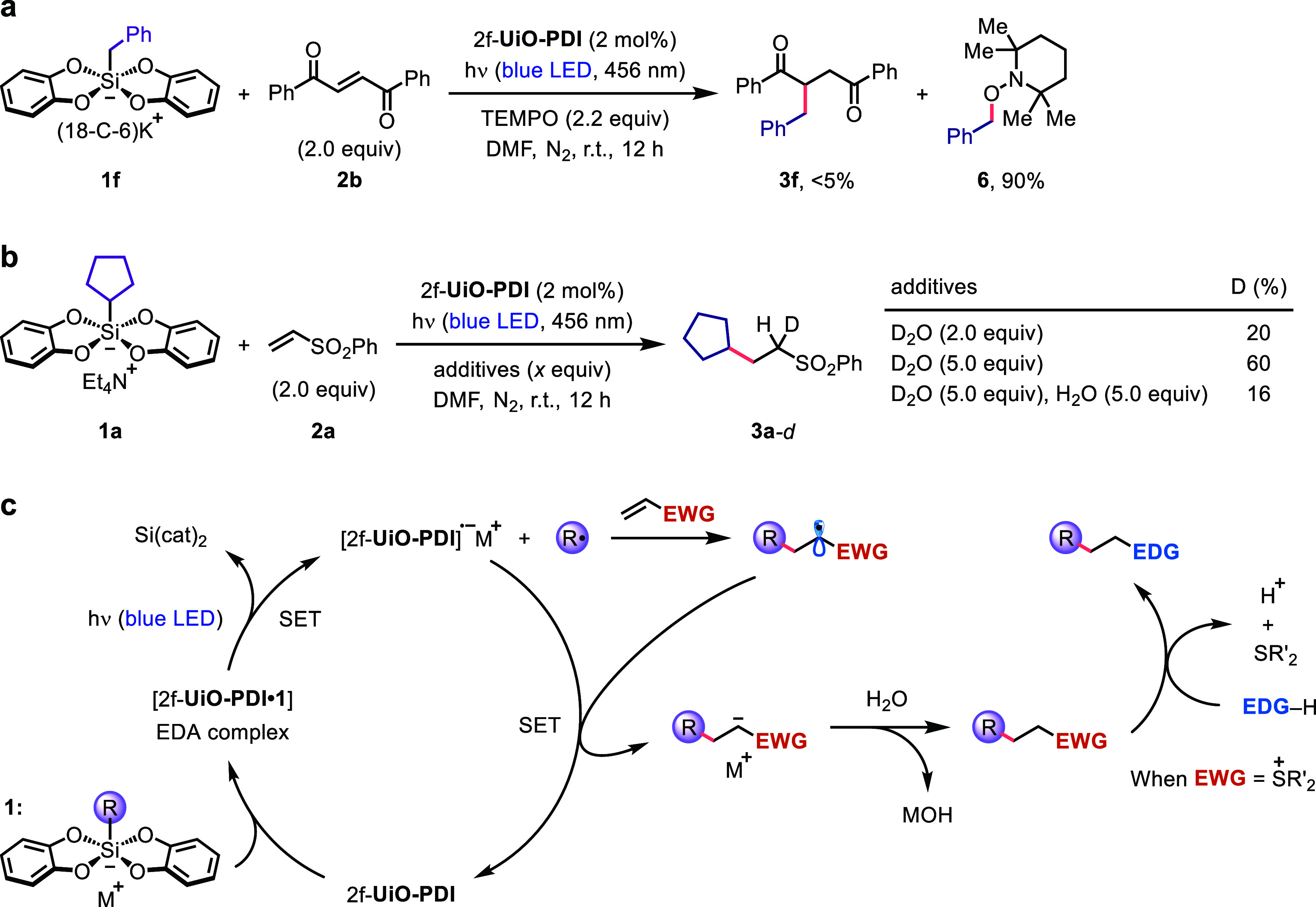
(a) Radical trapping experiment. (b) Deuterium labeling experiments. (c) Plausible mechanism for alkene hydroalkylation with 2f-**UiO-PDI**.

To demonstrate that exposure to light triggers the formation of alkyl radicals from the EDA complex between the PDI linker and alkyl silicates, we employed (2,2,6,6-tetramethylpiperidin-1-yl)oxyl (TEMPO) as a radical trapping reagent to intercept the key intermediate in the heterogeneous photocatalysis (Figure 4a). As expected, the alkene hydroalkylation reaction was completely inhibited in the presence of TEMPO. Instead, radical adduct **6** was obtained in 90% yield, indicating that TEMPO captured the benzylic radical generated from silicate **1m** before it could react with *trans*-1,2-dibenzoylethylene. Furthermore, a series of deuterium labeling experiments were conducted to elucidate the origin of the proton in the alkene functionalization processes (Figure 4b). Increasing the amount of D_2_O from 2 to 5 equiv improved the isotope labeling efficiency from 20 to 60%. The addition of another 5 equiv of water dropped this ratio to 16%, suggesting that the proton source was most likely water in the DMF solution. In the proposed catalytic cycle (Figure 4c), the efficient interaction between the PDI linker and silicate **1** within the MOF pores leads to the formation of EDA complex [2f-**UiO-PDI·1**]. This complex readily undergoes SET upon blue LED light irradiation, resulting in the production of bis(catecholato)silane, PDI^•–^ species, and an alkyl radical. Subsequent atom transfer radical addition with an electron-deficient alkene affords a stabilized electrophilic radical, which can be reduced by the PDI^•–^ species to form a carbanion and regenerate 2f-**UiO-PDI**. The carbanion is then protonated by water in the solution to yield the desired hydroalkylated product. In the presence of a sulfonium leaving group, the product in the heterogeneous photocatalysis can be converted into sulfides, ethers, and amines via an S_N_2 process with various nucleophiles.

## Conclusions

In this study, we described a well-defined crystalline 2-fold interpenetrated UiO-type MOF built with a perylene diimide linker, which acts as a robust heterogeneous photocatalyst for facilitating a series of radical addition reactions via EDA complex photoactivation. The incorporation of the photoactive linker into the rigid three-dimensional framework support significantly enhances the formation of EDA complexes within the MOF pores, as evidenced by photoluminescence and EPR investigations. The remarkable catalyst activity and outstanding recyclability demonstrated in MOF-based heterogeneous photocatalytic systems indicate a promising alternative for advancing the field of organocatalysis toward practical and sustainable organic synthesis.

## Supporting Information Available

Experimental details for the preparation and characterization of MOFs, catalysis, and additional data (PDF)
