# Supplementary material for: Enhancing Electron Donor–Acceptor Complex Photoactivation with a Stable Perylene Diimide Metal–Organic Framework
Source: J Am Chem Soc. 2025 Jan 27;147(10):8350–60. doi: 10.1021/jacs.4c16021 (PMC11912319; doi:10.1021/jacs.4c16021)
Supplement: Supplementary file 1 — ja4c16021_si_001.pdf [file ja4c16021_si_001.pdf]

Supporting Information

Enhancing Electron Donor–Acceptor Complex Photoactivation with a Stable Perylene Diimide Metal–Organic Framework

Xia Wu[1], Ming Cui[1], Kun Wu[2], Jun Guo[1,3], Tianyu Liu[1], Dongyi Liu[1], Zekun Li[1], Puxin Weng[2], Ri-Qin Xia[3], Xiao Xiong[2], Yong-Liang Huang[4], Dan Li[2], and Jian He[1,3,5]*

[1]Department of Chemistry, The University of Hong Kong, Hong Kong 999077, P.R. China.

[2]Guangdong Provincial Key Laboratory of Supramolecular Coordination Chemistry, Jinan University, Guangzhou 510632, P. R. China

[3]State Key Laboratory of Synthetic Chemistry, The University of Hong Kong, Hong Kong 999077, P.R. China.

[4]Department of Medicinal Chemistry, Shantou University Medical College, Shantou 515041, P.R. China

[5]Materials Innovation Institute for Life Sciences and Energy (MILES), HKU-SIRI, Shenzhen 518048, P.R. China.

*Email: jianhe@hku.hk

Table of Contents

1. General Information S–2
2. Synthesis of Linkers S–4
3. Preparation and Characterizations of 2f-**UiO-PDI** and **UiO-PDI** MOFs S–10
4. Synthesis of Substrates S–25
5. Catalytic Applications of Electron Donor–Acceptor Complex Photoactivation S–29
6. Stability Tests of 2f-**UiO-PDI** S–50
7. Mechanistic Studies S–51
8. References S–59
9. NMR Spectra S–60

1. General Information

Unless otherwise noted, materials were either purchased from commercial suppliers and used as received or prepared via literature procedures.

^{1}H NMR spectra were recorded on a Bruker 600 (600 MHz), Bruker 500 (500 MHz) or Bruker 400 (400 MHz) spectrometer in DMSO-d_6 or $CDCl_3$. Chemical shifts were quoted in parts per million (ppm) referenced to 0.0 ppm for tetramethylsilane or 7.26 ppm of $CDCl_3$. The following abbreviations (or combinations thereof) were used to explain multiplicities: s = singlet, d = doublet, t = triplet, q = quartet, m = multiplet, br = broad. Coupling constants, *J*, were reported in Hertz unit (Hz). ^{13}C NMR spectra were recorded on a Bruker 600 (150 MHz), Bruker 500 (125 MHz) or Bruker 400 (100 MHz) spectrometer in DMSO-d_6 or $CDCl_3$ and were fully decoupled by broad band proton decoupling. Chemical shifts were reported in ppm referenced to the center line of a triplet at 77.0 ppm of $CDCl_3$ or the center line of a multiplet at 39.5 ppm of DMSO-d_6.

X-ray diffraction data of single crystals were collected at 100 K on an XtaLab PRO MM007HF DW diffractometer system equipped with a MicroMax-007DW MicroFocus X-ray generator and a Pilatus 200 K silicon diarray detector (Rigaku, Japan, CuKα, λ = 1.54184 Å). Structures were solved by direct methods using the SHELXT program and refined by full matrix least-squares on F^2 with anisotropic displacement parameters for all non-hydrogen atoms using the SHELXL program.

Powder X-ray diffraction (PXRD) patterns were recorded on a Rigaku MiniFlex600 X-ray diffractometer (CuKα, λ = 1.5418 Å), operating at 40 kV and 30 mA. The measurement parameters included a scan speed of 10° min^{-1}, a step size of 0.01°.

Scanning electron microscopy (SEM) and EDS mapping were collected on a Zeiss Gemini 300 scanning electron microscope. TEM and EDS mapping were collected on a FEI Talos F200X scanning transmission electron Microscope with a Super X EDS detector.

The thermogravimetry analyses (TGA) were carried out on NETZSCH STA 449F3 with a heating rate of 10 °C min^{-1} from 30 to 800 °C in N_2 atmosphere.

The ultraviolet-visible absorption spectra were recorded on a Cary 5000 UV−vis−NIR spectrophotometer.

High-resolution EI mass spectra were recorded on a Thermo Scientific DFS Magnetic Sector GC-HRMS system. High-resolution ESI-MS measurements were performed on a Bruker impact II high-resolution LC-QTOF mass spectrometer. Accurate masses from high-resolution mass spectra were reported for the molecular ion $[M]^+$, $[M+H]^+$, $[M]^-$ or $[M+Na]^+$.

Nitrogen gas sorption experiments were performed on a BSD-PM2 specific surface & pore size analyzer. The samples were dried upon heating at 90 °C for 12 h under vacuum before the gas sorption experiments. Surface areas of the materials were obtained based on adsorption data analyzed by Brunauer−Emmett−Teller (BET) methods. The pore size distributions were calculated from the adsorption branches by density functional theory (DFT) method.

Cyclic voltammograms were performed on a CHI760E electro chemistry workstation. Regular three-electrode systems were used. Measurements were recorded in a *N*,*N*-dimethylformamide (DMF) solution of $(Bu_4N)(PF_6)$ (0.1 M) at a scan rate of 100 mV s^{-1} under the protection of N_2 using a glassy carbon disk (d = 0.3 cm) as a working electrode and a platinum plate (1 cm × 1 cm) as a counter electrode. An Ag/AgCl (3 M KCl) electrode was used as a reference electrode in all the experiments, and its potential (−0.54 V versus Fc^+/Fc) was calibrated with the ferrocenium/ferrocene (Fc^+/Fc) redox couple.

Steady-state emission spectra were obtained on an Edinburgh FLS5 spectrophotometer

2. Synthesis of Linkers

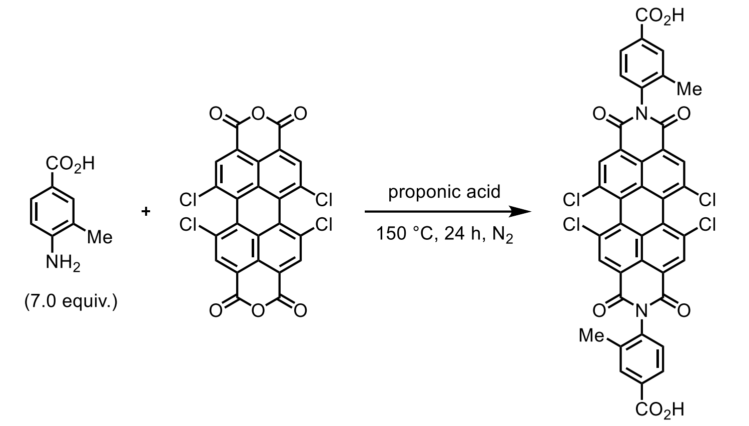

dcph-Me PDI, 87% yield

4,4'-(5,6,12,13-Tetrachloro-1,3,8,10-tetraoxo-1,3,8,10-tetrahydroanthra[2,1,9-*def*:6,5,10-*d'e'f'*]diisoquinoline-2,9-diyl)bis(3-methylbenzoic acid) (**dcph-Me PDI**). To a solution of 1,6,7,12-tetrachloro-3,4,9,10-perylenetetracarboxylic dianhydride (2.0 g, 3.8 mmol, 1.0 equiv.) in propionic acid (40 mL), 4-amino-3-methylbenzoic acid (4.0 g, 26.6 mmol, 7.0 equiv.) was added. After stirring at 150 °C for 24 hours under N_2 atmosphere, the solid was collected by filtration, washed with water, ethanol and dried *in vacuo* to afford 2.6 g (3.3 mmol, 87% yield) of the title compound as a red solid.

^{1}H NMR (DMSO-d_6, 400 MHz): δ 13.11 (s, 2H), 8.65 (s, 4H), 8.02 (s, 2H), 7.94 (d, J = 8.4 Hz, 2H), 7.53 (d, J = 8.0 Hz, 2H), 2.22 (s, 6H).

^{13}C NMR (DMSO-d_6, 100 MHz): δ 166.9, 161.6, 138.6, 136.8, 134.4, 131.8, 131.5, 131.3, 131.1, 129.5, 128.0, 127.9, 127.7, 124.0, 17.1.

HRMS (ESI) calculated for $[C_{40}H_{19}Cl_4N_2O_8]^+$ requires m/z 794.9890, found m/z 794.9897.

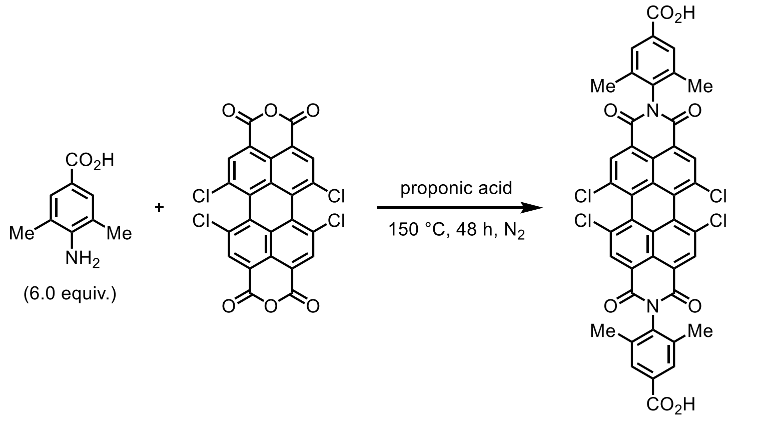

dcph-Me$_2$ PDI, 75% yield

4,4'-(5,6,12,13-Tetrachloro-1,3,8,10-tetraoxo-1,3,8,10-tetrahydroanthra[2,1,9-*def*:6,5,10-*d'e'f'*]diisoquinoline-2,9-diyl)bis(3,5-dimethylbenzoic acid) (**dcph-Me$_2$ PDI**). To a solution of 1,6,7,12-tetrachloro-3,4,9,10-perylenetetracarboxylic dianhydride (0.53 g, 1.0 mmol, 1.0 equiv.) in propionic acid (12 mL), 4-amino-3,5-dimethylbenzoic acid (1.0 g, 6.0 mmol, 6.0 equiv.) was added. After stirring at 150 °C for 48 hours under N_2 atmosphere, the solid was collected by filtration, washed with water, ethanol and dried *in vacuo* to afford 0.62 g (0.75 mmol, 75% yield) of the title compound as a red solid.

^{1}H NMR (DMSO-d_6, 600 MHz): δ 8.69 (s, 4H), 7.84 (s, 4H), 2.17 (s, 12H).

^{13}C NMR (DMSO-d_6, 150 MHz, 398 K): δ 166.3, 160.6, 136.9, 136.0, 134.1, 131.8, 131.0, 129.2, 128.6, 128.2, 128.0, 127.1, 16.9.

HRMS (ESI) calculated for $[C_{42}H_{23}Cl_4N_2O_8]^+$ requires *m*/*z* 823.0203, found *m*/*z* 823.0196.

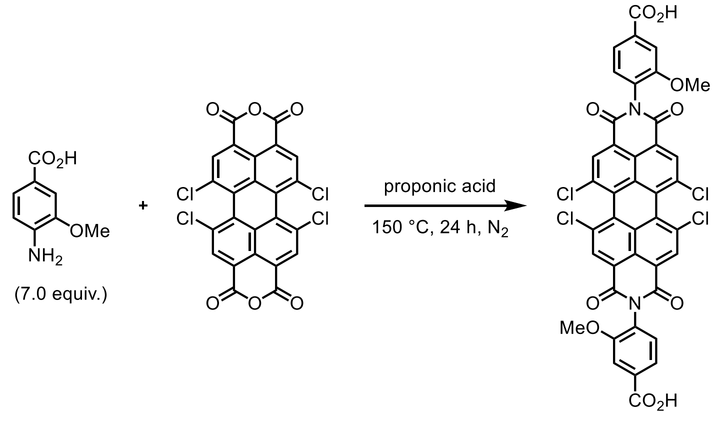

dcph-OMe PDI, 72% yield

4,4'-(5,6,12,13-tetrachloro-1,3,8,10-tetraoxo-1,3,8,10-tetrahydroanthra[2,1,9-*def*:6,5,10-*d'e'f'*]diisoquinoline-2,9-diyl)bis(3-methoxybenzoic acid) (**dcph-OMe PDI**). To a solution of 1,6,7,12-tetrachloro-3,4,9,10-perylenetetracarboxylic dianhydride (0.53 g, 1.0 mmol, 1.0 equiv.) in propionic acid (12 mL), 4-amino-3-methoxybenzoic acid (1.2 g, 7.0 mmol, 7.0 equiv.) was added. After stirring at 150 °C for 24 hours under N_2 atmosphere, the solid was collected by filtration, washed with water, ethanol and dried *in vacuo* to afford 0.60 g (0.72 mmol, 72% yield) of the title compound as a red solid.

^{1}H NMR (DMSO-d_6, 500 MHz): δ 13.21 (s, 2H), 8.64 (s, 4H), 7.98–7.26 (m, 4H), 7.55 (d, *J* = 8.0 Hz, 2H), 3.82 (s, 6H).

^{13}C NMR (DMSO-d_6, 150 MHz, 398 K): δ 166.1, 154.6, 132.9, 129.9, 128.0, 127.93, 127.88, 127.84, 127.4, 121.3, 112.6, 55.8.

HRMS (ESI) calculated for $[C_{40}H_{18}Cl_4N_2O_{10}Na]^+$ requires *m*/*z* 848.9608, found *m*/*z* 848.9605.

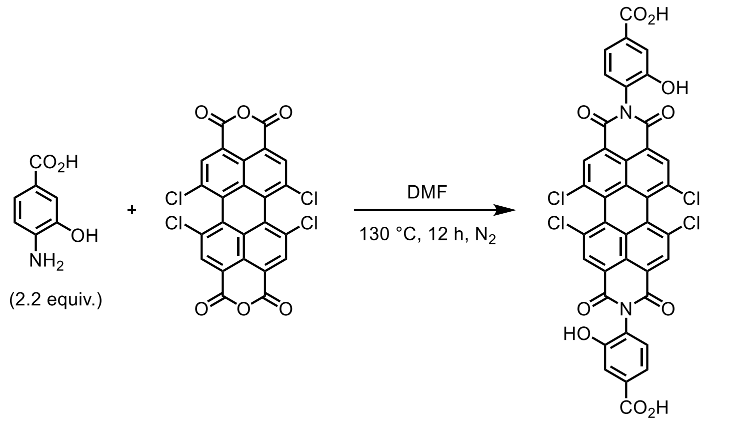

4,4'-(5,6,12,13-tetrachloro-1,3,8,10-tetraoxo-1,3,8,10-tetrahydroanthra[2,1,9-*def*:6,5,10-*d'e'f'*]diisoquinoline-2,9-diyl)bis(3-hydroxybenzoic acid) (**dcph-OH PDI**). To a solution of 1,6,7,12-tetrachloro-3,4,9,10-perylenetetracarboxylic dianhydride (0.53 g, 1.0 mmol, 1.0 equiv.) in DMF (10 mL), 4-amino-3-hydroxybenzoic acid (0.34 g, 2.2 mmol, 2.2 equiv.) was added. After stirring at 130 °C for 12 hours under N_2 atmosphere, the solid was collected by filtration, washed with water, ethanol and dried *in vacuo* to afford 0.67 g (0.84 mmol, 84% yield) of the title compound as a dark red solid.

^{1}H NMR (DMSO-d_6, **500 MHz**): δ 13.02 (s, 2H), 10.20 (s, 2H), 8.62 (s, 4H), 7.58 (s, 2H), 7.56 (d, J = 8.0 Hz, 2H), 7.42 (t, J = 8.0 Hz, 2H).

^{13}C NMR (DMSO-d_6, **150 MHz**): δ 166.9, 161.6, 153.5, 134.4, 132.6, 131.7, 130.5, 129.7, 128.09, 128.05, 126.3, 123.9, 120.2, 117.3.

HRMS (ESI) calculated for $[C_{38}H_{14}Cl_4N_2O_{10}Na]^+$ requires *m*/*z* 820.9295, found *m*/*z* 820.9259.

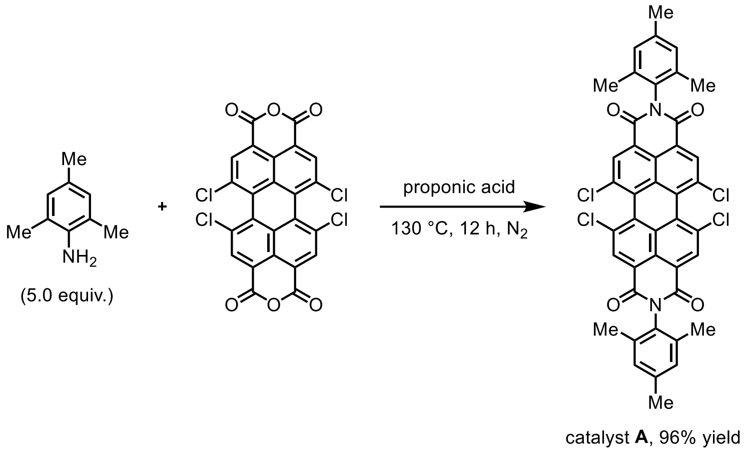

5,6,12,13-Tetrachloro-2,9-dimesitylanthra[2,1,9-*def*:6,5,10-*d'e'f'*]diisoquinoline-1,3,8,10(2*H*,9*H*)-tetraone (catalyst **A**).[9] To a solution of 1,6,7,12-tetrachloro-3,4,9,10-perylenetetracarboxylic dianhydride (0.27 g, 0.50 mmol, 1.0 equiv.) in propionic acid (10 mL), 2,4,6-trimethylaniline (0.34 g, 2.5 mmol, 5.0 equiv.) was added. After stirring at 130 °C for 12 hours under N_2 atmosphere, the solid was collected by filtration, washed with water, ethanol and dried *in vacuo* to afford 0.37 g (0.48 mmol, 96% yield) of the title compound as a red solid.

^{1}H NMR ($CDCl_3$, 600 MHz): δ 8.76 (s, 4H), 7.08 (s, 4H), 2.39 (s, 6H), 2.14 (s, 12H).

^{13}C NMR ($CDCl_3$, 150 MHz): δ 161.7, 139.2, 135.6, 135.0, 133.3, 131.7, 130.3, 129.6, 128.9, 123.9, 123.3, 21.2, 17.8.

HRMS (ESI) calculated for $[C_{42}H_{27}Cl_4N_2O_4]^+$ requires *m*/*z* 763.0719, found *m*/*z* 763.0725.

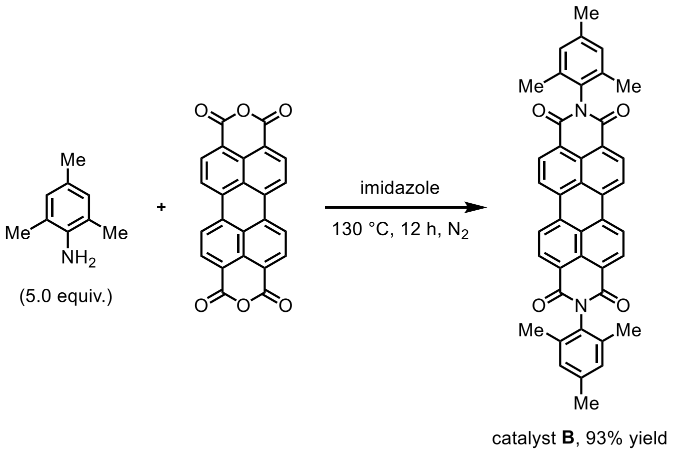

2,9-Dimesitylanthra[2,1,9-*def*:6,5,10-*d'e'f'*]diisoquinoline-1,3,8,10(2*H*,9*H*)-tetraone (catalyst **B**). To a solution of anthra[2,1,9-*def*:6,5,10-*d'e'f'*]diisochromene-1,3,8,10-tetraone (0.39 g, 1.0 mmol, 1.0 equiv.) in imidazole (2.5 g), 2,4,6-trimethylaniline (0.68 g, 5.0 mmol, 5.0 equiv.) was added. After stirring at 130 °C for 12 hours under N_2 atmosphere, the solid was

collected by filtration, washed with 2.0 M HCl, water and dried *in vacuo* to afford 0.58 g (0.93 mmol, 93% yield) of the title compound as a red solid.

^{1}H NMR ($CDCl_3$, 500 MHz): δ 8.79 (d, *J* = 8.0 Hz, 4H), 8.73 (d, *J* = 8.0 Hz, 4H), 7.07 (s, 4H), 2.37 (s, 6H), 2.14 (s, 12H).

^{13}C NMR ($CDCl_3$, 125 MHz): δ 162.8, 138.7, 135.1, 135.0, 132.0, 130.9, 130.1, 129.4, 126.8, 123.4, 123.3, 21.2, 17.8.

HRMS (ESI) calculated for $[C_{42}H_{31}N_2O_4]^+$ requires *m/z* 627.2278, found *m/z* 627.2272.

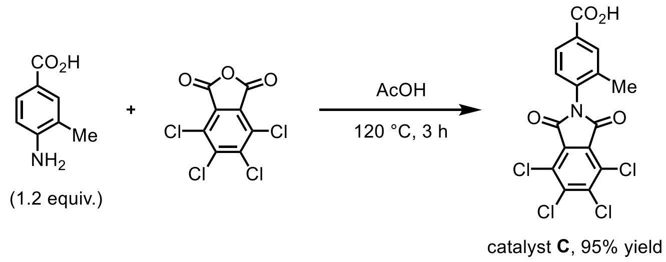

3-Methyl-4-(4,5,6,7-tetrachloro-1,3-dioxoisoindolin-2-yl)benzoic acid (catalyst **C**). To a solution of 4,5,6,7-tetrachloroisobenzofuran-1,3-dione (1.4 g, 5.0 mmol, 1.0 equiv.) in acetic acid (10 mL), 4-amino-3-methylbenzoic acid (0.91 g, 6.0 mmol, 1.2 equiv.) was added. After stirring at 120 °C for 3 hours, the solid was collected by filtration, washed with saturated Na_2CO_3, water, methanol and dried *in vacuo* to afford 1.99 g (4.75 mmol, 95% yield) of the title compound as a white solid.

^{1}H NMR (DMSO-d_6, 400 MHz): δ 13.18 (s, 1H), 7.99 (s, 1H), 7.91 (d, *J* = 8.0 Hz, 1H), 7.49 (d, *J* = 8.0 Hz, 1H), 2.23 (s, 3H).

^{13}C NMR (DMSO-d_6, 100 MHz): δ 166.7, 162.3, 138.4, 137.1, 134.1, 131.9, 131.7, 129.3, 128.6, 128.4, 127.5, 17.4.

HRMS (ESI) calculated for $[C_{16}H_8Cl_4NO_4]^+$ requires *m/z* 417.9202, found *m/z* 417.9194.

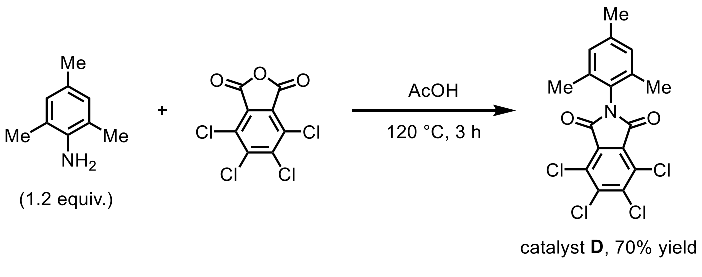

4,5,6,7-Tetrachloro-2-mesitylisoindoline-1,3-dione (catalyst **D**).[10] To a solution of 4,5,6,7-tetrachloroisobenzofuran-1,3-dione (0.57 g, 2.0 mmol, 1.0 equiv.) in acetic acid (4 mL), 2,4,6-trimethylaniline (0.32 g, 2.4 mmol, 1.2 equiv.) was added. After stirring at 120 °C for 3 hours, the solid was collected by filtration, washed with saturated Na_2CO_3, water, methanol and dried *in vacuo* to afford 0.56 g (1.4 mmol, 70% yield) of the title compound as a white solid.

^{1}H NMR ($CDCl_3$, 600 MHz): δ 7.00 (s, 2H), 2.34 (s, 3H), 2.10 (s, 6H).

^{13}C NMR ($CDCl_3$, 150 MHz): δ 162.5, 140.5, 139.8, 136.2, 130.1, 129.4, 127.5, 126.3, 21.1, 17.9.

HRMS (ESI) calculated for $[C_{17}H_{12}Cl_4NO_2]^+$ requires *m*/*z* 401.9617, found *m*/*z* 401.9620.

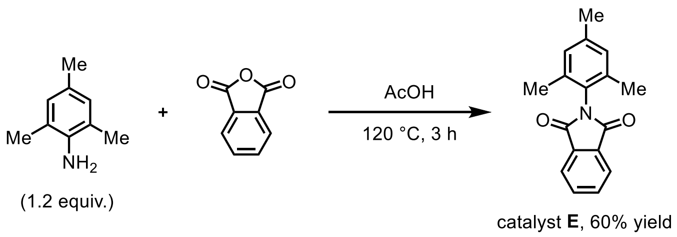

2-Mesitylisoindoline-1,3-dione (catalyst **E**). To a solution of isobenzofuran-1,3-dione (0.30 g, 2.0 mmol, 1.0 equiv.) in acetic acid (4 mL), 2,4,6-trimethylaniline (0.32 g, 2.4 mmol, 1.2 equiv.) was added. After stirring at 120 °C for 3 hours, the solid was collected by filtration, washed with saturated Na_2CO_3, water, methanol and dried *in vacuo* to afford 0.32 g (1.2 mmol, 60% yield) of the title compound as a white solid.

^{1}H NMR ($CDCl_3$, 600 MHz): δ 7.98–7.94 (m, 2H), 7.82–7.78 (m, 2H), 7.01 (s, 2H), 2.33 (s, 3H), 2.12 (s, 6H).

^{13}C NMR ($CDCl_3$, 150 MHz): δ 167.4, 139.4, 136.4, 134.2, 132.0, 129.3, 127.0, 123.7, 21.1, 18.0.

HRMS (ESI) calculated for $[C_{17}H_{16}NO_2]^+$ requires *m*/*z* 266.1176, found *m*/*z* 266.1187 .

3. Preparation and Characterizations of 2f-UiO-PDI and UiO-PDI MOFs

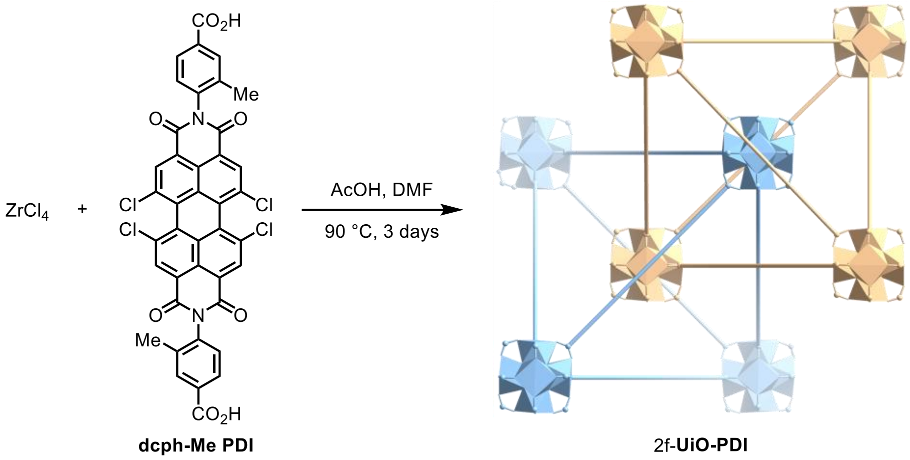

$ZrCl_4$ (4.2 mg, 0.018 mmol), **dcph-Me PDI** (14.3 mg, 0.018 mmol) and acetic acid (0.35 mL) were ultrasonically dispersed in dimethylformamide (3.3 mL). The resulting solution was heated to 90 °C in a 5-mL glass vial for 3 days. After cooling to room temperature, the supernatant was decanted and the solid was washed five times with dimethylformamide and ethanol. 2f-**UiO-PDI** was then collected by filtration and dried in air to give a dark red solid in 48% yield (8.8 mg) based on TGA (Figure S12).

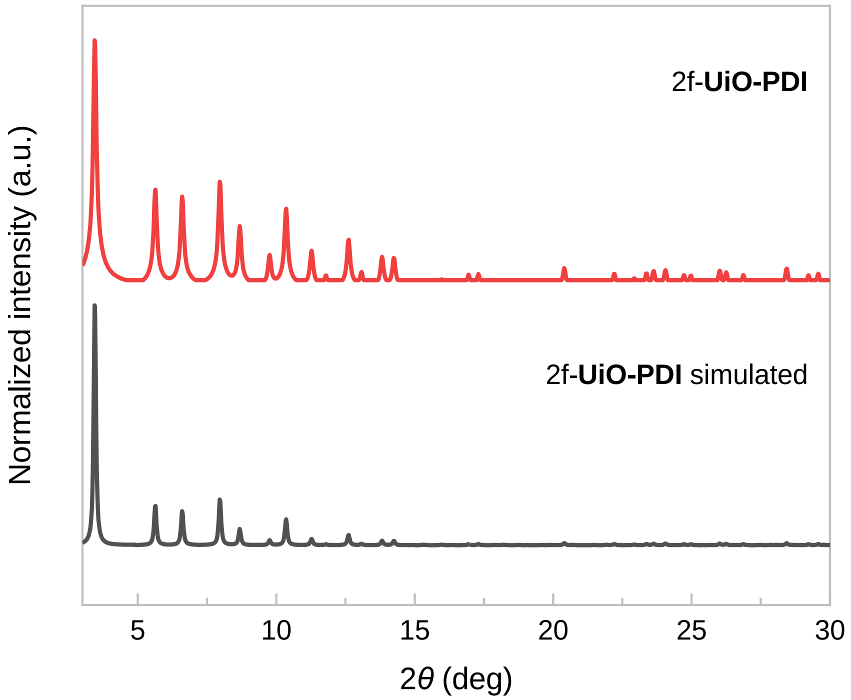

Figure S1. Comparing PXRD patterns of 2f-**UiO-PDI** with simulation of 2f-**UiO-PDI**.

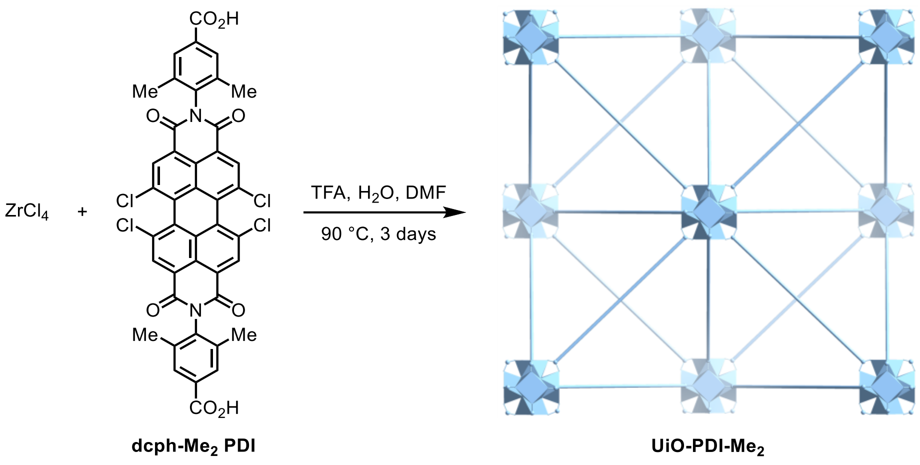

$ZrCl_4$ (4.2 mg, 0.018 mmol), **dcph-Me$_2$ PDI** (14.8 mg, 0.018 mmol), trifluoroacetic acid (0.4 mL) and H_2O (0.2 mL) were ultrasonically dispersed in dimethylformamide (3.2 mL). The resulting solution was heated to 90 °C in a 5-mL glass vial for 3 days. After cooling to room temperature, the supernatant was decanted and the solid was washed five times with dimethylformamide and ethanol. **UiO-PDI-Me$_2$** was then collected by filtration and dried in air to give a dark red solid.

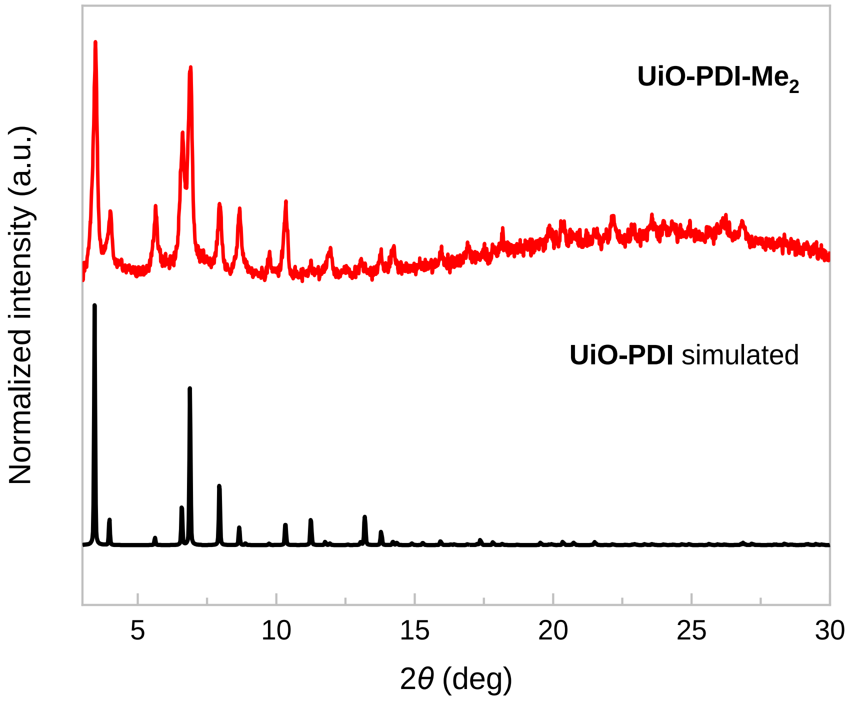

Figure S2. Comparing PXRD patterns of **UiO-PDI-Me$_2$** with simulation of **UiO-PDI**.

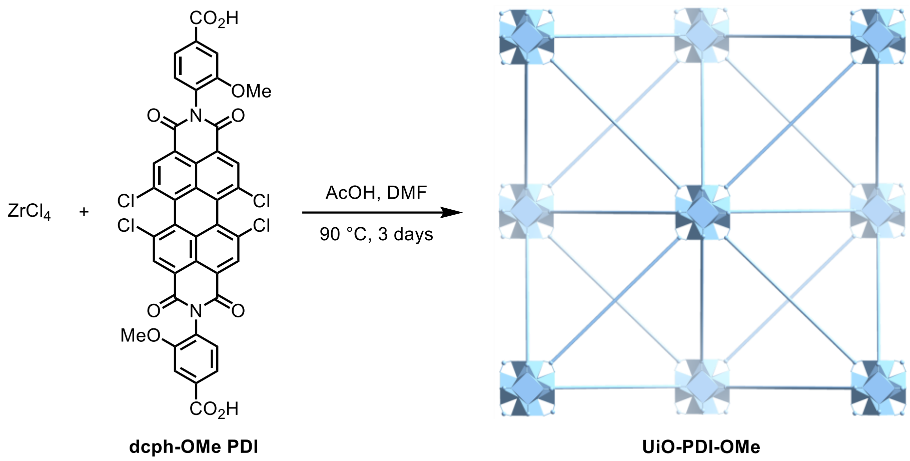

$ZrCl_4$ (4.2 mg, 0.018 mmol), **dcph-OMe PDI** (14.9 mg, 0.018 mmol) and acetic acid (0.17 mL) were ultrasonically dispersed in dimethylformamide (3.3 mL). The resulting solution was heated to 100 °C in a 5-mL glass vial for 3 days. After cooling to room temperature, the supernatant was decanted and the solid was washed five times with dimethylformamide and ethanol. **UiO-PDI-OMe** was then collected by filtration and dried in air to give a dark red solid.

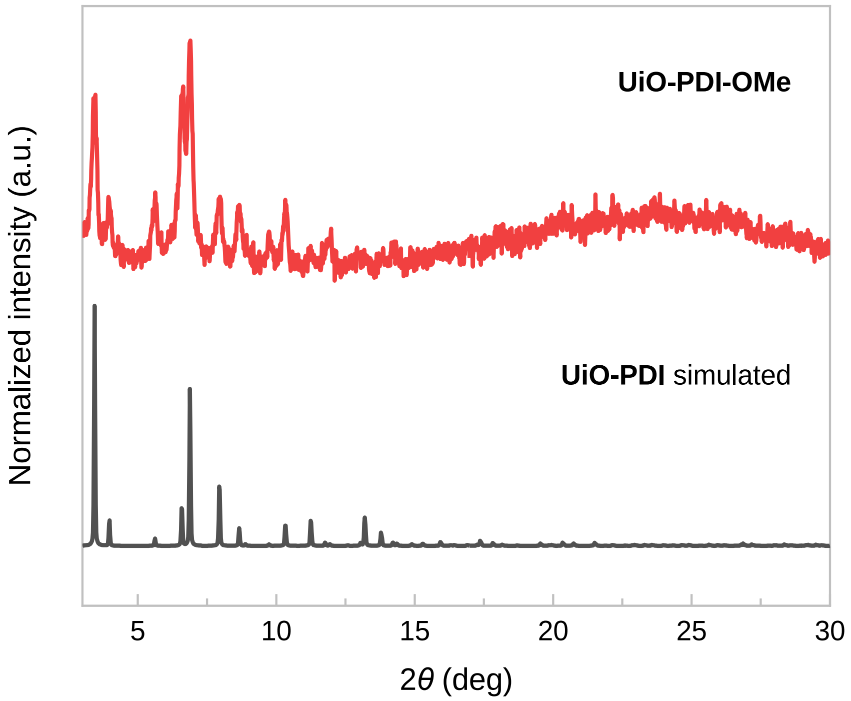

Figure S3. Comparing PXRD patterns of **UiO-PDI-OMe** with simulation of **UiO-PDI**.

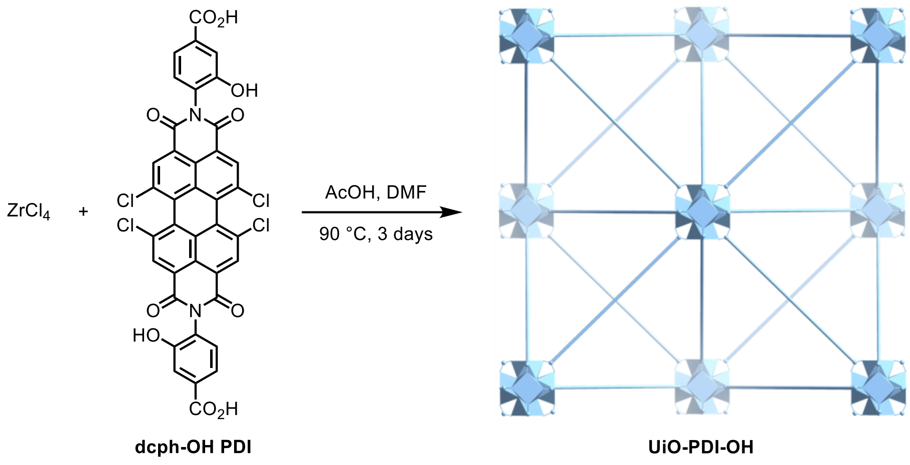

$ZrCl_4$ (4.2 mg, 0.018 mmol), **dcph-OH PDI** (14.4 mg, 0.018 mmol) and acetic acid (0.4 mL) were ultrasonically dispersed in dimethylformamide (1.6 mL). The resulting solution was heated to 90 °C in a 5-mL glass vial for 3 days. After cooling to room temperature, the supernatant was decanted and the solid was washed five times with dimethylformamide and ethanol. **UiO-PDI-OH** was then collected by filtration and dried in air to give a dark red solid.

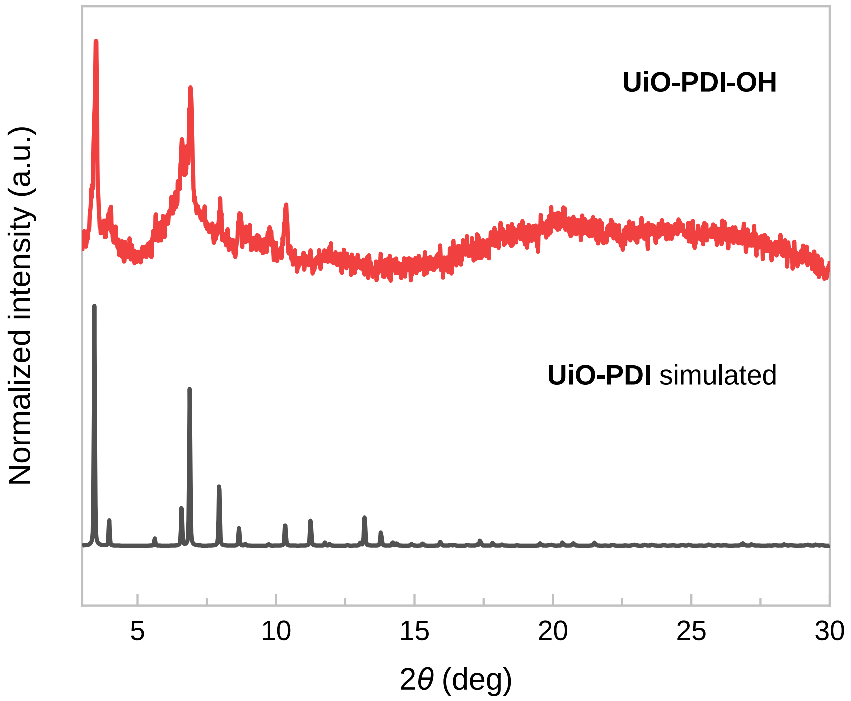

Figure S4. Comparing PXRD patterns of **UiO-PDI-OH** with simulation of **UiO-PDI**.

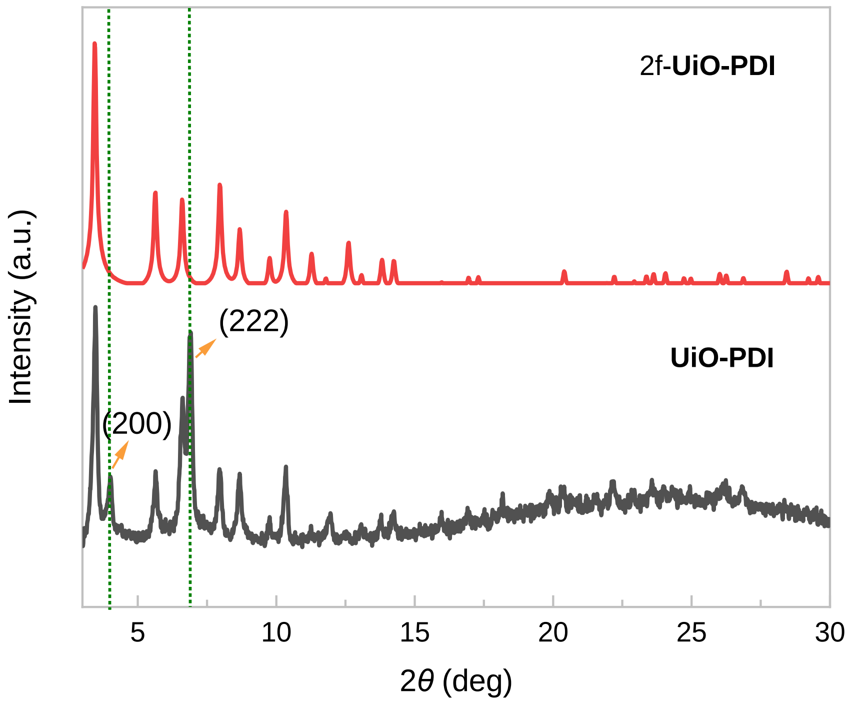

Figure S5. Comparing PXRD patterns of 2f-**UiO-PDI** with **UiO-PDI**.

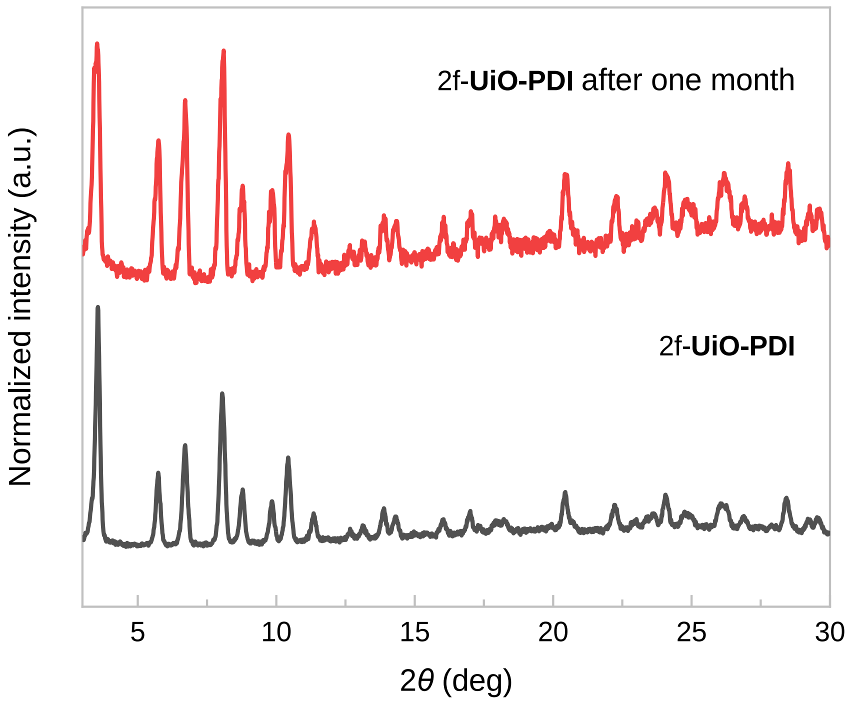

Figure S6. Comparing PXRD patterns of 2f-**UiO-PDI** with 2f-**UiO-PDI** after one month.

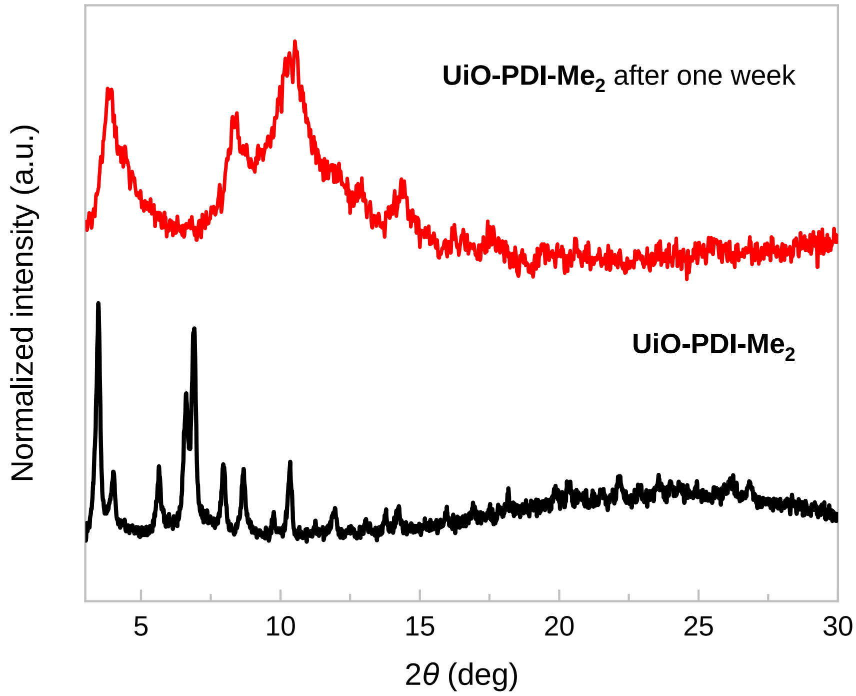

Figure S7. Comparing PXRD patterns of **UiO-PDI-Me$_2$** with **UiO-PDI-Me$_2$** after one week.

X-Ray Crystallography

Table S1. Crystal Data and Structure Refinement for 2f-UiO-PDI

Identification code	2f-**UiO-PDI**
CCDC number	2401830
Empirical formula	$C_{80}H_{36}Cl_8N_4O_{20}Zr_2$
Formula weight	1839.17
Temperature/K	100.00
Crystal system	Cubic
Space group	$Fd\bar{3}m$
a/Å	44.3536(4)
b/Å	44.3536(4)
c/Å	44.3536(4)
α/°	90
β/°	90
γ/°	90
Volume/Å^3	87254(2)
Z	12
ρ_{calc}/(g cm^{-3})	0.420
μ/mm^{-1}	1.437
F(000)	11040.0
Crystal size/mm^3	0.156 × 0.143 × 0.052
Radiation	CuKα (λ = 1.54184)
2θ range for data collection/°	6.61 to 155.412
Index ranges	$-37 \leq h \leq 28$, $-51 \leq k \leq 55$, $-40 \leq l \leq 51$
Reflections collected	26156
Independent reflections	4238 [R_{int} = 0.0919, R_{sigma} = 0.0522]
Data/restraints/parameters	4238/1470/431
Goodness-of-fit on F^2	0.972
Final R indexes [I >= 2σ (I)]	R_1 = 0.1051, wR_2 = 0.2482
Final R indexes [all data]	R_1 = 0.1400, wR_2 = 0.2739
Largest diff. peak/hole/eÅ^{-3}	0.55/−0.56

$R_1 = \sum||F_o|-|F_c||/\sum|F_o|$. $wR_2 = [\sum w(F_o^2-F_c^2)^2/\sum w(F_o^2)^2]^{1/2}$

Table S2. Bond Lengths in 2f-UiO-PDI

Bond	Length/Å	Bond	Length/Å
Zr1–Zr1[1]	3.4878(11)	C16–C18	1.358(5)
Zr1–Zr1[2]	3.4878(11)	C17–C19	1.338(7)
Zr1–Zr1[3]	3.4878(11)	C18–C19	1.334(7)
Zr1–Zr1[4]	3.4878(11)	C18–C37	1.405(8)
Zr1–O1	2.069(3)	C19–Cl27	1.701(7)
Zr1–O1[5]	2.069(3)	O5–C20	1.213(5)
Zr1–O2[5]	2.185(4)	C20–C21	1.491(9)
Zr1–O2	2.185(4)	C20–O6	1.223(5)
Zr1–O6	2.165(13)	C21–C22	1.3900
O3–C1	1.217(5)	C21–C26	1.3900
C1–C2	1.482(9)	C22–C23	1.3900
C1–O4	1.218(5)	C23–C24	1.3900
C2–C3	1.3900	C23–C40	1.520(10)
C2–C7	1.3900	C24–C25	1.3900
C3–C4	1.3900	C24–N2	1.522(8)
C4–C5	1.3900	C25–C26	1.3900
C4–C39	1.514(10)	C27–C28	1.3900
C5–C6	1.3900	C27–N2	1.3900
C5–N1	1.506(7)	C27–O10	1.241(19)
C6–C7	1.3900	C28–C29	1.3900
C8–N1	1.3900	C28–C32	1.342(10)
C8–C12	1.3900	C29–C30	1.3900
C8–O9	1.271(18)	C29–C35	1.356(5)
N1–C9	1.3900	C30–C31	1.3900
C9–C10	1.3900	C30–C36	1.346(7)
C9–O8	1.259(19)	C31–N2	1.3900
C10–C11	1.3900	C31–O7	1.253(18)
C10–C17	1.365(6)	C32–C33	1.338(7)
C11–C12	1.3900	C33–C34	1.333(10)
C11–C16	1.390(6)	C33–Cl6	1.705(7)
C12–C13	1.337(10)	C34–C35	1.370(6)
C13–C14	1.344(10)	C35–C37	1.360(5)
C14–C15	1.349(10)	C36–C38	1.352(10)
C14–Cl6A	1.702(10)	C37–C38	1.359(10)
C15–C16	1.365(5)	C38–Cl1	1.722(7)
C15–C34	1.397(8)		

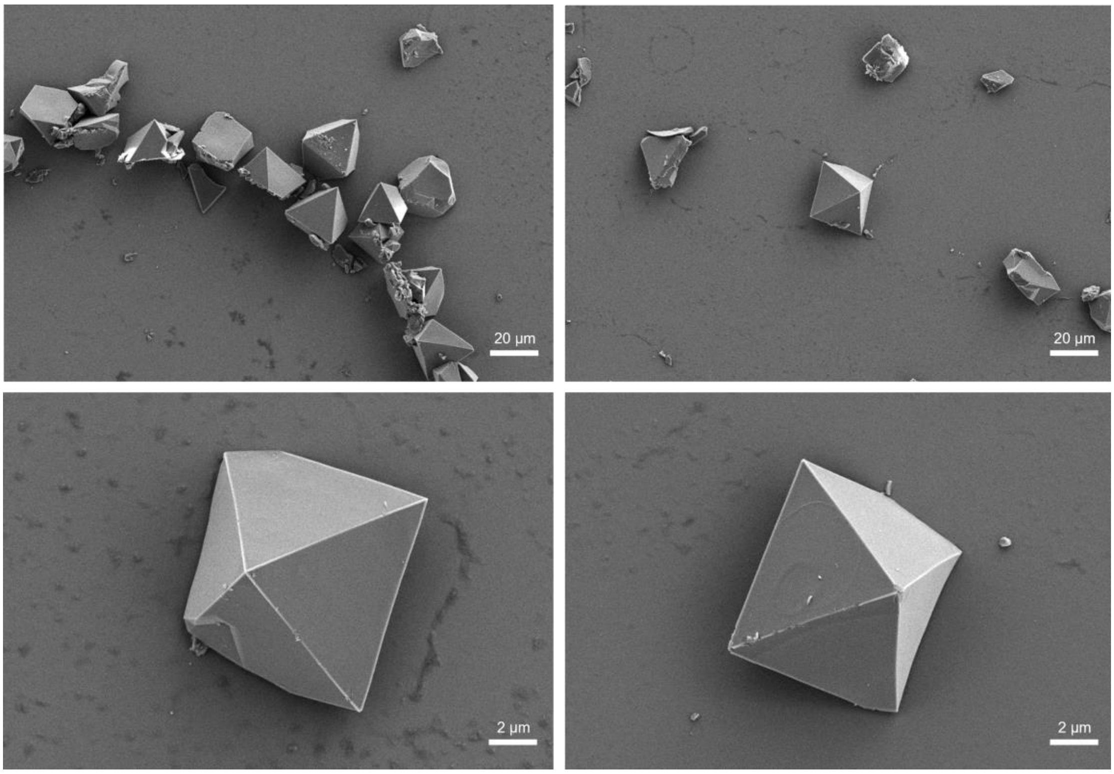

Figure S8. SEM images of 2f-**UiO-PDI**.

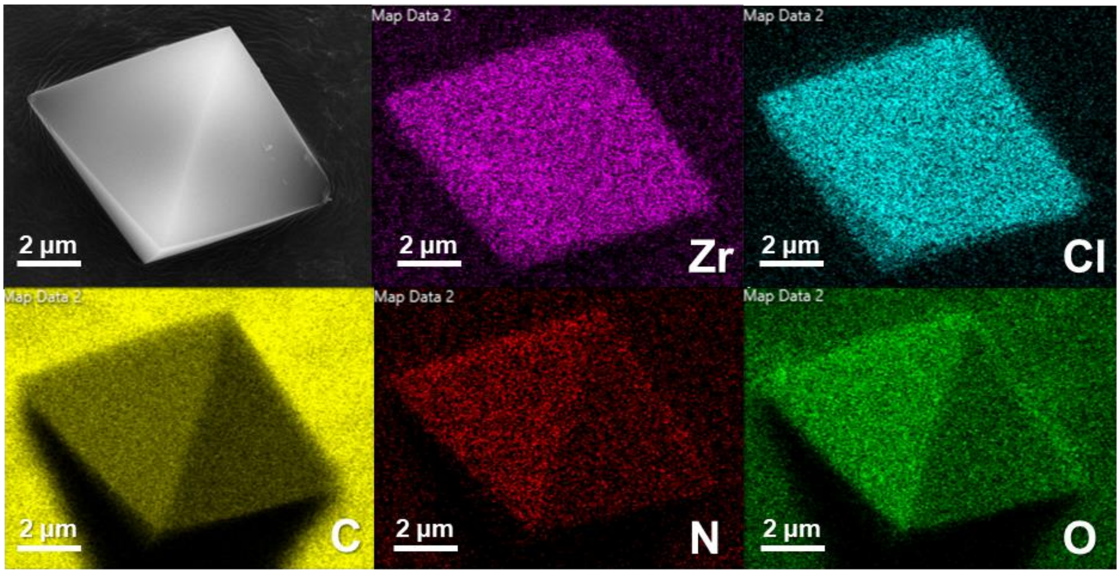

Figure S9. SEM image and elemental mapping of 2f-**UiO-PDI**. EDS mapping images of Zr, Cl, C, N, and O are represented in magenta, cyan, yellow, red, and green. Scale bar, 2 µm.

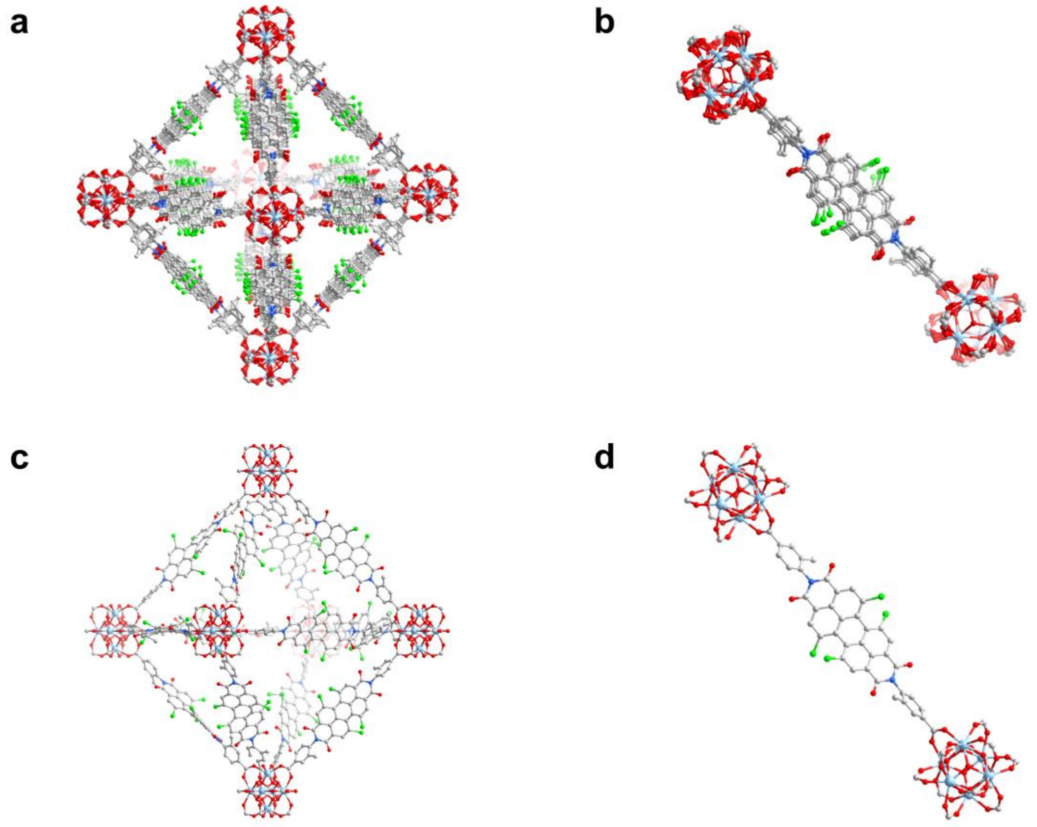

Figure S10. (a,b) Crystal structure of 2f-**UiO-PDI** with crystallographic disorder. (c,d) Crystal structure of 2f-**UiO-PDI** in a simplified version (Zr, pale blue; Cl, green; C, grey; O, red; N, blue).

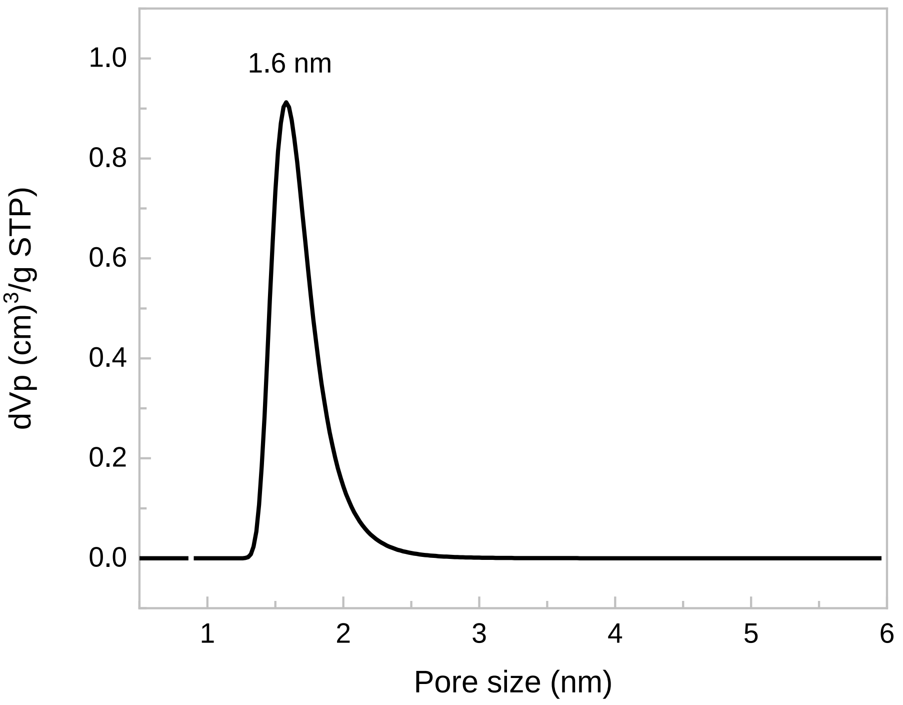

Figure S11. Pore size distribution of 2f-**UiO-PDI**.

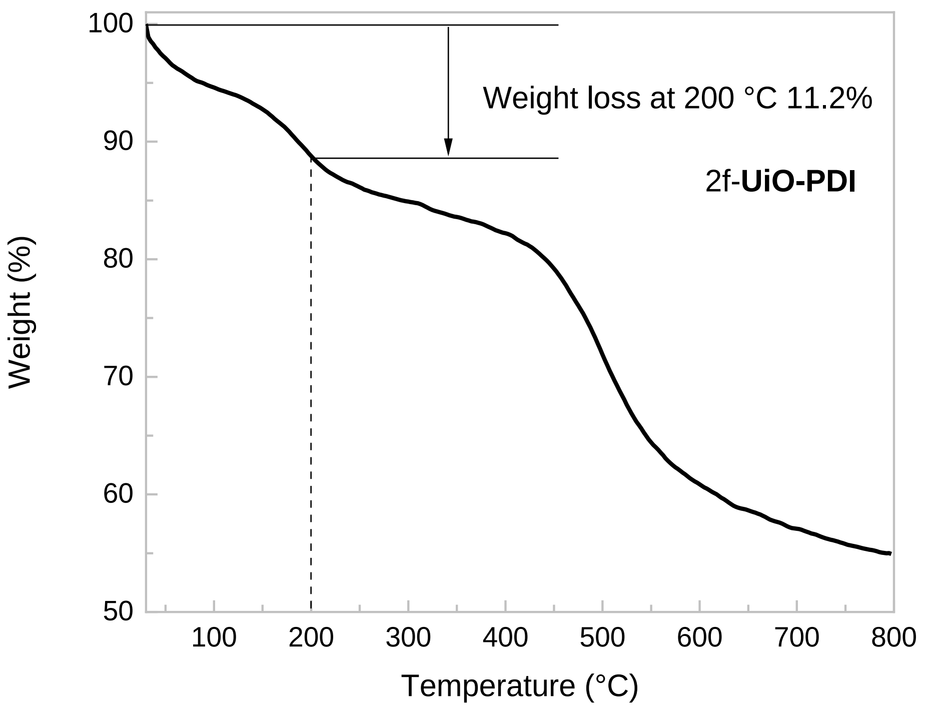

Figure S12. TGA plot of 2f-**UiO-PDI** under N_2 atmosphere.

According to TGA, 2f-**UiO-PDI** contained solvents in 11.2% weight.

The chemical formula of 2f-**UiO-PDI** was determined to be [$Zr_6O_4(OH)_4$(**dcph-Me PDI**)$_6$] (Mw = 5445.6).

To run a catalytic reaction (on a 0.1-mmol scale) with 2 mol% of the 2f-**UiO-PDI** catalyst:

the amount of 2f-**UiO-PDI** = (5445.6/6 × 0.1 mmol × 2 mol%)/(1 – 11.2%) = 2.0 mg

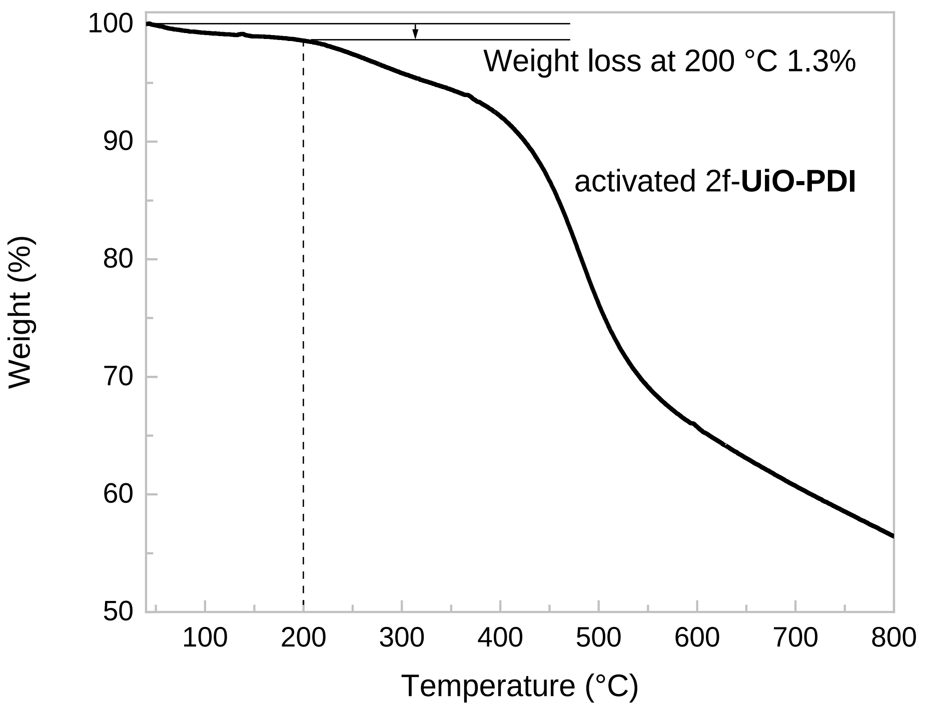

Figure S13. TGA plot of activated 2f-**UiO-PDI** under N_2 atmosphere.

According to TGA, the activated 2f-**UiO-PDI** contained solvents in 1.3% weight.

The chemical formula of 2f-**UiO-PDI** was determined to be [$Zr_6O_4(OH)_4$(**dcph-Me PDI**)$_6$] (Mw = 5445.6).

To run a catalytic reaction (on a 0.1-mmol scale) with 2 mol% of the activated 2f-**UiO-PDI** catalyst:

the amount of activated 2f-**UiO-PDI** = (5445.6/6 × 0.1 mmol × 2 mol%)/(1 – 1.3%) = 1.8 mg

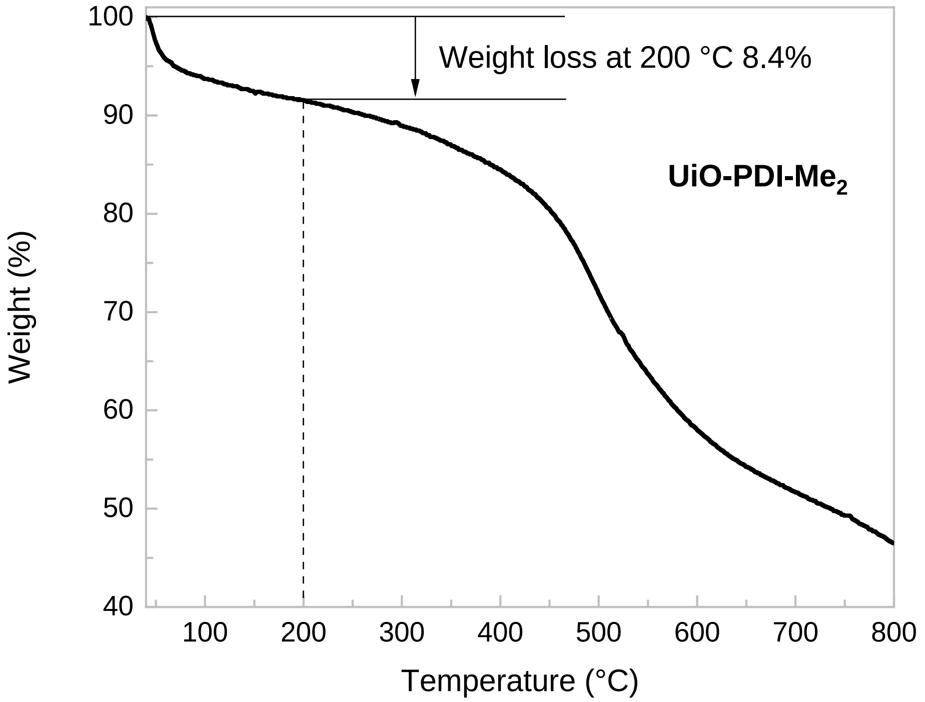

Figure S14. TGA plot of **UiO-PDI-Me$_2$** under N_2 atmosphere.

According to TGA, **UiO-PDI-Me$_2$** contained solvents in 8.4% weight.

The chemical formula of **UiO-PDI-Me$_2$** was determined to be [$Zr_6O_4(OH)_4$(**dcph-Me$_2$ PDI**)$_6$] (Mw = 5613.9).

To run a catalytic reaction (on a 0.1-mmol scale) with 2 mol% of the **UiO-PDI-Me$_2$** catalyst:

the amount of **UiO-PDI-Me$_2$** = (5613.9/6 × 0.1 mmol × 2 mol%)/(1 – 8.4%) = 2.0 mg

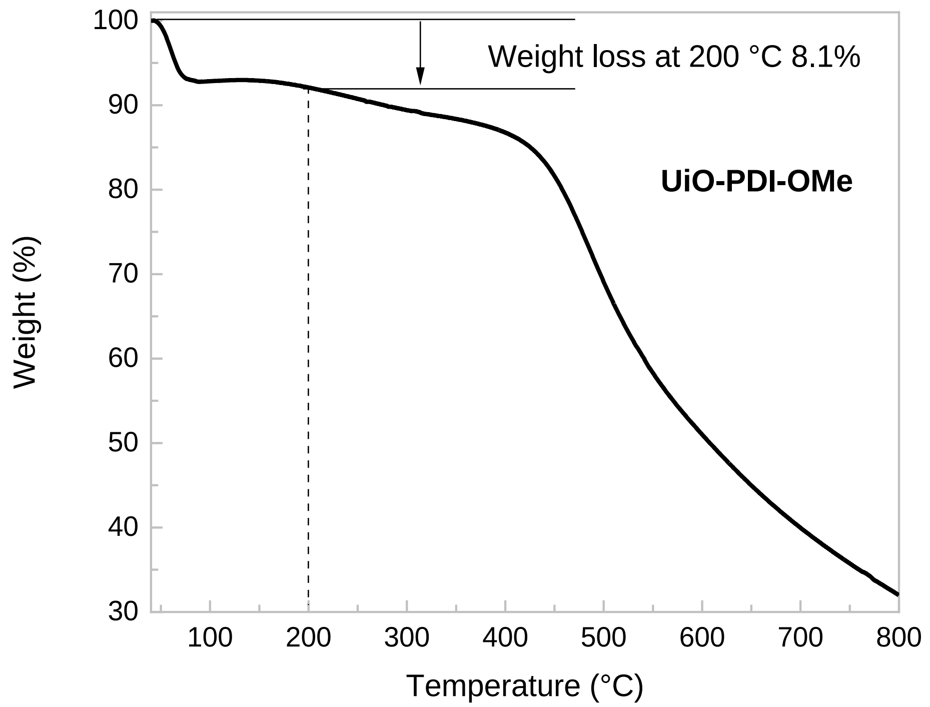

Figure S15. TGA plot of **UiO-PDI-OMe** under N_2 atmosphere.

According to TGA, **UiO-PDI-OMe** contained solvents in 8.1% weight.

The chemical formula of **UiO-PDI-OMe** was determined to be $[Zr_6O_4(OH)_4($**dcph-OMe PDI**$)_6]$ (Mw = 5637.6).

To run a catalytic reaction (on a 0.1-mmol scale) with 2 mol% of the **UiO-PDI-OMe** catalyst: the amount of **UiO-PDI-OMe** = (5637.6/6 × 0.1 mmol × 2 mol%)/(1 – 8.1%) = 2.0 mg

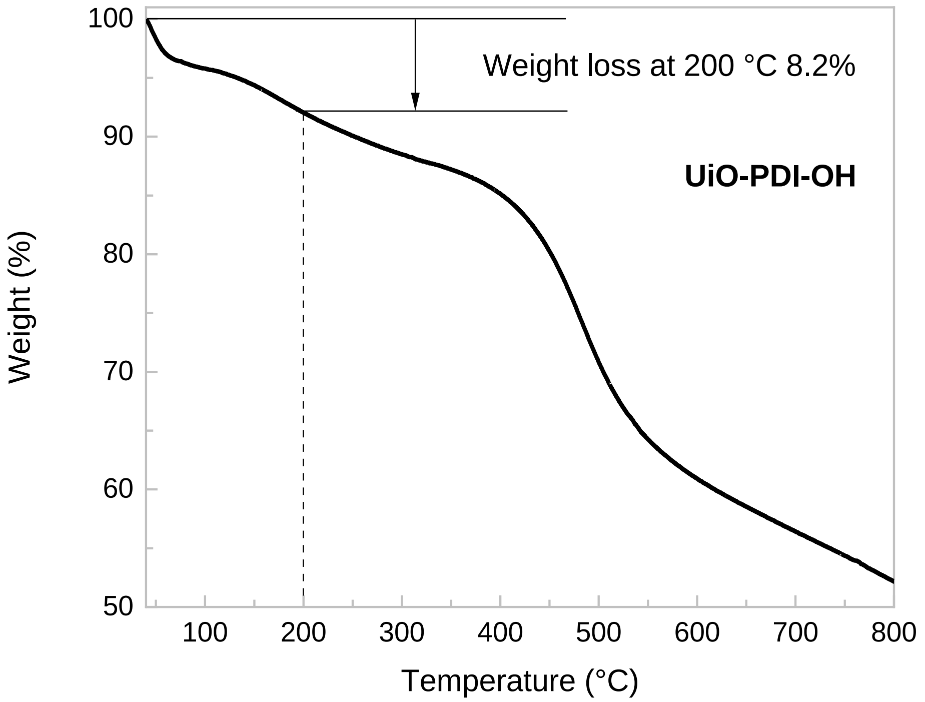

Figure S16. TGA plot of **UiO-PDI-OH** under N_2 atmosphere.

According to TGA, **UiO-PDI-OH** contained solvents in 8.2% weight.

The chemical formula of **UiO-PDI-OH** was determined to be [$Zr_6O_4(OH)_4$(**dcph-OH PDI**)$_6$] (Mw = 5469.3).

To run a catalytic reaction (on a 0.1-mmol scale) with 2 mol% of the **UiO-PDI-OH** catalyst:

the amount of **UiO-PDI-OH** = (5469.3/6 × 0.1 mmol × 2 mol%)/(1 – 8.2%) = 2.0 mg

4. Synthesis of Substrates

The silicates **1a**, **1c**, **1f–1m** were synthesized according to the literature.[1]

The alkenes **2d**,[2] **2e**,[3] **2g**,[2] **2j**,[4] **2k**,[5] **2l**,[6] dineopentyl(vinyl)sulfonium triflate **4**[7] were synthesized according to the literature.

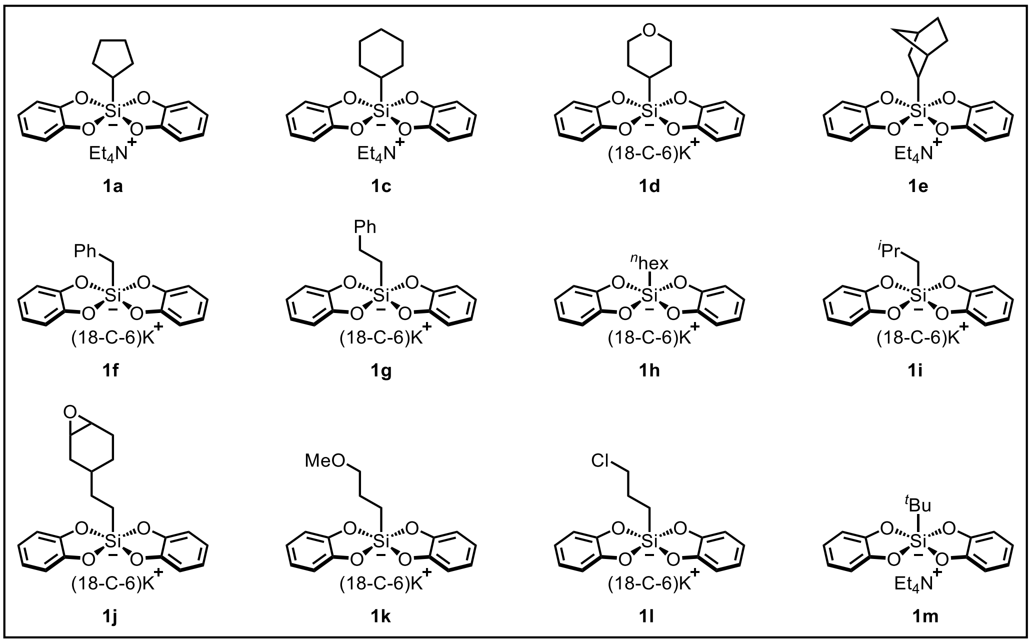

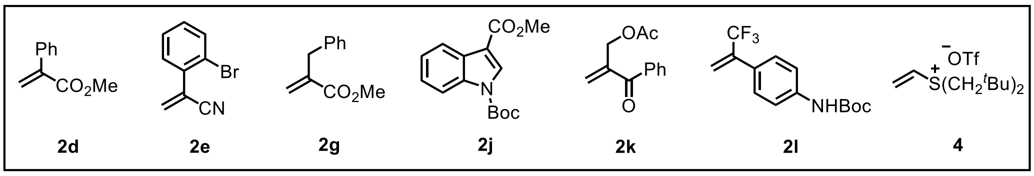

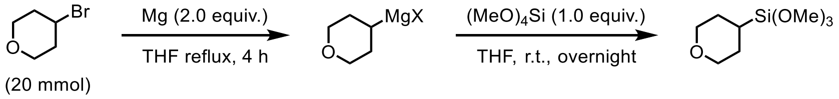

Trimethoxy(tetrahydro-2*H*-pyran-4-yl)silane. (Tetrahydro-2*H*-pyran-4-yl)magnesium bromide was prepared according to a standard Grignard procedure starting with magnesium (0.53 g, 22 mmol, 1.1 equiv.) and 4-bromotetrahydro-2*H*-pyran (3.3 g, 20 mmol, 1.0 equiv.). A 10-mL flame-dried Schlenk tube equipped with a magnetic stir bar was charged with tetramethoxysilane (3.0 g, 20 mmol, 1.0 equiv.) and THF (40 mL). The fresh prepared (tetrahydro-2*H*-pyran-4-yl)magnesium bromide was added dropwise by a syringe over 20 minutes at room temperature, and then the reaction mixture was stirred for 20 hours under reflux. After 20 hours, the reaction mixture was filtered through a pad of celite, and the filtrate was

concentrated under reduced pressure. The crude product was purified by distillation to afford the corresponding trimethoxysilane as a colorless liquid. This compound was used directly in the next step.

^{1}H NMR ($CDCl_3$, 400 MHz): δ 4.02–3.89 (m, 2H), 3.59 (s, 9H), 3.40–3.30 (m, 2H), 1.77–1.52 (m, 4H), 1.33–1.23 (m, 1H).

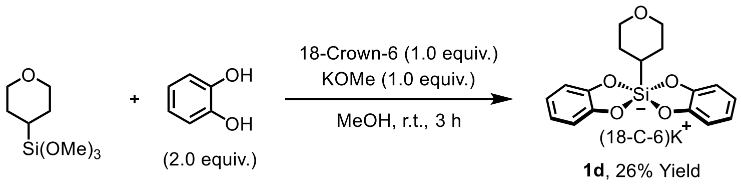

Potassium (18-Crown-6) bis(catecholato)-(tetrahydro-2*H*-pyran-4-yl)silicate (1d). To a stirred solution of catechol (0.44 g, 4.0 mmol, 2.0 equiv.) in dry methanol (20 mL) was added 18-Crown-6 (0.53 g, 2.0 mmol, 1.0 equiv.). After dissolution of the crown ether, trimethoxy(tetrahydro-2*H*-pyran-4-yl)silane (0.41 g, 2.0 mmol, 1.0 equiv.) was added, followed by a solution of KOMe (0.14 g, 2.0 mmol, 1.0 equiv.) in MeOH (5.0 mL). The reaction mixture was stirred for 3 hours at room temperature and the solvent was removed under reduced pressure. The residue was dissolved in the minimum volume of acetone, and then diethyl ether was added until a cloudy solution was observed. The flask was placed in a freezer at –20 °C overnight. The precipitate was collected by filtration, washed with diethyl ether and dried under vacuum to afford the corresponding silicate as an off-pink solid.

^{1}H NMR ($CDCl_3$, 400 MHz): δ 6.94–6.88 (m, 2H), 6.79–6.75 (m, 2H), 6.75–6.70 (m, 2H), 6.58–6.54 (m, 2H), 3.82–3.72 (m, 2H), 3.48 (s, 24H), 3.29–3.16 (m, 2H), 1.70–1.58 (m, 2H), 1.57–1.44 (m, 2H), 0.92–0.78 (m, 1H).

^{13}C NMR ($CDCl_3$, 100 MHz): δ 150.7, 145.0, 120.3, 117.8, 116.0, 110.4, 69.9, 28.3, 25.9.

HRMS (ESI) calculated for $[C_{17}H_{17}O_5Si]^-$ requires *m/z* 329.0851, found *m/z* 329.0851.

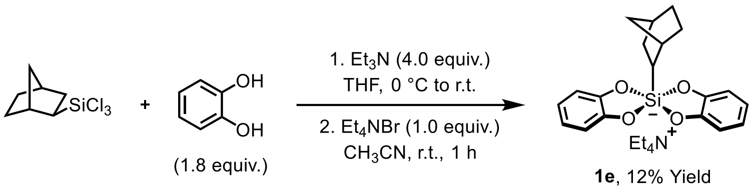

Tetraethylammonium *exo*-2-bicyclo[2.2.1]heptylbis(catecholato)silicate (1e). (Bicyclo[2.2.1]heptan-2-yl)trichlorosilane was synthesized according to the literature.[8] To a stirred solution of catechol (0.99 g, 9.0 mmol, 1.8 equiv.) in dry THF (20 mL) was added triethylamine (2.0 g, 20 mmol, 4.0 equiv.). The reaction mixture was cooled to 0 °C with an ice bath and (bicyclo[2.2.1]heptan-2-yl)trichlorosilane (1.1 g, 5.0 mmol, 1.0 equiv.) was added dropwise. The mixture was stirred for an hour at 0 °C and an additional hour at room temperature. The reaction mixture was filtered through a pad of celite and the filtrate was evaporated under reduced pressure. The residue was dissolved in CH_3CN (20 mL) and Et_4NBr (1.1 g, 5.0 mmol, 1.0 equiv.) was added. The mixture was stirred for an hour and the solvent was evaporated under reduced pressure. The residue was dissolved in the minimum volume of acetone, and then diethyl ether was added until a cloudy solution was observed. The flask was placed in a freezer at –20 °C overnight. The precipitate was collected by filtration, washed with diethyl ether and dried under vacuum to afford the corresponding tetraethylammonium silicate (0.28 g, 0.60 mmol, 12% yield) as a brown solid.

^{1}H NMR ($CDCl_3$, 400 MHz): δ 6.80–6.60 (m, 4H), 6.60–6.42 (m, 4H), 2.73 (q, *J* = 7.2 Hz 8H), 2.35–2.21 (m, 1H), 2.07–1.93 (m, 1H), 1.74–1.58 (m, 1H), 1.38–1.27 (m, 2H), 1.24–1.12 (m, 2H), 1.08–0.98 (m, 2H), 0.90 (t, *J* = 8.0 Hz, 12H), 0.83–0.75 (m, 2H).

^{13}C NMR ($CDCl_3$, 400 MHz): δ 150.9, 150.7, 117.7, 117.6, 110.30, 110.25, 51.8, 38.4, 38.3, 36.7, 33.4, 32.8, 31.5, 29.3, 7.0.

HRMS (ESI) calculated for $[C_{19}H_{19}O_4Si]^-$ requires *m*/*z* 339.1058 found *m*/*z* 339.1058.

Table S3. Investigation of PDI MOF Catalysts for Alkene Hydroalkylation with Silicates[a]

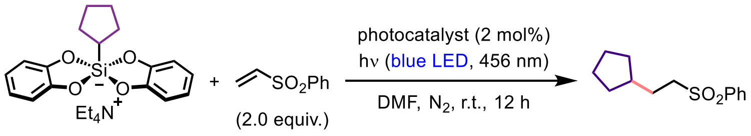

entry	photocatalyst	solvent contents (wt%)	catalyst loading (mol% based on linker)	catalyst amount (mg)	1st run yield (%)	2nd run yield (%)
1	2f-**UiO-PDI**	11.2	2	2.0	70	71
2	activated 2f-**UiO-PDI**	1.3	2	1.8	70	68
3	**UiO-PDI-Me$_2$**	8.4	2	2.0	62	34
4	**UiO-PDI-OMe**	8.1	2	2.0	65	29
5	**UiO-PDI-OH**	8.2	2	2.0	58	20

[a]Reaction conditions: **1a** (0.1 mmol, 1.0 equiv.), **2a** (2.0 equiv.), photocatalyst (2 mol%) in DMF (1.0 mL) under nitrogen atmosphere at room temperature with blue-LED light irradiation (456 nm) for 12 h. Yield was determined by ^{1}H NMR of the crude product using mesitylene as an internal standard.

5. Catalytic Applications of Electron Donor–Acceptor Complex Photoactivation

General Procedure A for the alkene hydroalkylation of various silicates with (vinylsulfonyl)benzene (2a) or *trans*-1,2-dibenzoylethylene (2b). To a 10-mL flame-dried Schlenk tube were added 2f-**UiO-PDI** (2.0 mg, 2 mol%), the corresponding silicate (0.10 mmol, 1.0 equiv.), DMF (1.0 mL), and (vinylsulfonyl)benzene (**2a**, 33.6 mg, 0.20 mmol, 2.0 equiv.) or *trans*-1,2-dibenzoylethylene (**2b**, 47.3 mg, 0.20 mmol, 2.0 equiv.) sequentially. After three freeze-pump-thaw cycles, the reaction mixture was irradiated by three 40-watt Kessil PR160L-456 blue-LED lamps at room temperature (with two fans) for 24 hours. After irradiation, ethyl acetate (5 mL) was added into the crude mixture; the resulting solution was filtered through a pad of silica gel and washed three times with ethyl acetate (3 × 10 mL), brine (3 × 10 mL), dried over Na_2SO_4 and evaporated under reduced pressure. The residue was purified by flash column chromatography on silica gel to afford the corresponding product.

General Procedure B for the alkene hydroalkylation of tetraethylammonium bis(catecholato)-*tert*-butylsilicate (1m) with Michael acceptors. To a 10-mL flame-dried Schlenk tube were added 2f-**UiO-PDI** (2.0 mg, 2 mol%), tetraethylammonium bis(catecholato)-*tert*-butylsilicate (**1m**, 43.2 mg, 0.10 mmol, 1.0 equiv.), DMF (1.0 mL), and electron-deficient alkene (0.20 mmol, 2.0 equiv.) sequentially. After three freeze-pump-thaw cycles, the reaction mixture was irradiated by three 40-watt Kessil PR160L-456 blue-LED lamps at room temperature (with two fans) for 24 hours. After irradiation, ethyl acetate (5 mL) was added into the crude mixture; the resulting solution was filtered through a pad of silica gel and washed three times with ethyl acetate (3 × 10 mL), brine (3 × 10 mL), dried over Na_2SO_4 and evaporated under reduced pressure. The residue was purified by flash column chromatography on silica gel to afford the corresponding product.

General Procedure C for the cascade radical addition/nucleophilic substitution. To a 10-mL flame-dried Schlenk tube were added 2f-**UiO-PDI** (2.0 mg, 2 mol%), tetraethylammonium bis(catecholato)-*tert*-butylsilicate (**1m**, 43.2 mg, 0.10 mmol, 1.0 equiv.),

DMF (1.0 mL), and dineopentyl(vinyl)sulfonium triflate (**4**, 52.6 mg, 0.15 mmol, 1.5 equiv.) sequentially. After three freeze-pump-thaw cycles, the reaction mixture was irradiated by three 40-watt Kessil PR160L-456 blue-LED lamps at room temperature (with two fans) for 24 hours. After irradiation, the crude reaction mixture was concentrated under vacuum for 1 hour. The reaction mixture was filtered and washed three times with CH_2Cl_2 (3 × 2 mL). The combined filtrates were transferred in a 10-mL flame-dried Schlenk tube and concentrated under vacuum. The corresponding nucleophile (0.25 mmol, 2.5 equiv.), K_3PO_4 (31.8 mg, 0.15 mmol, 1.5 equiv. or 53.1 mg, 0.25 mmol, 2.5 equiv.) and DMF (0.20 mL) were added. The mixture was heated to 65 °C without irradiation for 24 h under vigorous stirring (500 rpm). After the indicated reaction time, it was filtered through a pad of celite and eluted with CH_2Cl_2. Volatiles were evaporated under reduced pressure, and the residue was subjected to chromatography purification on silica gel to afford the final compound.

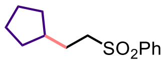

3a

((2-Cyclopentylethyl)sulfonyl)benzene (3a).[10] The title compound was synthesized according to the General Procedure A using tetraethylammonium bis(catecholato)-cyclopentylsilicate (**1a**, 44.4 mg, 0.10 mmol, 1.0 equiv.), (vinylsulfonyl)benzene (**2a**, 33.6 mg, 0.20 mmol, 2.0 equiv.), 2f-**UiO-PDI** (2.0 mg, 2 mol%) and DMF (1.0 mL). The crude mixture was purified by flash column chromatography using hexanes/ethyl acetate (6:1 v/v) as the eluent to give 18.6 mg (0.078 mmol, 78% yield) of the title compound as a colorless oil.

^{1}H NMR ($CDCl_3$, 400 MHz): δ 7.97–7.85 (m, 2H), 7.72–7.62 (m, 1H), 7.62–7.52 (m, 2H), 3.13–3.05 (m, 2H), 1.79–1.67 (m, 5H), 1.63–1.57 (m, 2H), 1.54–1.43 (m, 2H), 1.11–0.98 (m, 2H).

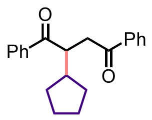

3b

2-Cyclopentyl-1,4-diphenylbutane-1,4-dione (3b).[10] The title compound was synthesized according to the General Procedure A using tetraethylammonium bis(catecholato)-cyclopentylsilicate (**1a**, 44.4 mg, 0.10 mmol, 1.0 equiv.), *trans*-1,2-dibenzoylethylene (**2b**, 47.3 mg, 0.20 mmol, 2.0 equiv.), 2f-**UiO-PDI** (2.0 mg, 2 mol%) and DMF (1.0 mL). The crude mixture was purified by flash column chromatography using hexanes/ethyl acetate (20:1 v/v) as the eluent to give 27.6 mg (0.09 mmol, 90% yield) of the title compound as a white solid.

^{1}H NMR ($CDCl_3$, 400 MHz): δ 8.14–8.07 (m, 2H), 8.01–7.92 (m, 2H), 7.60–7.52 (m, 2H), 7.52–7.40 (m, 4H), 4.11–4.00 (m, 1H), 3.82 (dd, J = 20.0, 12.0 Hz, 1H), 3.24 (dd, J = 20.0, 3.2 Hz, 1H), 2.15–2.02 (m, 1H), 1.87–1.77 (m, 1H), 1.65–1.41 (m, 5H), 1.32–1.21 (m, 1H), 1.20–1.05 (m, 1H).

^{13}C NMR ($CDCl_3$, 100 MHz): δ 204.2, 199.0, 138.1, 136.5, 133.1, 132.7, 128.6, 128.5 (2C), 128.1, 45.5, 43.2, 40.6, 31.1, 30.6, 25.1, 24.5.

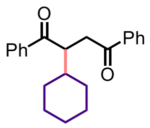

3c

2-Cyclohexyl-1,4-diphenylbutane-1,4-dione (3c).[12] The title compound was synthesized according to the General Procedure A using tetraethylammonium bis(catecholato)-cyclohexylsilicate (**1c**, 45.8 mg, 0.10 mmol, 1.0 equiv.), *trans*-1,2-dibenzoylethylene (**2b**, 47.3 mg, 0.20 mmol, 2.0 equiv.), 2f-**UiO-PDI** (2.0 mg, 2 mol%) and DMF (1.0 mL). The crude mixture was purified by flash column chromatography using hexanes/ethyl acetate (20:1 v/v) as the eluent to give 27.6 mg (0.083 mmol, 83% yield) of the title compound as a yellow oil.

^{1}H NMR ($CDCl_3$, 400 MHz): δ 8.12–8.02 (m, 2H), 8.02–7.92 (m, 2H), 7.62–7.37 (m, 6H), 4.11–4.00 (m, 1H), 3.79 (dd, J = 20.0, 12 Hz, 1H), 3.18 (dd, J = 20.0, 3.2 Hz, 1H), 1.82–1.62 (m, 6H), 1.30–1.00 (m, 5H).

^{13}C NMR ($CDCl_3$, 100 MHz): δ 203.3, 199.1, 137.8, 136.7, 133.1, 132.7, 128.6, 128.50, 128.47, 128.1, 46.5, 40.3, 38.1, 31.9, 29.9, 26.6, 26.4, 26.2.

HRMS (ESI) calculated for $[C_{22}H_{25}O_2]^+$ requires *m/z* 321.1849, found *m/z* 321.1845.

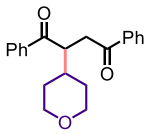

3d

1,4-Diphenyl-2-(tetrahydro-2*H*-pyran-4-yl)butane-1,4-dione (3d). The title compound was synthesized according to the General Procedure A using potassium (18-Crown-6) bis(catecholato)-(tetrahydro-2*H*-pyran-4-yl)silicate (**1d**, 63.3 mg, 0.10 mmol, 1.0 equiv.), *trans*-1,2-dibenzoylethylene (**2b**, 47.3 mg, 0.20 mmol, 2.0 equiv.), 2f-**UiO-PDI** (2.0 mg, 2 mol%) and DMF (1.0 mL). The crude mixture was purified by flash column chromatography using hexanes/ethyl acetate (10:1 v/v) as the eluent to give 14.2 mg (0.044 mmol, 44% yield) of the title compound as a yellow oil.

^{1}H NMR ($CDCl_3$, 400 MHz): δ 8.12–8.04 (m, 2H), 8.00–7.93 (m, 2H), 7.63–7.54 (m, 2H), 7.53–7.43 (m, 4H), 4.14–4.04 (m, 1H), 4.03–3.84 (m, 2H), 3.77 (dd, *J* = 20.0, 12.0 Hz, 1H), 3.36–3.26 (m, 2H), 3.22 (dd, *J* = 20.0, 3.2 Hz, 1H), 2.03–1.91 (m, 1H), 1.56–1.39 (m, 4H).

^{13}C NMR ($CDCl_3$, 100 MHz): δ 202.9, 198.6, 137.6, 136.5, 133.3, 133.0, 128.7, 128.6, 128.5, 128.1, 68.0, 67.9, 45.8, 38.2, 37.7, 31.5, 30.0.

HRMS (ESI) calculated for $[C_{21}H_{23}O_3]^+$ requires *m/z* 323.1642, found *m/z* 323.1637.

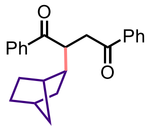

3e

Bicyclo[2.2.1]heptan-2-yl)-1,4-diphenylbutane-1,4-dione (3e). The title compound was synthesized according to the General Procedure A using tetraethylammonium bis(catecholato)-(bicyclo[2.2.1]heptan-2-yl)silicate (**1e**, 47.0 mg, 0.1 mmol, 1.0 equiv.), *trans*-1,2-dibenzoylethylene (**2b**, 47.3 mg, 0.2 mmol, 2.0 equiv.), 2f-**UiO-PDI** (2.0 mg, 2 mol%) and DMF (1.0 mL). The crude mixture was purified by flash column chromatography using hexanes/ethyl acetate (20:1 v/v) as the eluent to give 31.2 mg (0.094 mmol, 94% yield, mixture of diastereomers, 1.3:1 d.r.) of the title compound as a white solid.

^{1}H NMR ($CDCl_3$, 400 MHz) (mixture of diastereomers): δ 8.13–8.07 (m, 2H), 8.00–7.88 (m, 2H), 7.61–7.47 (m, 4H), 7.47–7.39 (m, 2H), 4.01 (dt, *J* = 8.0, 2.8 Hz, 0.43H), 3.84 (t, *J* = 1.2 Hz, 0.57H), 3.77–3.63 (m, 1H), 3.32 (dd, *J* = 20.0, 2.8 Hz, 0.43H), 3.16 (d, *J* = 20.0 Hz, 0.57H), 2.29–2.23 (m, 0.57H), 2.20–2.08 (m, 0.88H), 1.87–1.71 (m, 1.60H), 1.58–1.00 (m, 9.25H).

^{13}C NMR ($CDCl_3$, 100 MHz) (mixture of diastereomers): δ 204.8, 204.7, 199.1, 198.8, 138.7, 136.52, 136.48, 133.22, 133.19, 132.8, 132.7, 128.7, 128.63, 128.60, 128.583, 128.581, 128.6, 128.5, 128.14, 128.11, 45.9, 45.8, 45.6, 45.1, 42.2, 41.0, 39.5, 39.3, 37.2, 36.7, 36.5, 36.2, 36.0, 35.8, 30.6, 30.2, 28.6, 28.5.

HRMS (ESI) calculated for $[C_{23}H_{25}O_2]^+$ requires *m/z* 333.1849, found *m/z* 333.1850.

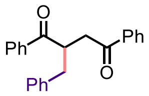

3f

2-Benzyl-1,4-diphenylbutane-1,4-dione (3f).[12] The title compound was synthesized according to the General Procedure A using potassium (18-Crown-6) bis(catecholato)-benzylsilicate (**1f**, 63.9 mg, 0.10 mmol, 1.0 equiv.), *trans*-1,2-dibenzoylethylene (**2b**, 47.3 mg, 0.20 mmol, 2.0 equiv.), 2f-**UiO-PDI** (2.0 mg, 2 mol%) and DMF (1.0 mL). The crude mixture was purified by flash column chromatography using hexanes/ethyl acetate (20:1 v/v) as the eluent to give 31.9 mg (0.097 mmol, 97% yield) of the title compound as a yellow oil.

^{1}H NMR ($CDCl_3$, 400 MHz): δ 8.07–8.02 (m, 2H), 7.94–7.87 (m, 2H), 7.59–7.52 (m, 2H), 7.50–7.39 (m, 4H), 7.27–7.15 (m, 5H), 4.48–4.34 (m, 1H), 3.71 (dd, *J* = 20.0, 8.0 Hz, 1H), 3.19–3.02 (m, 2H), 2.73 (dd, *J* = 12.0, 8.0 Hz, 1H).

^{13}C NMR ($CDCl_3$, 100 MHz): δ 202.7, 198.4, 138.6, 136.6, 136.5, 133.2, 133.0, 129.0, 128.9,128.63, 128.59, 128.51, 128.1, 126.6, 43.4, 40.2, 38.2.

HRMS (ESI) calculated for $[C_{23}H_{21}O_2]^+$ requires *m/z* 329.1536, found *m/z* 329.1538.

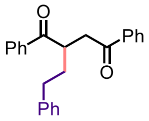

3g

2-Phenethyl-1,4-diphenylbutane-1,4-dione (3g).[13] The title compound was synthesized according to the General Procedure A using potassium (18-Crown-6) bis(catecholato)-benzylsilicate (**1g**, 65.3 mg, 0.10 mmol, 1.0 equiv.), *trans*-1,2-dibenzoylethylene (**2b**, 47.3 mg, 0.20 mmol, 2.0 equiv.), 2f-**UiO-PDI** (2.0 mg, 2 mol%) and DMF (1.0 mL). The crude mixture was purified by flash column chromatography using hexanes/ethyl acetate (20:1 v/v) as the eluent to give 13.7 mg (0.04 mmol, 40% yield) of the title compound as a yellow oil.

^{1}H NMR ($CDCl_3$, 400 MHz): δ 8.01–7.92 (m, 4H), 7.60–7.52 (m, 2H), 7.50–7.40 (m, 4H), 7.29–7.23 (m, 2H), 7.22–7.15 (m, 1H), 7.15–7.08 (m, 2H), 4.22–4.12 (m, 1H), 3.76 (dd, J = 20.0, 8.0 Hz, 1H), 3.22 (dd, J = 20.0, 4.0 Hz, 1H), 2.74–2.58 (m, 2H), 2.17–2.03 (m, 1H), 1.95–1.83 (m, 1H).

^{13}C NMR ($CDCl_3$, 100 MHz): δ 203.1, 198.4, 141.1, 136.7, 136.6, 133.2, 133.0, 128.63, 128.57, 128.51, 128.46, 128.4, 128.1, 126.1, 40.7, 40.6, 33.9, 33.5.

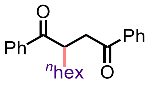

3h

2-Hexyl-1,4-diphenylbutane-1,4-dione (3h). The title compound was synthesized according to the General Procedure A using potassium (18-Crown-6) bis(catecholato)-hexylsilicate (**1h**, 63.3 mg, 0.10 mmol, 1.0 equiv.), *trans*-1,2-dibenzoylethylene (**2b**, 47.3 mg, 0.20 mmol, 2.0 equiv.), 2f-**UiO-PDI** (2.0 mg, 2 mol%) and DMF (1.0 mL). The crude mixture was purified by flash column chromatography using hexanes/ethyl acetate (20:1 v/v) as the eluent to give 20.0 mg (0.062 mmol, 62% yield) of the title compound as a yellow oil.

^{1}H NMR ($CDCl_3$, 400 MHz): δ 8.12–8.01 (m, 2H), 8.01–7.91 (m, 2H), 7.61–7.52 (m, 2H), 7.52–7.40 (m, 4H), 4.18–4.09 (m, 1H), 3.73 (dd, J = 16.0, 8.0 Hz, 1H), 3.17 (dd, J = 20.0, 4.0 Hz, 1H), 1.80–1.71 (m, 1H), 1.58–1.49 (m, 1H), 1.37–1.17 (m, 8H), 0.85 (t, J = 6.0 Hz, 3H).

^{13}C NMR ($CDCl_3$, 100 MHz): δ 203.5, 198.8, 136.9, 136.7, 133.1, 132.9, 128.6, 128.54, 128.47, 128.1, 41.3, 40.7, 32.5, 31.6, 29.3, 27.3, 22.5, 14.0.

HRMS (ESI) calculated for $[C_{22}H_{27}O_2]^+$ requires *m*/*z* 323.2006, found *m*/*z* 323.2005.

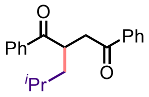

3i

2-Isobutyl-1,4-diphenylbutane-1,4-dione (3i). The title compound was synthesized according to the General Procedure A using potassium (18-Crown-6) bis(catecholato)-isobutylsilicate (**1i**, 60.5 mg, 0.10 mmol, 1.0 equiv.), *trans*-1,2-dibenzoylethylene (**2b**, 47.3 mg, 0.20 mmol, 2.0 equiv.), 2f-**UiO-PDI** (2.0 mg, 2 mol%) and DMF (1.0 mL). The crude mixture was purified by flash column chromatography using hexanes/ethyl acetate (20:1 v/v) as the eluent to give 11.8 mg (0.04 mmol, 40% yield) of the title compound as a yellow oil.

^{1}H NMR ($CDCl_3$, 400 MHz): δ 8.12–8.03 (m, 2H), 8.02–7.92 (m, 2H), 7.63–7.52 (m, 2H), 7.52-7.41 (m, 4H), 4.26–4.15 (m, 1H), 3.70 (dd, *J* = 20.0, 8.0 Hz, 1H), 3.16 (dd, *J* = 20.0, 4.0 Hz, 1H), 1.71–1.59 (m, 2H), 1.47–1.34 (m, 1H), 0.96 (d, *J* = 6.0 Hz, 3H), 0.92 (d, *J* = 6.0 Hz, 3H).

^{13}C NMR ($CDCl_3$, 100 MHz): δ 203.8, 198.7, 136.8, 136.6, 133.2, 132.9, 128.64, 128.55, 128.49, 128.1, 41.6, 40.9, 39.4, 26.1, 23.1, 22.1.

HRMS (ESI) calculated for $[C_{20}H_{23}O_2]^+$ requires *m*/*z* 295.1693, found *m*/*z* 295.1690.

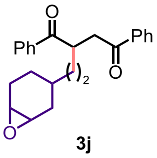

3j

2-(2-(7-Oxabicyclo[4.1.0]heptan-3-yl)ethyl)-1,4-diphenylbutane-1,4-dione (3j). The title compound was synthesized according to the General Procedure A using potassium (18-Crown-6) bis(catecholato)-[2-(7-oxabicyclo[4.1.0]hept-3-yl)ethyl]silicate (**1j**, 67.3 mg, 0.10 mmol, 1.0 equiv.), *trans*-1,2-dibenzoylethylene (**2b**, 47.3 mg, 0.20 mmol, 2.0 equiv.), 2f-**UiO-PDI** (2.0 mg, 2 mol%) and DMF (1.0 mL). The crude mixture was purified by flash column chromatography using hexanes/ethyl acetate (5:1 v/v) as the eluent to give 21.0 mg (0.058 mmol, 58% yield) of the title compound as a colorless oil.

^{1}**H NMR** ($CDCl_3$, 400 MHz): δ 8.10–8.00 (m, 2H), 8.00–7.91 (m, 2H), 7.64–7.53 (m, 2H), 7.53–7.41 (m, 4H), 4.17–4.04 (m, 1H), 3.74–3.64 (m, 1H), 3.21–3.02 (m, 3H), 2.19–2.04 (m, 1H), 2.04–1.87 (m, 1H), 1.85–1.62 (m, 2H), 1.48–1.04 (m, 6H), 1.02–0.69 (m, 1H).

^{13}**C NMR** ($CDCl_3$, 100 MHz): δ 203.2, 198.5, 136.8, 136.6, 133.2, 133.0, 128.7, 128.6, 128.4, 128.1, 52.98, 52.97, 52.5, 51.8, 51.7, 51.6, 41.32, 41.27, 40.67, 40.65, 33.7, 33.6, 32.7, 31.7, 31.6, 30.4, 29.74, 29.71, 29.69, 29.66, 29.57, 27.0, 26.9, 25.1, 24.3, 23.4.

HRMS (ESI) calculated for $[C_{24}H_{27}O_3]^+$ requires *m*/*z* 363.1955, found *m*/*z* 363.1950.

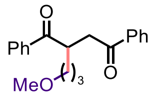

3k

2-(3-Methoxypropyl)-1,4-diphenylbutane-1,4-dione (3k). The title compound was synthesized according to the General Procedure A using potassium (18-Crown-6) bis(catecholato)(3-methoxypropyl)silicate (**1k**, 62.1 mg, 0.10 mmol, 1.0 equiv.), *trans*-1,2-dibenzoylethylene (**2b**, 47.3 mg, 0.20 mmol, 2.0 equiv.), 2f-**UiO-PDI** (2.0 mg, 2 mol%) and DMF (1.0 mL). The crude mixture was purified by flash column chromatography using hexanes/ethyl acetate (5:1 v/v) as the eluent to give 11.8 mg (0.038 mmol, 38% yield) of the title compound as a yellow oil.

^{1}**H NMR** ($CDCl_3$, 400 MHz): δ 8.12–8.02 (m, 2H), 8.02–7.91 (m, 2H),7.61-7.52 (m, 2H), 7.52-7.39 (m, 4H), 4.27–4.08 (m, 1H), 3.73 (dd, *J* = 20.0, 8.0 Hz, 1H), 3.35 (t, *J* = 6.0 Hz, 2H), 3.28 (s, 3H), 3.18 (dd, *J* = 20.0, 4.4 Hz, 1H), 1.92–1.78 (m, 1H), 1.75–1.59 (m, 3H).

^{13}**C NMR** ($CDCl_3$, 100 MHz): δ 203.2, 198.6, 136.8, 136.6, 133.2, 133.0, 128.7, 128.6, 128.5, 128.1, 72.3, 58.6, 40.9, 40.8, 29.1, 27.3.

HRMS (ESI) calculated for $[C_{20}H_{23}O_3]^+$ requires *m*/*z* 311.1642, found *m*/*z* 311.1640.

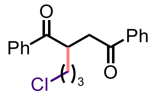

3l

2-(3-Chloropropyl)-1,4-diphenylbutane-1,4-dione (3l). The title compound was synthesized according to the General Procedure A using potassium (18-Crown-6)

bis(catecholato)-3-chloropropylsilicate (**1l**, 62.5 mg, 0.10 mmol, 1.0 equiv.), *trans*-1,2-dibenzoylethylene (**2b**, 47.3 mg, 0.20 mmol, 2.0 equiv.), 2f-**UiO-PDI** (2.0 mg, 2 mol%) and DMF (1.0 mL). The crude mixture was purified by flash column chromatography using hexanes/ethyl acetate (10:1 v/v) as the eluent to give 12.6 mg (0.040 mmol, 40% yield) of the title compound as a yellow oil.

^{1}H NMR ($CDCl_3$, 400 MHz): δ 8.15–8.01 (m, 2H), 8.01–7.90 (m, 2H), 7.65–7.54 (m, 2H), 7.54–7.38 (m, 4H), 4.29–4.10 (m, 1H), 3.73 (dd, J = 20.0, 8.8 Hz, 1H), 3.50 (t, J = 6.0 Hz, 2H), 3.17 (dd, J = 20.0, 4.4 Hz, 1H), 2.02–1.87 (m, 1H), 2.02–1.64 (m, 3H).

^{13}C NMR ($CDCl_3$, 100 MHz): δ 202.8, 198.2, 136.6, 136.5, 133.3, 133.2, 128.8, 128.6, 128.5, 128.1, 44.6, 40.8, 40.4, 30.1, 29.7.

HRMS (ESI) calculated for $[C_{19}H_{20}ClO_2]^+$ requires m/z 315.1146, found m/z 315. 1145.

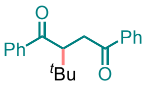

3m

2-(*Tert*-butyl)-1,4-diphenylbutane-1,4-dione (3m).[10] The title compound was synthesized according to the General Procedure B using tetraethylammonium bis(catecholato)-*tert*-butylsilicate (**1m**, 43.2 mg, 0.10 mmol, 1.0 equiv.), *trans*-1,2-dibenzoylethylene (**2b**, 47.3 mg, 0.20 mmol, 2.0 equiv.), 2f-**UiO-PDI** (2.0 mg, 2 mol%) and DMF (1.0 mL). The crude mixture was purified by flash column chromatography using hexanes/ethyl acetate (20:1 v/v) as the eluent to give 26.8 mg (0.091 mmol, 91% yield) of the title compound as a yellow oil.

^{1}H NMR ($CDCl_3$, 400 MHz): δ 8.23–8.03 (m, 2H), 8.03–7.84 (m, 2H), 7.61–7.51 (m, 2H), 7.51–7.33 (m, 4H), 4.06 (dd, J = 11.2, 2.4 Hz, 1H), 3.89 (dd, J = 20.0, 12.0 Hz, 1H), 3.24 (dd, J = 20.0, 2.0 Hz, 1H), 0.98 (s, 9H).

^{13}C NMR ($CDCl_3$, 100 MHz): δ 204.3, 199.3, 139.8, 136.6, 133.1, 132.4, 128.501, 128.497, 128.4, 128.1, 49.3, 38.8, 33.4, 28.6.

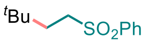

3n

((3,3-Dimethylbutyl)sulfonyl)benzene (3n).[10] The title compound was synthesized according to the General Procedure B using tetraethylammonium bis(catecholato)-*tert*-

butylsilicate (**1m**, 43.2 mg, 0.10 mmol, 1.0 equiv.), (vinylsulfonyl)benzene (**2a**, 33.6 mg, 0.20 mmol, 2.0 equiv.), 2f-**UiO-PDI** (2.0 mg, 2 mol%) and DMF (1.0 mL). The crude mixture was purified by flash column chromatography using hexanes/ethyl acetate (5:1 v/v) as the eluent to give 20.4 mg (0.090 mmol, 90% yield) of the title compound as a colorless oil.

^{1}H NMR ($CDCl_3$, 400 MHz): δ 7.98–7.83 (m, 2H), 7.72–7.62 (m, 1H), 7.60–7.50 (m, 2H), 3.13–2.95 (m, 2H), 1.63–1.58 (m, 2H), 0.86 (s, 9H).

^{13}C NMR ($CDCl_3$, 100 MHz): δ 139.2, 133.6, 129.3, 128.0, 52.9, 35.6, 30.0, 28.9.

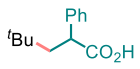

3o

4,4-Dimethyl-2-phenylpentanoic acid (3o).[11] The title compound was synthesized according to the General Procedure B using tetraethylammonium bis(catecholato)-*tert*-butylsilicate (**1m**, 43.2 mg, 0.10 mmol, 1.0 equiv.), 2-phenylacrylic acid (29.6 mg, 0.20 mmol, 2.0 equiv.), 2f-**UiO-PDI** (2.0 mg, 2 mol%) and DMF (1.0 mL). The crude mixture was purified by flash column chromatography using hexanes/ethyl acetate (10:1 v/v) as the eluent to give 18.4 mg (0.089 mmol, 89% yield) of the title compound as a white solid.

^{1}H NMR ($CDCl_3$, 400 MHz): δ 7.35–7.27 (m, 4H), 7.26–7.20 (m, 1H), 3.65 (dd, J = 8.4, 4.4 Hz, 1H), 2.27 (dd, J = 14.0, 8.8 Hz, 1H), 1.62 (dd, J = 14.0, 4.4 Hz, 1H), 0.90 (s, 9H).

^{13}C NMR ($CDCl_3$, 100 MHz): δ 180.4, 140.3, 128.7, 127.9, 127.2, 48.0, 46.9, 31.0, 29.4.

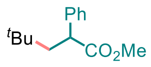

3p

Methyl 4,4-dimethyl-2-phenylpentanoate (3p).[11] The title compound was synthesized according to the General Procedure B using tetraethylammonium bis(catecholato)-*tert*-butylsilicate (**1m**, 43.2 mg, 0.10 mmol, 1.0 equiv.), methyl 2-phenylacrylate (32.4 mg, 0.20 mmol, 2.0 equiv.), 2f-**UiO-PDI** (2.0 mg, 2 mol%) and DMF (1.0 mL). The crude mixture was purified by flash column chromatography using hexanes/ethyl acetate (50:1 v/v) as the eluent to give 19.8 mg (0.090 mmol, 90% yield) of the title compound as a yellow oil.

^{1}**H NMR** ($CDCl_3$, 400 MHz): δ 7.34–7.27 (m, 4H), 7.27–7.20 (m, 1H), 3.69–3.64 (m, 1H, overlapped), 3.64 (s, 3H, overlapped), 2.30 (dd, J = 12.0, 8.0 Hz, 1H), 1.58 (dd, J = 12.0, 4.0 Hz, 1H), 0.89 (s, 9H).

^{13}**C NMR** ($CDCl_3$, 100 MHz): δ 175.3, 140.9, 128.6, 127.8, 127.0, 52.0, 48.1, 47.4, 31.0, 29.4.

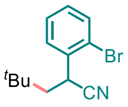

3q

2-(2-Bromophenyl)-4,4-dimethylpentanenitrile (3q). The title compound was synthesized according to the General Procedure B using tetraethylammonium bis(catecholato)-*tert*-butylsilicate (**1m**, 43.2 mg, 0.10 mmol, 1.0 equiv.), 2-(2-bromophenyl)acrylonitrile (41.6 mg, 0.20 mmol, 2.0 equiv.), 2f-**UiO-PDI** (2.0 mg, 2 mol%) and DMF (1.0 mL). The crude mixture was purified by flash column chromatography using hexanes/ethyl acetate (20:1 v/v) as the eluent to give 25.3 mg (0.095 mmol, 95% yield) of the title compound as a colorless oil.

^{1}**H NMR** ($CDCl_3$, 400 MHz): δ 7.64–7.52 (m, 2H), 7.42–7.31 (m, 1H), 7.23–7.14 (m, 1H), 4.29 (dd, J = 11.2, 3.2 Hz, 1H), 1.88 (dd, J = 16.0, 12.0 Hz, 1H), 1.64 (dd, J = 16.0, 3.2 Hz, 1H), 1.10 (s, 9H).

^{13}**C NMR** ($CDCl_3$, 100 MHz): δ 137.1, 133.3, 129.5, 129.0, 128.3, 122.5, 121.7, 48.7, 33.1, 31.4, 29.5.

HRMS (ESI) calculated for $[C_{13}H_{17}BrN]^+$ requires m/z 266.0539, found m/z 266.0544.

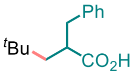

3r

2-Benzyl-4,4-dimethylpentanoic acid (3r).[11] The title compound was synthesized according to the General Procedure B using tetraethylammonium bis(catecholato)-*tert*-butylsilicate (**1m**, 43.2 mg, 0.10 mmo, 1.0 equiv.l), 2-benzylacrylic acid (32.4 mg, 0.20 mmol, 2.0 equiv.), 2f-**UiO-PDI** (2.0 mg, 2 mol%) and DMF (1.0 mL). The crude mixture was purified by flash column chromatography using hexanes/ethyl acetate (5:1 v/v) as the eluent to give 19.8 mg (0.090 mmol, 90% yield) of the title compound as a colorless oil.

^{1}H NMR ($CDCl_3$, 400 MHz): δ 7.30–7.24 (m, 2H), 7.24–7.20 (m, 1H), 7.20–7.14 (m, 2H), 3.01–2.90 (m, 1H), 2.78–2.61 (m, 2H), 1.88–1.73 (m, 1H), 1.32 (dd, J = 16.0, 2.0 Hz, 1H), 0.86 (s, 9H).

^{13}C NMR ($CDCl_3$, 100 MHz): δ 182.7, 138.8, 128.9, 128.4, 126.5, 45.2, 43.9, 40.6, 30.7, 29.3.

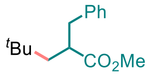

3s

Methyl 2-benzyl-4,4-dimethylpentanoate (3s).[11] The title compound was synthesized according to the General Procedure B using tetraethylammonium bis(catecholato)-*tert*-butylsilicate (**1m**, 43.2 mg, 0.10 mmol, 1.0 equiv.), methyl 2-benzylacrylate (35.2 mg, 0.20 mmol, 2.0 equiv.), 2f-**UiO-PDI** (2.0 mg, 2 mol%) and DMF (1.0 mL). The crude mixture was purified by flash column chromatography using hexanes/ethyl acetate (50:1 v/v) as the eluent to give 21.6 mg (0.092 mmol, 92% yield) of the title compound as a colorless oil.

^{1}H NMR ($CDCl_3$, 400 MHz): δ 7.30–7.26 (m, 2H), 7.23–7.18 (m, 1H), 7.18–7.09 (m, 2H), 3.54 (s, 3H), 2.95–2.83 (m, 1H), 2.77–2.62 (m, 2H), 1.90–1.77 (m, 1H), 1.37–1.24 (m, 1H), 0.84 (s, 9H).

^{13}C NMR ($CDCl_3$, 100 MHz): δ 177.2, 139.1, 128.9, 128.3, 126.4, 51.4, 45.6, 44.2, 40.9, 30.6, 29.3.

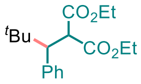

3t

Diethyl 2-(2,2-dimethyl-1-phenylpropyl)malonate (3t).[11] The title compound was synthesized according to the General Procedure B using tetraethylammonium bis(catecholato)-*tert*-butylsilicate (**1m**, 43.2 mg, 0.1 mmol, 1.0 equiv.), diethyl 2-(1-phenylvinyl)malonate (49.7 mg, 0.2 mmol, 2.0 equiv.), 2f-**UiO-PDI** (2.0 mg, 2 mol%) and DMF (1.0 mL). The crude mixture was purified by flash column chromatography using hexanes/ethyl acetate (20:1 v/v) as the eluent to give 27.0 mg (0.088 mmol, 88% yield) of the title compound as a colorless oil.

^{1}H NMR ($CDCl_3$, 400 MHz): δ 7.25–7.19 (m, 2H), 7.19–7.10 (m, 3H), 4.29–4.14 (m, 2H), 3.97 (d, *J* = 12.0 Hz, 1H), 3.80–3.63 (m, 2H), 3.47 (d, *J* = 12.0 Hz, 1H), 1.30 (t, *J* = 7.2 Hz, 3H), 0.88 (s, 9H), 0.79 (t, *J* = 7.2 Hz, 3H).

^{13}C NMR ($CDCl_3$, 100 MHz): δ 169.6, 168.2, 140.1, 127.4, 126.5, 61.7, 61.2, 55.1, 54.9, 34.4, 28.2, 13.9, 13.4.

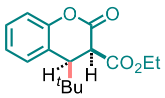

3u

***cis*-Ethyl 4-(*tert*-butyl)-2-oxochromane-3-carboxylate (3u).**[11] The title compound was synthesized according to the General Procedure B using tetraethylammonium bis(catecholato)-*tert*-butylsilicate (**1m**, 43.2 mg, 0.10 mmol, 1.0 equiv.), ethyl 2-oxo-2*H*-chromene-3-carboxylate (43.6 mg, 0.20 mmol, 2.0 equiv.), 2f-**UiO-PDI** (2.0 mg, 2 mol%) and DMF (1.0 mL). The crude mixture was purified by flash column chromatography using hexanes/ethyl acetate (10:1 v/v) as the eluent to give 20.2 mg (0.073 mmol, 73% yield) of the title compound as a colorless oil.

^{1}H NMR ($CDCl_3$, 400 MHz): δ 7.33–7.26 (m, 1H), 7.19–7.13 (dd, *J* = 8.0, 2.0 Hz, 1H), 7.13–7.04 (m, 2H), 4.12–3.93 (m, 3H), 3.14 (d, *J* = 1.2 Hz, 1H), 0.99 (t, *J* = 7.2 Hz, 3H, overlapped), 0.98 (s, 9H, overlapped).

^{13}C NMR ($CDCl_3$, 100 MHz): δ 167.9, 165.8, 151.6, 131.1, 128.9, 124.0, 121.3, 117.0, 62.2, 50.6, 48.6, 34.5, 27.3, 13.7.

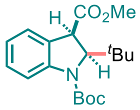

3v

***cis*-1-(*tert*-Butyl) 3-methyl 2-(*tert*-butyl)indoline-1,3-dicarboxylate (3v).** The title compound was synthesized according to the General Procedure B using tetraethylammonium bis(catecholato)-*tert*-butylsilicate (**1m**, 43.2 mg, 0.10 mmol, 1.0 equiv.), 1-(*tert*-butyl) 3-methyl 1*H*-indole-1,3-dicarboxylate (55.1 mg, 0.20 mmol, 2.0 equiv.), 2f-**UiO-PDI** (2.0 mg, 2 mol%) and DMF (1.0 mL). The crude mixture was purified by flash column chromatography using

hexanes/ethyl acetate (5:1 v/v) as the eluent to give 23.3 mg (0.070 mmol, 70% yield) of the title compound as a colorless oil. The relative stereochemistry of **3v** was established by NOE experiments.

^{1}H NMR ($CDCl_3$, 400 MHz): δ 7.73–7.45 (m, 1H), 7.32–7.20 (m, 2H), 7.05–6.91 (m, 1H), 4.65 (s, 1H), 3.80 (s, 1H), 3.68 (s, 3H), 1.56 (s, 9H), 0.85 (s, 9H).

^{13}C NMR ($CDCl_3$, 100 MHz): δ 172.2, 153.7, 143.9, 129.7, 128.6, 124.7, 123.1, 117.3, 81.1, 70.0, 52.5, 48.0, 36.3, 28.3, 26.0.

HRMS (ESI) calculated for $[C_{19}H_{27}NO_4]^+$ requires *m*/*z* 334.2013, found *m*/*z* 334.2013.

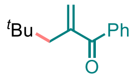

3w

4,4-Dimethyl-2-methylene-1-phenylpentan-1-one (3w). The title compound was synthesized according to the General Procedure B using tetraethylammonium bis(catecholato)-*tert*-butylsilicate (**1m**, 43.2 mg, 0.10 mmol, 1.0 equiv.), 2-benzoylallyl acetate (40.8 mg, 0.20 mmol, 2.0 equiv.), 2f-**UiO-PDI** (2.0 mg, 2 mol%) and DMF (1.0 mL). The crude mixture was purified by flash column chromatography using hexanes/ethyl acetate (50:1 v/v) as the eluent to give 13.4 mg (0.066 mmol, 66% yield) of the title compound as a colorless oil.

^{1}H NMR ($CDCl_3$, 400 MHz): δ 7.86–7.79 (m, 2H), 7.59–7.49 (m, 1H), 7.49–7.39 (m, 2H), 5.78 (dd, J = 2.4, 1.2 Hz, 1H), 5.65 (d, J = 1.2 Hz, 1H), 2.50 (d, J = 0.8 Hz, 2H), 0.93 (s, 9H).

^{13}C NMR ($CDCl_3$, 100 MHz): δ 198.1, 146.6, 137.3, 132.2, 129.9, 128.2, 128.0, 45.6, 31.7, 29.5.

HRMS (ESI) calculated for $[C_{14}H_{19}O]^+$ requires *m*/*z* 203.1430, found *m*/*z* 203.1425.

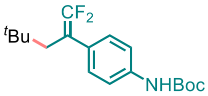

3x

***tert*-Butyl (4-(1,1-difluoro-4,4-dimethylpent-1-en-2-yl)phenyl)carbamate (3x)**. The title compound was synthesized according to the General Procedure B using tetraethylammonium bis(catecholato)-*tert*-butylsilicate (**1m**, 43.2 mg, 0.10 mmol, 1.0 equiv.), *tert*-butyl (4-(3,3,3-

trifluoroprop-1-en-2-yl)phenyl)carbamate (57.5 mg, 0.20 mmol, 2.0 equiv.), 2f-**UiO-PDI** (2.0 mg, 2 mol%) and DMF (1.0 mL). The crude mixture was purified by flash column chromatography using hexanes/ethyl acetate (10:1 v/v) as the eluent to give 24.7 mg (0.076 mmol, 76% yield) of the title compound as a colorless oil.

^{1}H NMR ($CDCl_3$, 400 MHz): δ 7.33 (d, *J* = 8.4 Hz, 2H), 7.23 (d, *J* = 8.4 Hz, 2H), 6.48 (s, 1H), 2.30 (s, 2H), 1.52 (s, 9H), 0.79 (s, 9H).

^{13}C NMR ($CDCl_3$, 100 MHz): δ 154.4 (dd, *J* = 287.7, 285.3 Hz), 152.7, 137.1, 130.1 (dd, *J* = 4.7, 2.8 Hz), 129.0 (t, *J* = 2.8 Hz), 118.2, 90.5 (dd, *J* = 21.7, 13 Hz), 80.6, 41.1, 32.7 (t, *J* = 2.5 Hz), 29.7, 28.3.

^{19}F NMR ($CDCl_3$, 377 MHz): δ –90.2 (d, *J* = 41.1 Hz), –92.7 (d, *J* = 42.2 Hz).

HRMS (ESI) calculated for $[C_{18}H_{25}F_2NO_2Na]^+$ requires *m/z* 326.1926, found *m/z* 348.1743.

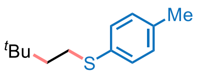

5a

(3,3-Dimethylbutyl)(*p*-tolyl)sulfane (5a). The title compound was synthesized according to the General Procedure C using tetraethylammonium bis(catecholato)-*tert*-butylsilicate (**1m**, 43.2 mg, 0.10 mmol, 1.0 equiv.), dineopentyl(vinyl)sulfonium triflate (**4**, 52.6 mg, 0.15 mmol, 1.5 equiv.), 2f-**UiO-PDI** (2.0 mg, 2 mol%) and DMF (1.0 mL). After photo reaction, 4-methylbenzenethiol (31.1 mg, 0.25 mmol, 2.5 equiv.), K_3PO_4 (31.8 mg, 0.15 mmol, 1.5 equiv.) in DMF (0.20 mL) were added. The reaction was performed under nitrogen atmosphere at 65 °C for 24 h. The crude mixture was purified by flash column chromatography using hexanes as the eluent to give 19.4 mg (0.093 mmol, 93% yield) of the title compound as a colorless oil.

^{1}H NMR ($CDCl_3$, 400 MHz): δ 7.23 (d, *J* = 8.0 Hz, 2H), 7.10 (d, *J* = 8.0 Hz, 2H), 2.96–2.73 (m, 2H), 2.32 (s, 3H), 1.55–1.50 (m, 2H), 0.91 (s, 9H).

^{13}C NMR ($CDCl_3$, 100 MHz): δ 135.7, 133.1, 129.6, 129.4, 43.4, 30.8, 29.8, 29.1, 21.0.

HRMS (ESI) calculated for $[C_{13}H_{21}S]^+$ requires *m/z* 209.1358, found *m/z* 209.1356.

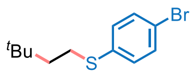

5b

(4-Bromophenyl)(3,3-dimethylbutyl)sulfane (5b). The title compound was synthesized according to the General Procedure C using tetraethylammonium bis(catecholato)-*tert*-butylsilicate (**1m**, 43.2 mg, 0.10 mmol, 1.0 equiv.), dineopentyl(vinyl)sulfonium triflate (**4**, 52.6 mg, 0.15 mmol, 0.15 equiv.), 2f-**UiO-PDI** (2.0 mg, 2 mol%) and DMF (1.0 mL). After photo reaction, 4-bromobenzenethiol (47.3 mg, 0.25 mmol, 2.5 equiv.), K_3PO_4 (31.8 mg, 0.15 mmol, 1.5 equiv.) in DMF (0.20 mL) were added. The reaction was performed under nitrogen atmosphere at 65 °C for 24 h. The crude mixture was purified by flash column chromatography using hexanes as the eluent to give 25.1 mg (0.092 mmol, 92% yield) of the title compound as a colorless oil.

^{1}H NMR ($CDCl_3$, 400 MHz): δ 7.39 (d, *J* = 8.4 Hz, 2H), 7.17 (d, *J* = 8.4 Hz, 2H), 2.90–2.82 (m, 2H), 1.55–1.50 (m, 2H), 0.93 (s, 9H).

^{13}C NMR ($CDCl_3$, 100 MHz): δ 136.3, 131.8, 130.1, 119.3, 43.1, 30.8, 29.2, 29.1.

HRMS (EI) calculated for $[C_{12}H_{17}BrS]^+$ requires *m*/*z* 272.0229, found *m*/*z* 272.0228.

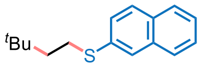

5c

(3,3-Dimethylbutyl)(naphthalen-2-yl)sulfane (5c). The title compound was synthesized according to the General Procedure C using tetraethylammonium bis(catecholato)-*tert*-butylsilicate (**1m**, 43.2 mg, 0.10 mmol, 1.0 equiv.), dineopentyl(vinyl)sulfonium triflate (**4**, 52.6 mg, 0.15 mmol, 1.5 equiv.), 2f-**UiO-PDI** (2.0 mg, 2 mol%) and DMF (1.0 mL). After photo reaction, naphthalene-2-thiol (40.1 mg, 0.25 mmol, 2.5 equiv.), K_3PO_4 (31.8 mg, 0.15 mmol, 1.5 equiv.) in DMF (0.20 mL) were added. The reaction was performed under nitrogen atmosphere at 65 °C for 24 h. The crude mixture was purified by flash column chromatography using hexanes as the eluent to give 21.8 mg (0.089 mmol, 89% yield) of the title compound as a colorless oil.

^{1}H NMR ($CDCl_3$, 400 MHz): δ 7.82–7.67 (m, 4H), 7.52–7.37 (m, 3H), 3.08–2.92 (m, 2H), 1.67–1.56 (m, 2H), 0.96 (s, 9H).

^{13}**C NMR** ($CDCl_3$, 100 MHz): δ 134.6, 133.8, 131.6, 128.3, 127.7, 127.1, 127.0, 126.5, 126.0, 125.4, 43.2, 30.9, 29.2, 29.0.

HRMS (ESI) calculated for $[C_{16}H_{21}S]^+$ requires *m/z* 245.1358, found *m/z* 245.1357.

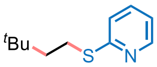

5d

2-((3,3-Dimethylbutyl)thio)pyridine (5d). The title compound was synthesized according to the General Procedure C using tetraethylammonium bis(catecholato)-*tert*-butylsilicate (**1m**, 43.2 mg, 0.10 mmol, 1.0 equiv.), dineopentyl(vinyl)sulfonium triflate (**4**, 52.6 mg, 0.15 mmol, 1.5 equiv.), 2f-**UiO-PDI** (2.0 mg, 2 mol%) and DMF (1.0 mL). After photo reaction, pyridine-2-thiol (27.8 mg, 0.25 mmol, 2.5 equiv.), K_3PO_4 (31.8 mg, 0.15 mmol, 1.5 equiv.) in DMF (0.20 mL) were added. The reaction was performed under nitrogen atmosphere at 65 °C for 24 h. The crude mixture was purified by flash column chromatography using hexanes/ethyl acetate (5:1 v/v) as the eluent to give 15.2 mg (0.078 mmol, 78% yield) of the title compound as a colorless oil.

^{1}**H NMR** ($CDCl_3$, 400 MHz): δ 8.42 (d, *J* = 4.4 Hz, 1H), 7.46 (td, *J* = 8.0, 2.0 Hz, 1H), 7.15 (d, *J* = 8.0 Hz, 1H), 7.02–6.89 (m, 1H), 3.16–3.09 (m, 2H), 1.63–1.56 (m, 2H), 0.97 (s, 9H).

^{13}**C NMR** ($CDCl_3$, 100 MHz): δ 159.6, 149.5, 135.8, 122.0, 119.1, 43.4, 31.0, 29.2, 26.0.

HRMS (EI) calculated for $[C_{11}H_{17}NS]^+$ requires *m/z* 195.1082, found *m/z* 195.1080 .

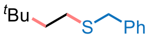

5e

Benzyl(3,3-dimethylbutyl)sulfane (5e). The title compound was synthesized according to the General Procedure C using tetraethylammonium bis(catecholato)-*tert*-butylsilicate (**1m**, 43.2 mg, 0.10 mmol, 1.0 equiv.), dineopentyl(vinyl)sulfonium triflate (**4**, 52.6 mg, 0.15 mmol, 1.5 equiv.), 2f-**UiO-PDI** (2.0 mg, 2 mol%) and DMF (1.0 mL). After photo reaction, phenylmethanethiol (31.1 mg, 0.25 mmol, 2.5 equiv.), K_3PO_4 (31.8 mg, 0.15 mmol, 1.5 equiv.) in DMF (0.20 mL) were added. The reaction was performed under nitrogen atmosphere at 65 °C for 24 h. The crude mixture was purified by flash column chromatography using hexanes as the eluent to give 17.7 mg (0.085 mmol, 85% yield) of the title compound as a colorless oil.

^{1}H NMR ($CDCl_3$, 400 MHz): δ 7.43–7.28 (m, 4H), 7.27–7.21 (m, 1H), 3.71 (s, 2H), 2.45–2.28 (m, 2H), 1.52–1.41 (m, 2H), 0.86 (s, 9H).

^{13}C NMR ($CDCl_3$, 100 MHz): δ 138.6, 128.8, 128.4, 126.9, 43.6, 36.2, 30.7, 29.1, 26.8.

HRMS (EI) calculated for $[C_{13}H_{20}S]^+$ requires *m/z* 208.1280, found *m/z* 208.1281.

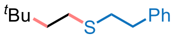

5f

(3,3-Dimethylbutyl)(phenethyl)sulfane (5f). The title compound was synthesized according to the General Procedure C using tetraethylammonium bis(catecholato)-*tert*-butylsilicate (**1m**, 43.2 mg, 0.10 mmol, 1.0 equiv.), dineopentyl(vinyl)sulfonium triflate (**4**, 52.6 mg, 0.15 mmol, 1.5 equiv.), 2f-**UiO-PDI** (2.0 mg, 2 mol%) and DMF (1.0 mL). After photo reaction, 2-phenylethane-1-thiol (34.6 mg, 0.25 mmol, 2.5 equiv.), K_3PO_4 (31.8 mg, 0.15 mmol, 1.5 equiv.) in DMF (0.20 mL) were added. The reaction was performed under nitrogen atmosphere at 65 °C for 24 h. The crude mixture was purified by flash column chromatography using hexanes as the eluent to give 19.6 mg (0.088 mmol, 88% yield) of the title compound as a colorless oil.

^{1}H NMR ($CDCl_3$, 400 MHz): δ 7.36–7.27 (m, 2H), 7.25–7.18 (m, 3H), 2.94–2.85 (m, 2H), 2.85–2.72 (m, 2H), 2.56–2.37 (m, 2H), 1.53–1.44 (m, 2H), 0.90 (s, 9H).

^{13}C NMR ($CDCl_3$, 100 MHz): δ 140.7, 128.5, 128.5, 126.3, 44.0, 36.4, 33.6, 30.7, 29.2, 27.8.

HRMS (ESI) calculated for $[C_{14}H_{23}S]^+$ requires *m/z* 223.1515, found *m/z* 223.1513.

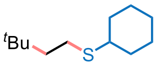

5g

Cyclohexyl(3,3-dimethylbutyl)sulfane (5g). The title compound was synthesized according to the General Procedure C using tetraethylammonium bis(catecholato)-*tert*-butylsilicate (**1m**, 43.2 mg, 0.10 mmol, 1.0 equiv.), dineopentyl(vinyl)sulfonium triflate (**4**, 52.6 mg, 0.15 mmol, 1.5 equiv.), 2f-**UiO-PDI** (2.0 mg, 2 mol%) and DMF (1.0 mL). After photo reaction, 2-cyclohexanethiol (29.1 mg, 0.25 mmol, 2.5 equiv.), K_3PO_4 (31.8 mg, 0.15 mmol, 1.5 equiv.) in DMF (0.20 mL) were added. The reaction was performed under nitrogen

atmosphere at 65 °C for 24 h. The crude mixture was purified by flash column chromatography using hexanes as the eluent to give 15.0 mg (0.075 mmol, 75% yield) of the title compound as a colorless oil.

^{1}H NMR ($CDCl_3$, 400 MHz): δ 2.72–2.56 (m, 1H), 2.56–2.37 (m, 2H), 2.06–1.87 (m, 2H), 1.87–1.70 (m, 2H), 1.67–1.58 (m, 1H), 1.53–1.42 (m, 2H), 1.37–1.20 (m, 5H), 0.90 (s, 9H).

^{13}C NMR ($CDCl_3$, 100 MHz): δ 44.3, 43.4, 33.7, 30.8, 29.2, 26.2, 25.9, 25.6.

HRMS (EI) calculated for $[C_{12}H_{24}S]^+$ requires *m*/*z* 200.1593, found *m*/*z* 200.1595.

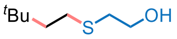

5h

2-((3,3-Dimethylbutyl)thio)ethan-1-ol (5h). The title compound was synthesized according to the General Procedure C using tetraethylammonium bis(catecholato)-*tert*-butylsilicate (**1m**, 43.2 mg, 0.10 mmol, 1.0 equiv.), dineopentyl(vinyl)sulfonium triflate (**4**, 52.6 mg, 0.15 mmol, 1.5 equiv.), 2f-**UiO-PDI** (2.0 mg, 2 mol%) and DMF (1.0 mL). After photo reaction, 2-mercaptoethanol (19.5 mg, 0.25 mmol, 2.5 equiv.), K_3PO_4 (31.8 mg, 0.15 mmol, 1.5 equiv.) in DMF (0.20 mL) were added. The reaction was performed under nitrogen atmosphere at 65 °C for 24 h. The crude mixture was purified by flash column chromatography using hexanes/ethyl acetate (5:1 v/v) as the eluent to give 9.7 mg (0.060 mmol, 60% yield) of the title compound as a colorless oil.

^{1}H NMR ($CDCl_3$, 400 MHz): δ 3.72 (t, *J* = 6.0 Hz, 2H), 2.83–2.65 (m, 2H), 2.56–2.41 (m, 2H), 1.60 (brs, 1H), 1.52–1.46 (m, 2H), 0.90 (s, 9H).

^{13}C NMR ($CDCl_3$, 100 MHz): δ 60.1, 44.1, 35.3, 30.8, 29.1, 27.1.

HRMS (EI) calculated for $[C_8H_{18}OS]^+$ requires *m*/*z* 162.1073, found *m*/*z* 162.1073.

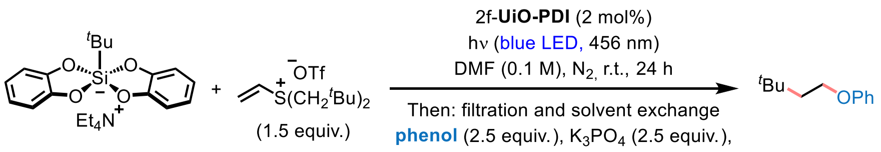

(3,3-Dimethylbutoxy)benzene (5i). The title compound was synthesized according to the General Procedure C using tetraethylammonium bis(catecholato)-*tert*-butylsilicate (**1m**, 43.2

mg, 0.10 mmol, 1.0 equiv.), dineopentyl(vinyl)sulfonium triflate (**4**, 52.6 mg, 0.15 mmol, 1.5 equiv.), 2f-**UiO-PDI** (2.0 mg, 2 mol%) and DMF (1.0 mL). After photo reaction, phenol (23.5 mg, 0.25 mmol, 2.5 equiv.), K_3PO_4 (53.1 mg, 0.25 mmol, 2.5 equiv.) in DMF (0.20 mL) were added. The reaction was performed under nitrogen atmosphere at 65 °C for 24 h. The crude mixture was purified by flash column chromatography using hexanes/ethyl acetate (50:1 v/v) as the eluent to give 9.8 mg (0.055 mmol, 55% yield) of the title compound as a colorless oil.

^{1}H NMR ($CDCl_3$, 400 MHz): δ 7.34–7.20 (m, 2H), 7.00–6.80 (m, 3H), 4.02 (t, J = 7.2 Hz, 2H), 1.73 (t, J = 7.2 Hz, 2H), 0.99 (s, 9H).

^{13}C NMR ($CDCl_3$, 100 MHz): δ 159.0, 129.4, 120.4, 114.5, 65.2, 42.4, 29.8, 29.7.

HRMS (EI) calculated for $[C_{12}H_{18}O]^+$ requires m/z 178.1352, found m/z 178.1352.

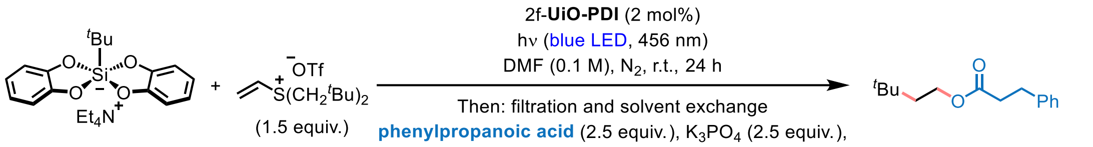

1m **4** **5j**, 70%

3,3-Dimethylbutyl 3-phenylpropanoate (5j). The title compound was synthesized according to the General Procedure C using tetraethylammonium bis(catecholato)-*tert*-butylsilicate (**1m**, 43.2 mg, 0.10 mmol, 1.0 equiv.), dineopentyl(vinyl)sulfonium triflate (**4**, 52.6 mg, 0.15 mmol, 1.5 equiv.), 2f-**UiO-PDI** (2.0 mg, 2 mol%) and DMF (1.0 mL). After photo reaction, 3-phenylpropanoic acid (37.6 mg, 0.25 mmol, 2.5 equiv.), K_3PO_4 (53.1 mg, 0.25 mmol, 2.5 equiv.) in DMF (0.20 mL) were added. The reaction was performed under nitrogen atmosphere at 65 °C for 24 h. The crude mixture was purified by flash column chromatography using hexanes/ethyl acetate (10:1 v/v) as the eluent to give 16.4 mg (0.070 mmol, 70% yield) of the title compound as a yellow oil.

^{1}H NMR ($CDCl_3$, 400 MHz): δ 7.31–7.25 (m, 2H), 7.25–7.16 (m, 3H), 4.12 (t, J = 8.0 Hz, 2H), 2.95 (t, J = 8.0 Hz, 2H), 2.61 (t, J = 8.0 Hz, 2H), 1.53 (t, J = 8.0 Hz, 2H), 0.92 (s, 9H).

^{13}C NMR ($CDCl_3$, 100 MHz): δ 173.0, 140.6, 128.5, 128.3, 126.2, 62.2, 41.7, 36.0, 31.0, 29.7, 29.5.

HRMS (ESI) calculated for $[C_{15}H_{23}O_2]^+$ requires m/z 235.1693, found m/z 235.1696.

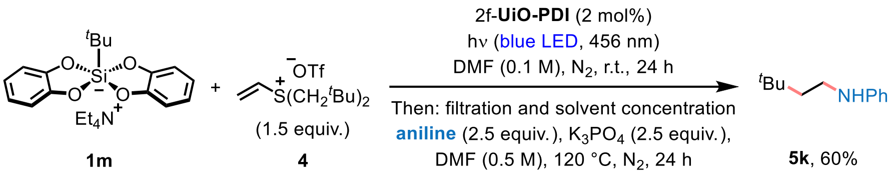

***N*-(3,3-dimethylbutyl)aniline (5k).**[14] The title compound was synthesized according to the General Procedure C using tetraethylammonium bis(catecholato)-*tert*-butylsilicate (**1m**, 43.2 mg, 0.10 mmol, 1.0 equiv.), dineopentyl(vinyl)sulfonium triflate (**4**, 52.6 mg, 0.15 mmol, 1.5 equiv.), 2f-**UiO-PDI** (2.0 mg, 2 mol%) and DMF (1.0 mL). After photo reaction, aniline (23.3 mg, 0.25 mmol, 2.5 equiv.), K_3PO_4 (53.1 mg, 0.25 mmol, 2.5 equiv.) in DMF (0.20 mL) were added. The reaction was performed under nitrogen atmosphere at 120 °C for 24 h. The crude mixture was purified by flash column chromatography using hexanes/ethyl acetate (5:1 v/v) as the eluent to give 10.6 mg (0.060 mmol, 60% yield) of the title compound as a colorless oil.

^{1}H NMR ($CDCl_3$, 400 MHz): δ 7.18 (t, *J* = 8.0 Hz, 2H), 6.70 (t, *J* = 7.2 Hz, 1H), 6.62 (d, *J* = 8.0 Hz, 2H), 3.21–3.00 (m, 2H), 1.54–1.49 (m, 3H), 0.97 (s, 9H).

^{13}C NMR ($CDCl_3$, 100 MHz): δ 148.3, 129.2, 117.3, 112.9, 43.4, 40.5, 30.0, 29.6.

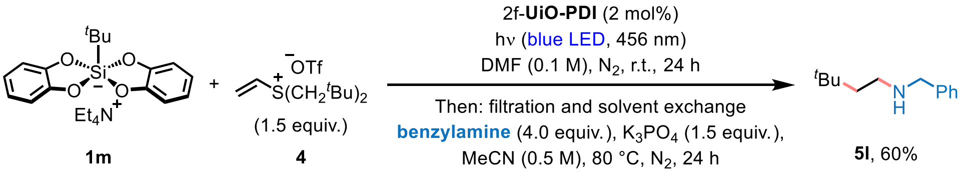

***N*-Benzyl-3,3-dimethylbutan-1-amine (3l).**[15] The title compound was synthesized according to the General Procedure C using tetraethylammonium bis(catecholato)-*tert*-butylsilicate (**1m**, 43.2 mg, 0.10 mmol, 1.0 equiv.), dineopentyl(vinyl)sulfonium triflate (**4**, 52.6 mg, 0.15 mmol, 1.5 equiv.), 2f-**UiO-PDI** (2.0 mg, 2 mol%) and DMF (1.0 mL). After photo reaction, benzylamine (42.9 mg, 0.40 mmol, 4.0 equiv.), K_3PO_4 (31.8 mg, 0.15 mmol, 1.5 equiv.) in MeCN (0.20 mL) were added. The reaction was performed under nitrogen atmosphere at 80 °C for 24 h. The crude mixture was purified by flash column chromatography using hexanes/ethyl acetate (1:1 v/v) as the eluent to give 11.5 mg (0.060 mmol, 60% yield) of the title compound as a colorless oil.

^{1}H NMR ($CDCl_3$, 400 MHz): δ 7.37–7.30 (m, 4H), 7.29–7.26 (m, 1H), 3.82 (s, 2H), 2.74–2.58 (m, 2H), 1.88 (br, 1H), 1.50–1.40 (m, 2H), 0.90 (s, 9H).

^{13}**C NMR** ($CDCl_3$, 100 MHz): δ 139.2, 128.5, 128.3, 127.2, 53.8, 45.4, 43.4, 29.9, 29.5.

HRMS (ESI) calculated for $[C_{13}H_{22}N]^+$ requires *m/z* 192.1747, found *m/z* 192.1746.

6. Stability Tests of 2f-UiO-PDI

6.1 Solvent Stability Studies

To a 10-mL centrifuge tube was added 2f-**UiO-PDI** crystal (5.0 mg) and solvent (5.0 mL). After the treatment with organic solvents or H_2O for 24 hours, 2f-**UiO-PDI** was recovered by centrifugation. The resulting powder was then subjected to PXRD testing.

6.2 Recycling Experiments of 2f-UiO-PDI

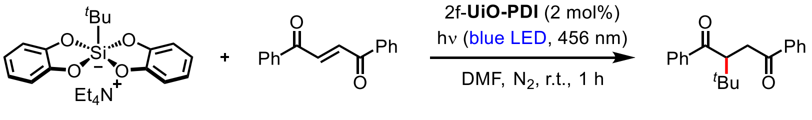

In the recycling experiments for the synthesis of **3m**, tetraethylammonium bis(catecholato)-*tert*-butylsilicate (**1m**, 43.2 mg, 0.1 mmol, 1.0 equiv.), *trans*-1,2-dibenzoylethylene (**2b**, 47.3 mg, 0.2 mmol, 2.0 equiv.), 2f-**UiO-PDI** (2.0 mg, 2 mol%) and DMF (1.0 mL) were used. After three freeze-pump-thaw cycles, the reaction mixture was irradiated by three 40-watt Kessil PR160L-456 blue-LED lamps at room temperature (with two fans) for 1 hour. After each cycle, the catalyst was dried and then used directly without further treatment.

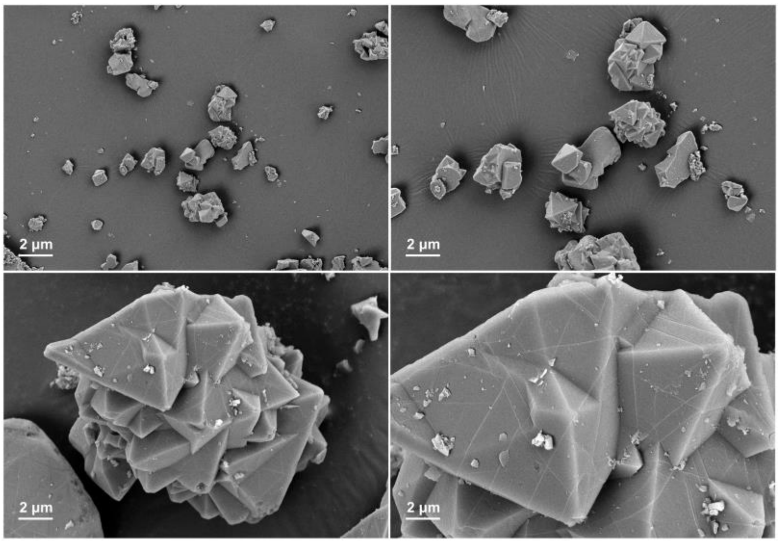

Figure S17. SEM images of 2f-**UiO-PDI** after recycling experiments.

7. Mechanistic Studies

7.1 Influence of TEMPO

To a 10-mL glass tube under N_2 atmosphere were added potassium (18-Crown-6) bis(catecholato)-benzylsilicate (**1f**, 63.9 mg, 0.10 mmol, 1.0 equiv.), *trans*-1,2-dibenzoylethylene (**2b**, 47.3 mg, 0.2 mmol, 2.0 equiv.), 2f-**UiO-PDI** (2.0 mg, 2 mol%), 2,2,6,6-tetramethylpiperidin-1-yl)oxyl (TEMPO, 34.4 mg, 0.22 mmol 2.2 equiv.) and DMF (1.0 mL) sequentially. The reaction mixture was irradiated by three 40-watt Kessil PR160L-456 blue-LED lamps at room temperature (with two fans) for 12 hours. After irradiation, ethyl acetate (5 mL) was added into the crude mixture; the resulting solution was filtered through a pad of silica gel and washed three times with ethyl acetate (3 × 10 mL), brine (3 × 10 mL), dried over Na_2SO_4 and evaporated under reduced pressure. The crude mixture was purified by flash column chromatography using hexanes as the eluent to give **6** 22.3 mg (0.09 mmol, 90% yield) of the title compound as a colorless oil.

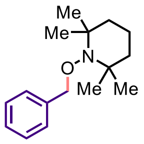

6

1-(Benzyloxy)-2,2,6,6-tetramethylpiperidine (6).[16]

^{1}H NMR ($CDCl_3$, 600 MHz): δ 7.38–7.33 (m, 4H), 7.30–7.26(m, 1H), 4.83 (s, 2H), 1.57–1.44 (m, 5H), 1.38–1.33 (m, 1H), 1.26 (s, 6H), 1.16 (s, 6H).

7.2 Deuterium Labelling Experiments

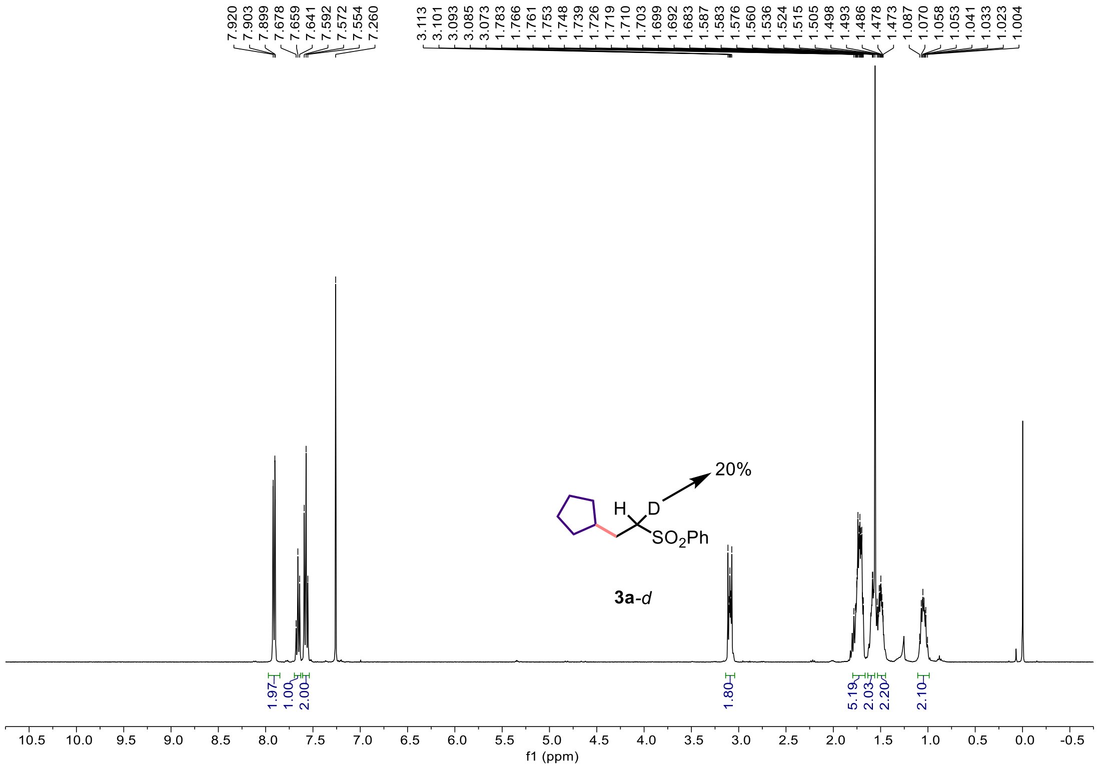

To a 10-mL glass tube under N_2 atmosphere were added tetraethylammonium bis(catecholato)-cyclopentylsilicate (**1a**, 44.4 mg, 0.10 mmol, 1.0 equiv.), (vinylsulfonyl)benzene (**2a**, 33.6 mg, 0.20 mmol, 2.0 equiv.), 2f-**UiO-PDI** (2.0 mg, 2 mol%), D_2O (4.0 mg, 0.20 mmol, 2.0 equiv.) and anhydrous DMF (1.0 mL) sequentially. The reaction mixture was irradiated by three 40-watt Kessil PR160L-456 blue-LED lamps at room temperature (with two fans) for 24 hours. After irradiation, ethyl acetate (5 mL) was added into the crude mixture; the resulting solution was filtered through a pad of silica gel and washed three times with ethyl acetate (3 × 10 mL), brine (3 × 10 mL), dried over Na_2SO_4 and evaporated under reduced pressure. The residue was purified by flash column chromatography on silica gel to afford the corresponding product. The D-incorporation was determined by ^1H NMR of the isolated product.

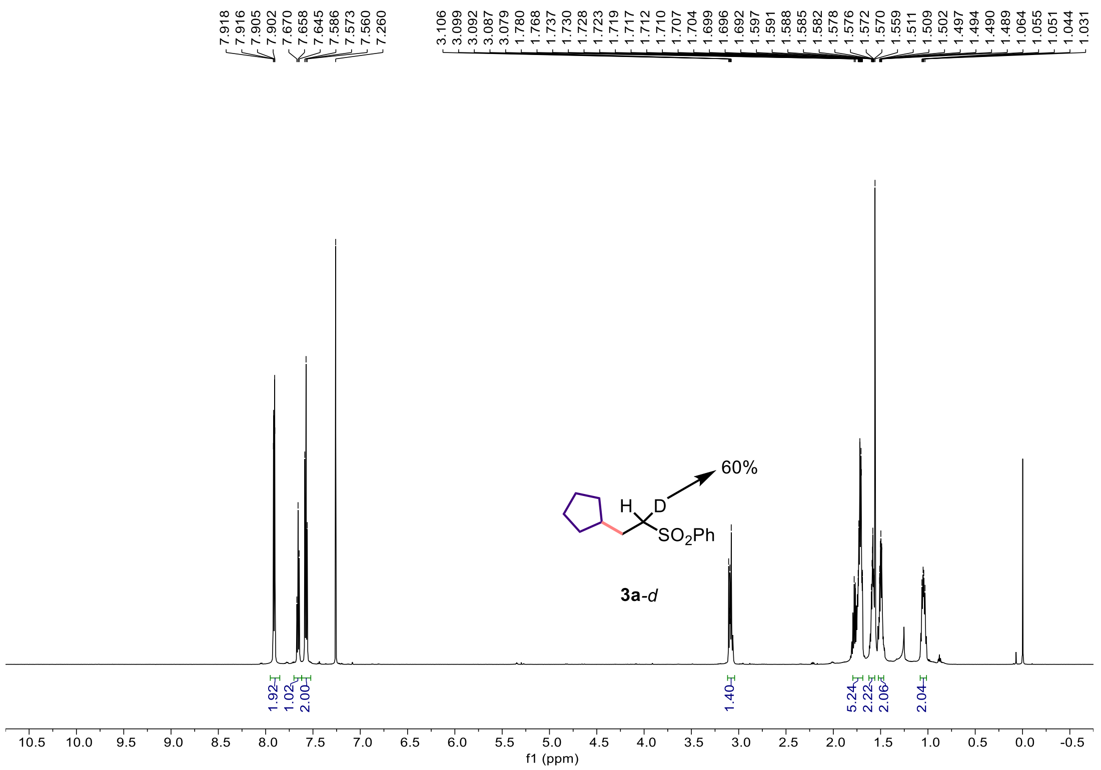

To a 10-mL glass tube under N_2 atmosphere were added tetraethylammonium bis(catecholato)-cyclopentylsilicate (**1a**, 44.4 mg, 0.10 mmol, 1.0 equiv.), (vinylsulfonyl)benzene (**2a**, 33.6 mg, 0.20 mmol, 2.0 equiv.), 2f-**UiO-PDI** (2.0 mg, 2 mol%), D_2O (10.0 mg, 0.50 mmol, 5.0 equiv.) and anhydrous DMF (1.0 mL) sequentially. The reaction mixture was irradiated by three 40-watt Kessil PR160L-456 blue-LED lamps at room temperature (with two fans) for 24 hours. After irradiation, ethyl acetate (5 mL) was added into the crude mixture; the resulting solution was filtered through a pad of silica gel and washed three times with ethyl acetate (3 × 10 mL), brine (3 × 10 mL), dried over Na_2SO_4 and evaporated under reduced pressure. The residue was purified by flash column chromatography on silica gel to afford the corresponding product. The D-incorporation was determined by ^{1}H NMR of the isolated product.

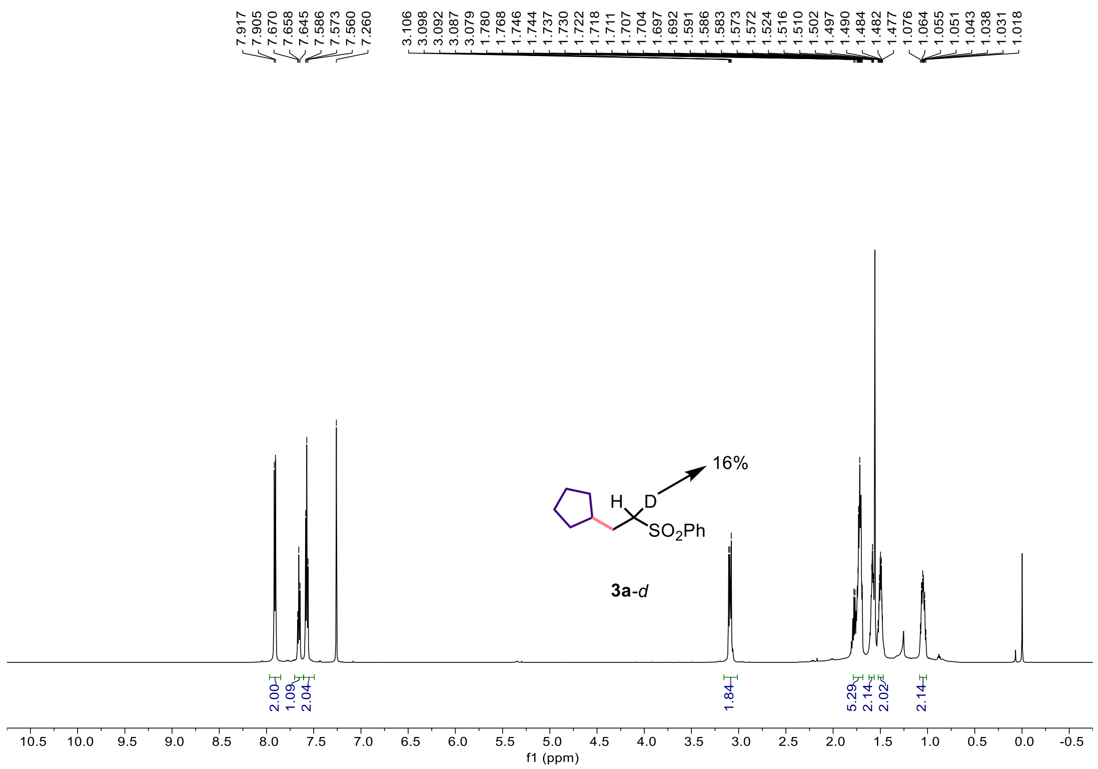

To a 10-mL glass tube under N_2 atmosphere were added tetraethylammonium bis(catecholato)-cyclopentylsilicate (**1a**, 44.4 mg, 0.10 mmol, 1.0 equiv.), (vinylsulfonyl)benzene (**2a**, 33.6 mg, 0.20 mmol, 2.0 equiv.), 2f-**UiO-PDI** (2.0 mg, 2 mol%), D_2O (10.0 mg, 0.50 mmol, 5.0 equiv.), H_2O (9.0 mg, 0.50 mmol, 5.0 equiv.) and anhydrous DMF (1.0 mL) sequentially. The reaction mixture was irradiated by three 40-watt Kessil PR160L-456 blue-LED lamps at room temperature (with two fans) for 24 hours. After irradiation, ethyl acetate (5 mL) was added into the crude mixture; the resulting solution was filtered through a pad of silica gel and washed three times with ethyl acetate (3 × 10 mL), brine (3 × 10 mL), dried over Na_2SO_4 and evaporated under reduced pressure. The residue was purified by flash column chromatography on silica gel to afford the corresponding product. The D-incorporation was determined by ^1H NMR of the isolated product.

7.3 Photoluminescence (PL) Studies

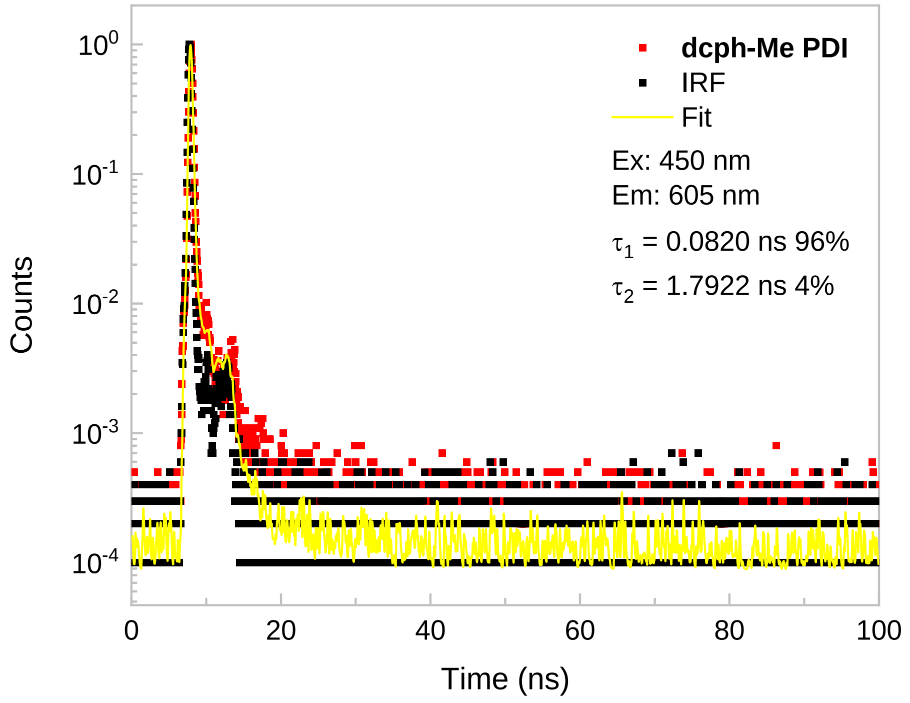

Figure S18. Emission decay of **dcph-Me PDI** measured at the excitation wavelength of 450 nm and emission wavelength of 605 nm.

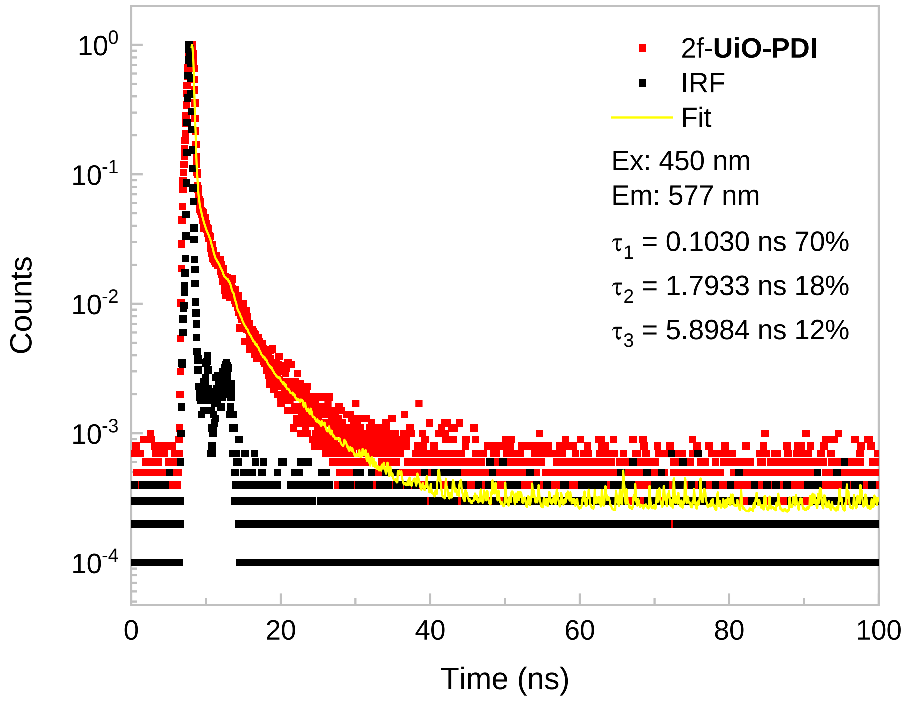

Figure S19. Emission decay of 2f-**UiO-PDI** measured at the excitation wavelength of 450 nm and emission wavelength of 577 nm.

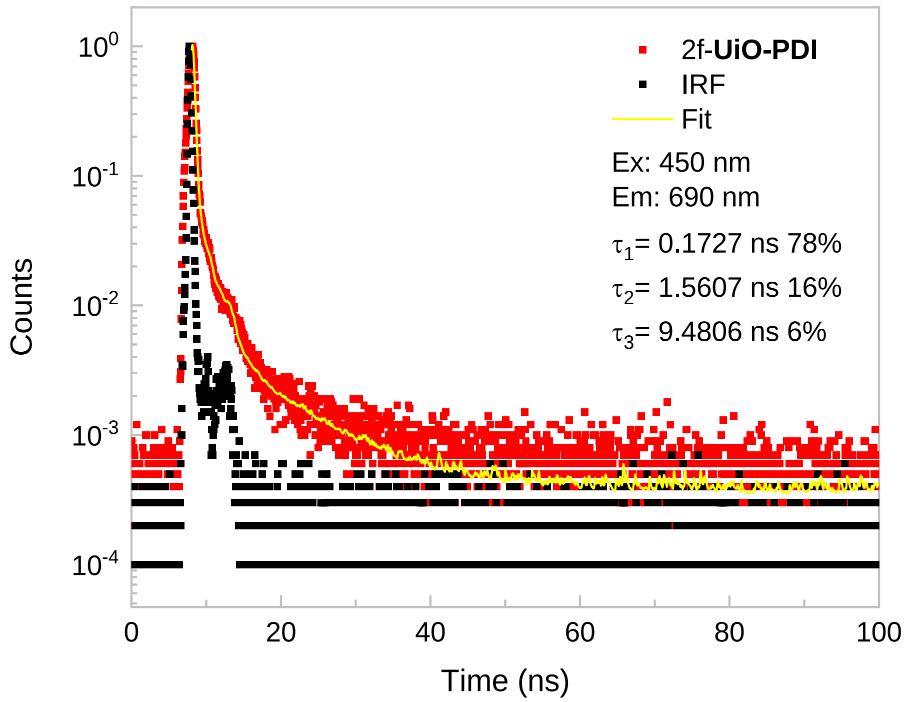

Figure S20. Emission decay of 2f-**UiO-PDI** measured at the excitation wavelength of 450 nm and emission wavelength of 690 nm.

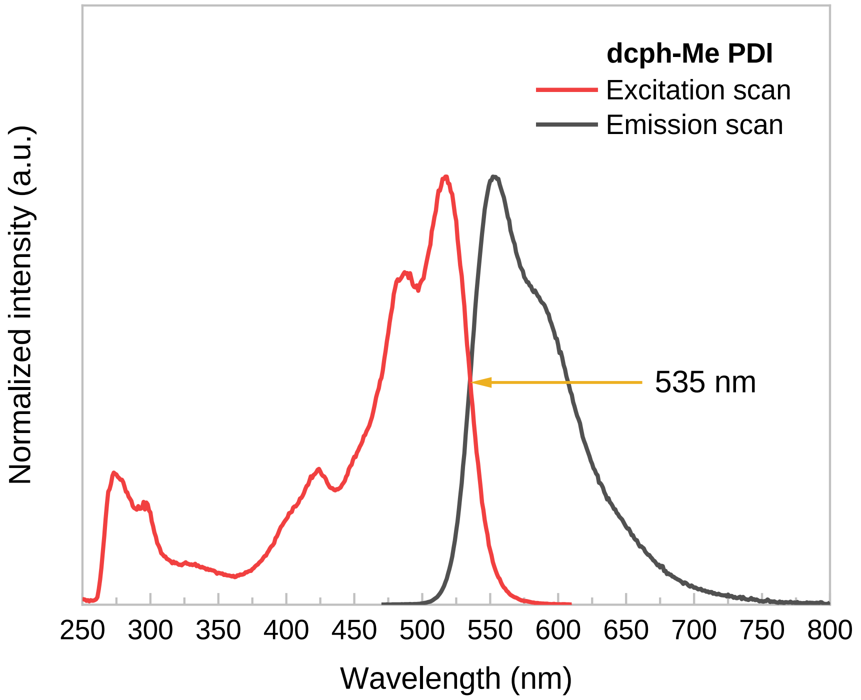

Figure S21. Excitation spectra (emission wavelength of 625 nm) and luminescence emission (excitation wavelength of 456 nm) of **dcph-Me-PDI** in DMF at room temperature.

7.4 Electron Paramagnetic Resonance (EPR) Studies

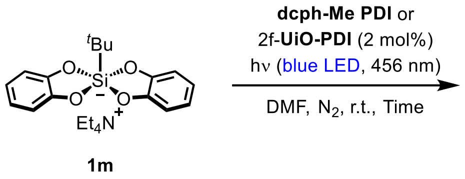

To a 10-mL flame-dried Schlenk tube with a stir bar were added **dcph-Me PDI** or 2f-**UiO-PDI** (2 mol%), tetraethylammonium bis(catecholato)-*tert*-butylsilicate (**1m**, 43.2 mg, 0.1 mmol, 1.0 equiv.), and DMF (1.0 mL) under N_2 atmosphere. Upon vigorous stirring for 5 min, the solution sample was transferred into an EPR tube for X-band EPR measurement at room temperature to obtain the black line. Subsequently, the reaction mixture was irradiated with one 40-watt blue-LED lamp (456 nm) at room temperature for 2.5, 5, 10, 20, and 30 min and then analyzed by EPR. EPR spectra were recorded on a Bruker ESR 5000 spectrometer operating at 9.431 GHz with a sweep time of 40 s.

7.5 Cyclic Voltammogram (CV) Studies

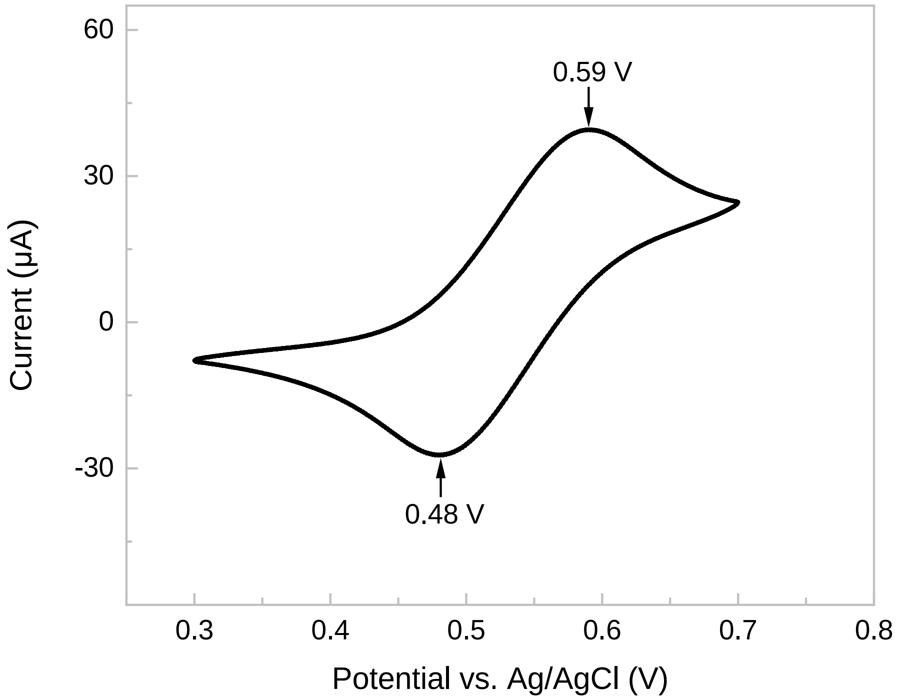

Figure S22. CV of ferrocenium [half-wave redox potential $E^0_{1/2}$ (Fc^+/Fc) = 0.54 V].

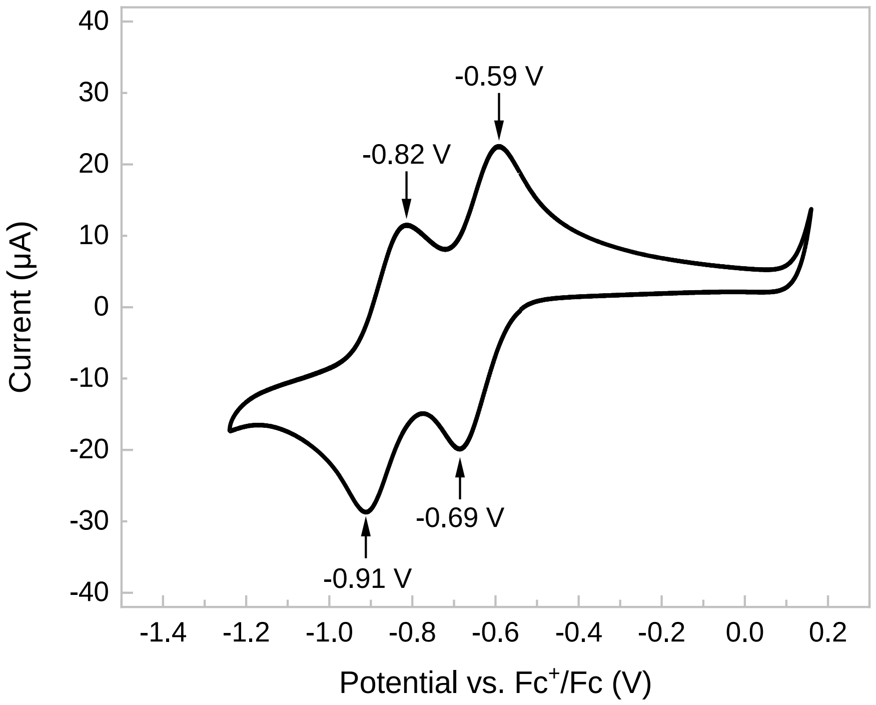

Figure S23. CV of **dcph-Me PDI** [half-wave redox potential $E_{1/2}$ (PDI/PDI$^{\bullet-}$)/V *vs*. Fc^+/Fc = –0.64 V] [half-wave redox potential $E_{1/2}$ (PDI$^{\bullet-}$/PDI^{2-})/V *vs*. Fc^+/Fc = –0.87 V].

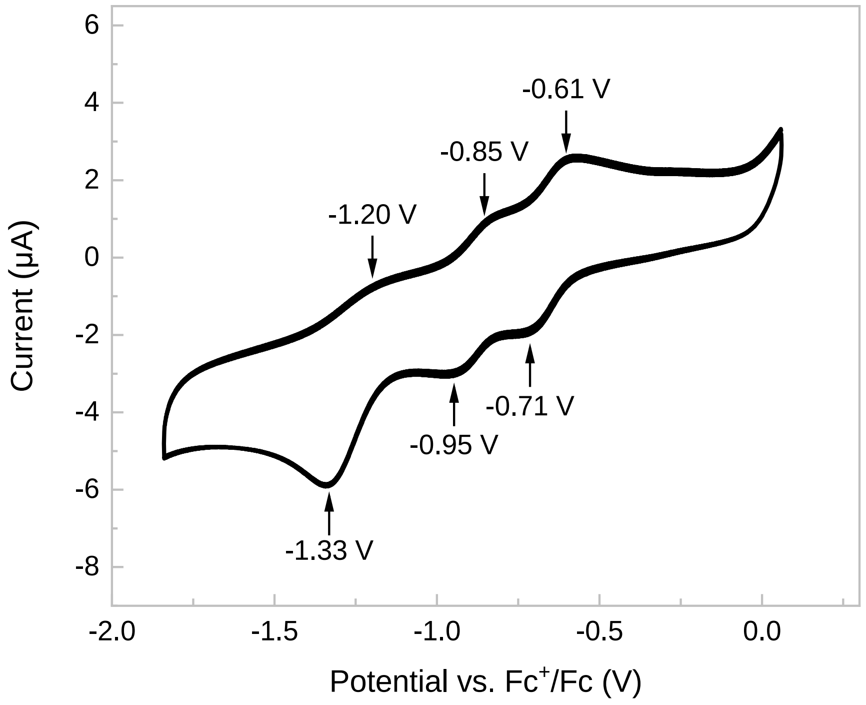

Figure S24. CV of 2f-**UiO-PDI** [half-wave redox potential $E_{1/2}$ (2f-**UiO-PDI**/2f-**UiO-PDI$^{\bullet-}$**)/V *vs*. Fc^+/Fc = –0.66 V] [half-wave redox potential $E_{1/2}$ (2f-**UiO-PDI$^{\bullet-}$**/2f-**UiO-PDI^{2-}**)/V *vs*. Fc^+/Fc = –0.90 V]

8. References

1. Corce, V.; Chamoreau, L.-M.; Derat, E.; Goddard, J.-P.; Ollivier, C.; Fensterbank, L. Silicates as Latent Alkyl Radical Precursors: Visible-Light Photocatalytic Oxidation of Hypervalent Bis-Catecholato Silicon Compounds. *Angew. Chem., Int. Ed.* **2015**, *54*, 11414–11418.
2. Berger, M.; Ma, D.; Baumgartner, Y.; Wong, T. H.-F.; Melchiorre, P. Stereoselective conjugate cyanation of enals by combining photoredox and organocatalysis. *Nat. Catal.* **2023**, *6*, 332–338.
3. Luo, W.; Fang, Y.; Zhang, L.; Xu, T.; Liu, Y.; Li, Y.; Jin, X.; Bao, J.; Wu, X.; Zhang, Z., Bromomethyl Silicate: A Robust Methylene Transfer Reagent for Radical-Polar Crossover Cyclopropanation of Alkenes. *Eur. J. Org. Chem.* **2020**, 1778–1781.
4. Huang, X.-L.; Cheng, Y.-Z.; You, S.-L. Visible-light enabled synthesis of cyclopropane-fused indolines dearomatization of indoles. *Org. Chem. Front.* **2022**, *9*, 5463–5468.
5. Wen, Z.-K.; Liu, X.-H.; Liu, Y.-F.; Chao, J.-B. Acid Promoted Direct Cross-Coupling of Methyl Ketones with Dimethyl Sulfoxide: Access to Ketoallyl Methylsulfides and -sulfones. *Org. Lett.* **2017**, *19*, 5798–5801.
6. Phelan, J. P.; Lang, S. B.; Compton, J. S.; Kelly, C. B.; Dykstra, R.; Gutierrez, O.; Molander, G. A. Redox-Neutral Photocatalytic Cyclopropanation via Radical/Polar Crossover. *J. Am. Chem. Soc.* **2018**, *140*, 8037–8047.
7. Paul, S.; Filippini, D.; Silvi, M. Polarity Transduction Enables the Formal Electronically Mismatched Radical Addition to Alkenes. *J. Am. Chem. Soc.* **2023**, *145*, 2773–2778.
8. Uozumi, Y.; Lee, S.-Y.; Hayashi, T. Asymmetric Functionalization of Bicycloalkenes by Catalytic Enantioposition-Selective Hydrosilylation. *Tetrahedron Lett.* **1992**, *33*, 7185–7188.
9. Avellanal-Zaballa, E.; Duran-Sampedro, G.; Prieto-Castaneda, A.; Agarrabeitia, A. R.; Garcia-Moreno, I.; Lopez-Arbeloa, I.; Banuelos, J.; Ortiz, M. J. Rational molecular design enhancing the photonic performance of red-emitting perylene bisimide dyes. *Phys. Chem. Chem. Phys.* **2017**, *19*, 13210–13218.
10. Zhou, W.; Wu, S.; Melchiorre, P. Tetrachlorophthalimides as Organocatalytic Acceptors for Electron Donor–Acceptor Complex Photoactivation. *J. Am. Chem. Soc.* **2022**, *144*, 8914–8919.
11. Xue, F.; Wang, F.; Liu, J.; Di, J.; Liao, Q.; Lu, H.; Zhu, M.; He, L.; He, H.; Zhang, D. Song, H., Liu, X.-Y., Qin, Y. A Desulfurative Strategy for the Generation of Alkyl Radicals Enabled by Visible-Light Photoredox Catalysis. *Angew. Chem., Int. Ed.* **2018**, *57*, 6667–6671.
12. Luo, Y.; Wei, Q.; Yang, L.; Zhou, Y.; Cao, W.; Su, Z.; Liu, X.; Feng, X. Enantioselective Radical Hydroacylation of *α,β*-Unsaturated Carbonyl Compounds with Aldehydes by Triplet Excited Anthraquinone. *ACS Catal.* **2022**, *12*, 12984–12992.
13. Wang, L.; Sun, J.; Xia, J.; Li, M.; Zhang, L.; Ma, R.; Zheng, G.; Zhang, Q. Visible light-mediated NHCs and photoredox co-catalyzed radical 1,2-dicarbonylation of alkenes for 1,4-diketones. *Sci China Chem.* **2022**, *65*, 1938–1944.
14. Sorribes, I.; Junge, K.; Beller, M. Direct catalytic N-alkylation of amines with carboxylic acids. *J. Am. Chem. Soc.* **2014**, *136*, 14314–14319.
15. Largeron, M.; Fleury, M.-B. A biomimetic electrocatalytic system for the atom-economical chemoselective synthesis of secondary amines. *Org. Lett.* **2009**, *11*, 883–886.
16. Liwosz, T. W.; Chemler, S. R. Copper-Catalyzed Oxidative Heck Reactions between Alkyltrifluoroborates and Vinyl Arenes. *Org. Lett.* **2013**, *15*, 3034–3037.

9. NMR Spectra

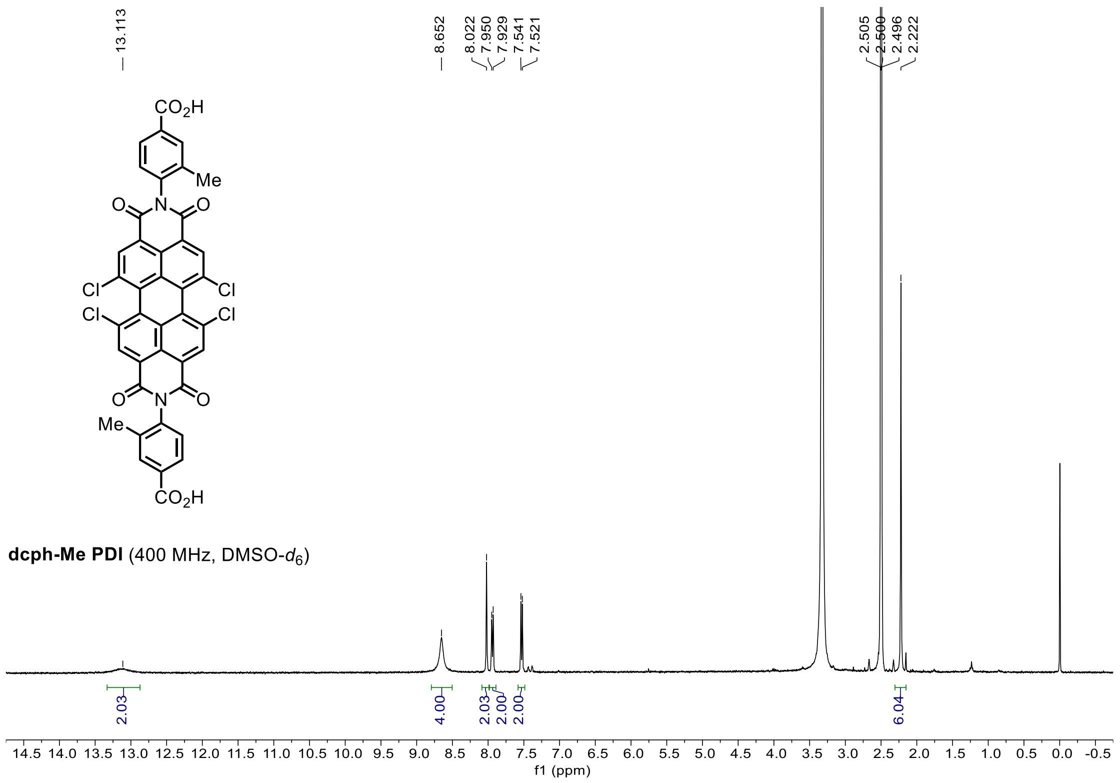

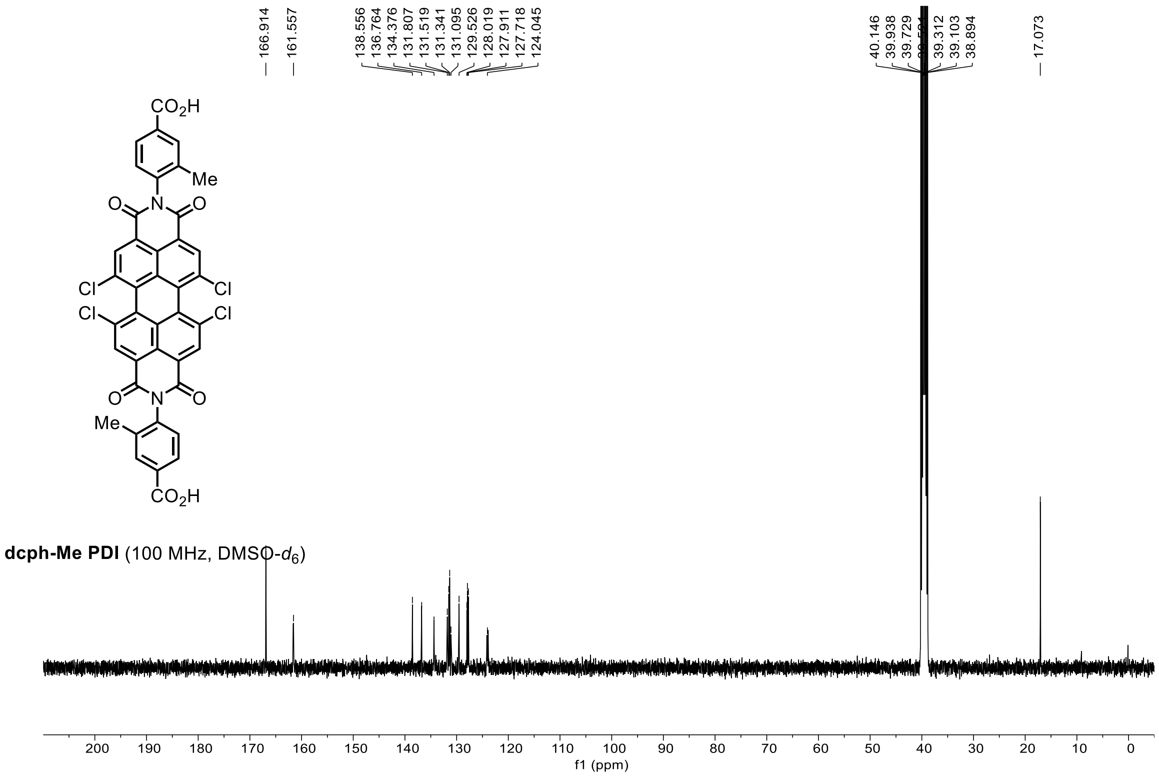

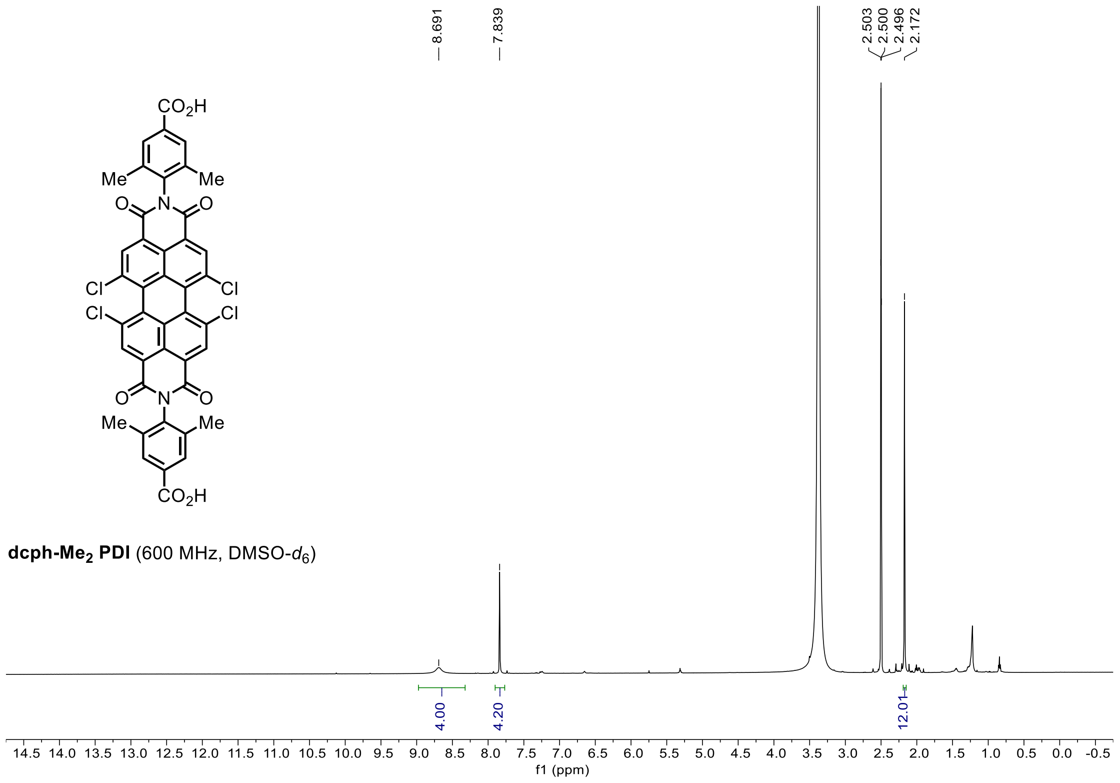

dcph-Me$_2$ PDI (600 MHz, DMSO-d_6)

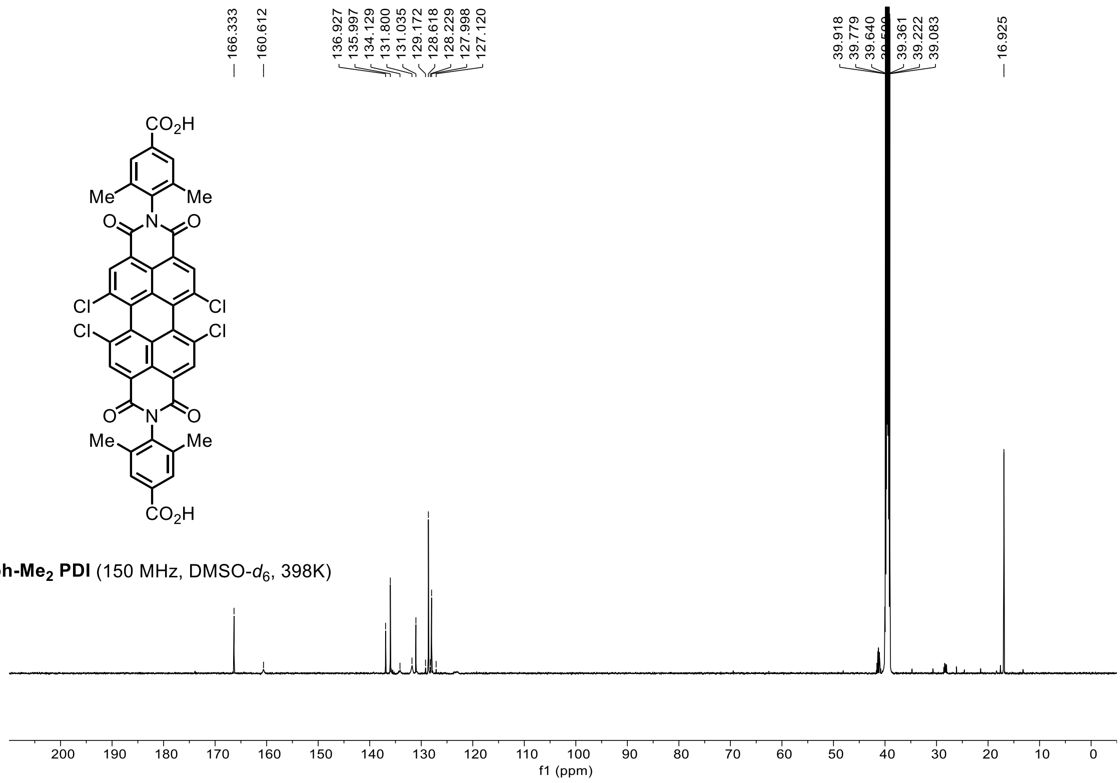

dcph-Me$_2$ PDI (150 MHz, DMSO-d_6, 398K)

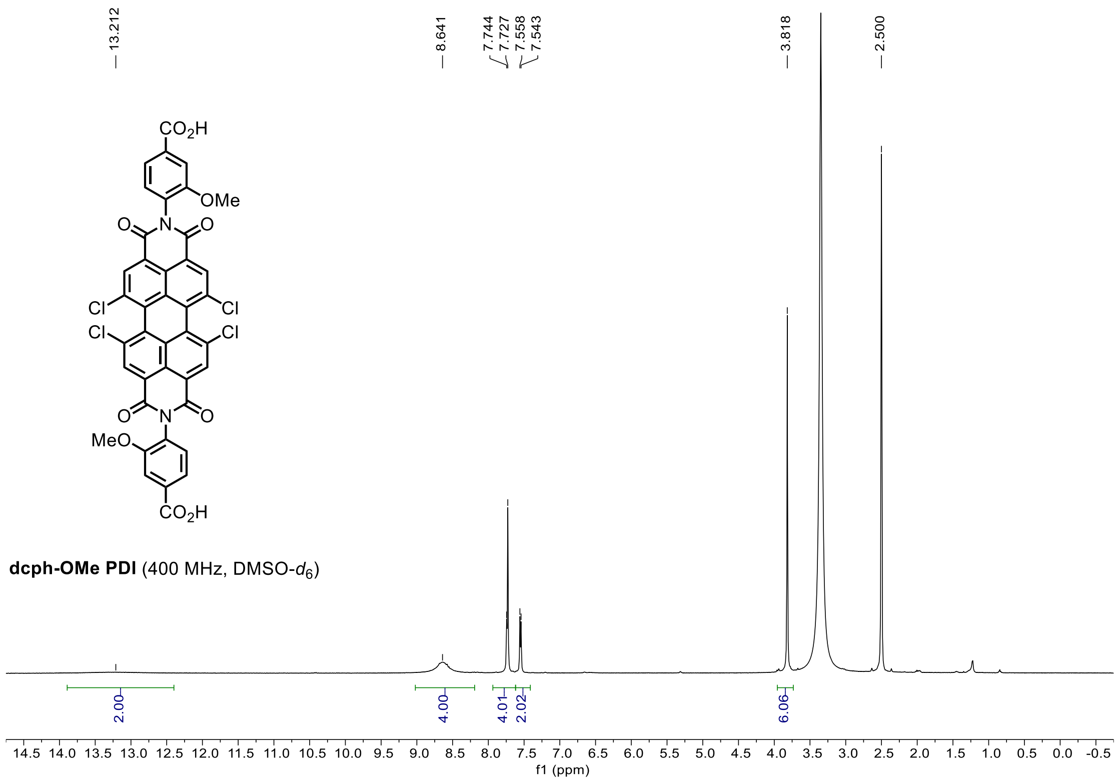

dcph-OMe PDI (400 MHz, DMSO-d_6)

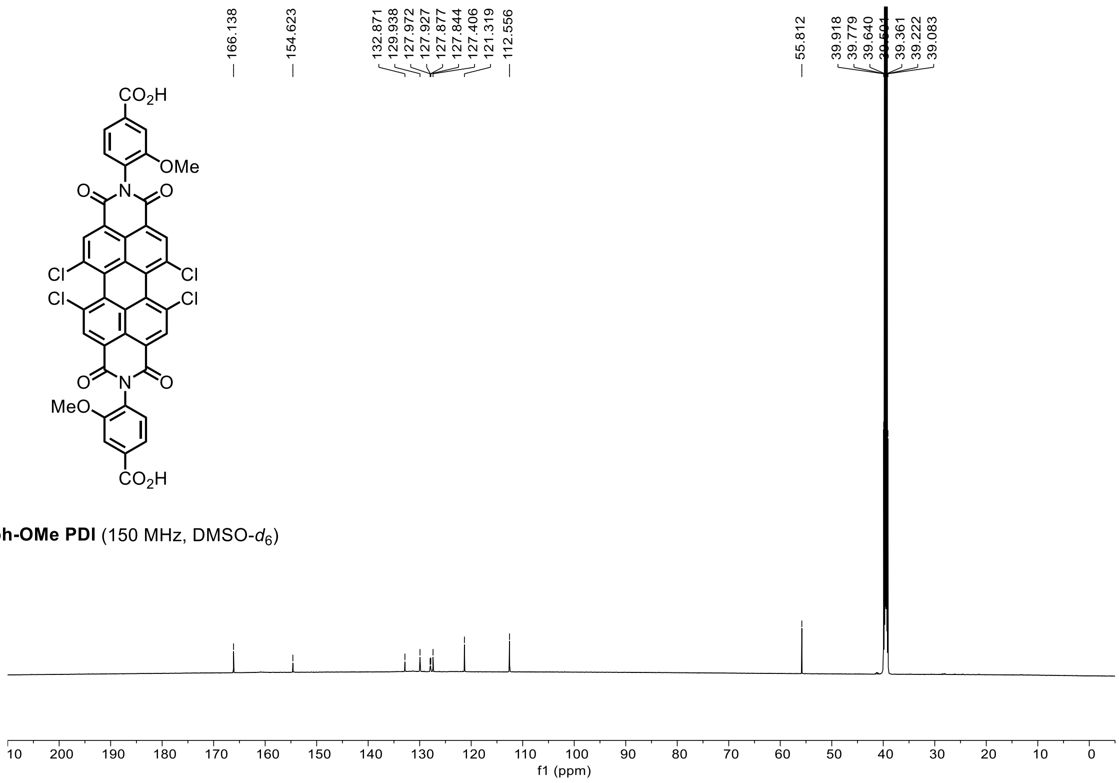

dcph-OMe PDI (150 MHz, DMSO-d_6)

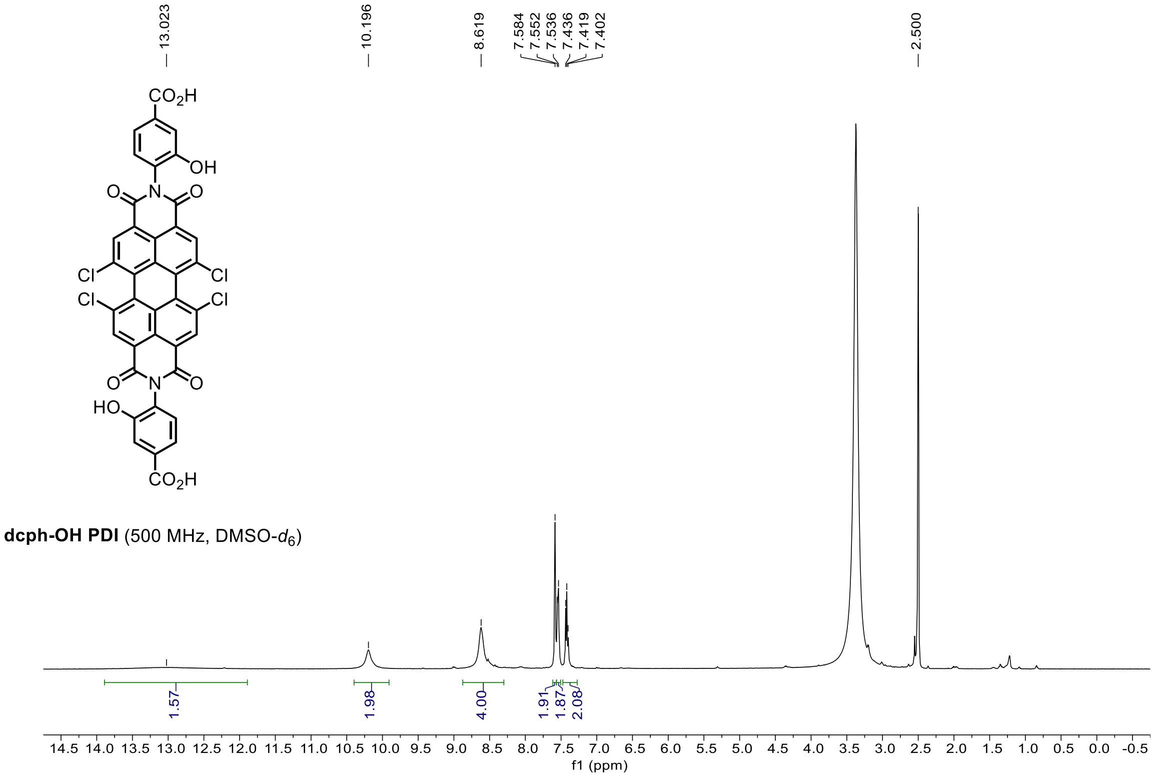

dcph-OH PDI (500 MHz, DMSO-d_6)

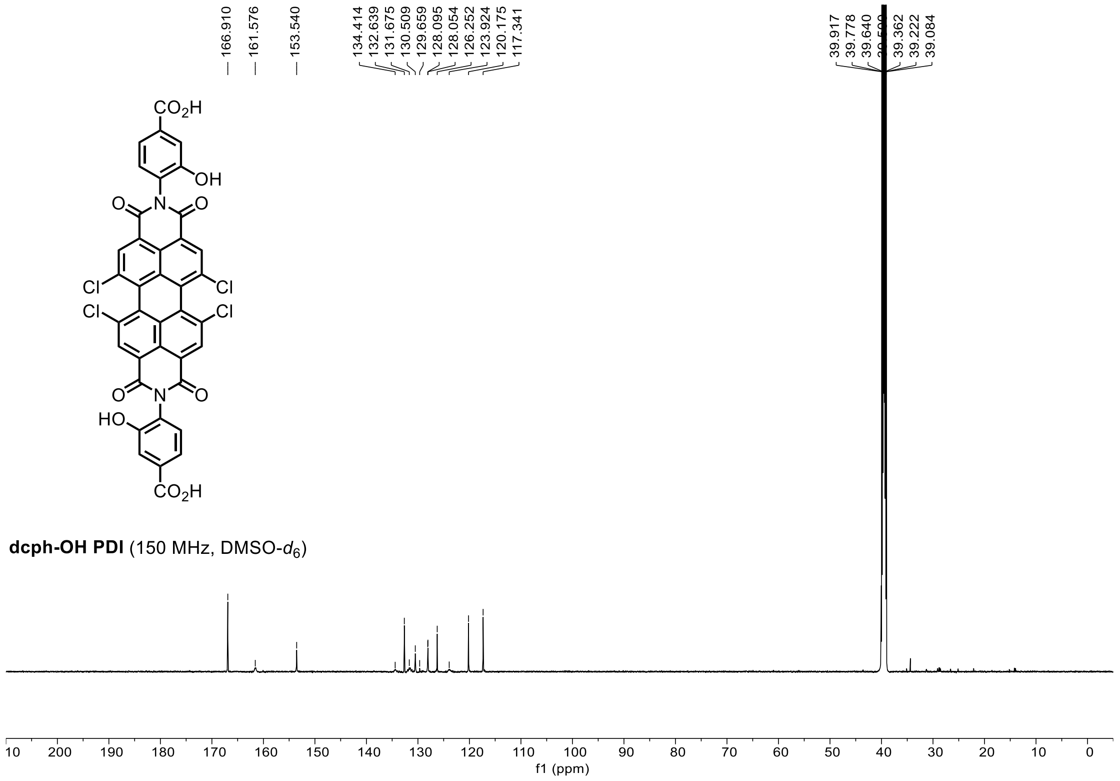

dcph-OH PDI (150 MHz, DMSO-d_6)

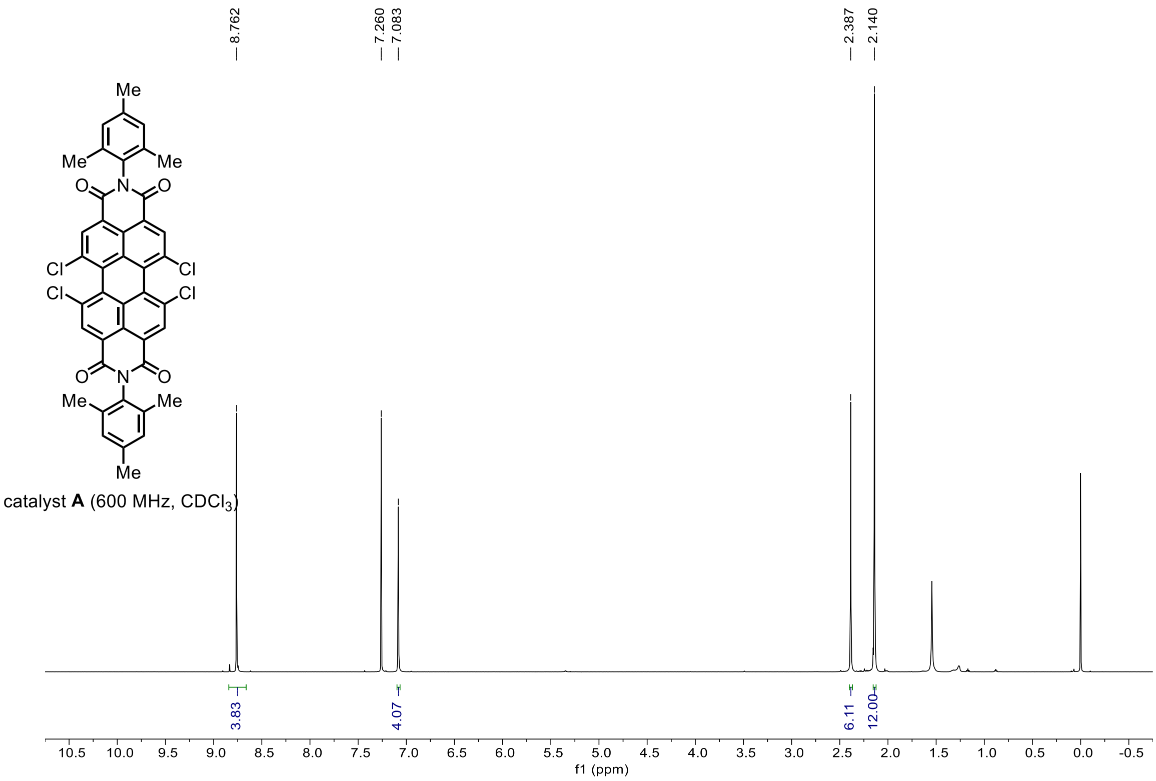

catalyst **A** (600 MHz, $CDCl_3$)

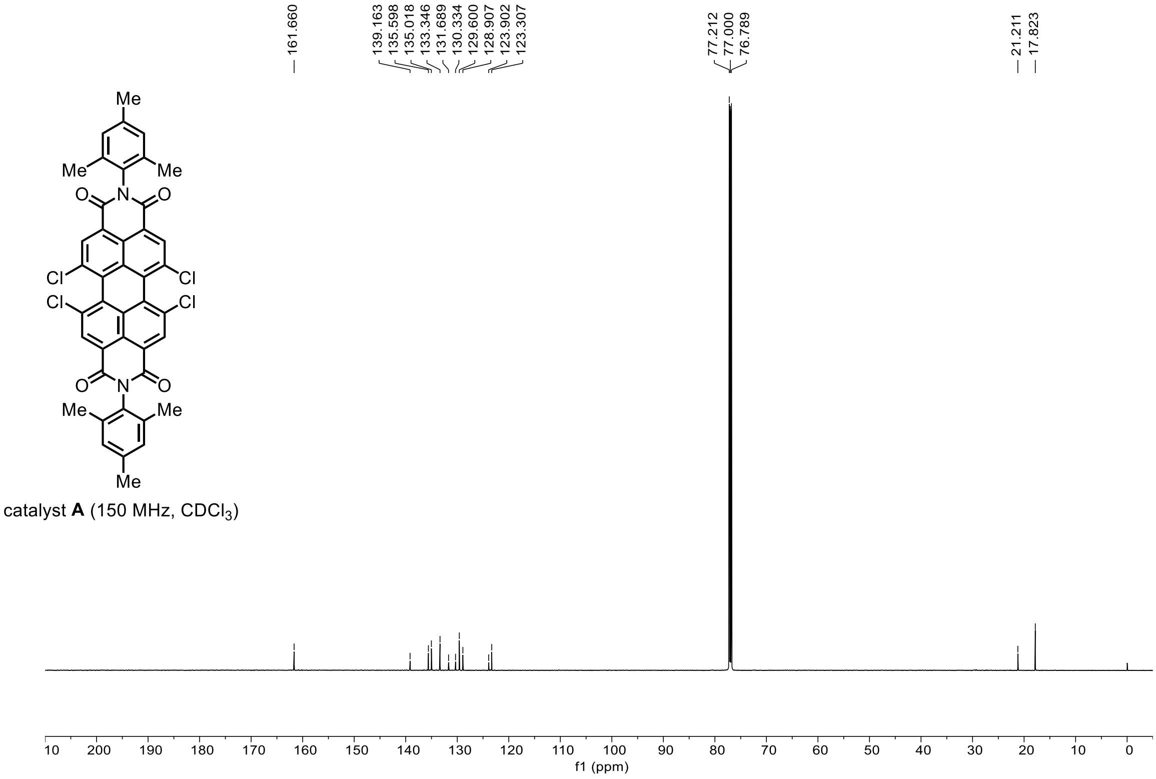

catalyst **A** (150 MHz, $CDCl_3$)

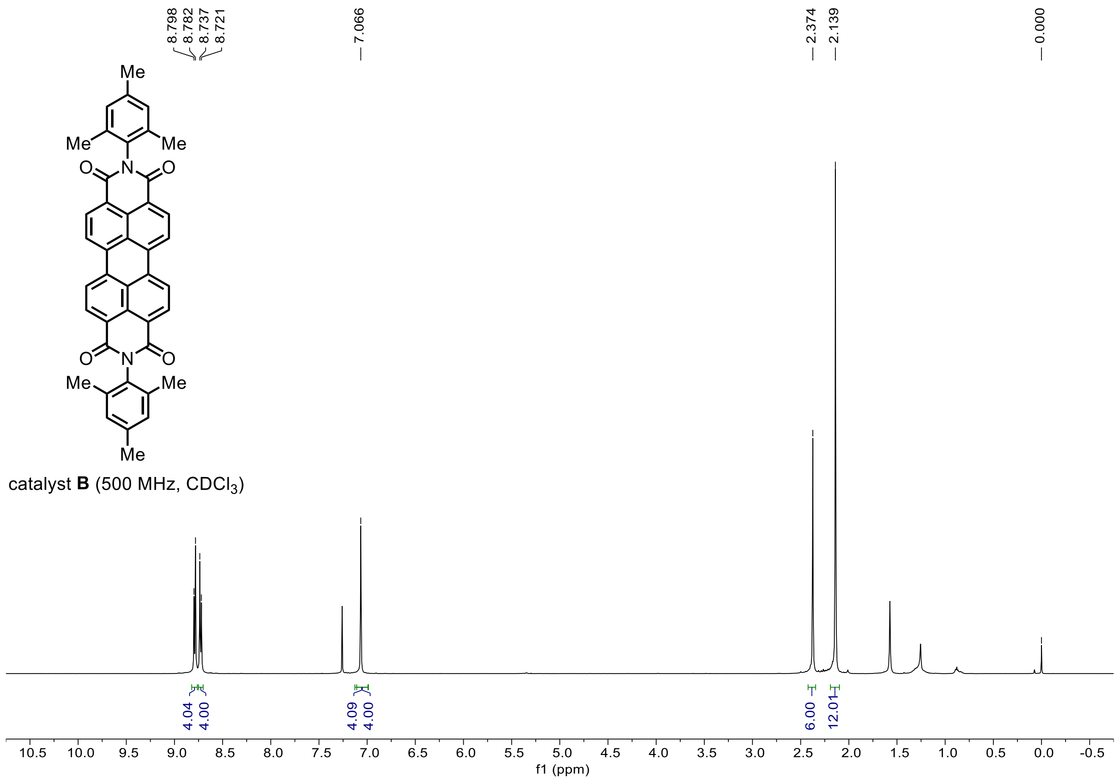
8.798
8.782
8.737
8.721
7.066
2.374
2.139
0.000
Me
Me
Me
O
N
O
O
N
O
Me
Me
Me
catalyst **B** (500 MHz, $CDCl_3$)
4.04
4.00
4.09
4.00
6.00
12.01
10.5 10.0 9.5 9.0 8.5 8.0 7.5 7.0 6.5 6.0 5.5 5.0 4.5 4.0 3.5 3.0 2.5 2.0 1.5 1.0 0.5 0.0 -0.5
f1 (ppm)

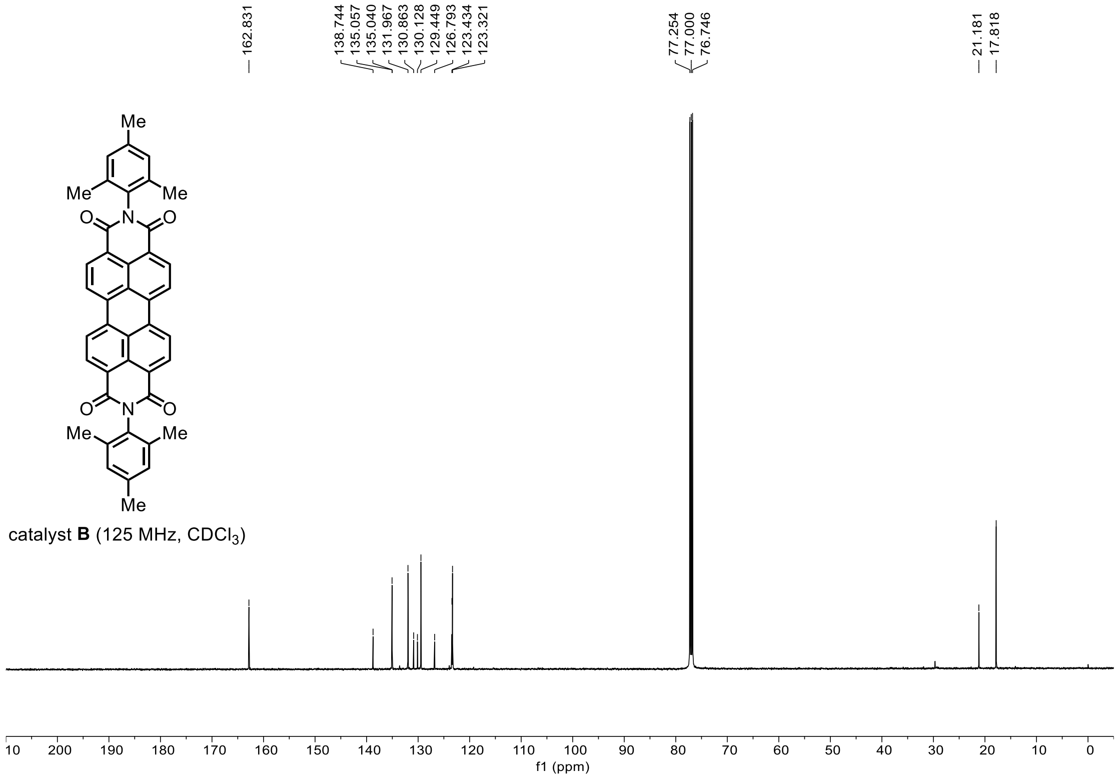
162.831
138.744
135.057
135.040
131.967
130.863
130.128
129.449
126.793
123.434
123.321
77.254
77.000
76.746
21.181
17.818
Me
Me
Me
O
N
O
O
N
O
Me
Me
Me
catalyst **B** (125 MHz, $CDCl_3$)
10 200 190 180 170 160 150 140 130 120 110 100 90 80 70 60 50 40 30 20 10 0
f1 (ppm)

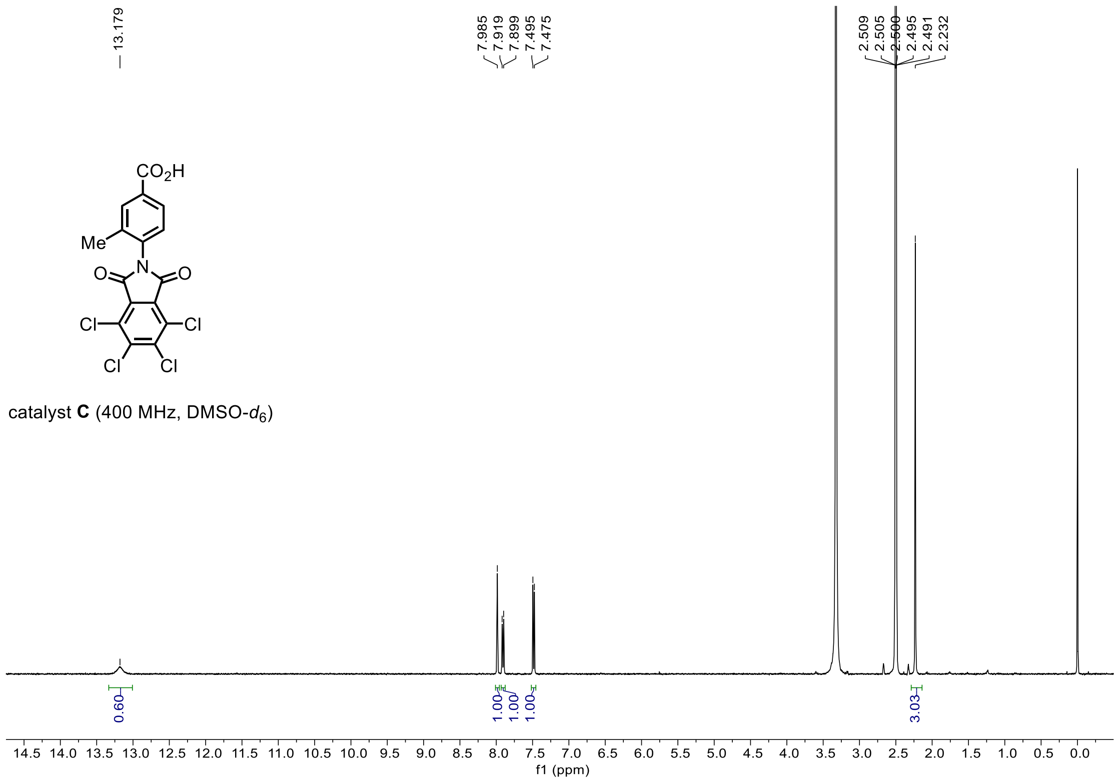
13.179
7.985
7.919
7.899
7.495
7.475
2.509
2.505
2.500
2.495
2.491
2.232
CO2H
Me
O
N
O
Cl
Cl
Cl
Cl
catalyst C (400 MHz, DMSO-d6)
0.60
1.00
1.00
1.00
3.03
f1 (ppm)

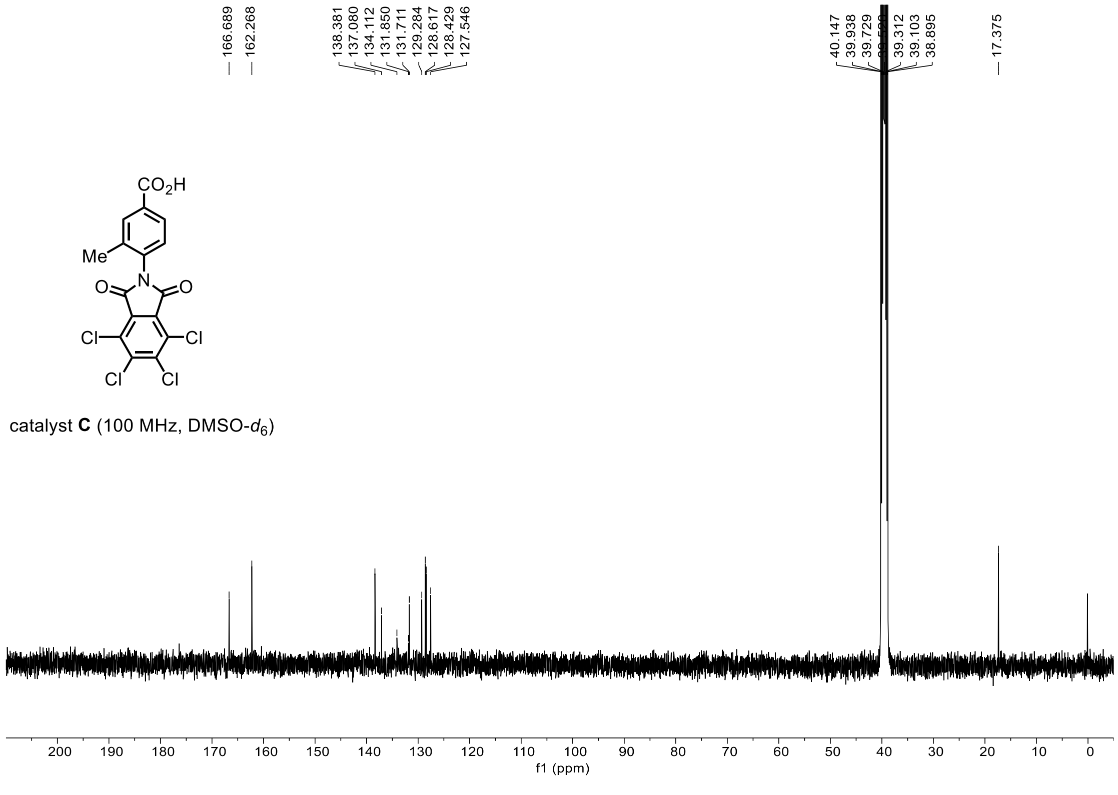
166.689
162.268
138.381
137.080
134.112
131.850
131.711
129.284
128.617
128.429
127.546
40.147
39.938
39.729
39.312
39.103
38.895
17.375
CO2H
Me
O
N
O
Cl
Cl
Cl
Cl
catalyst C (100 MHz, DMSO-d6)
f1 (ppm)

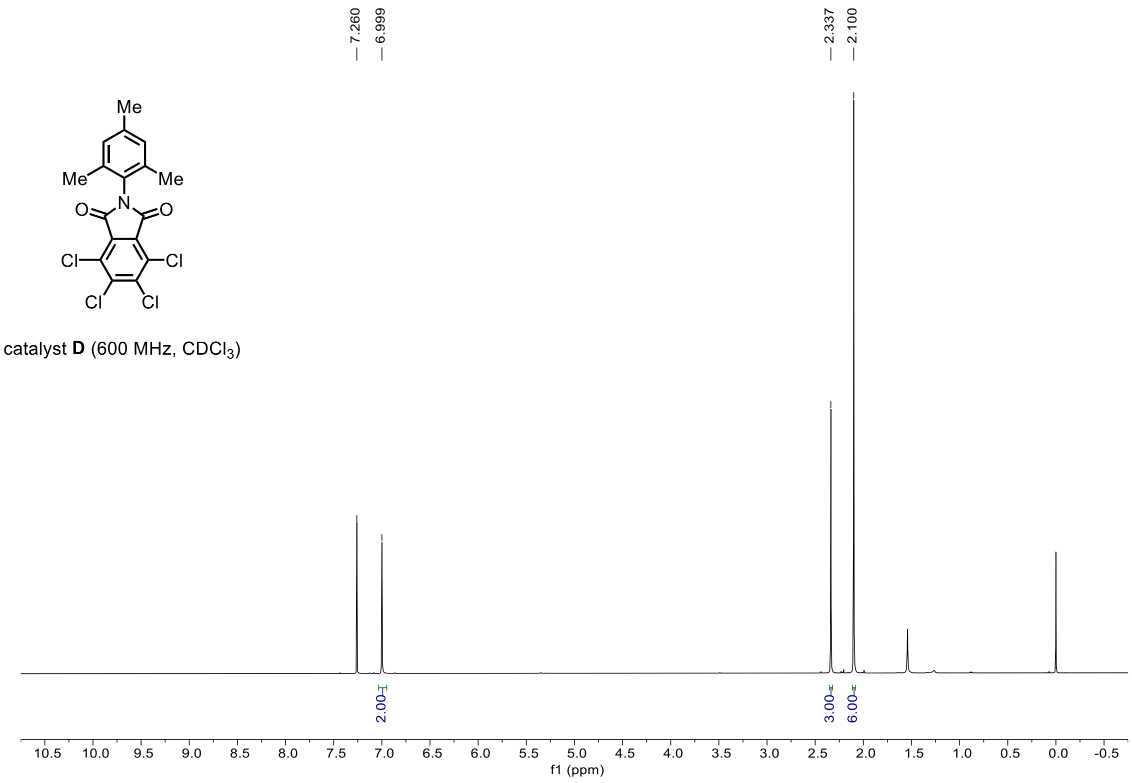

catalyst **D** (600 MHz, $CDCl_3$)

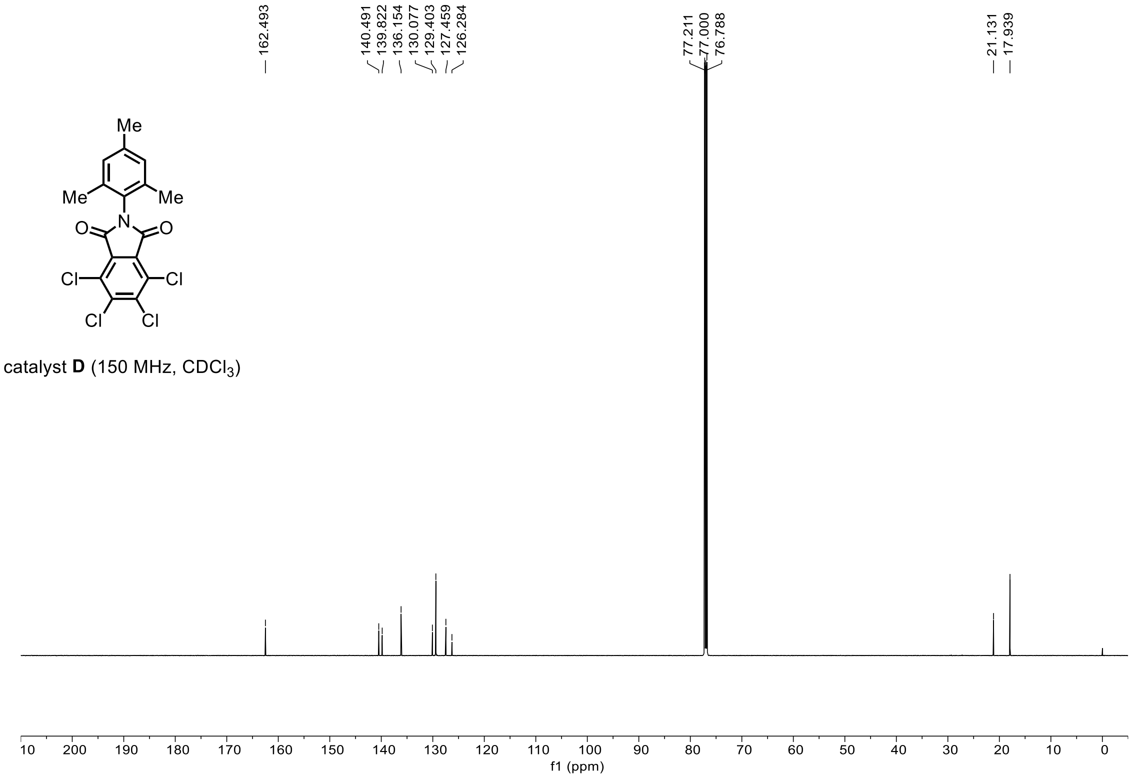

catalyst **D** (150 MHz, $CDCl_3$)

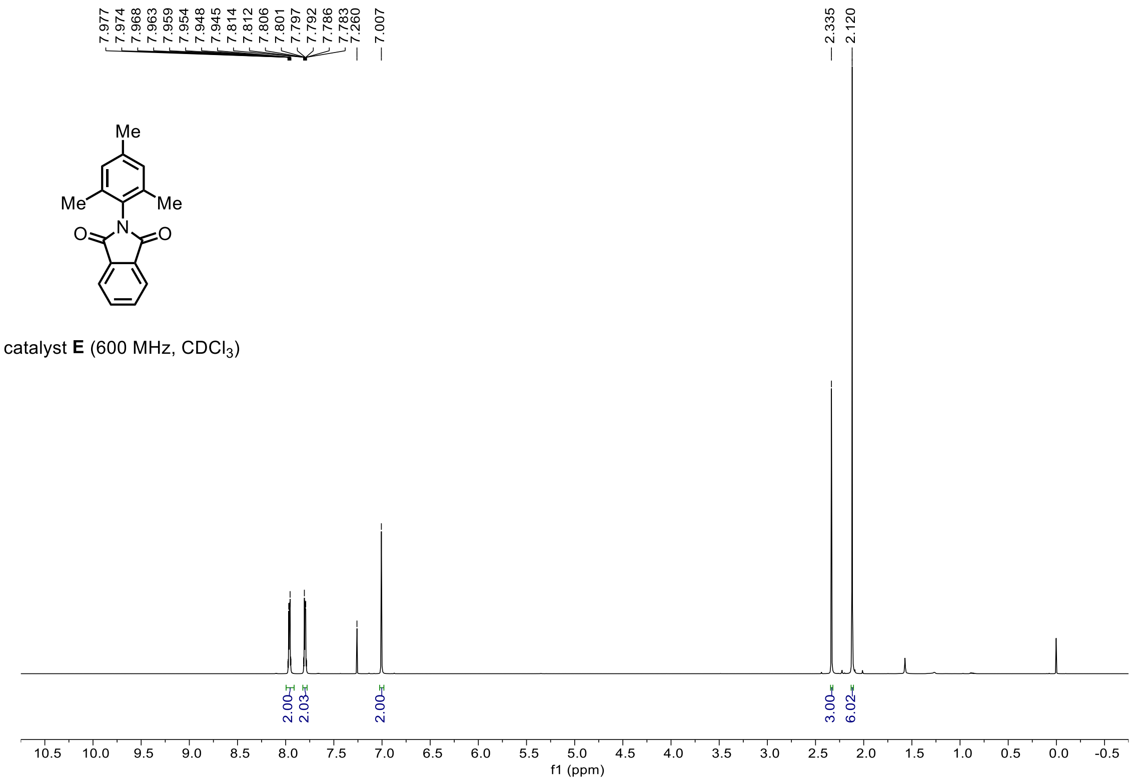

catalyst **E** (600 MHz, $CDCl_3$)

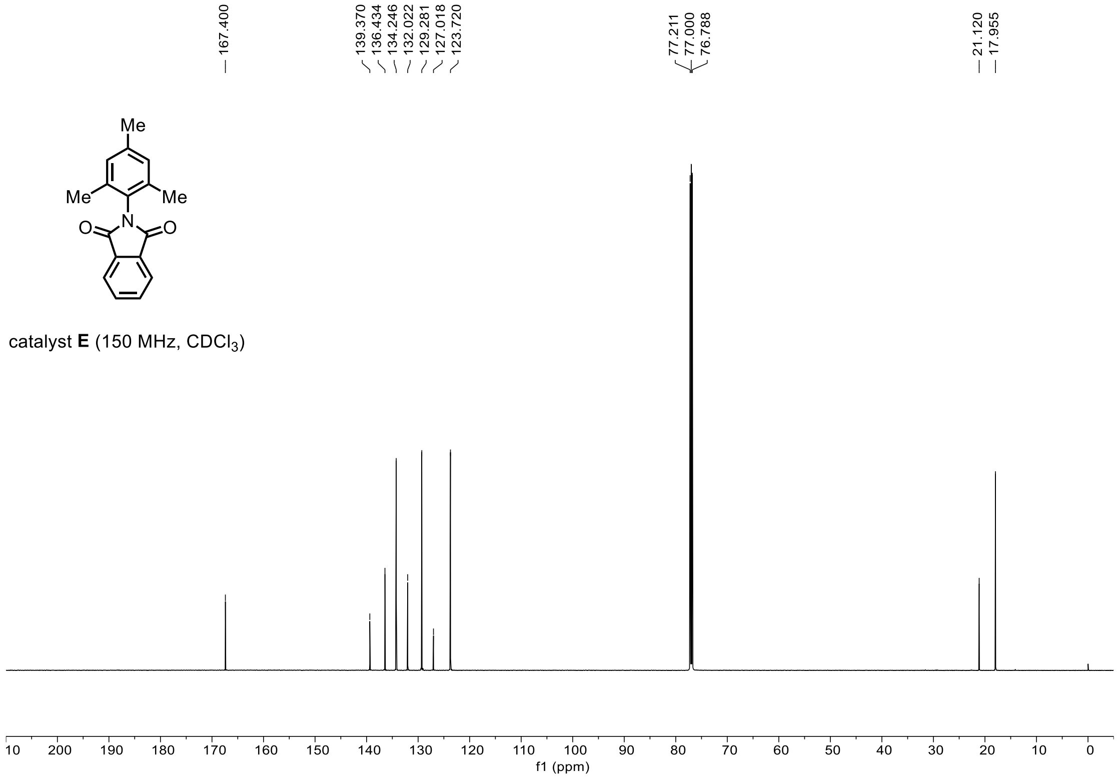

catalyst **E** (150 MHz, $CDCl_3$)

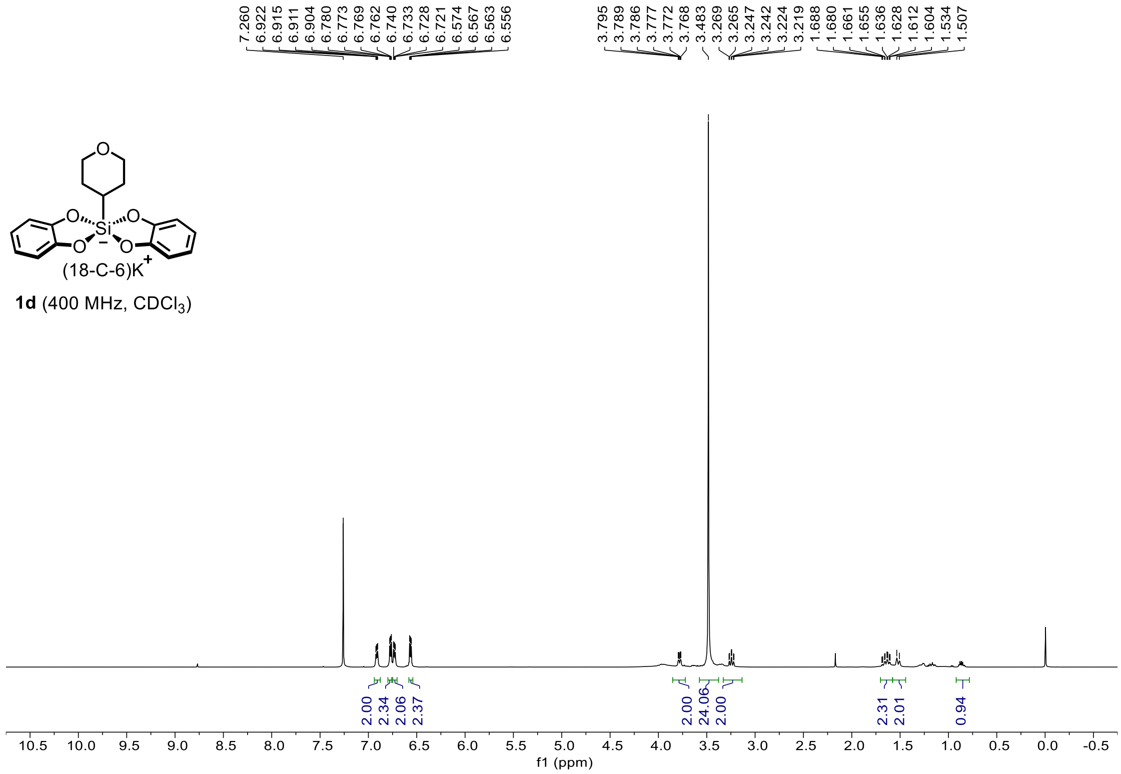

1d (400 MHz, $CDCl_3$)

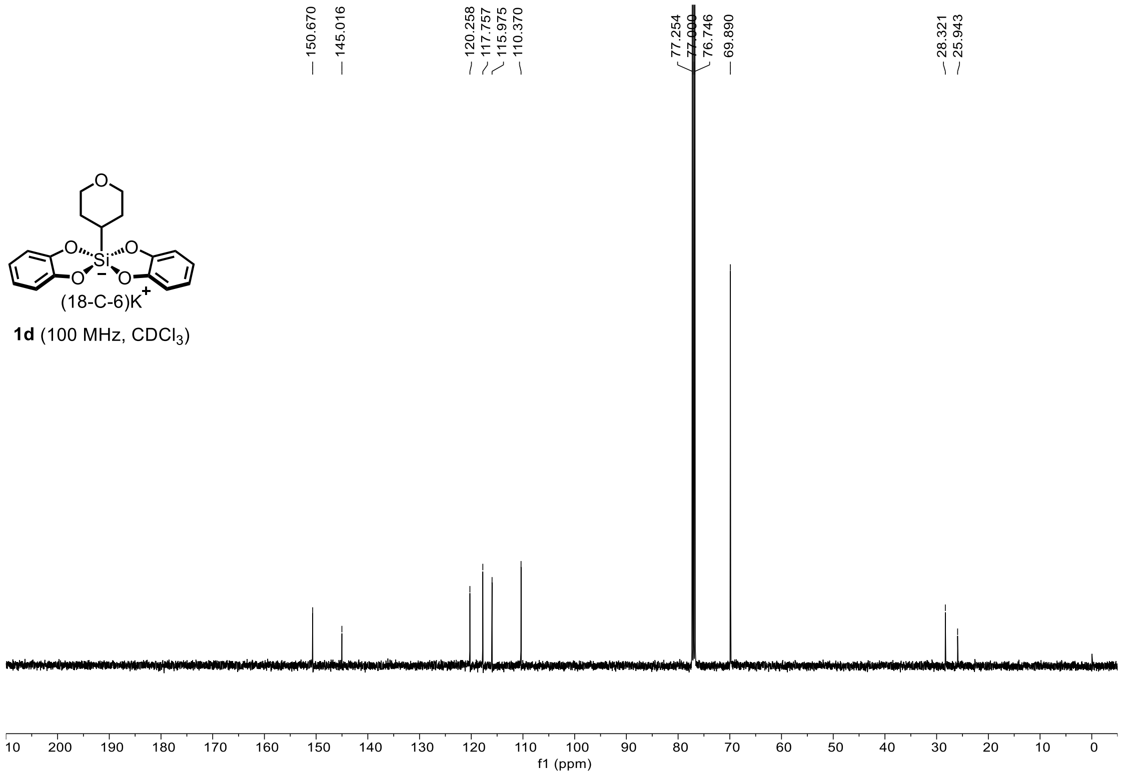

1d (100 MHz, $CDCl_3$)

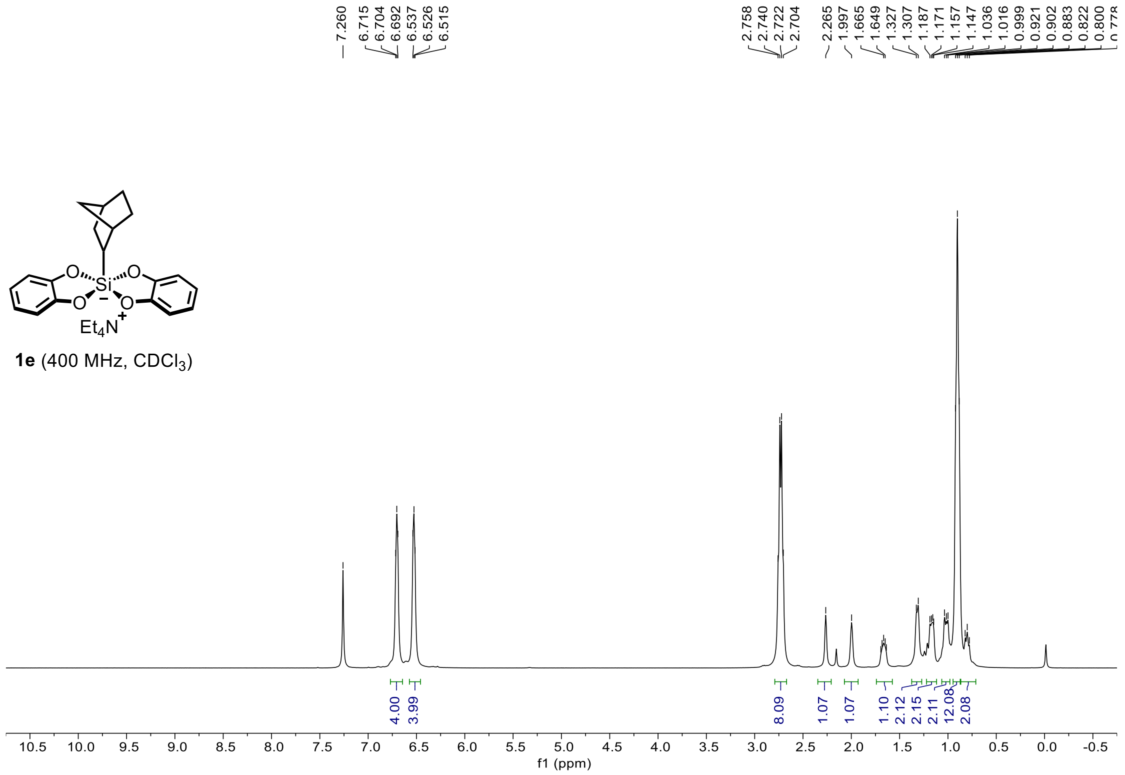

1e (400 MHz, $CDCl_3$)

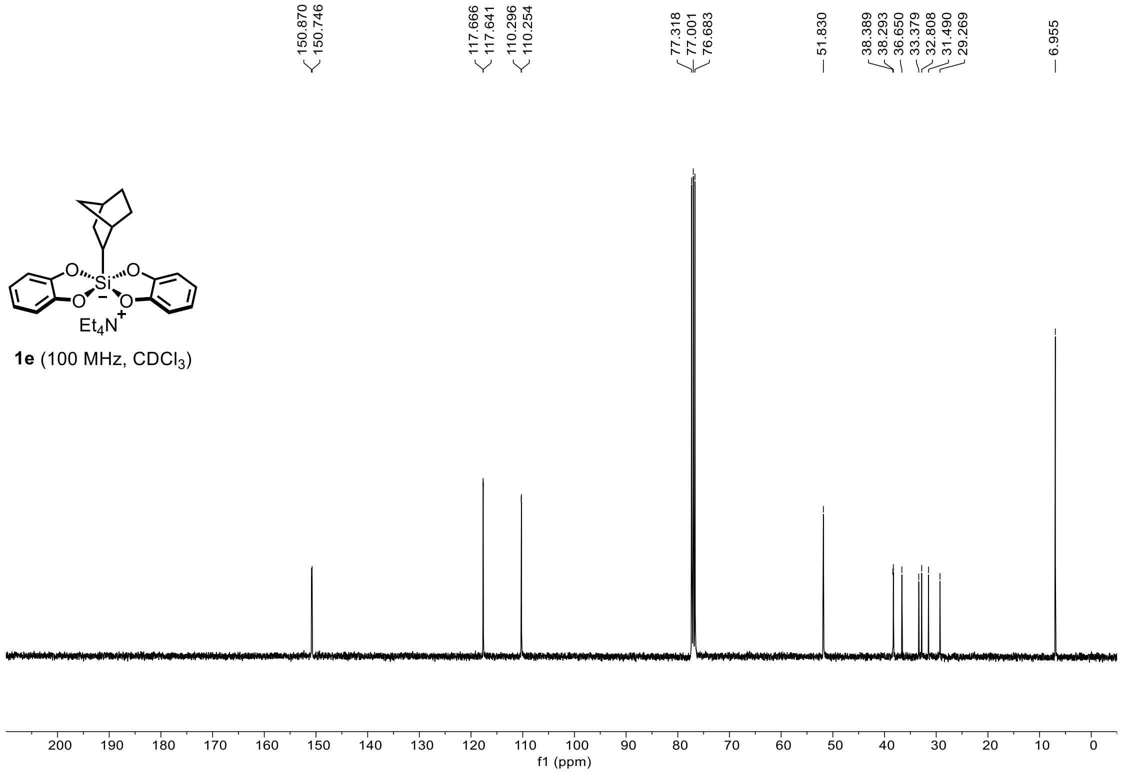

1e (100 MHz, $CDCl_3$)

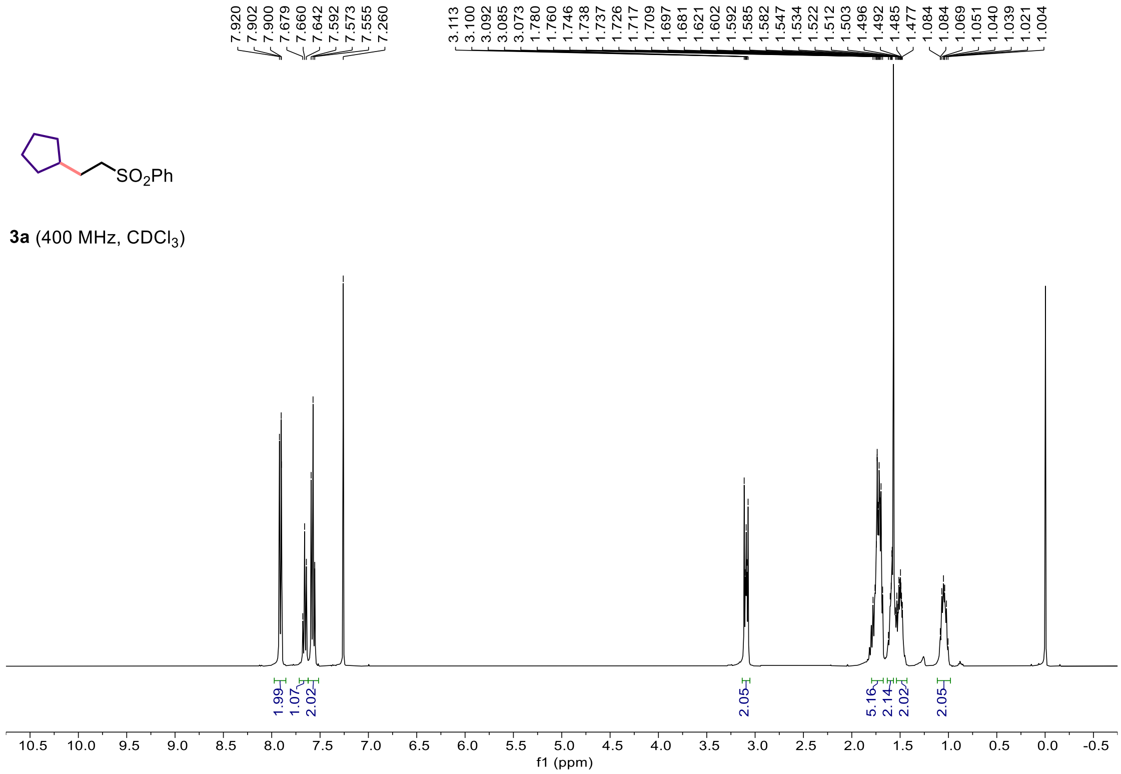

3a (400 MHz, $CDCl_3$)

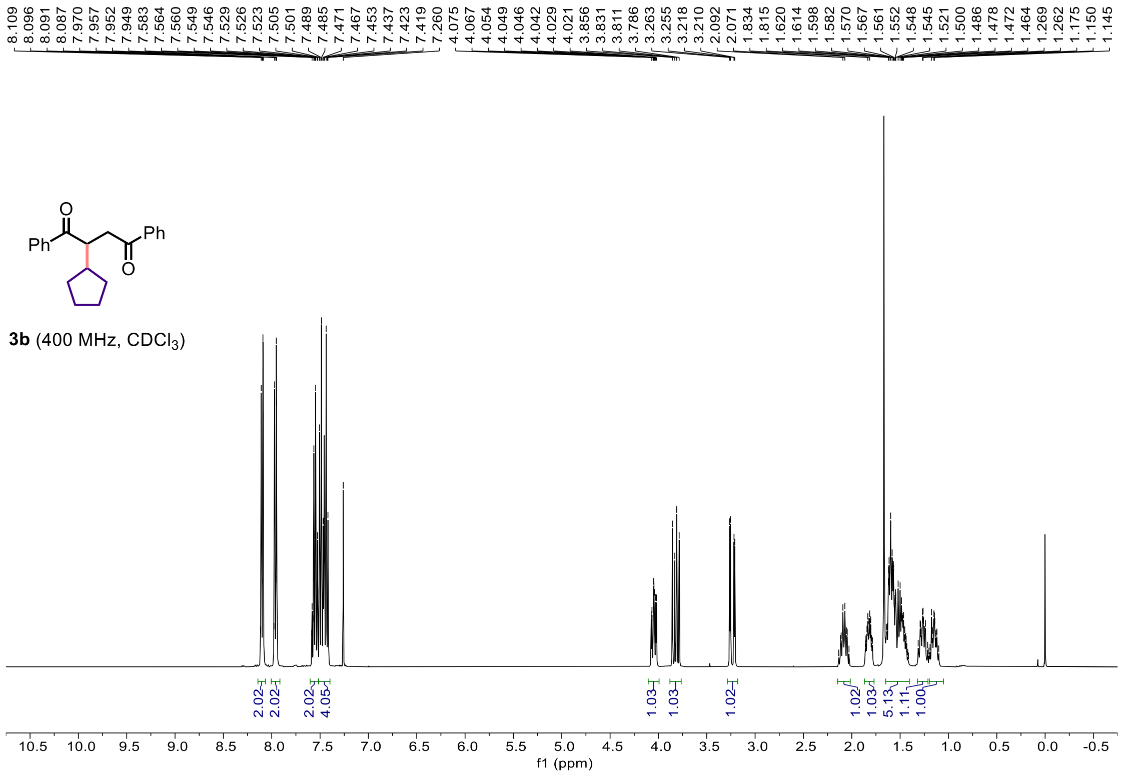

3b (400 MHz, $CDCl_3$)

3b (100 MHz, $CDCl_3$)

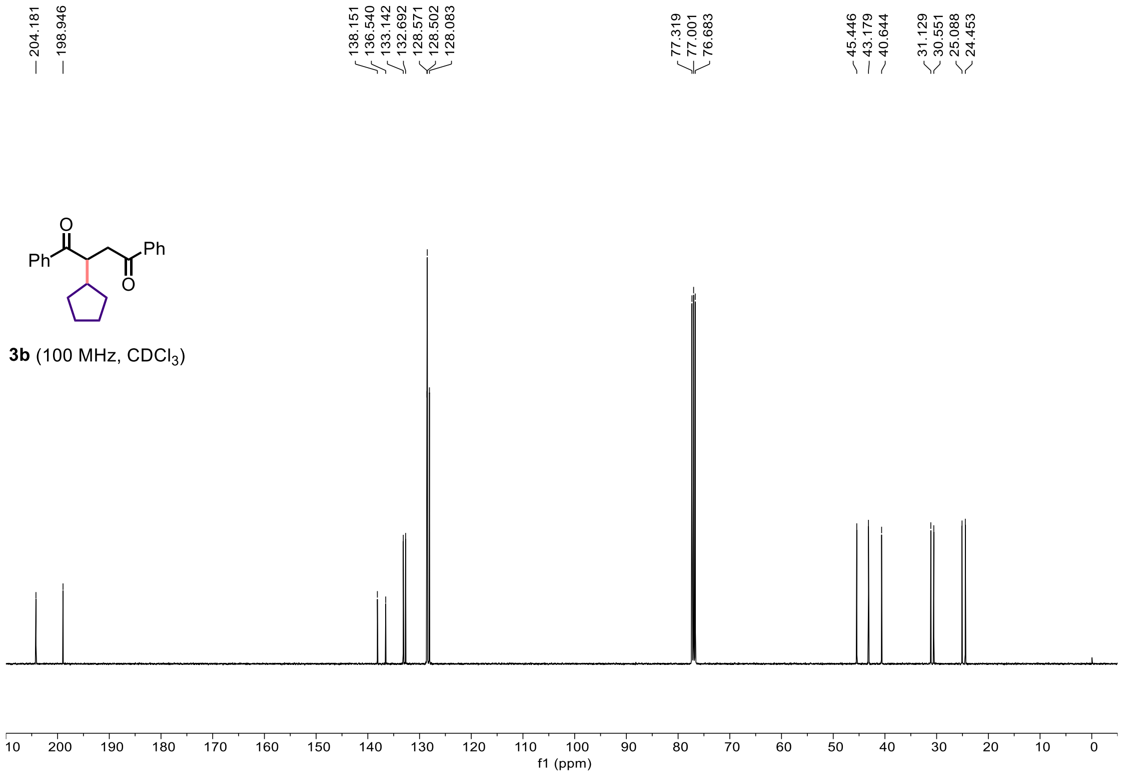

3c (400 MHz, $CDCl_3$)

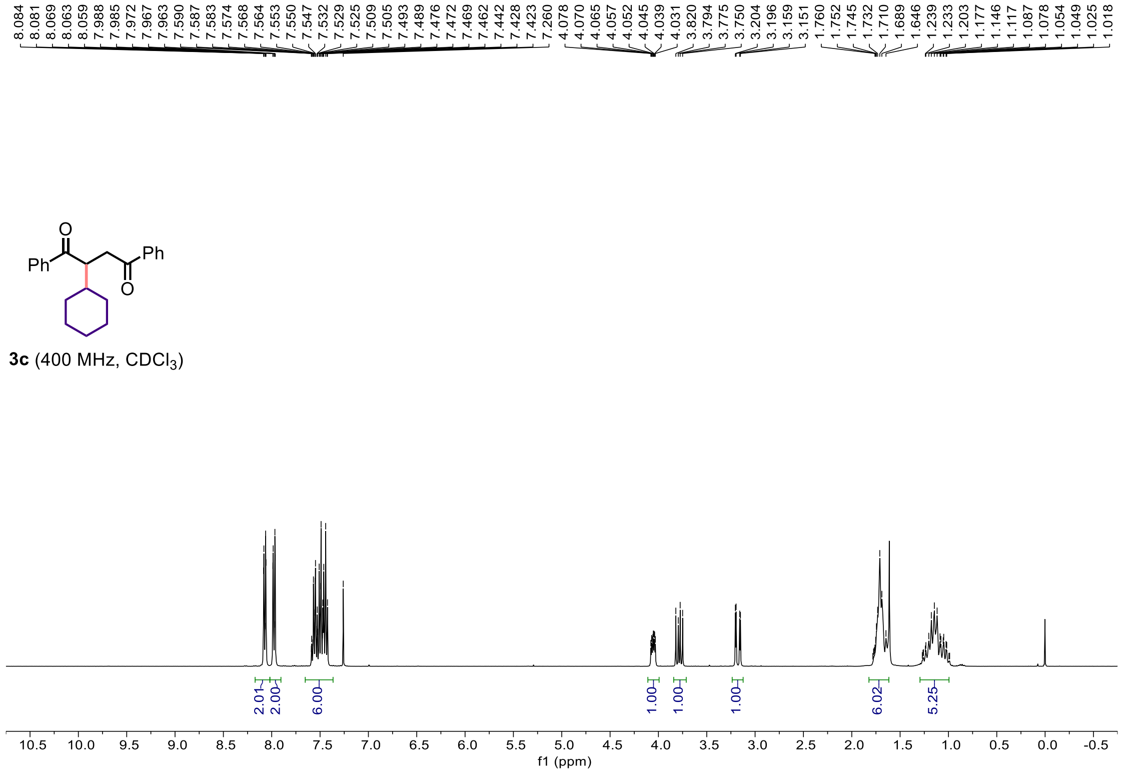

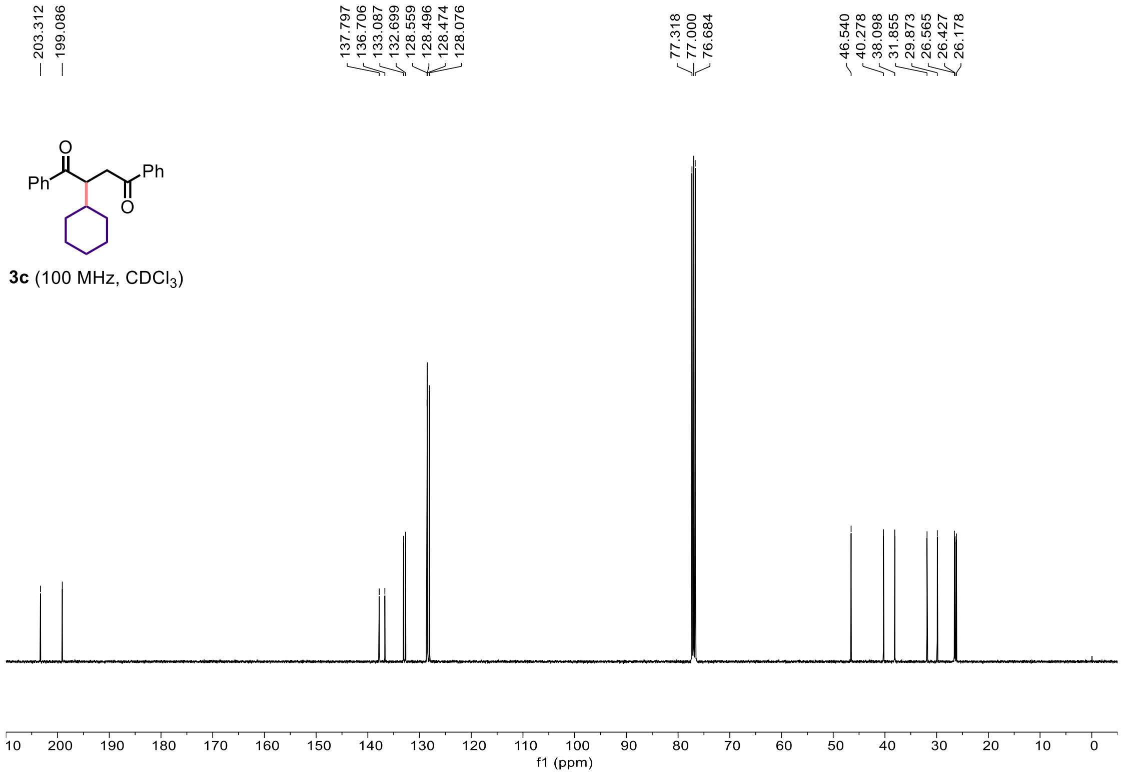
3c (100 MHz, CDCl3)
f1 (ppm)

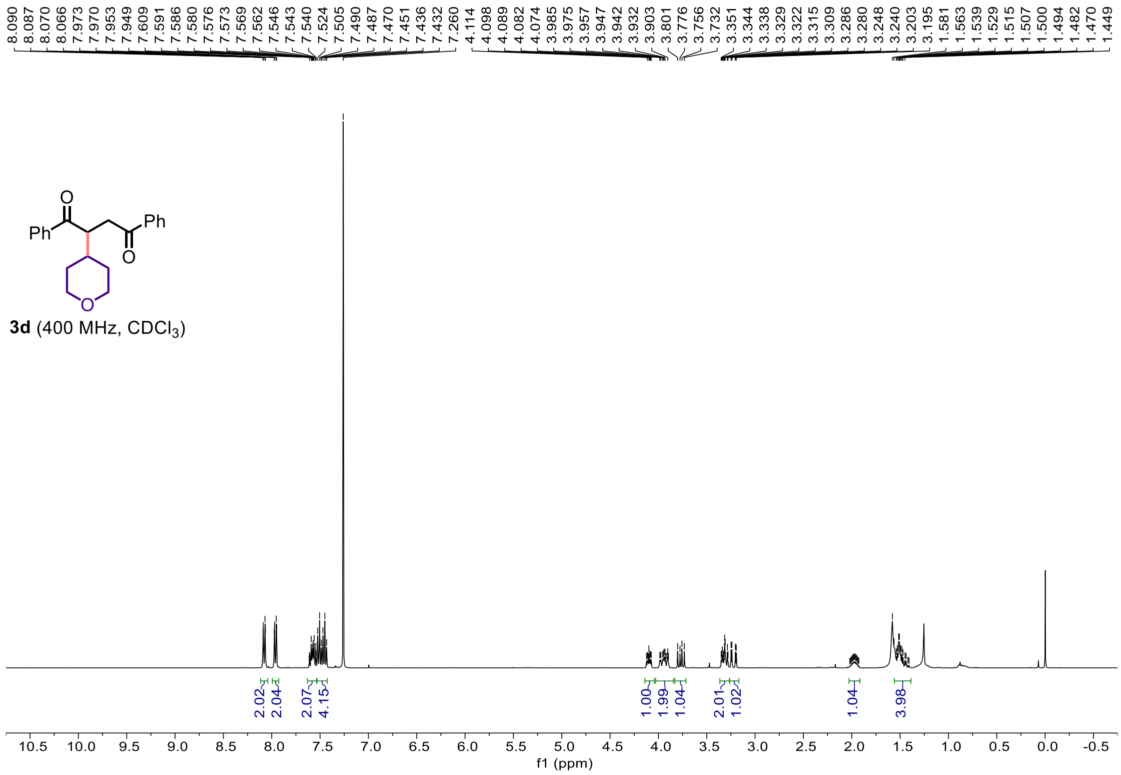
3d (400 MHz, CDCl3)
f1 (ppm)

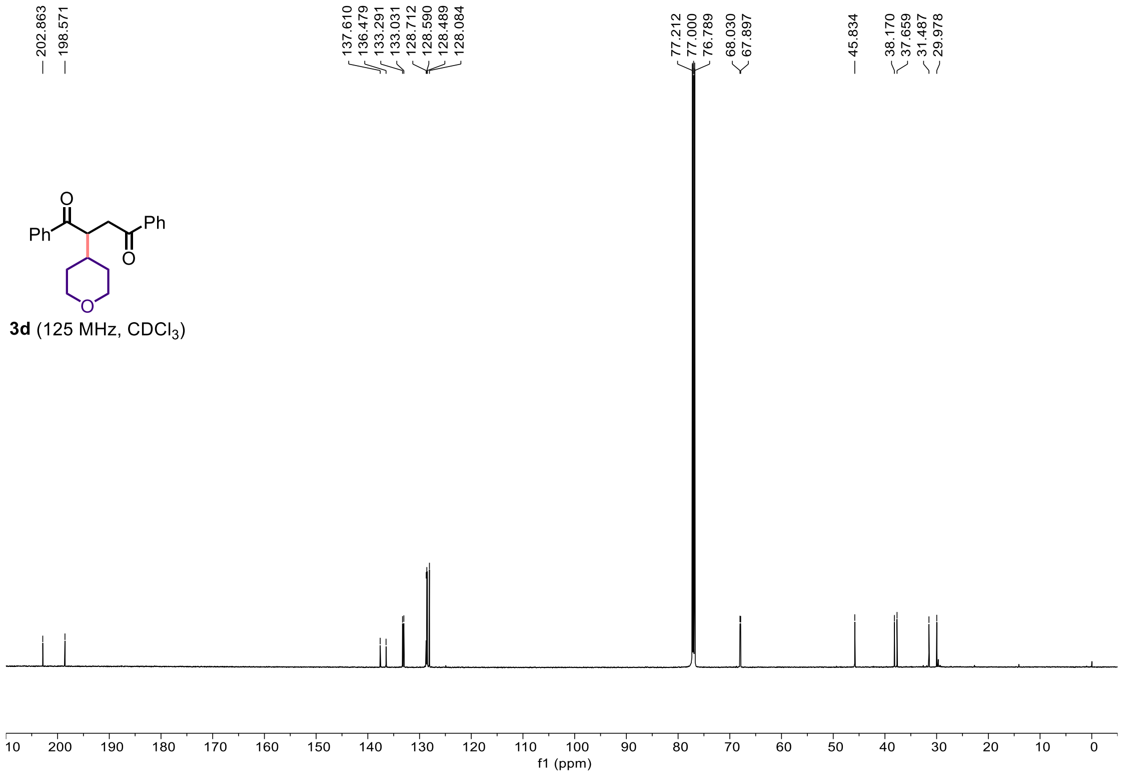

3d (125 MHz, $CDCl_3$)

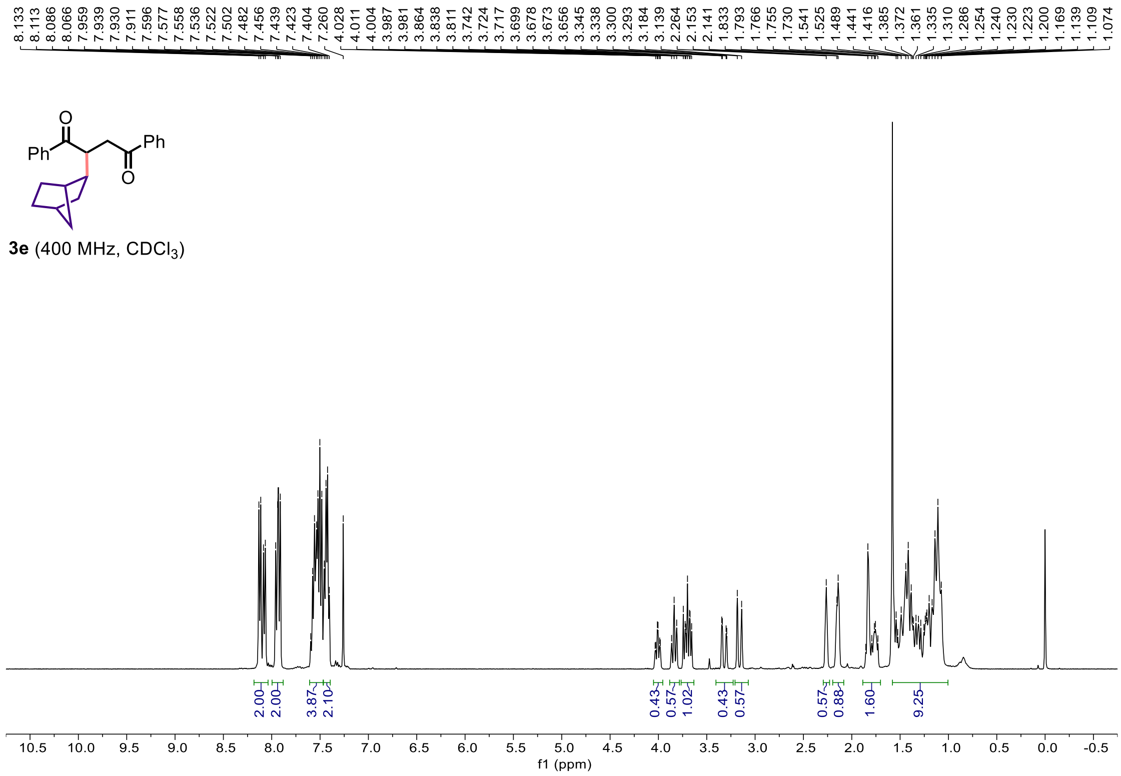

3e (400 MHz, $CDCl_3$)

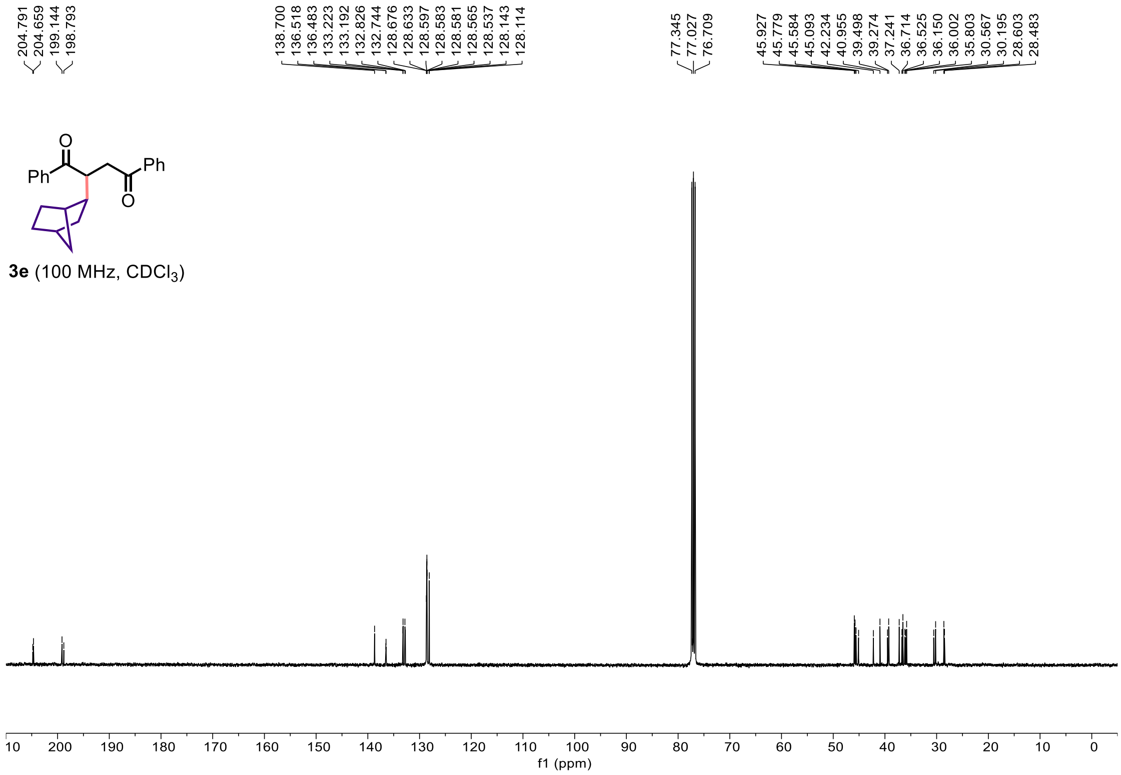

3e (100 MHz, $CDCl_3$)

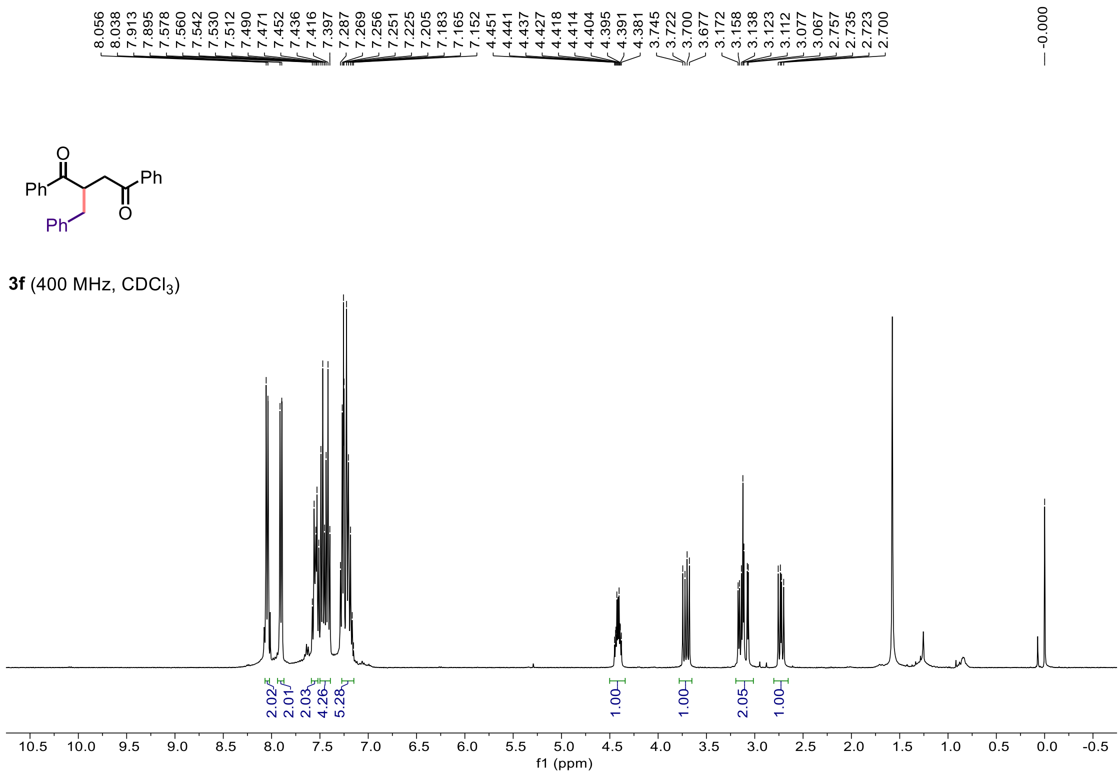

3f (400 MHz, $CDCl_3$)

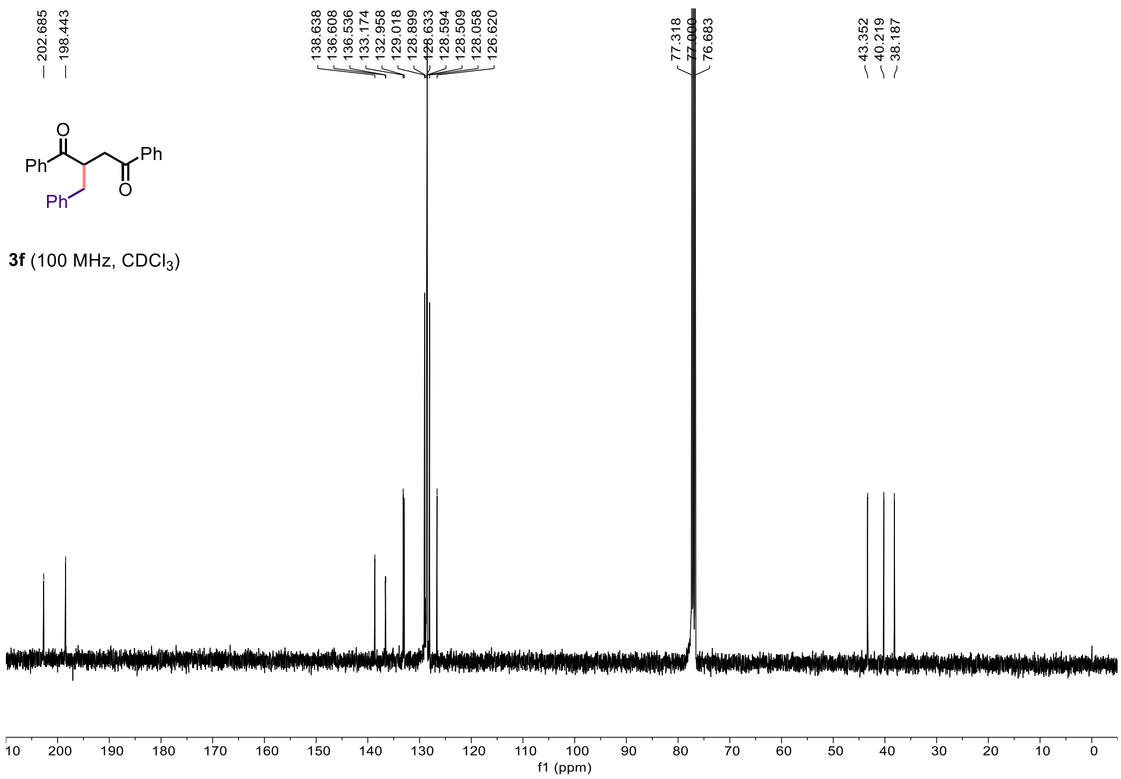

3f (100 MHz, $CDCl_3$)

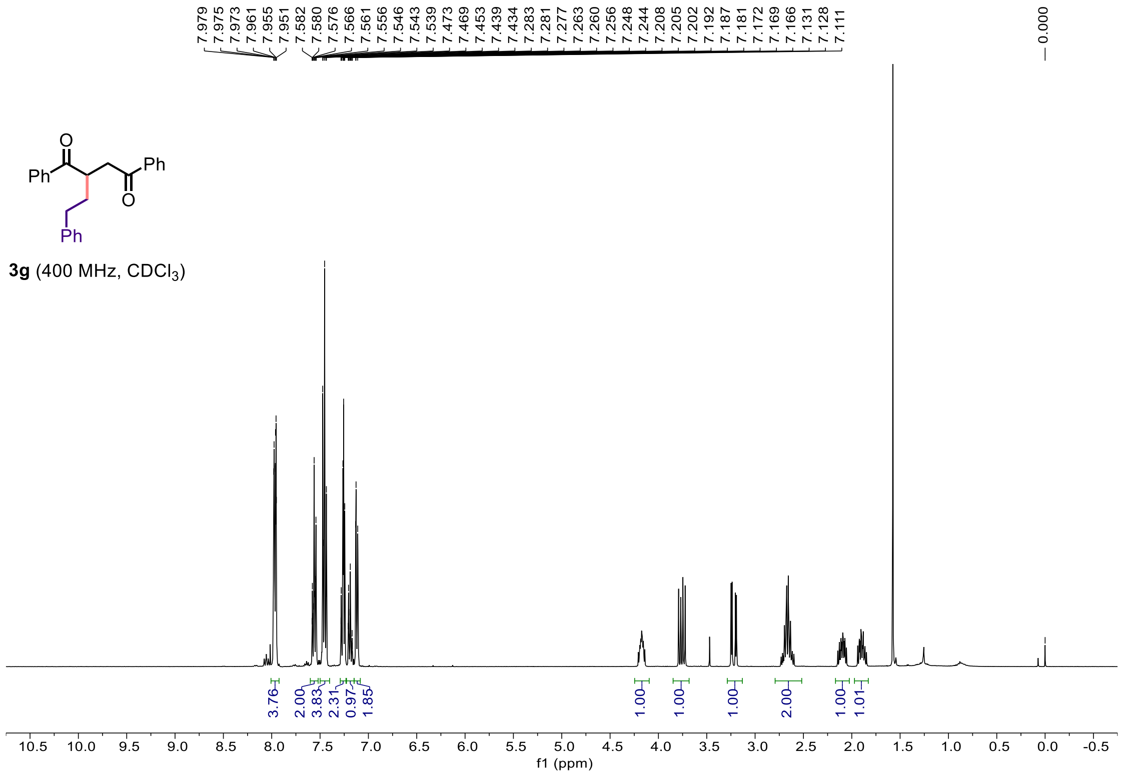

3g (400 MHz, $CDCl_3$)

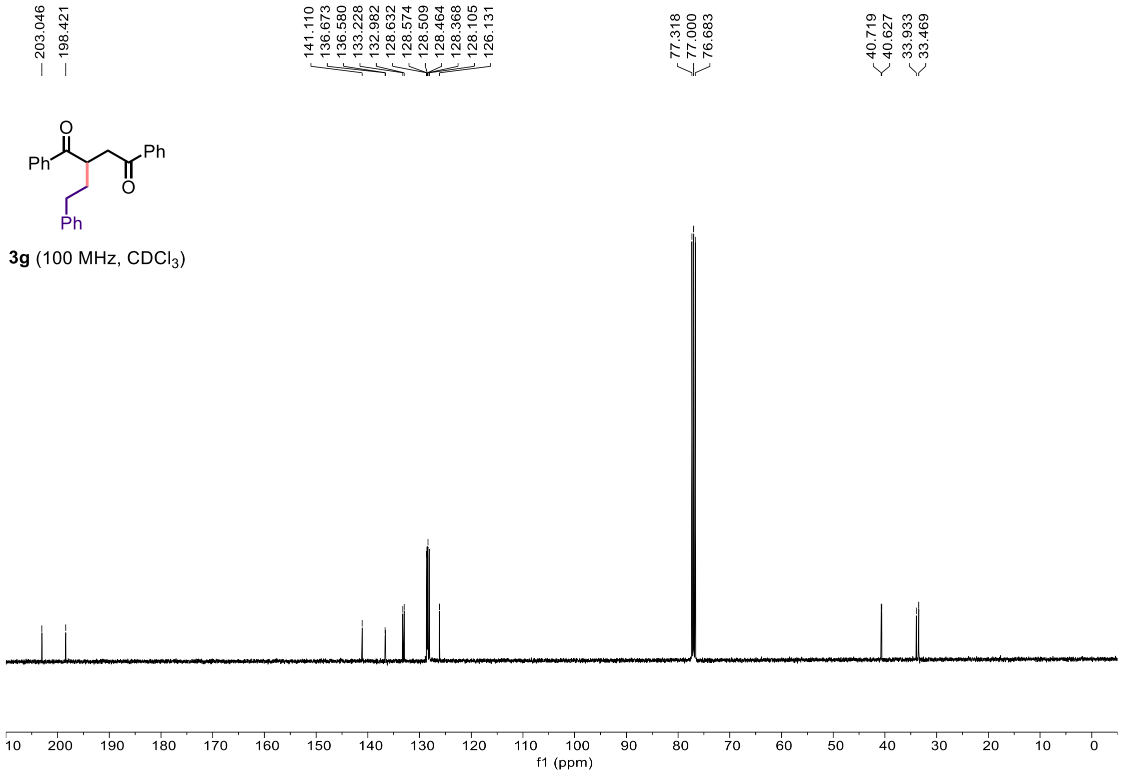

3g (100 MHz, $CDCl_3$)

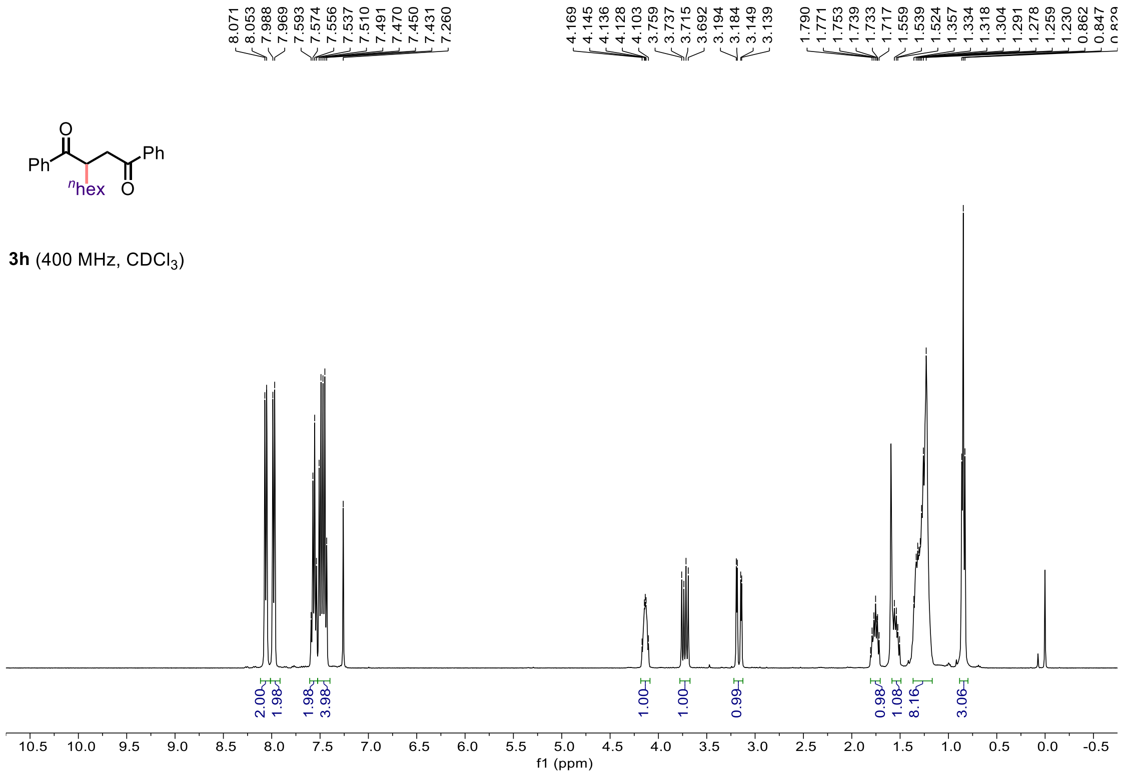

3h (400 MHz, $CDCl_3$)

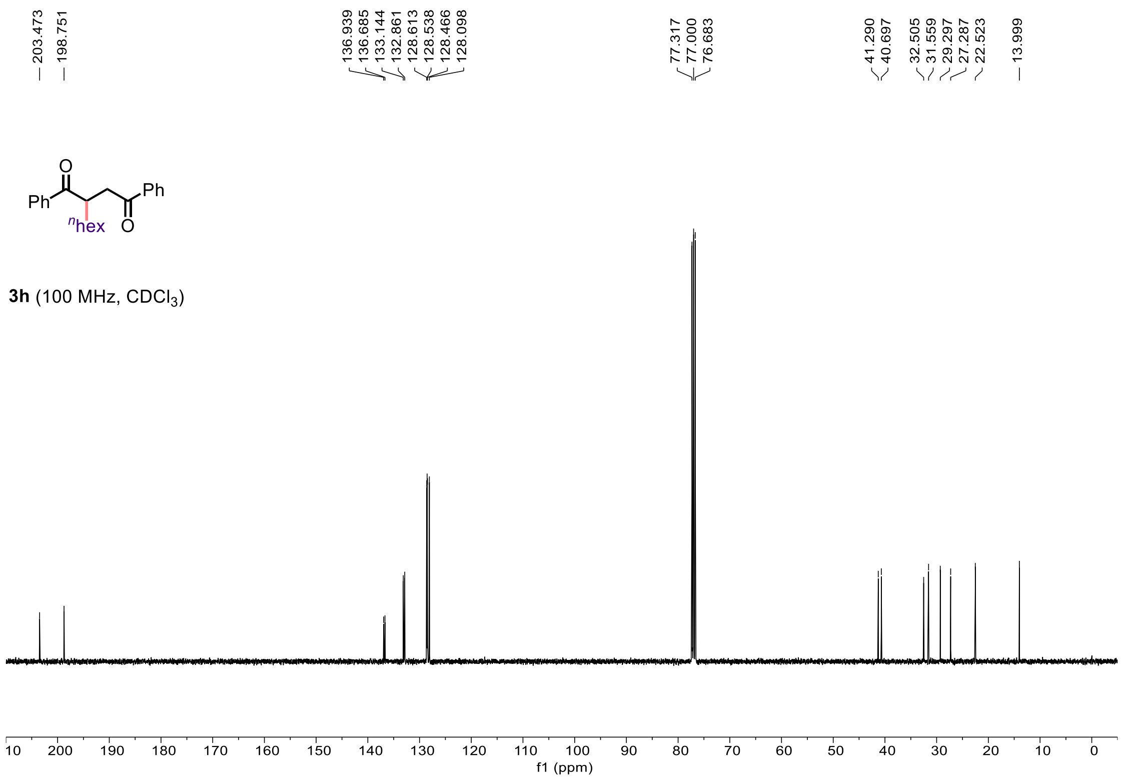
203.473
198.751
136.939
136.685
133.144
132.861
128.613
128.538
128.466
128.098
77.317
77.000
76.683
41.290
40.697
32.505
31.559
29.297
27.287
22.523
13.999
Ph
O
Ph
nhex
O
3h (100 MHz, CDCl3)
f1 (ppm)

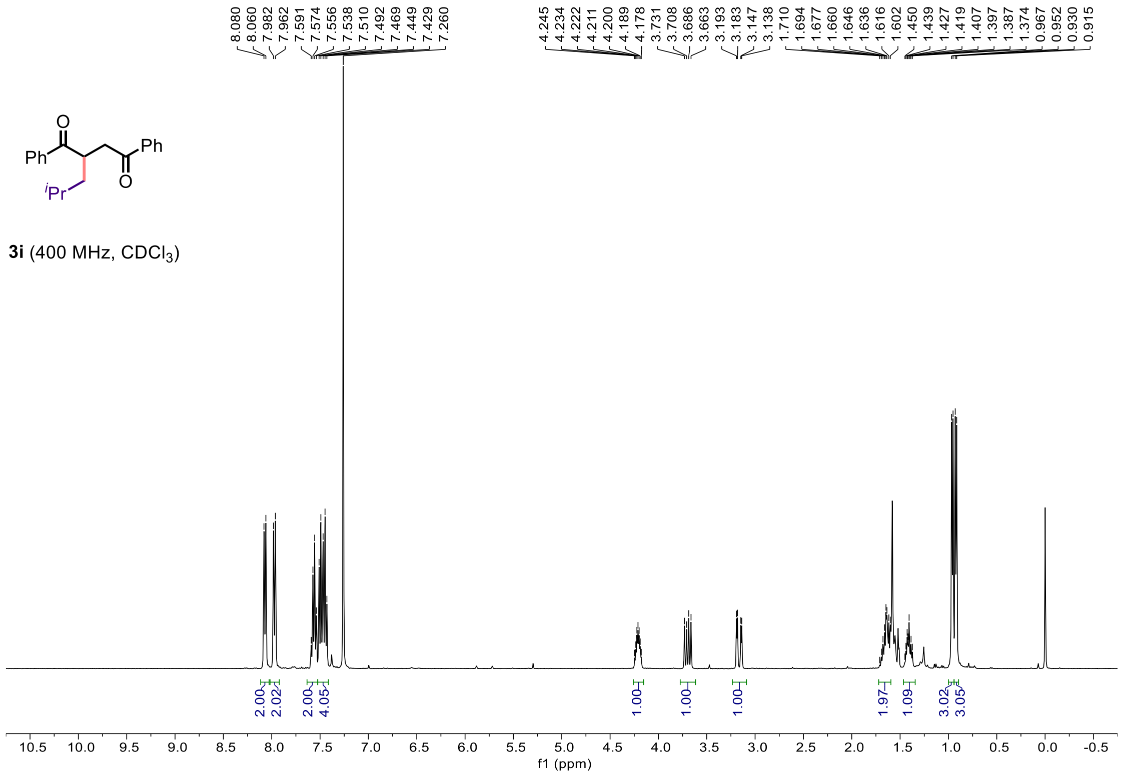
8.080
8.060
7.982
7.962
7.591
7.574
7.556
7.538
7.510
7.492
7.469
7.449
7.429
7.260
4.245
4.234
4.222
4.211
4.200
4.189
4.178
3.731
3.708
3.686
3.663
3.193
3.183
3.147
3.138
1.710
1.694
1.677
1.660
1.646
1.636
1.616
1.602
1.450
1.439
1.427
1.419
1.407
1.397
1.387
1.374
0.967
0.952
0.930
0.915
Ph
O
Ph
iPr
O
3i (400 MHz, CDCl3)
2.00
2.02
2.00
4.05
1.00
1.00
1.00
1.97
1.09
3.02
3.05
f1 (ppm)

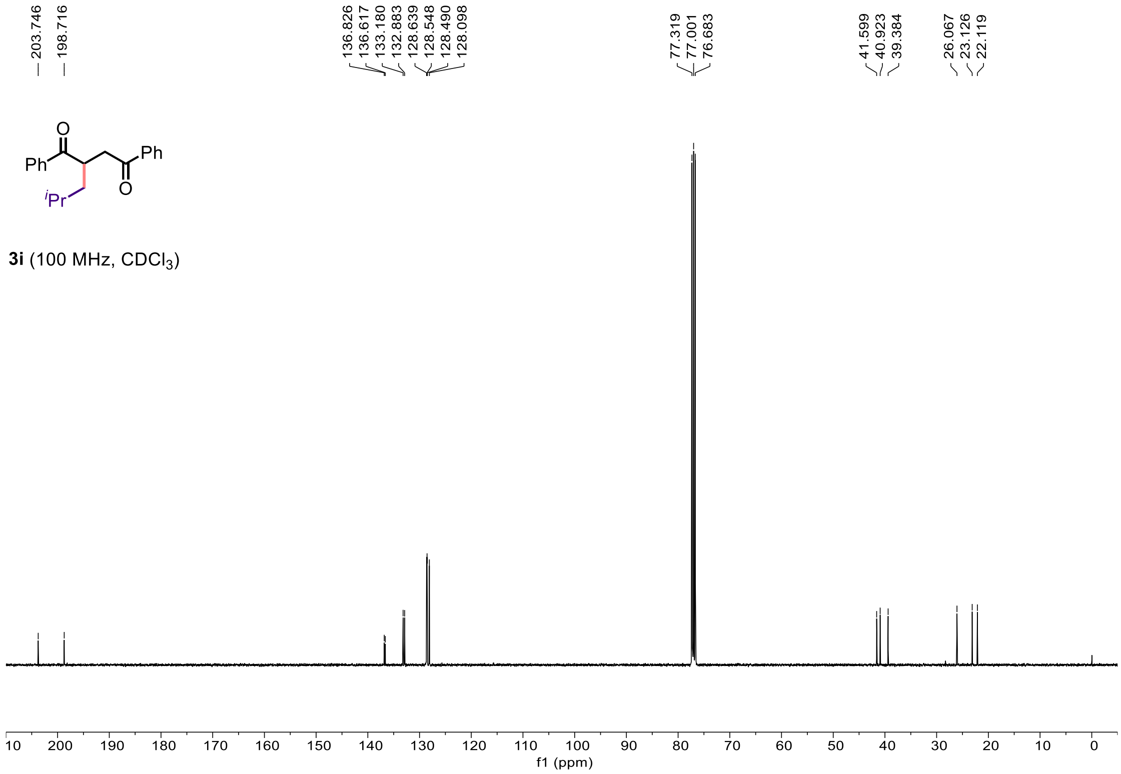

3i (100 MHz, $CDCl_3$)

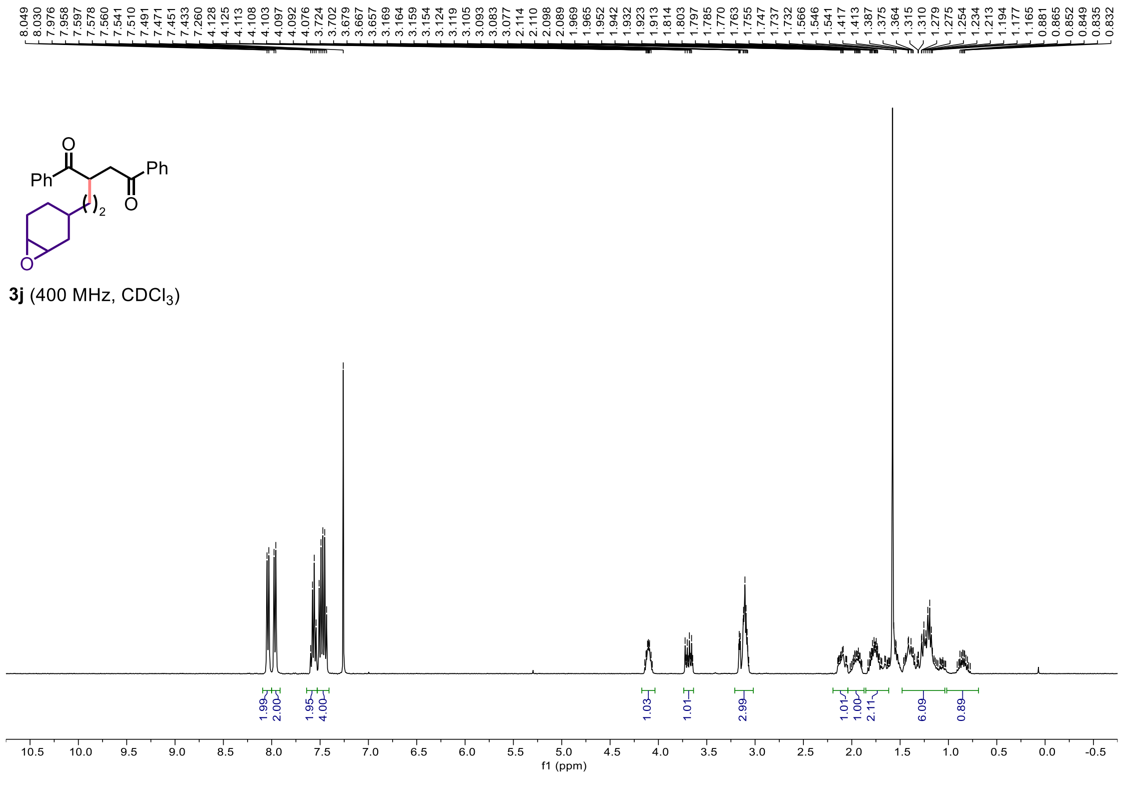

3j (400 MHz, $CDCl_3$)

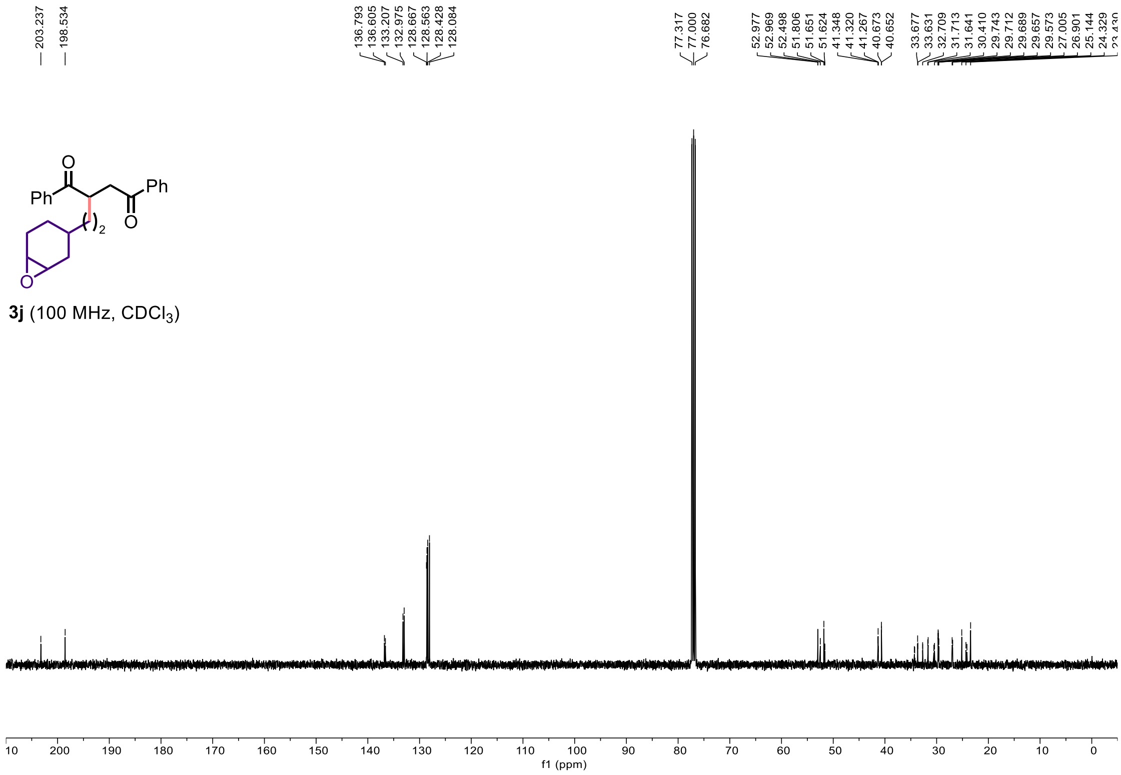

3j (100 MHz, $CDCl_3$)

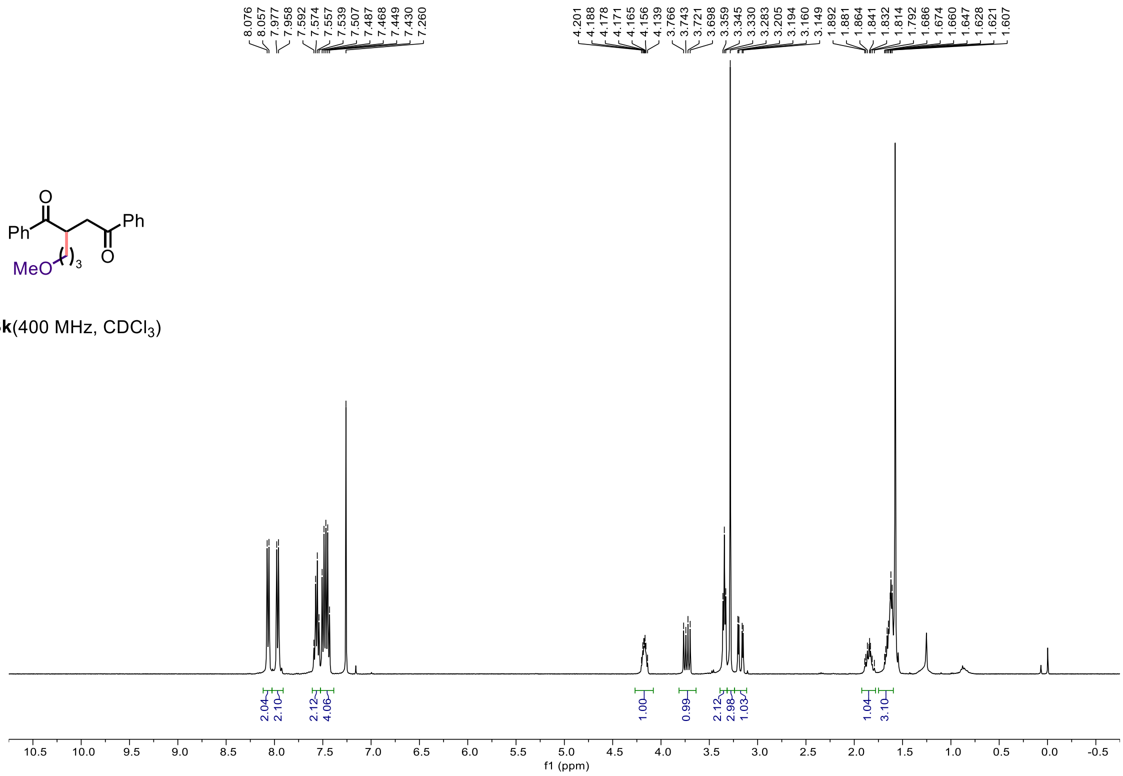

3k(400 MHz, $CDCl_3$)

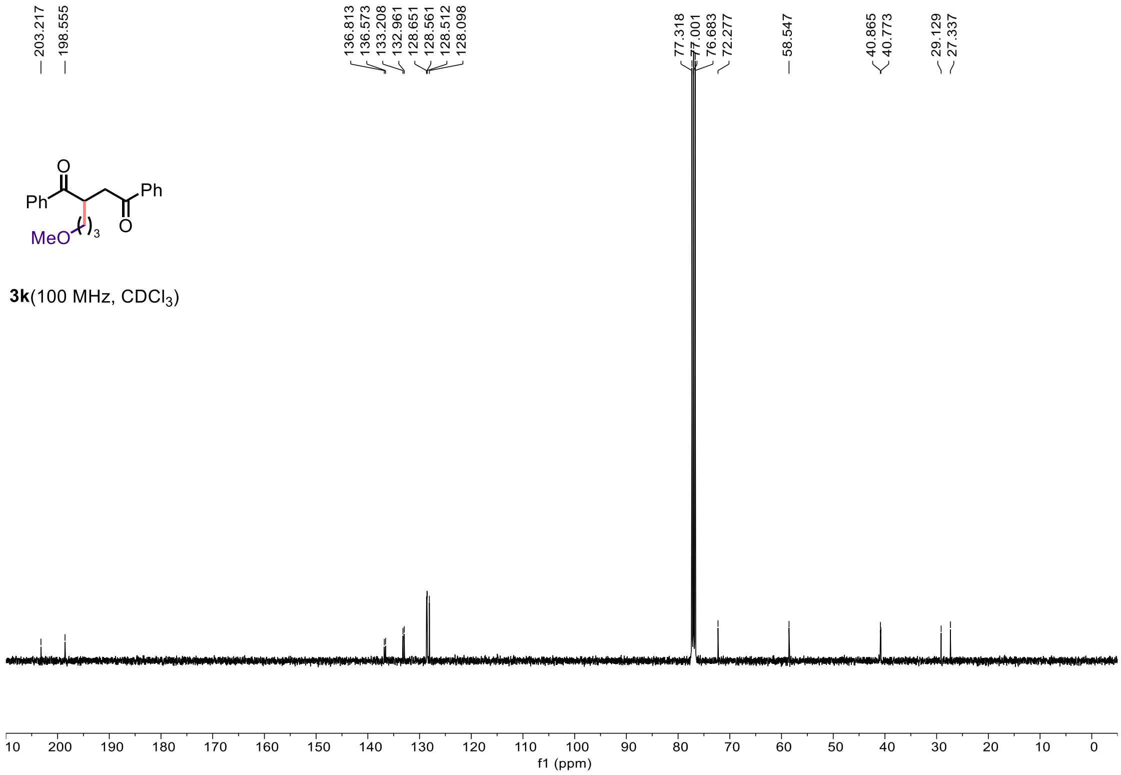

3k(100 MHz, $CDCl_3$)

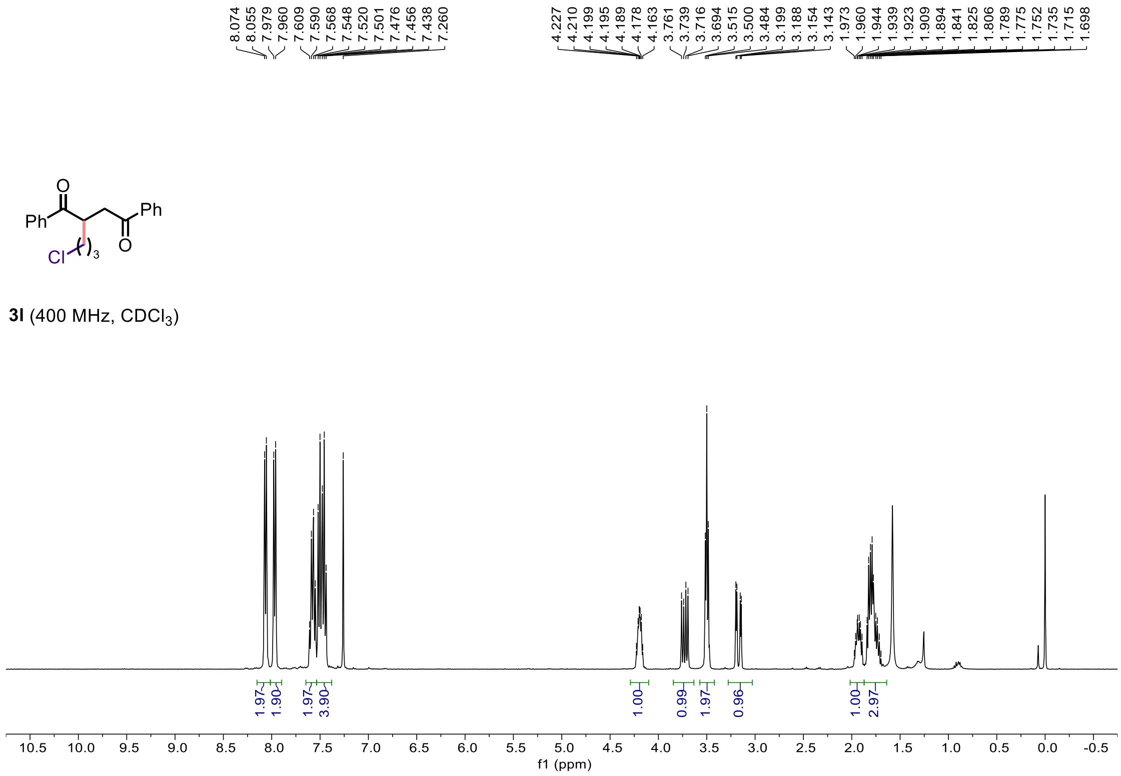

3l (400 MHz, $CDCl_3$)

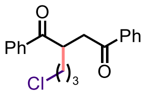

3l (100 MHz, $CDCl_3$)

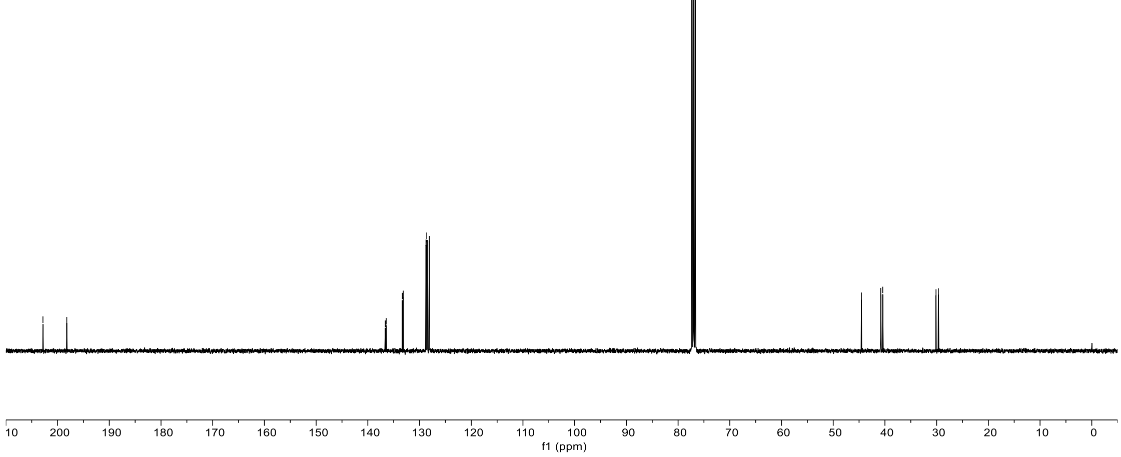

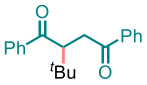

3m (400 MHz, $CDCl_3$)

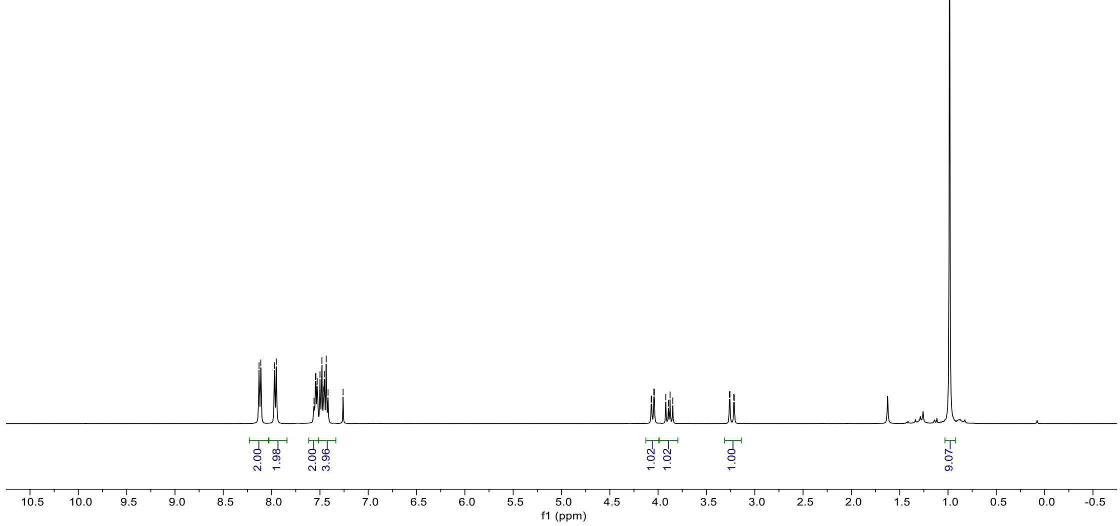

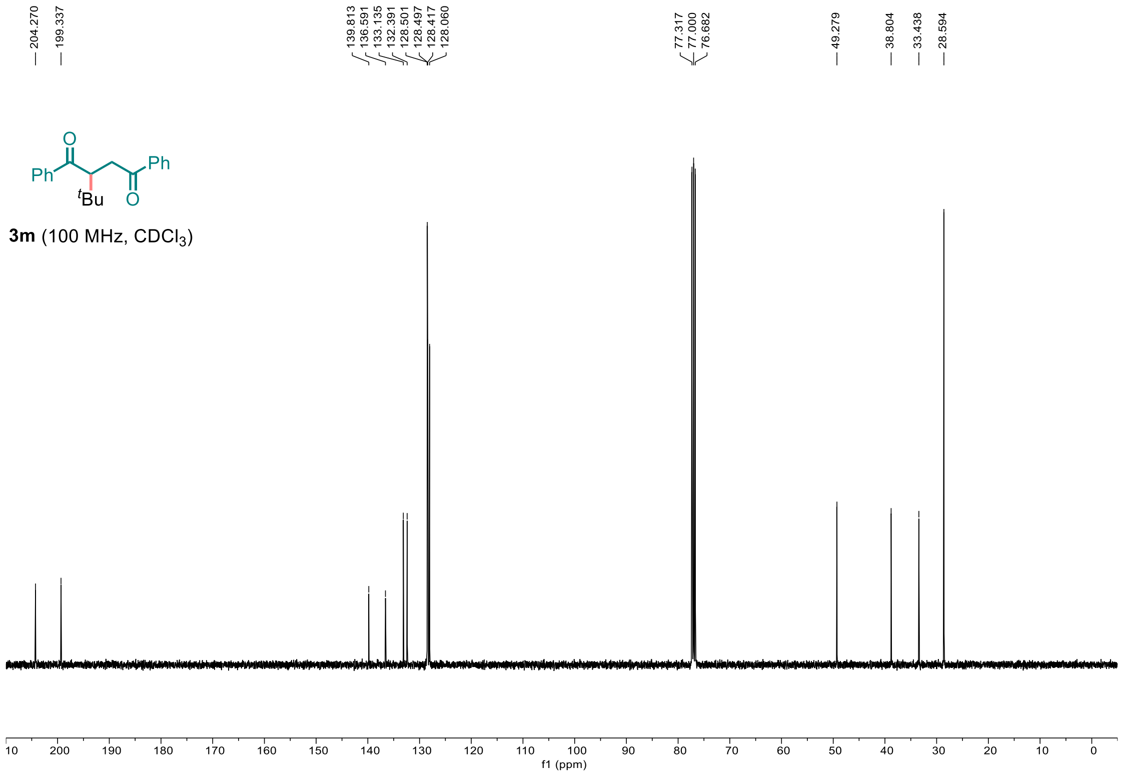

3m (100 MHz, $CDCl_3$)

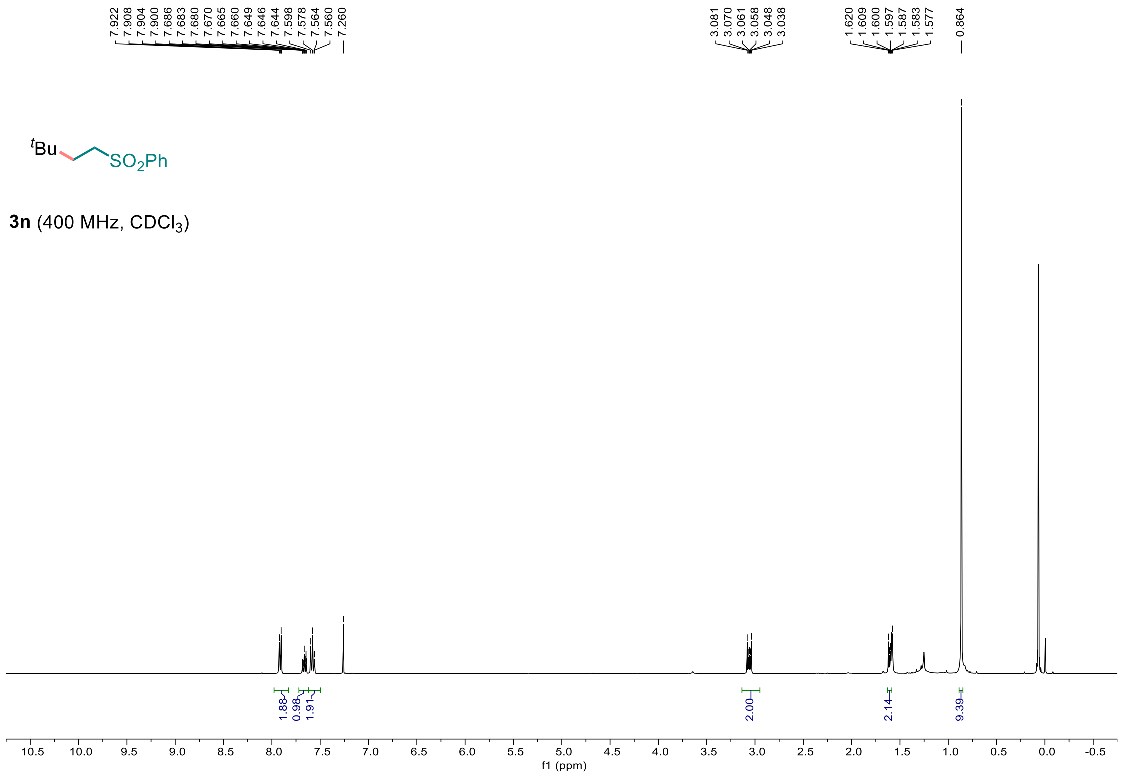

3n (400 MHz, $CDCl_3$)

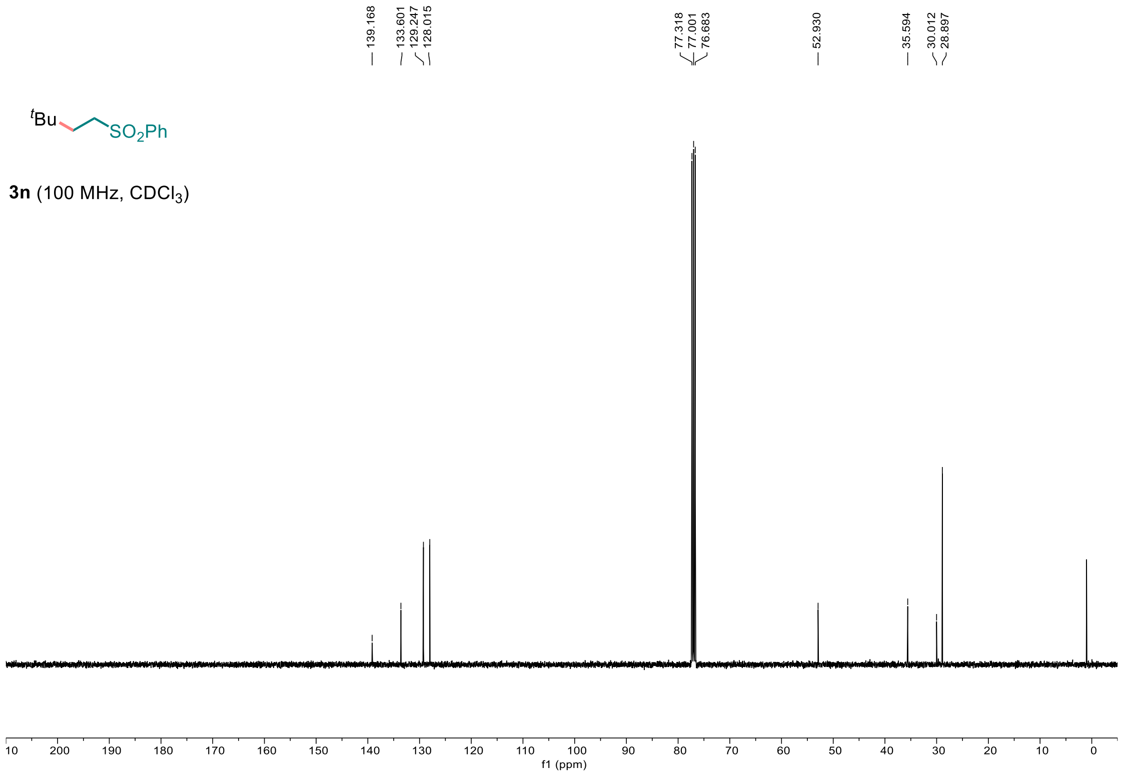

139.168
133.601
129.247
128.015
77.318
77.001
76.683
52.930
35.594
30.012
28.897
^{t}Bu
SO_2Ph
3n (100 MHz, $CDCl_3$)
f1 (ppm)

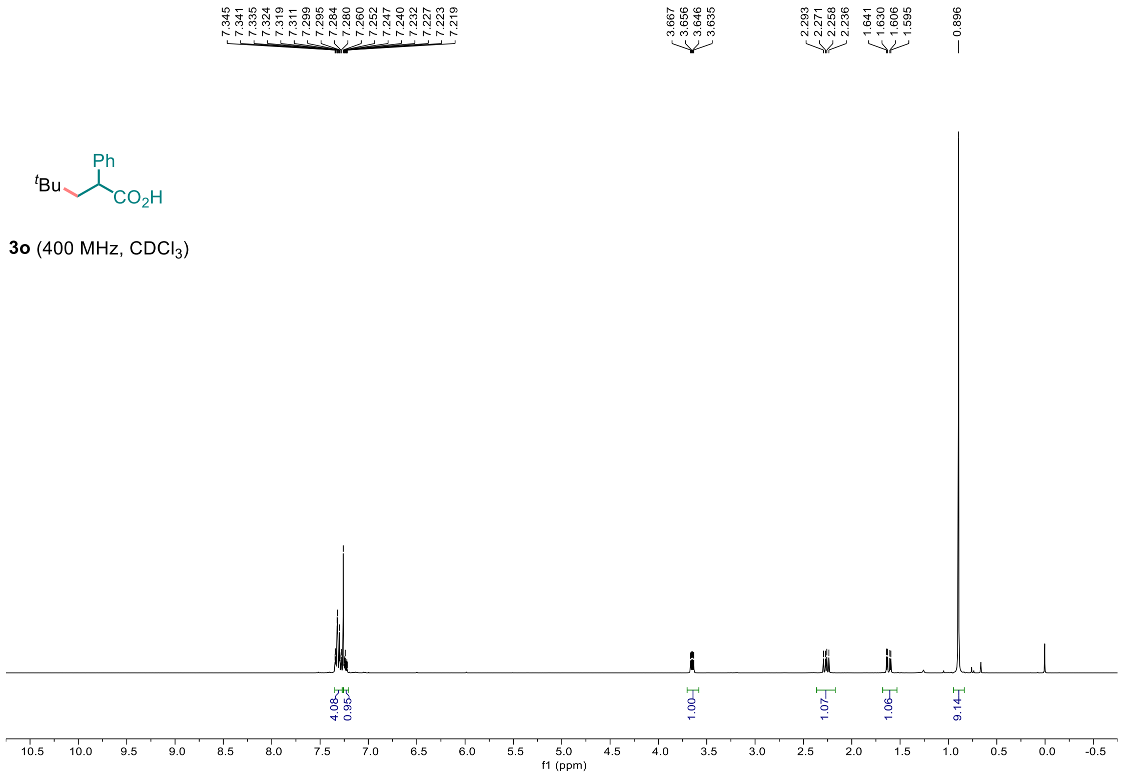

7.345
7.341
7.335
7.324
7.319
7.311
7.299
7.295
7.284
7.280
7.260
7.252
7.247
7.240
7.232
7.227
7.223
7.219
3.667
3.656
3.646
3.635
2.293
2.271
2.258
2.236
1.641
1.630
1.606
1.595
0.896
Ph
^{t}Bu
CO_2H
3o (400 MHz, $CDCl_3$)
4.08
0.95
1.00
1.07
1.06
9.14
f1 (ppm)

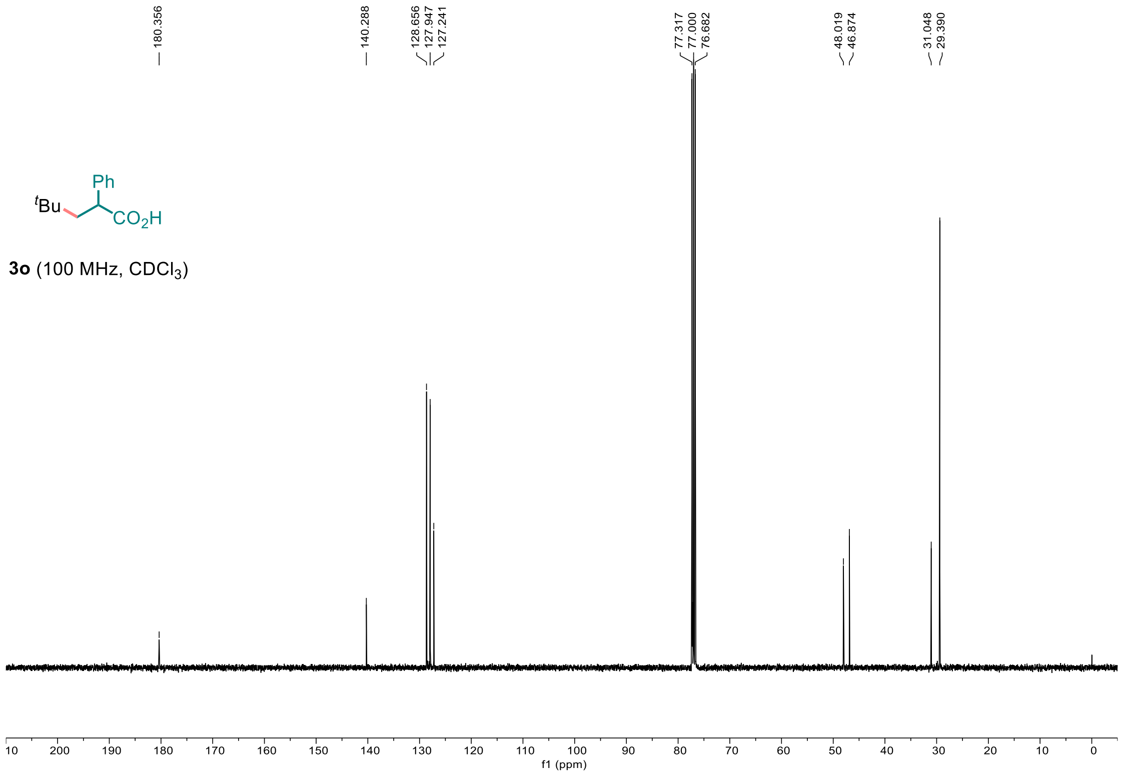
180.356
140.288
128.656
127.947
127.241
77.317
77.000
76.682
48.019
46.874
31.048
29.390
Ph
tBu
CO2H
3o (100 MHz, CDCl3)
f1 (ppm)

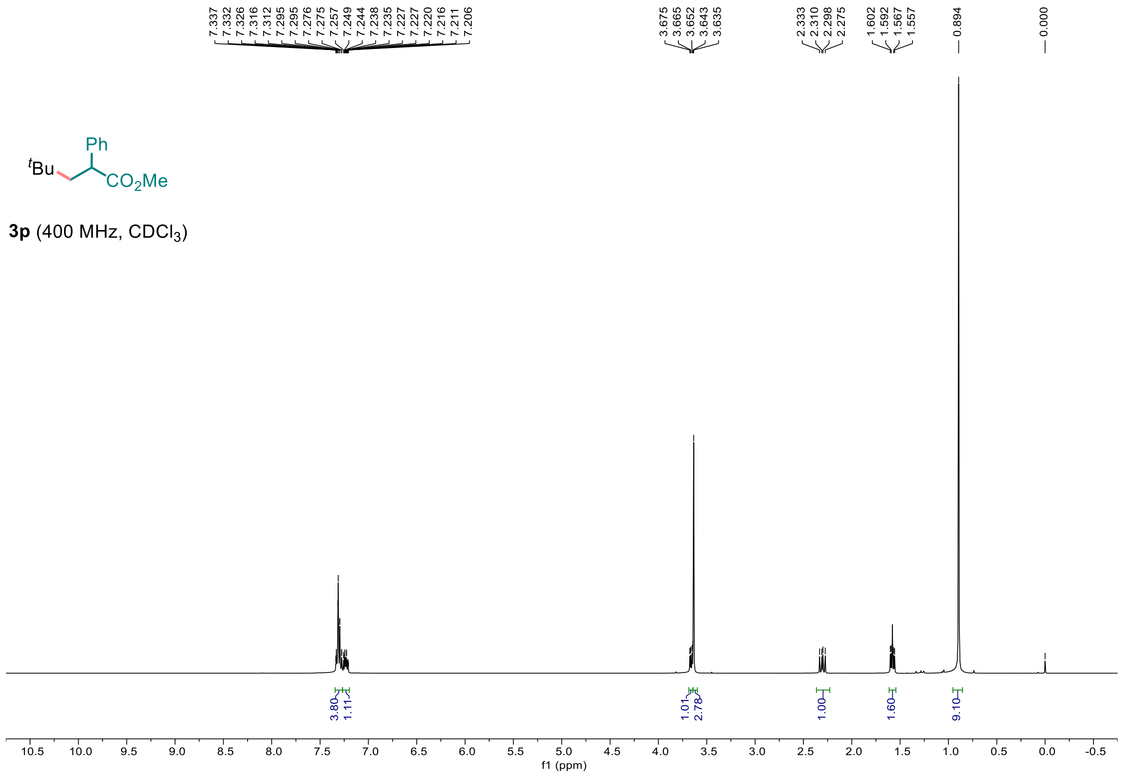
7.337
7.332
7.326
7.316
7.312
7.295
7.295
7.276
7.275
7.257
7.249
7.244
7.238
7.235
7.227
7.227
7.220
7.216
7.211
7.206
3.675
3.665
3.652
3.643
3.635
2.333
2.310
2.298
2.275
1.602
1.592
1.567
1.557
0.894
0.000
Ph
tBu
CO2Me
3p (400 MHz, CDCl3)
3.80
1.11
1.01
2.78
1.00
1.60
9.10
f1 (ppm)

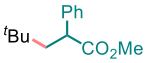

3p (100 MHz, $CDCl_3$)

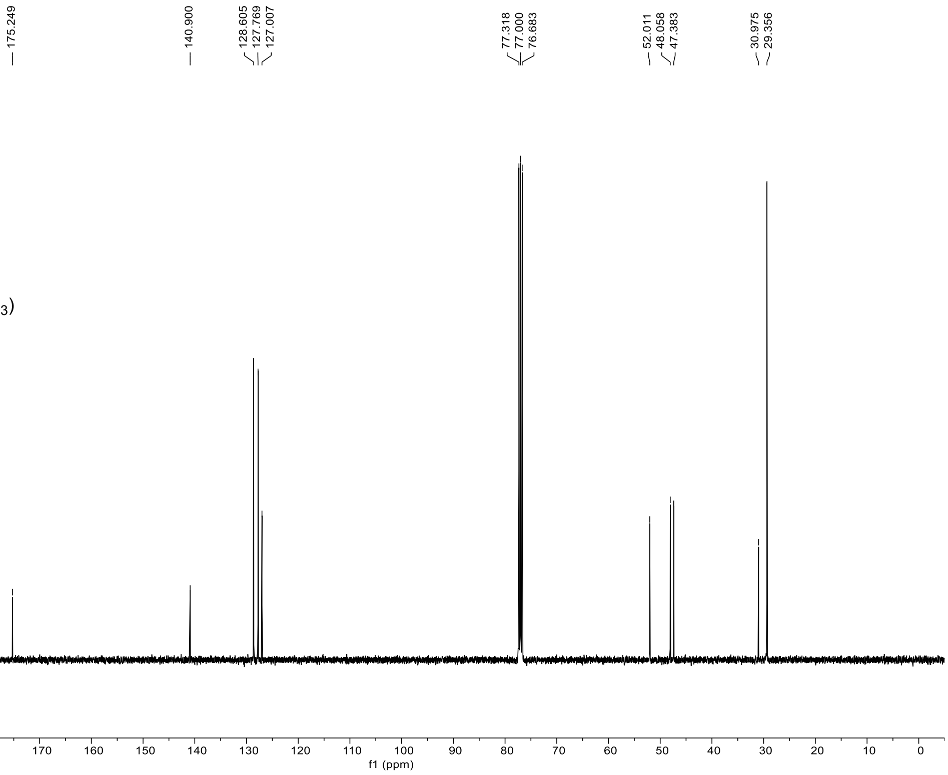

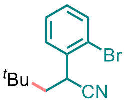

3q (400 MHz, $CDCl_3$)

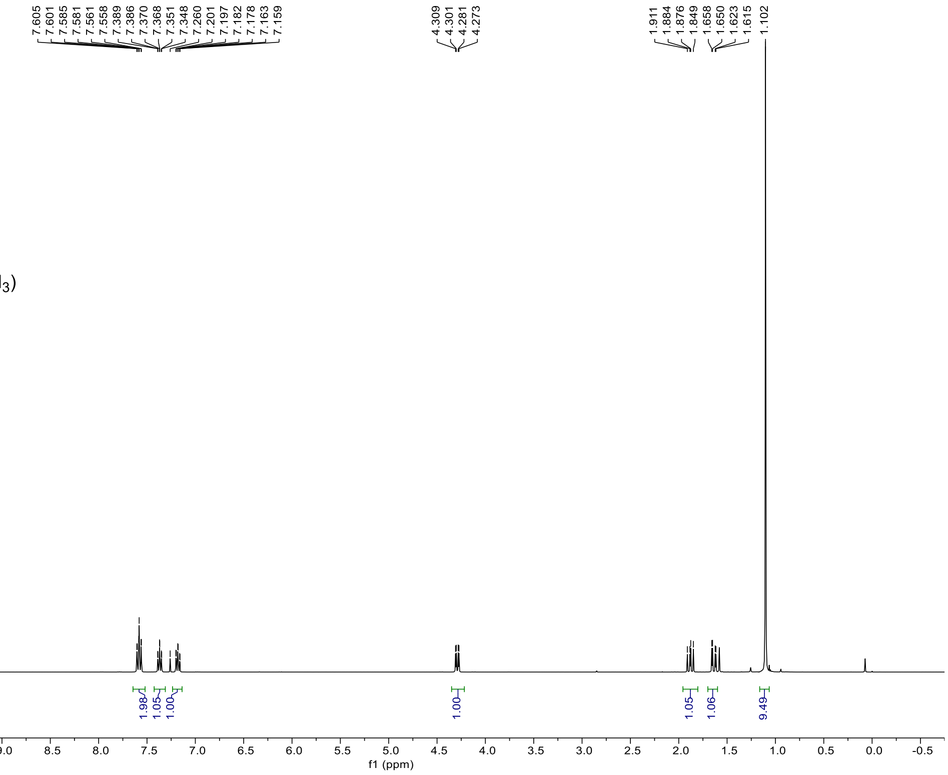

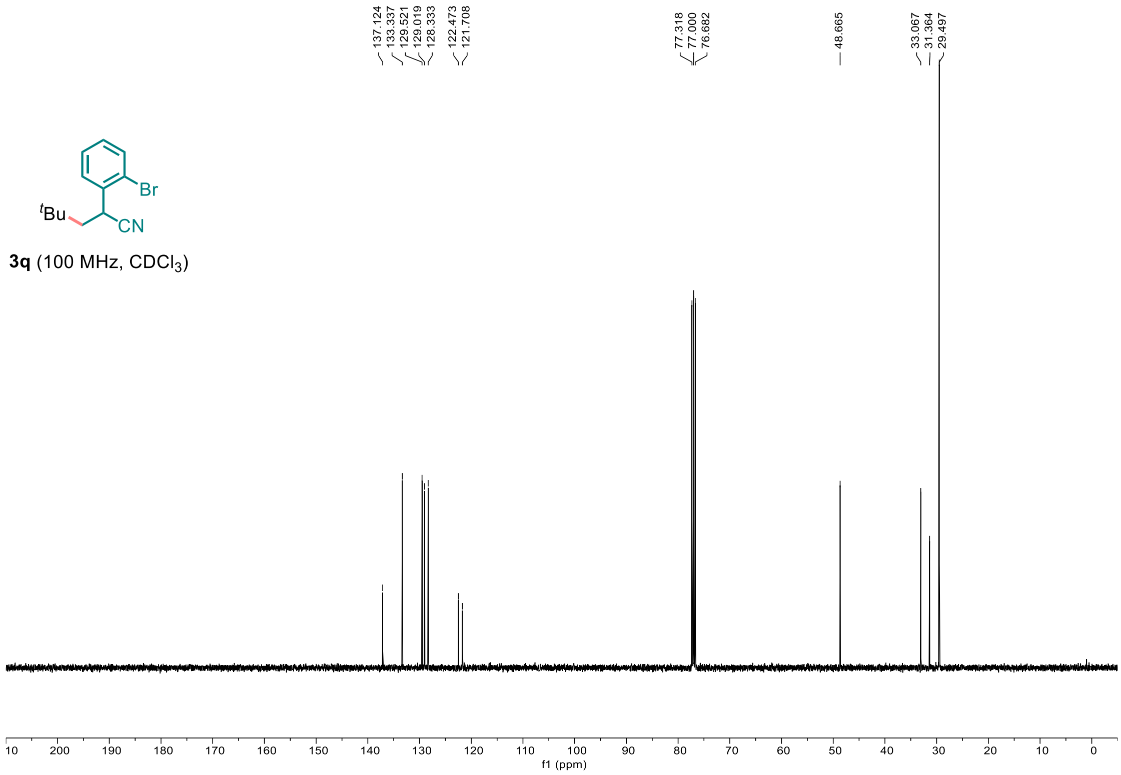

3q (100 MHz, $CDCl_3$)

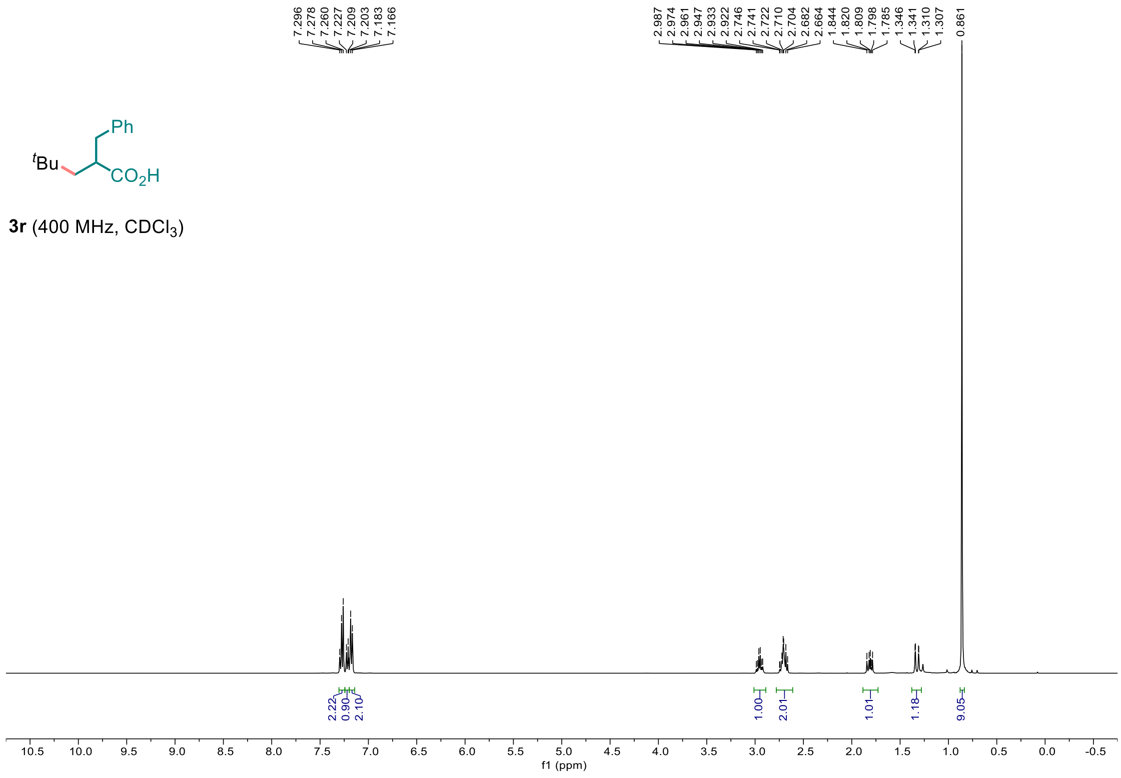

3r (400 MHz, $CDCl_3$)

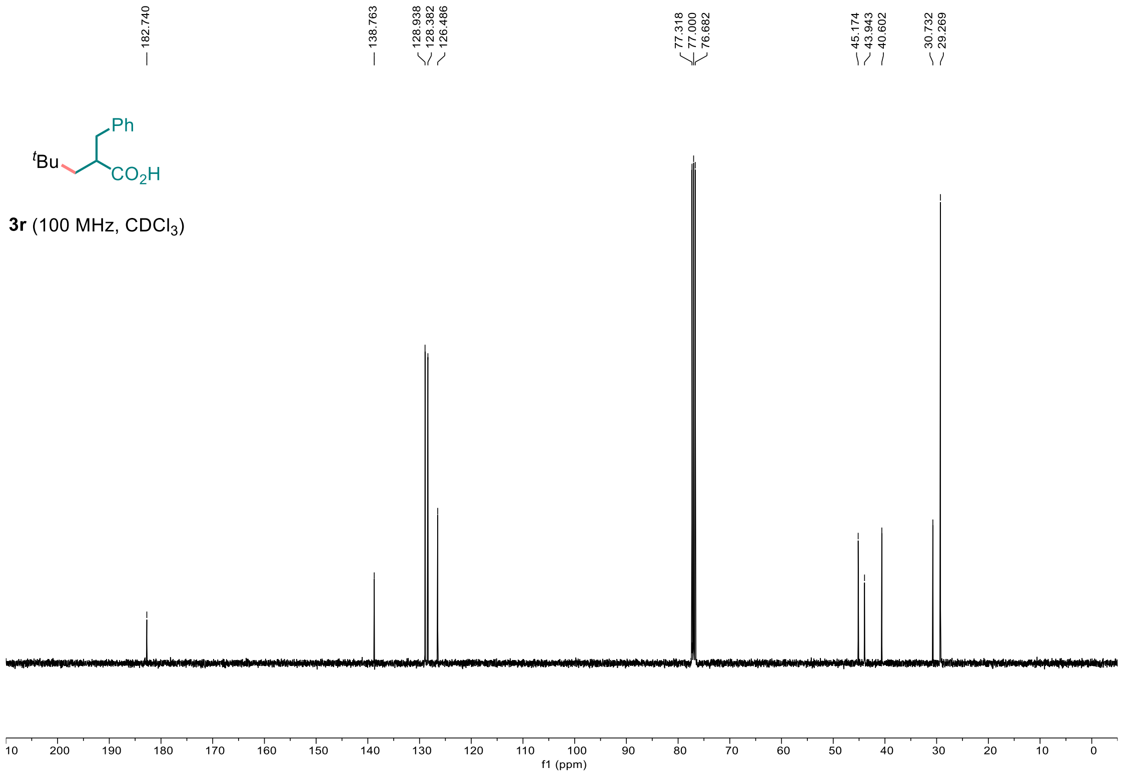

3r (100 MHz, $CDCl_3$)

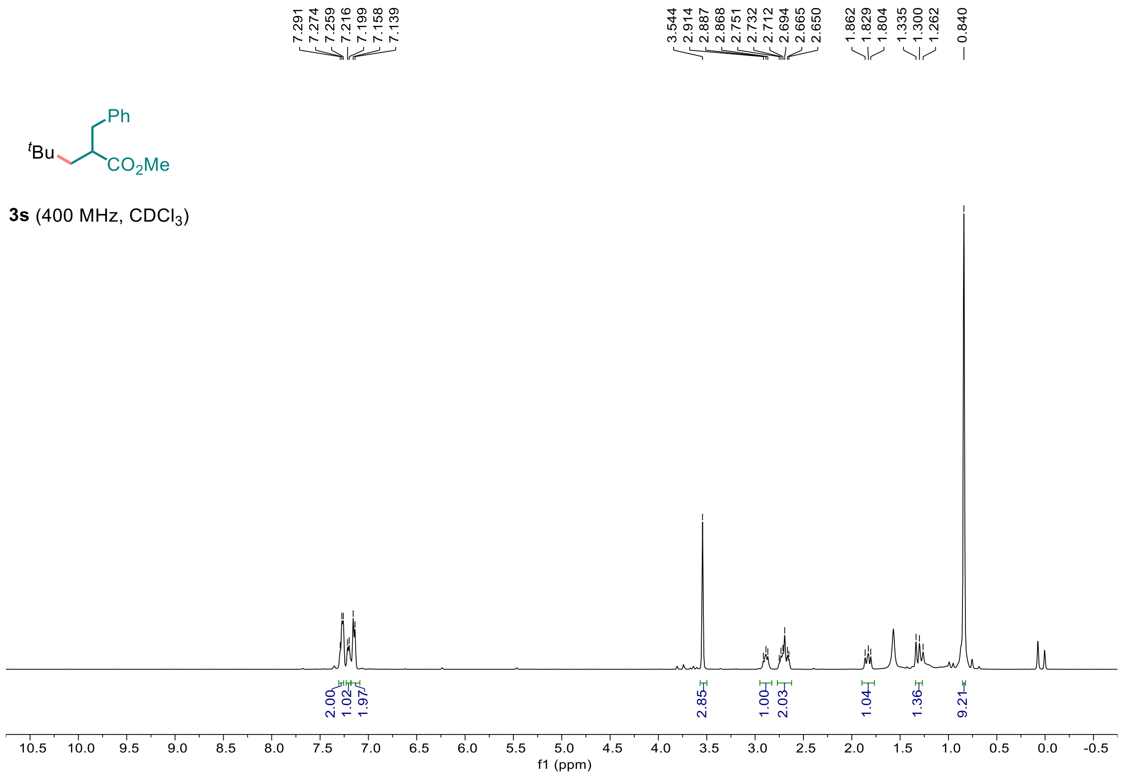

3s (400 MHz, $CDCl_3$)

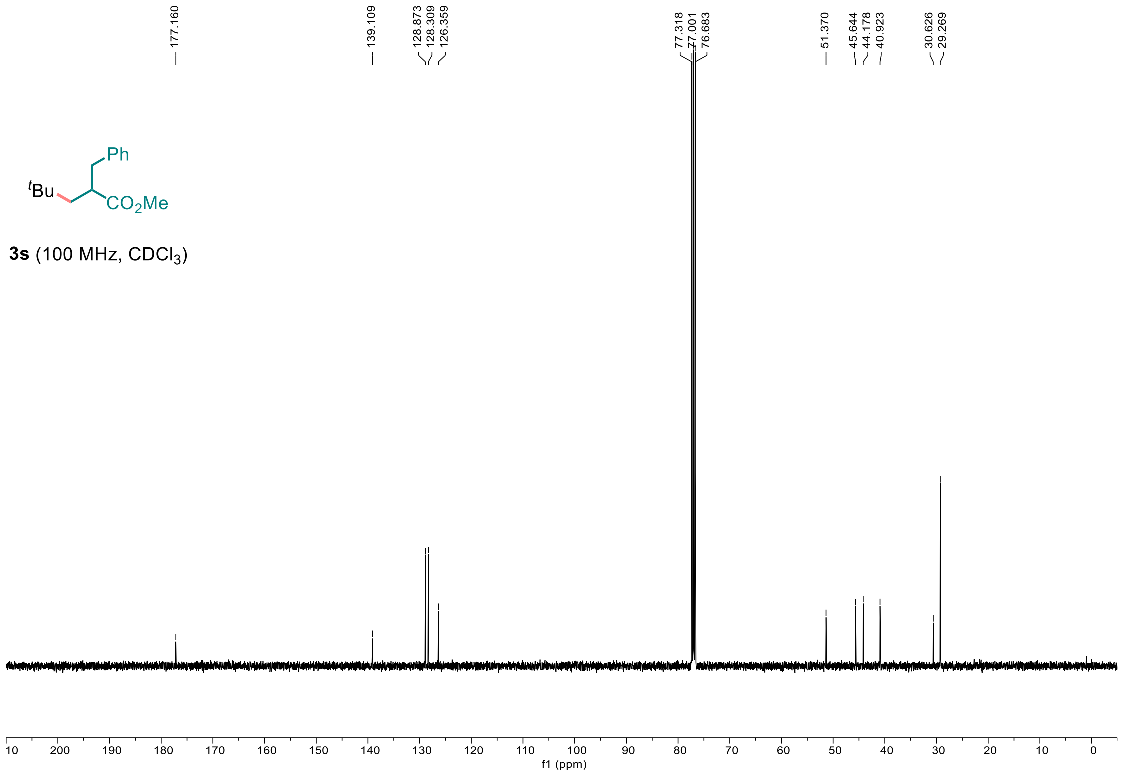

3s (100 MHz, $CDCl_3$)

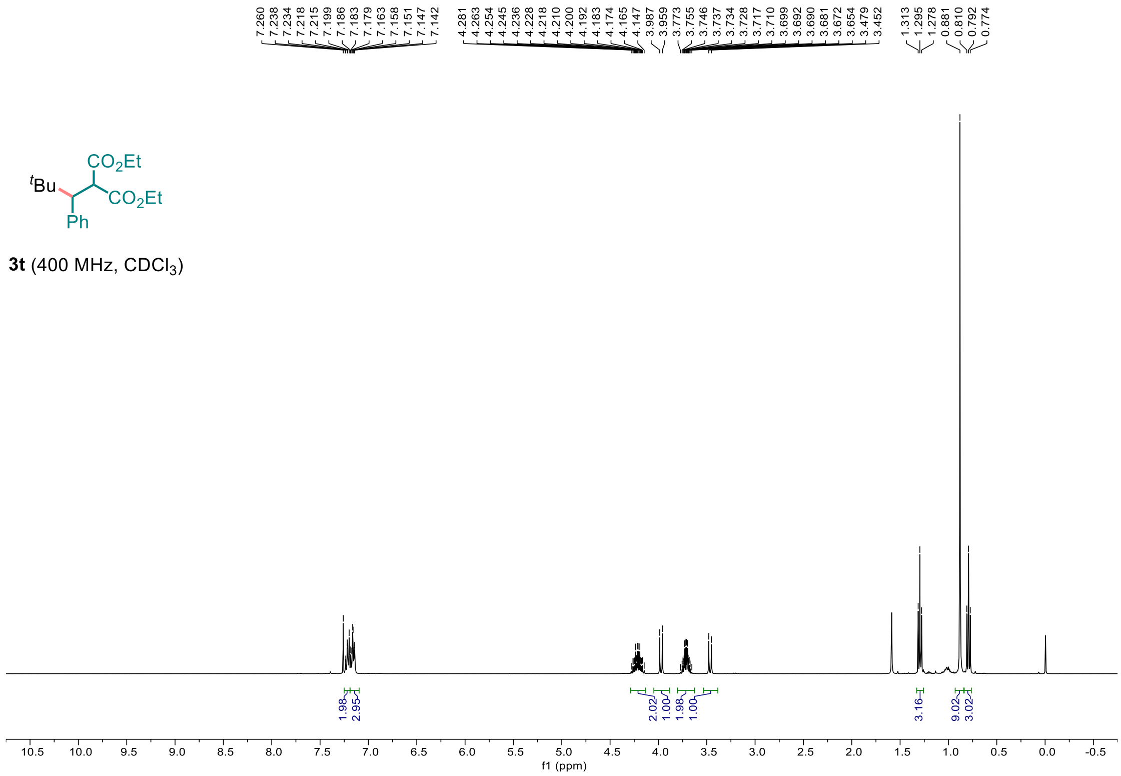

3t (400 MHz, $CDCl_3$)

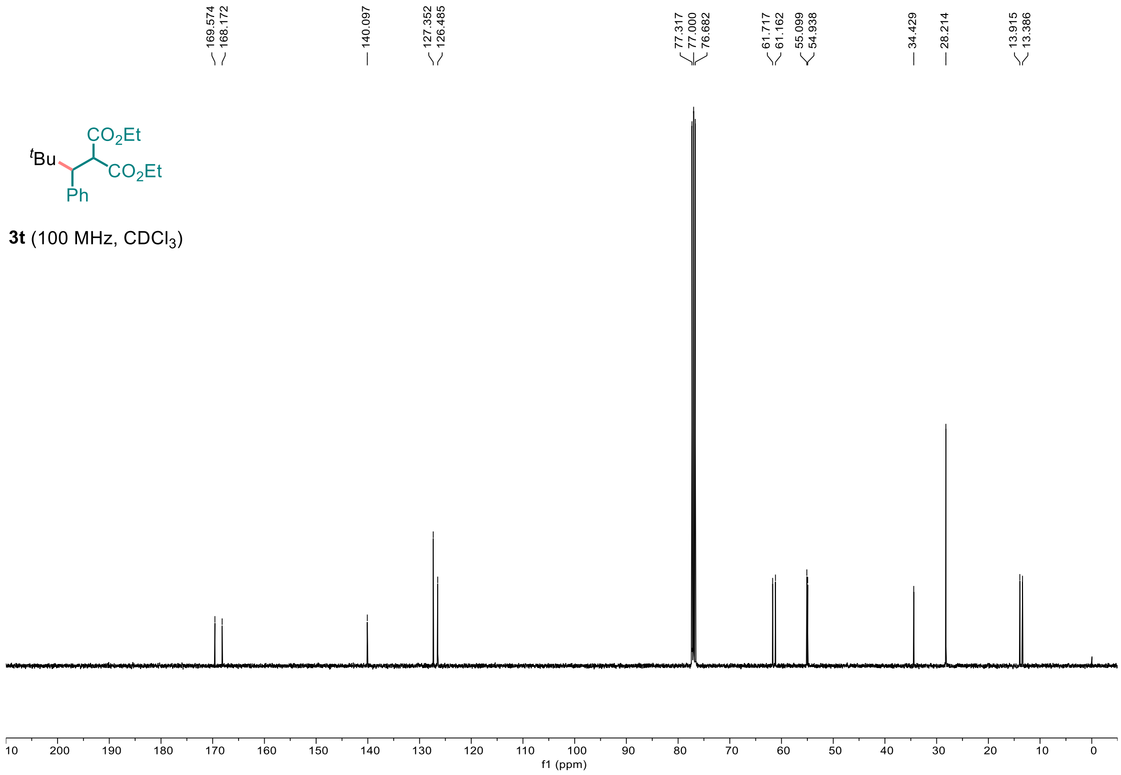

3t (100 MHz, $CDCl_3$)

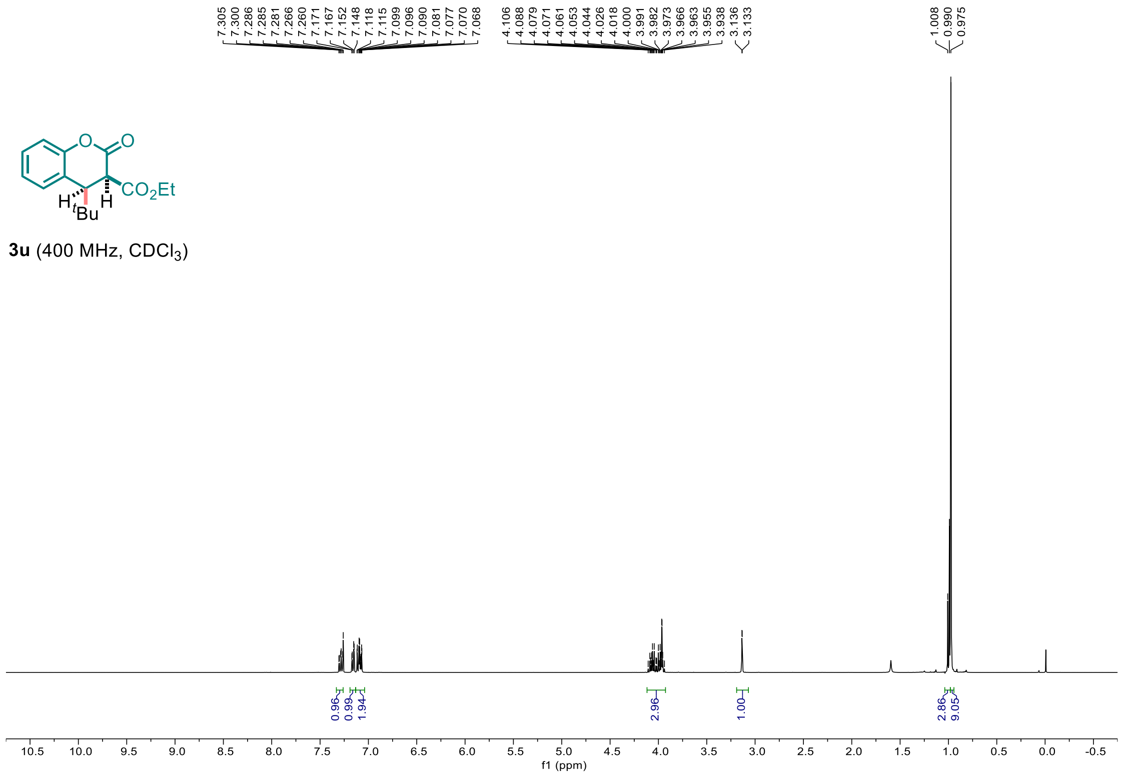

3u (400 MHz, $CDCl_3$)

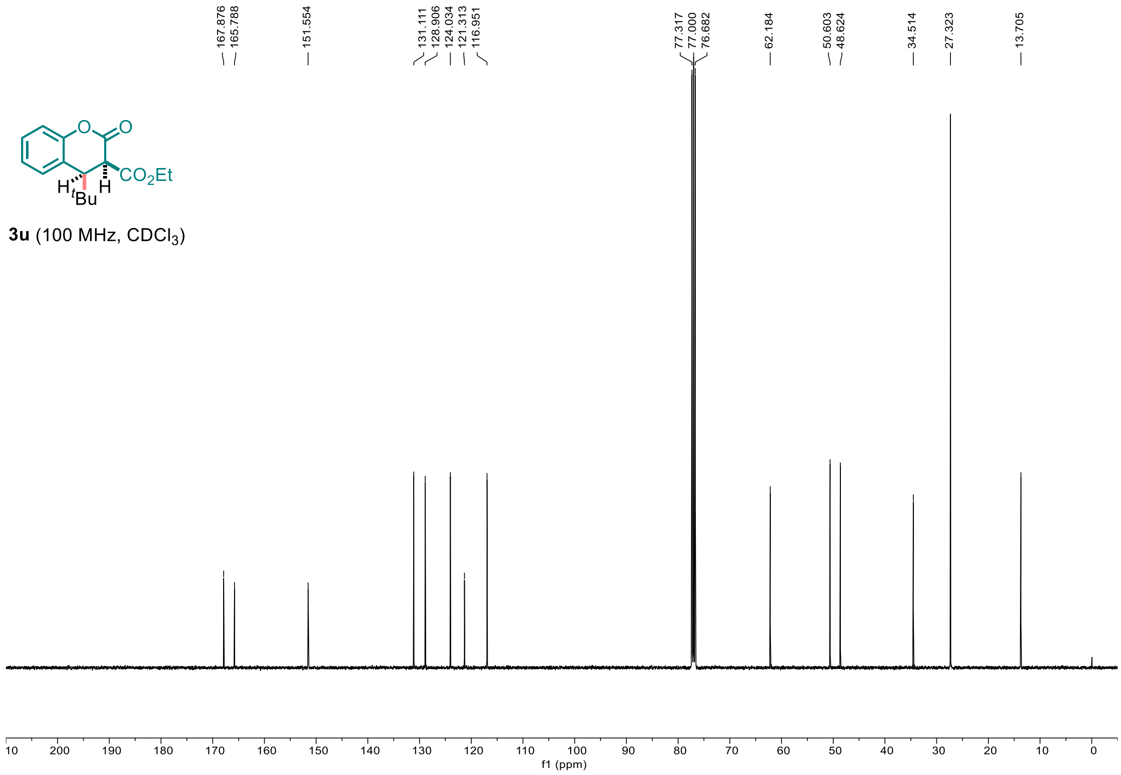

3u (100 MHz, $CDCl_3$)

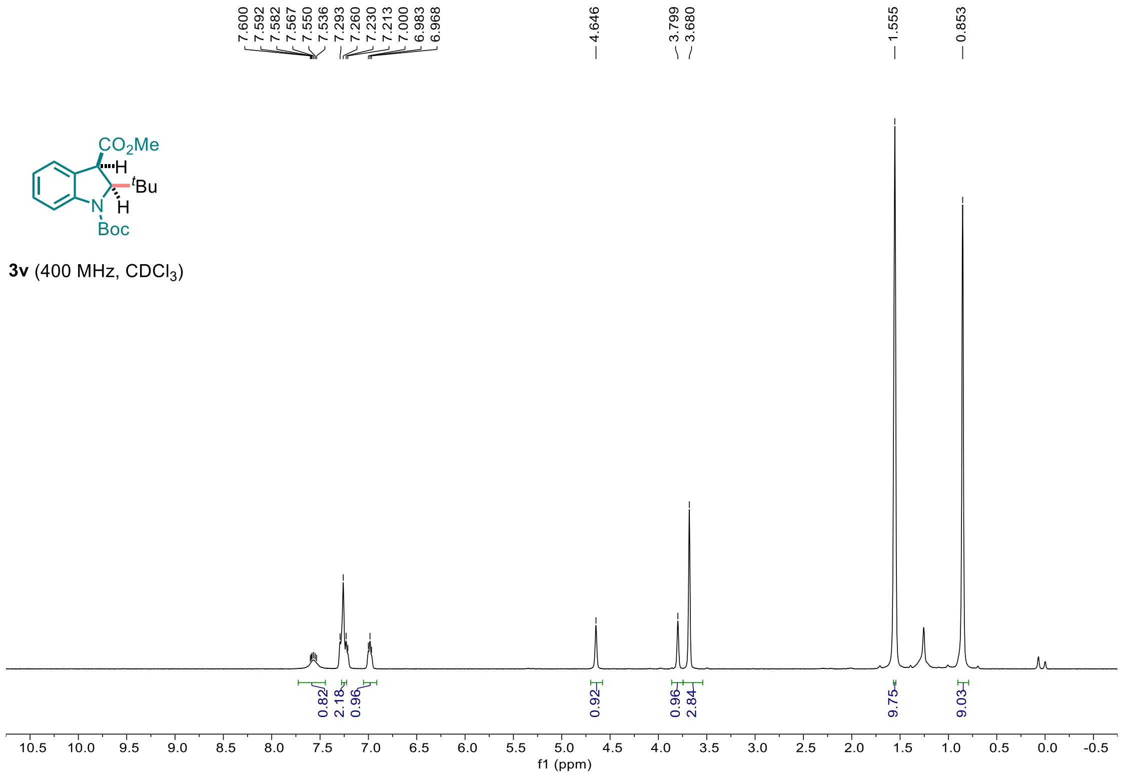

3v (400 MHz, $CDCl_3$)

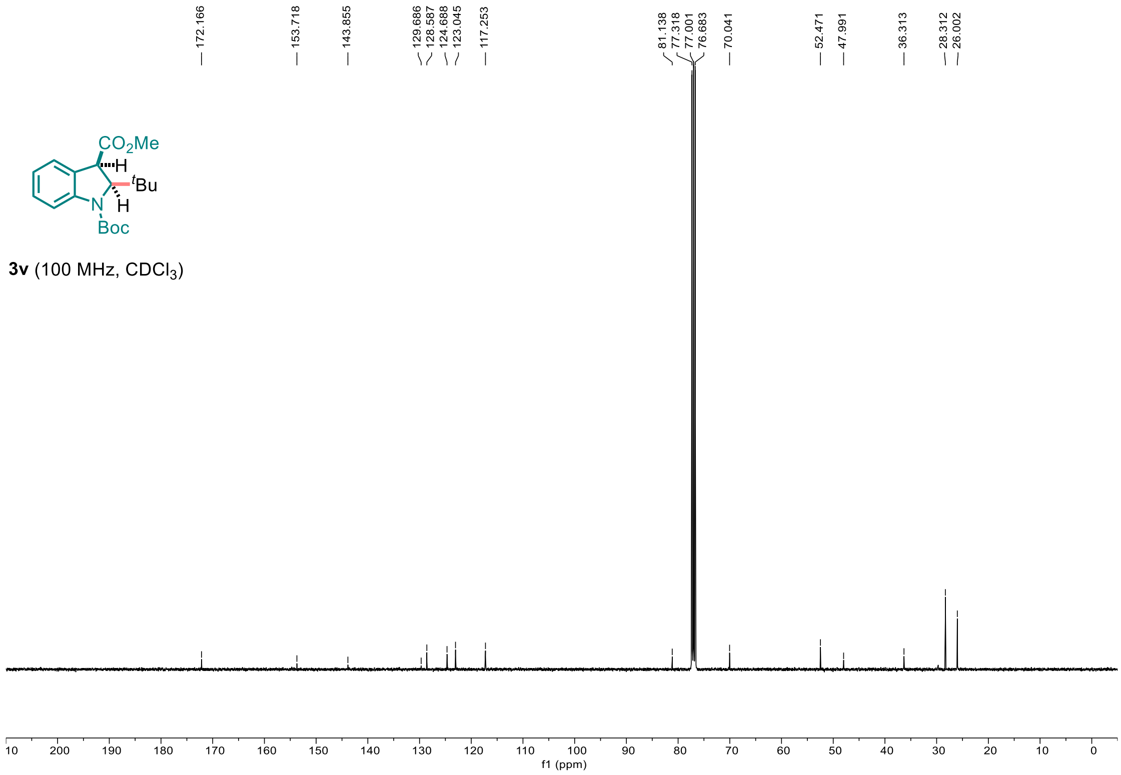
172.166
153.718
143.855
129.686
128.587
124.688
123.045
117.253
81.138
77.318
77.001
76.683
70.041
52.471
47.991
36.313
28.312
26.002
CO2Me
H
tBu
N
H
Boc
3v (100 MHz, CDCl3)
f1 (ppm)

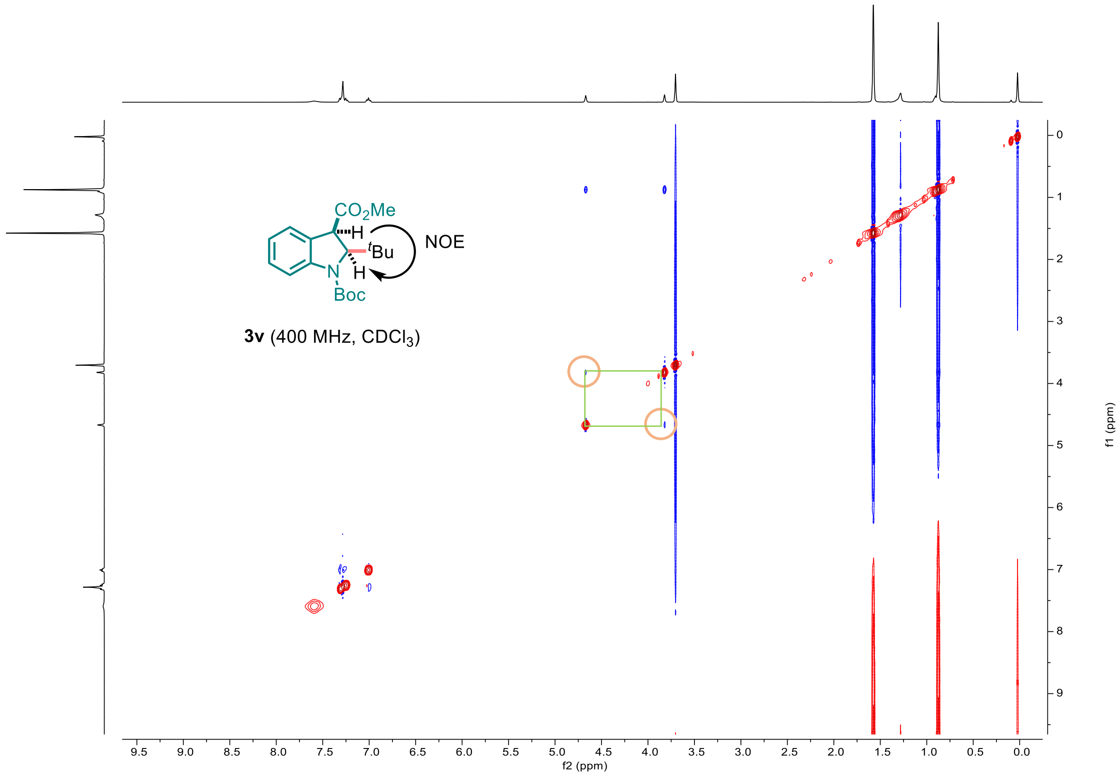
CO2Me
H
tBu
N
H
Boc
NOE
3v (400 MHz, CDCl3)
f2 (ppm)
f1 (ppm)

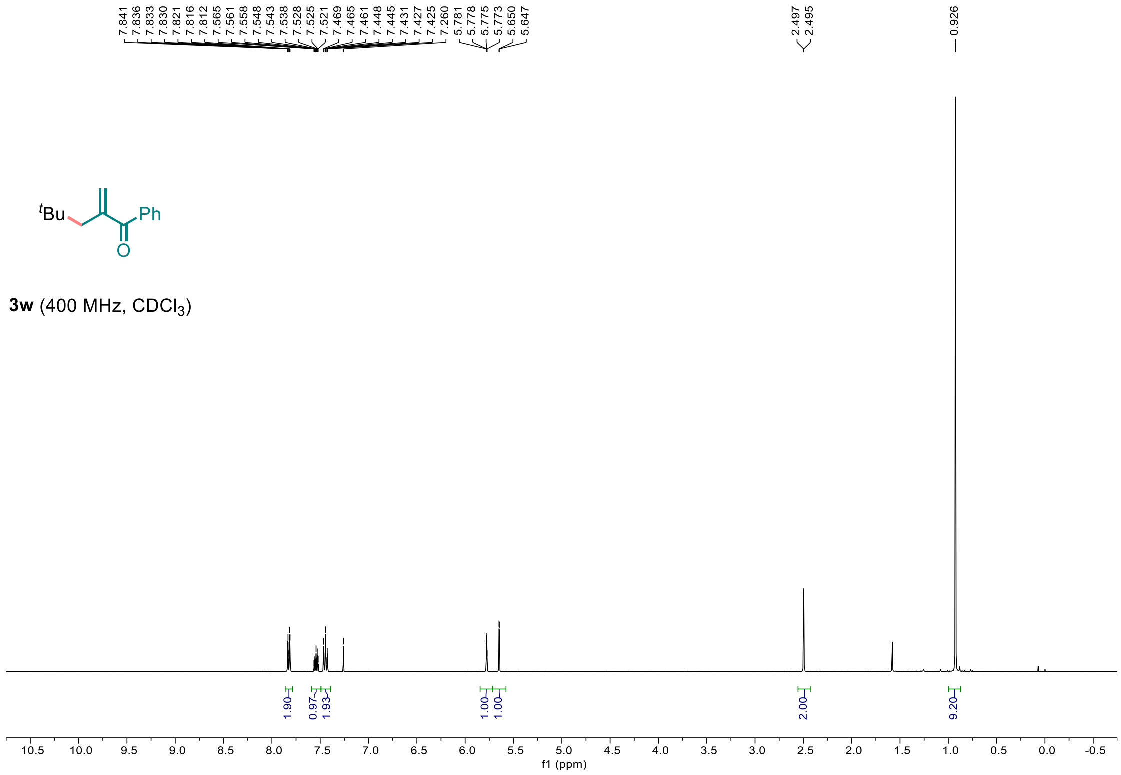

3w (400 MHz, $CDCl_3$)

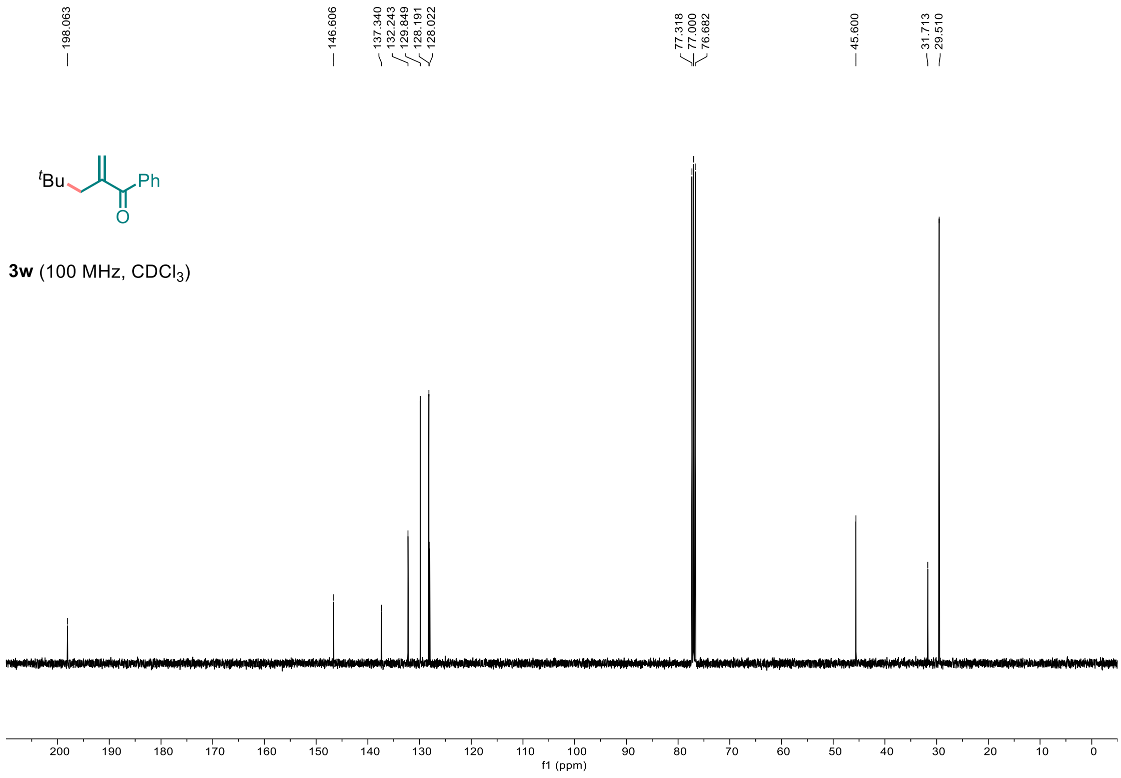

3w (100 MHz, $CDCl_3$)

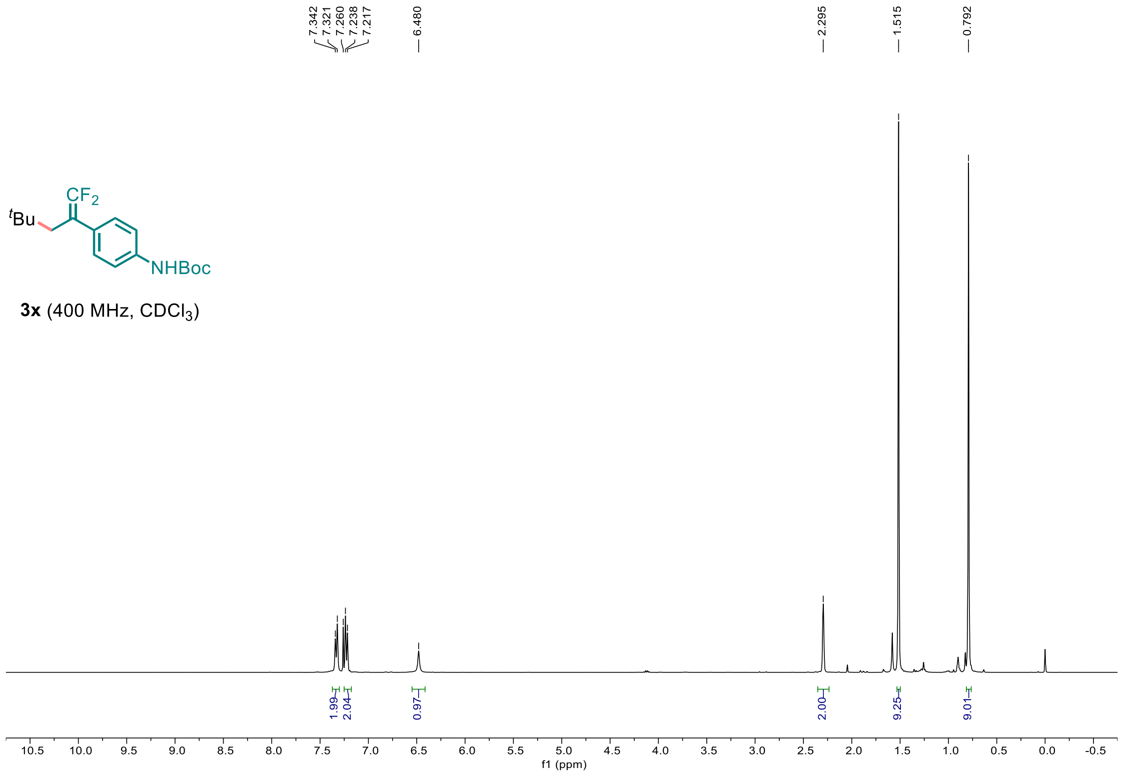

3x (400 MHz, $CDCl_3$)

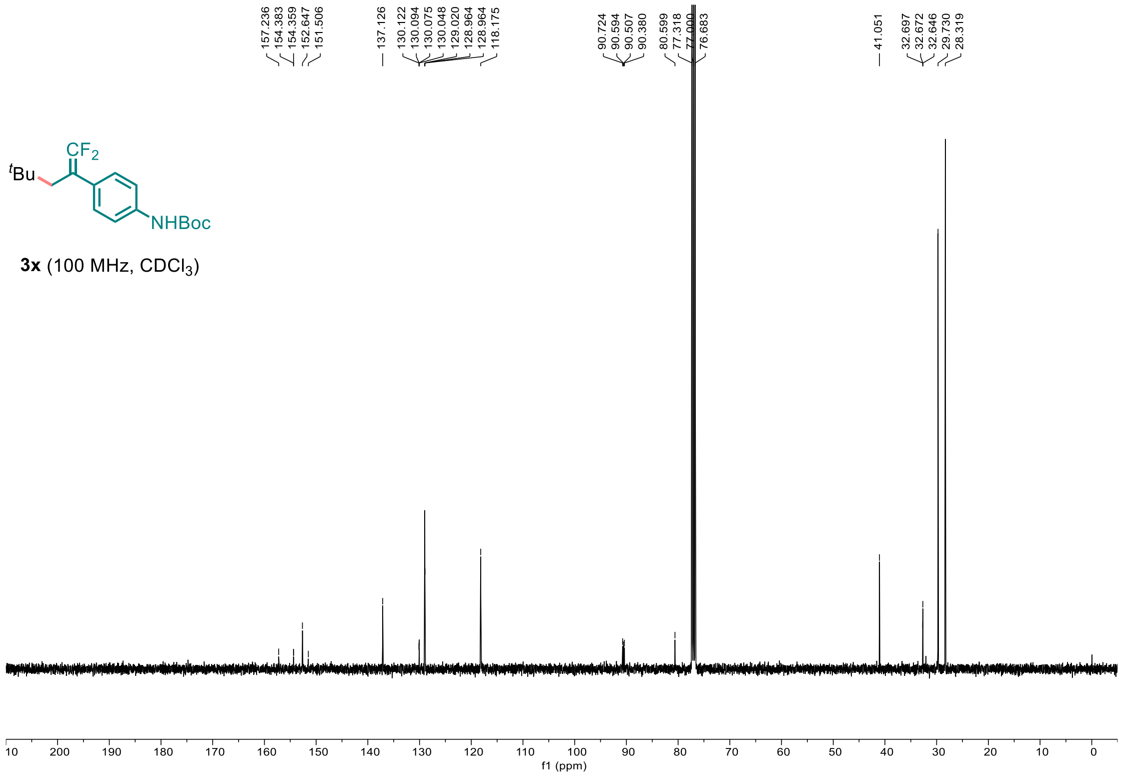

3x (100 MHz, $CDCl_3$)

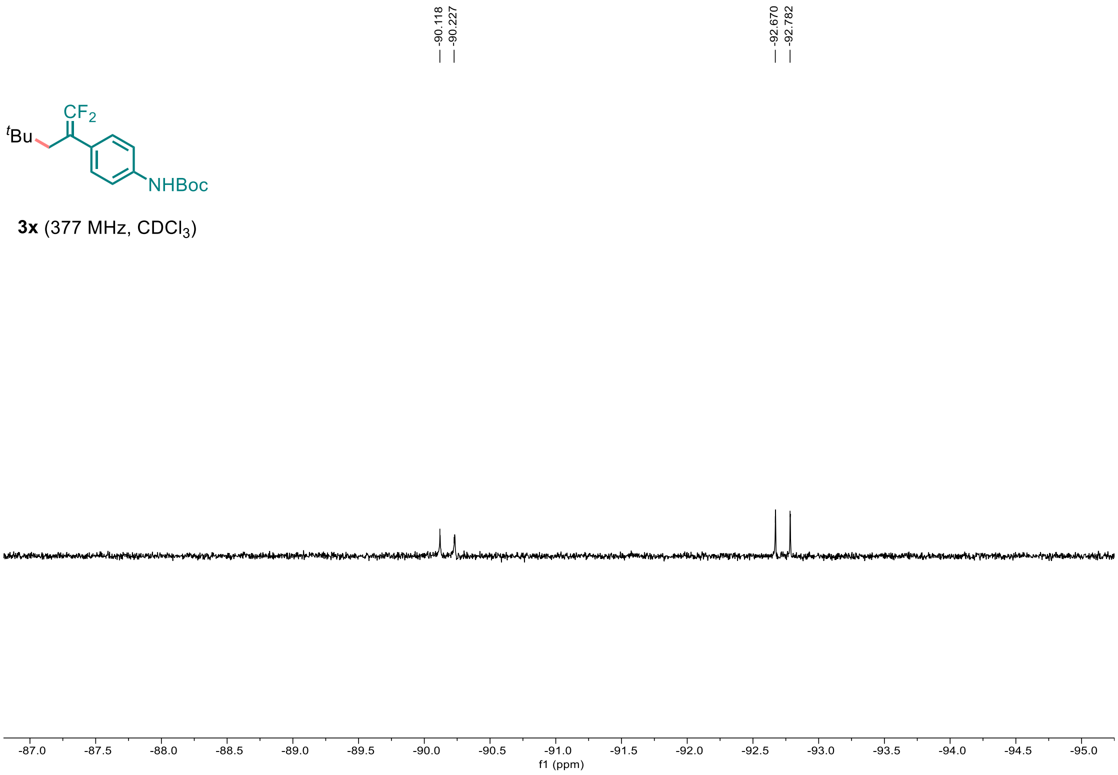

3x (377 MHz, $CDCl_3$)

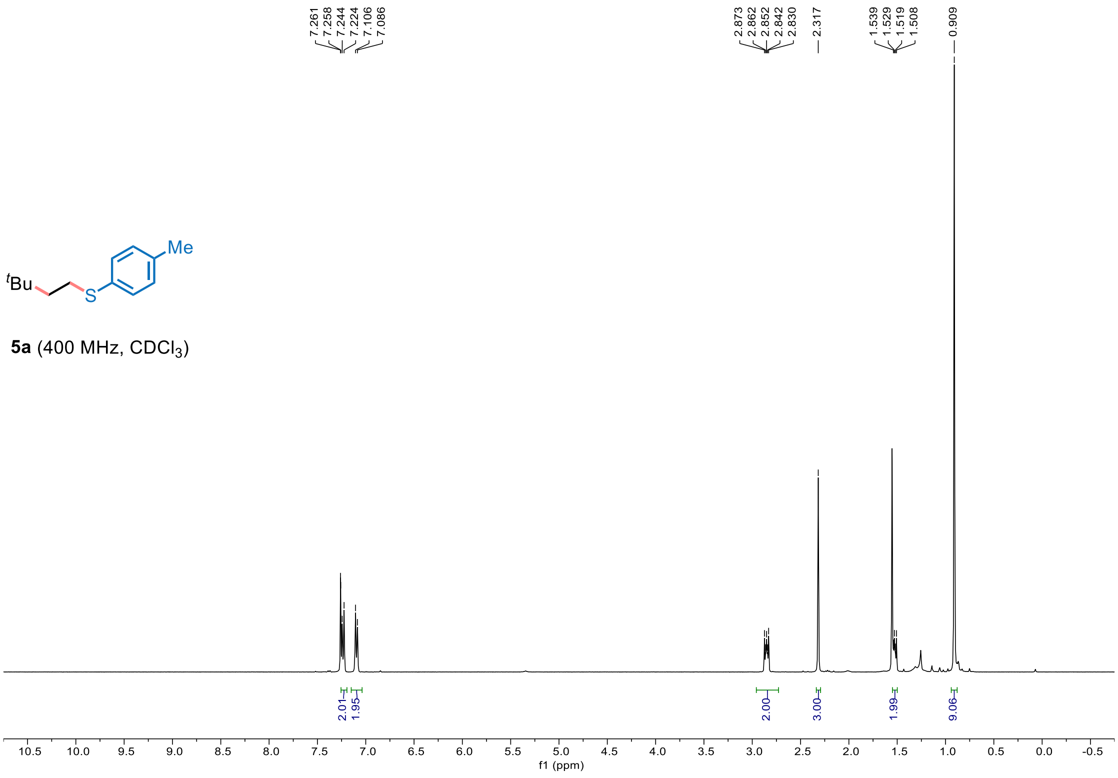

5a (400 MHz, $CDCl_3$)

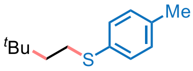

5a (100 MHz, $CDCl_3$)

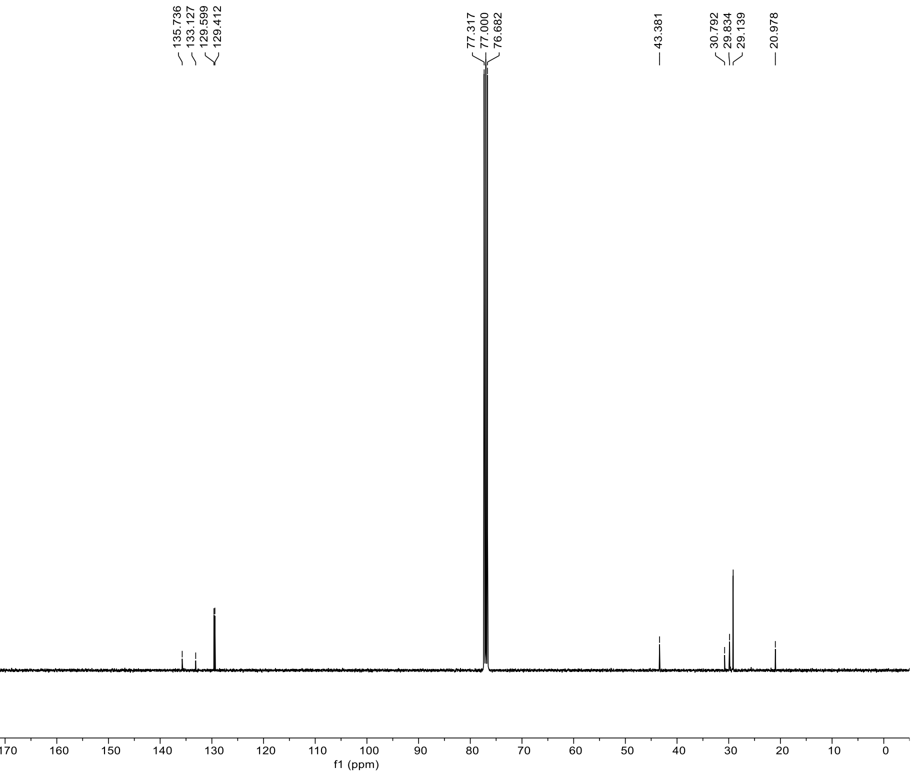

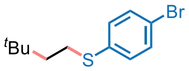

5b (400 MHz, $CDCl_3$)

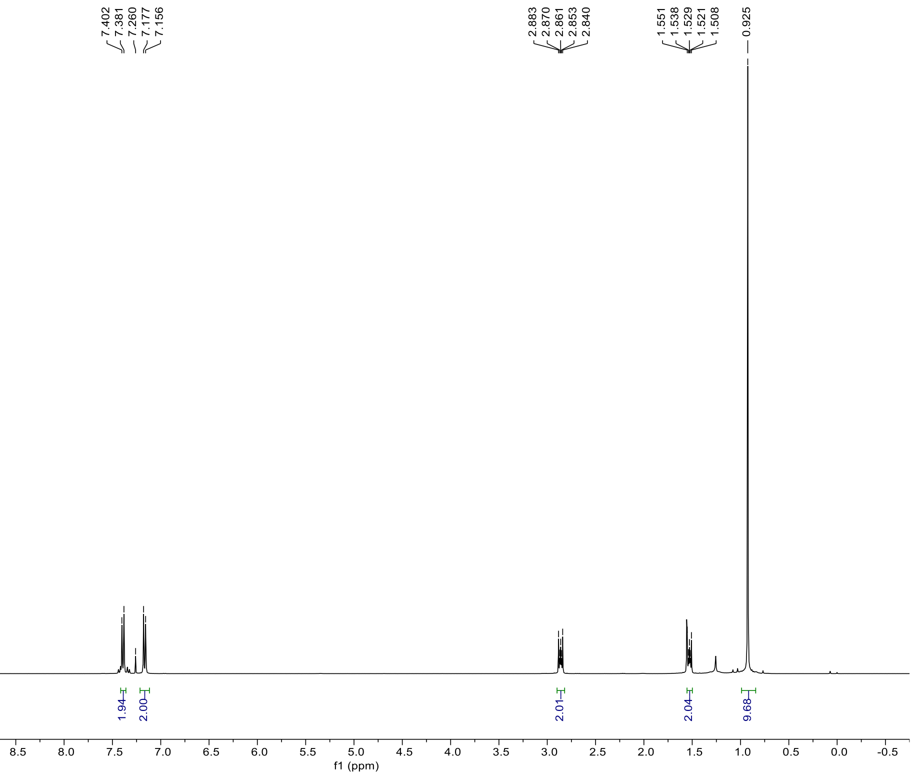

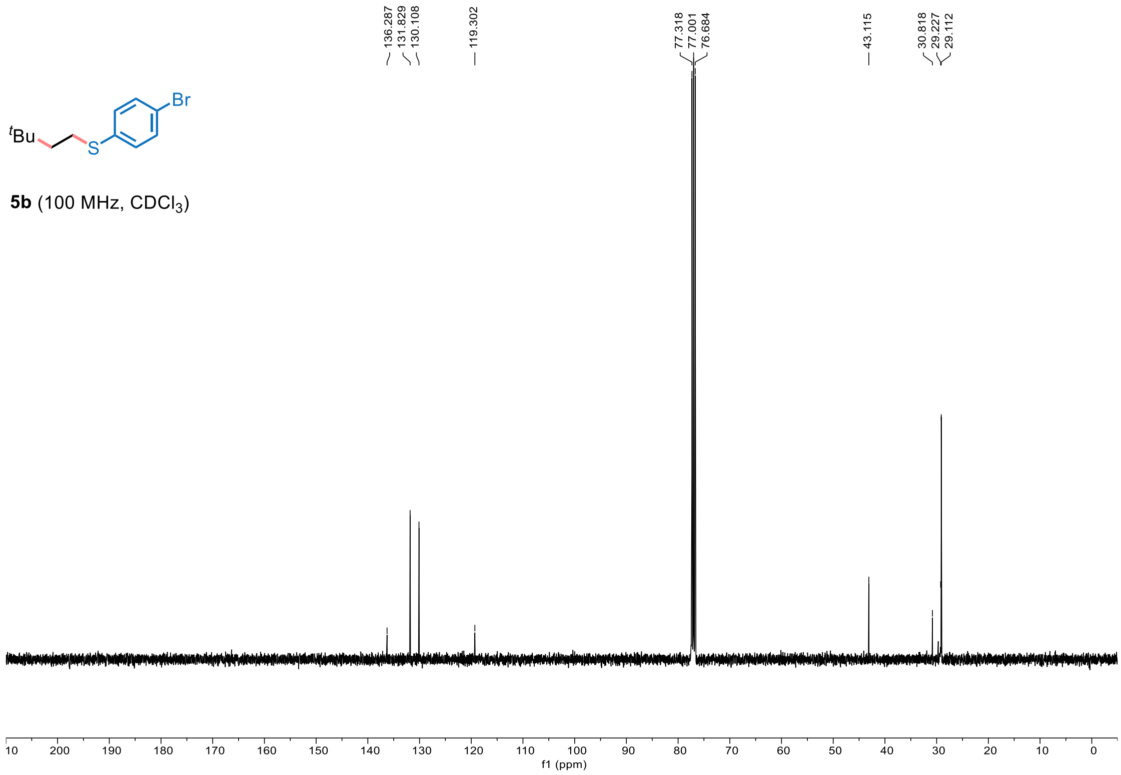

5b (100 MHz, $CDCl_3$)

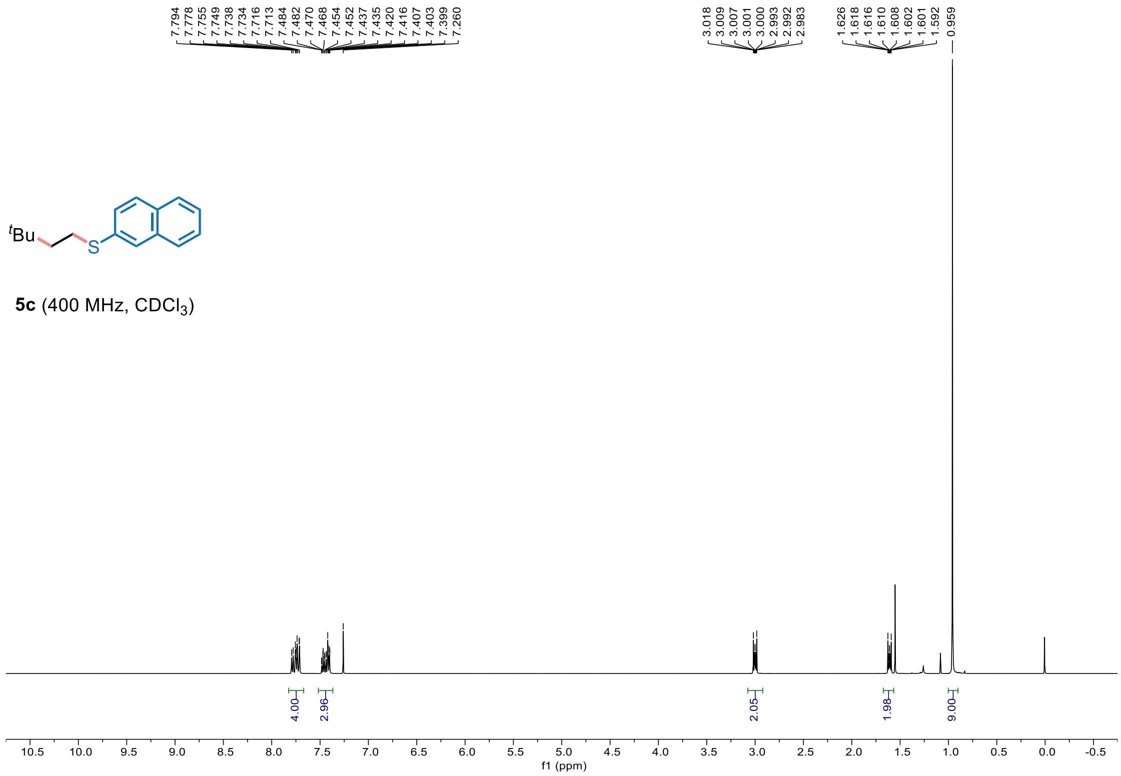

5c (400 MHz, $CDCl_3$)

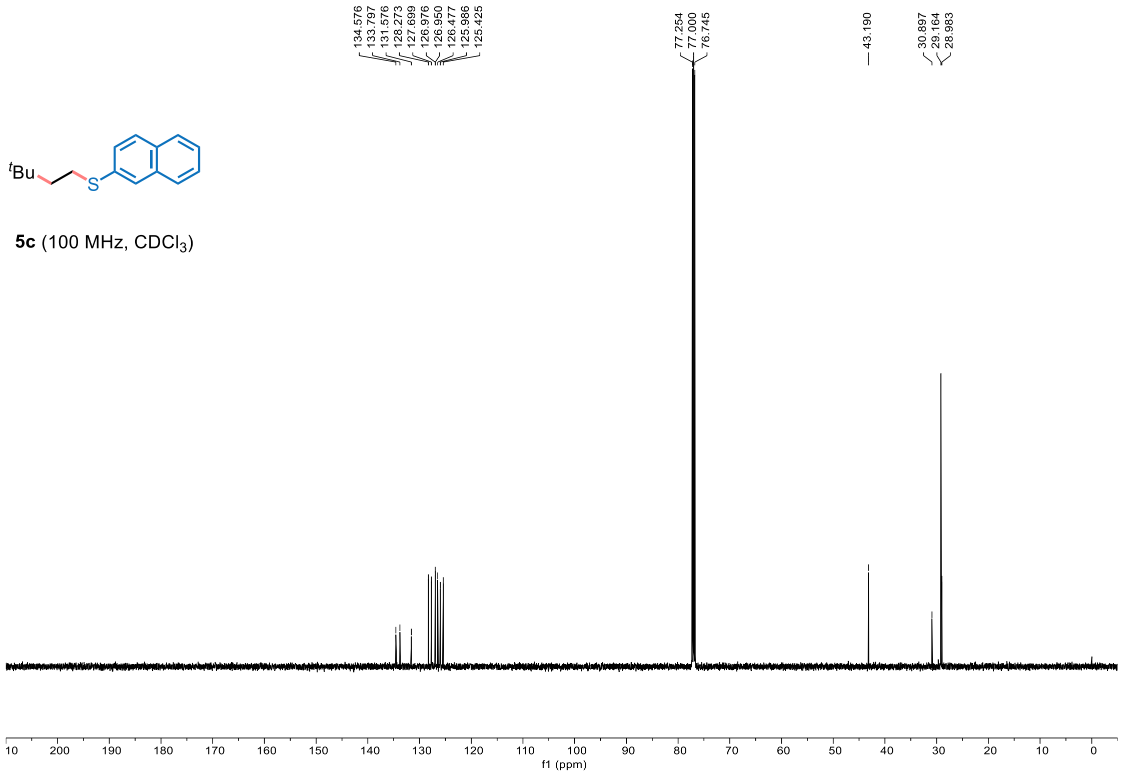
tBu
S
5c (100 MHz, CDCl3)
134.576
133.797
131.576
128.273
127.699
126.976
126.950
126.477
125.986
125.425
77.254
77.000
76.745
43.190
30.897
29.164
28.983
f1 (ppm)

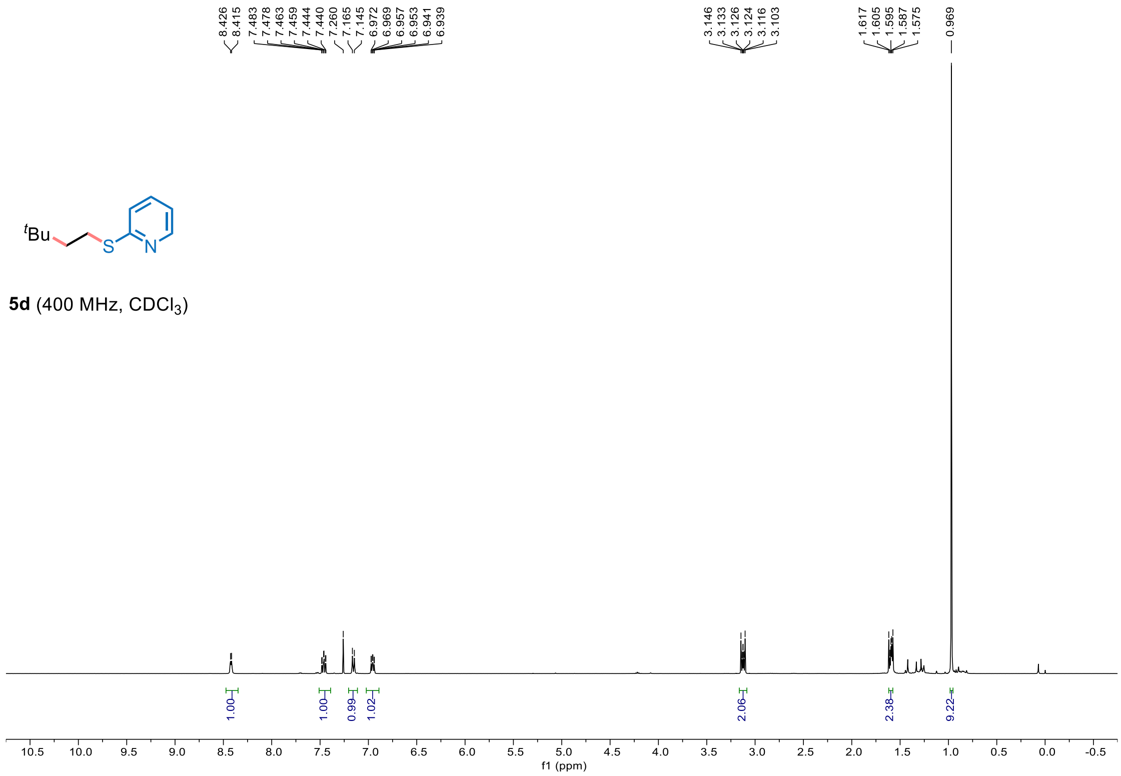
tBu
S
N
5d (400 MHz, CDCl3)
8.426
8.415
7.483
7.478
7.463
7.459
7.444
7.440
7.260
7.165
7.145
6.972
6.969
6.957
6.953
6.941
6.939
3.146
3.133
3.126
3.124
3.116
3.103
1.617
1.605
1.595
1.587
1.575
0.969
1.00
1.00
0.99
1.02
2.06
2.38
9.22
f1 (ppm)

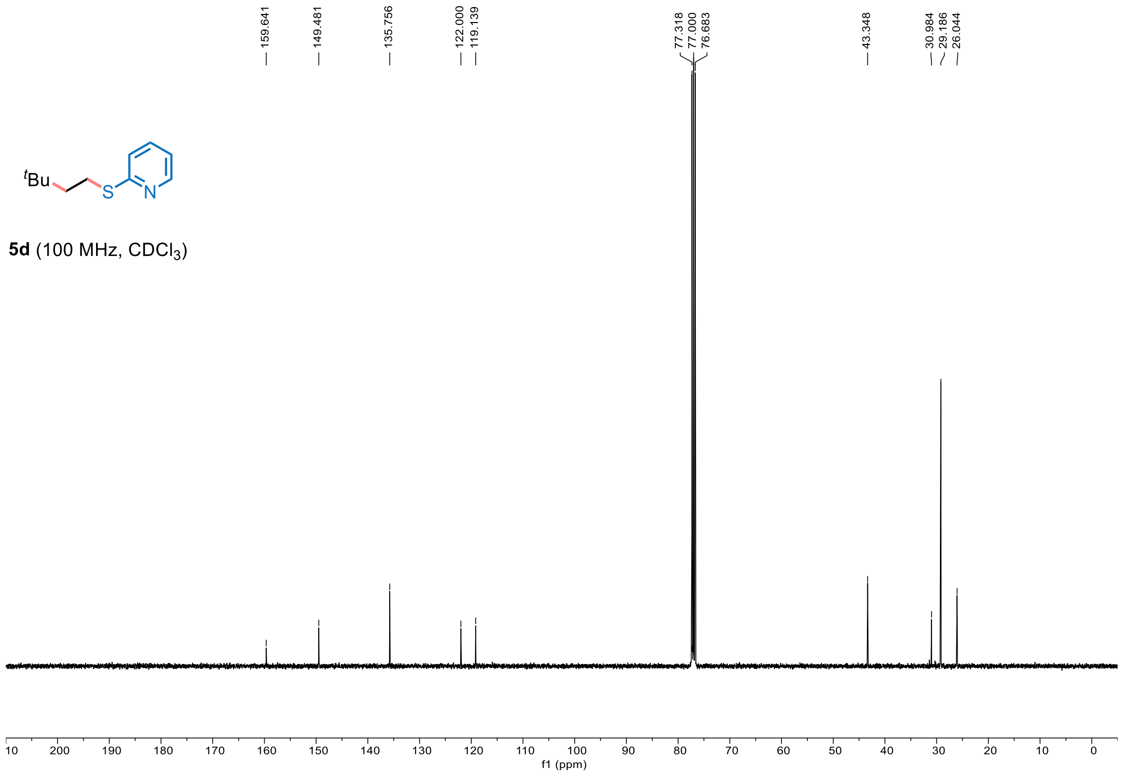

5d (100 MHz, $CDCl_3$)

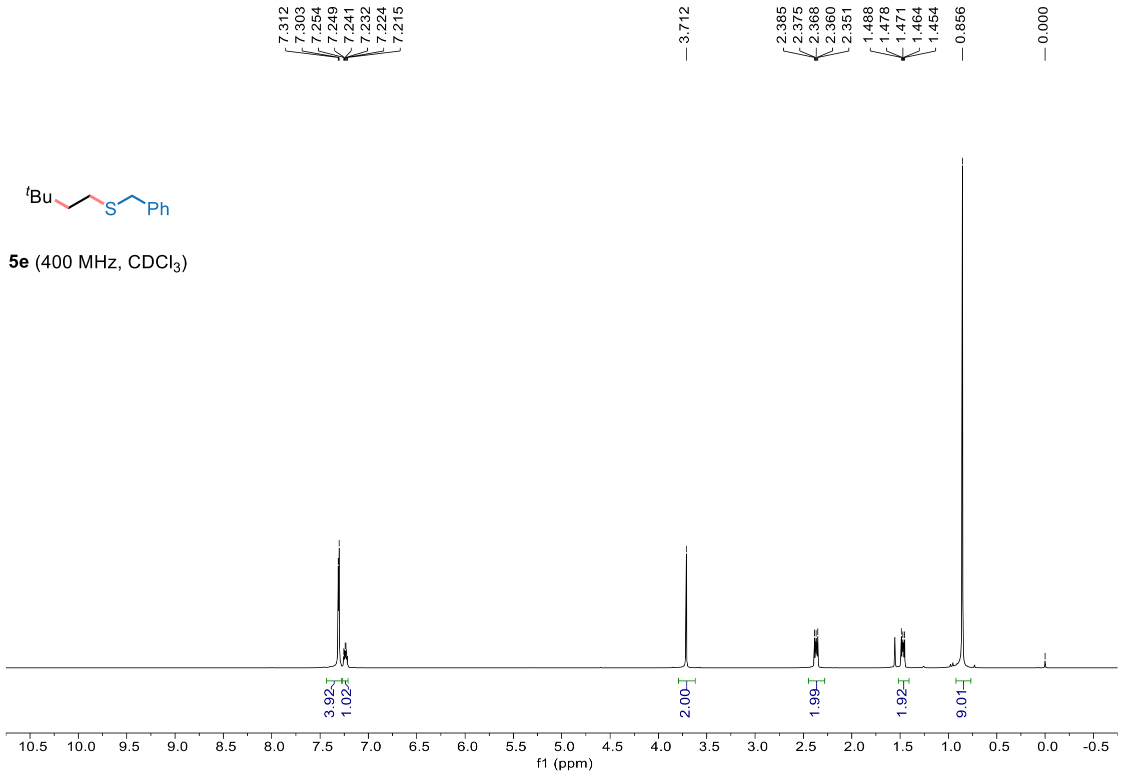

5e (400 MHz, $CDCl_3$)

5e (100 MHz, $CDCl_3$)

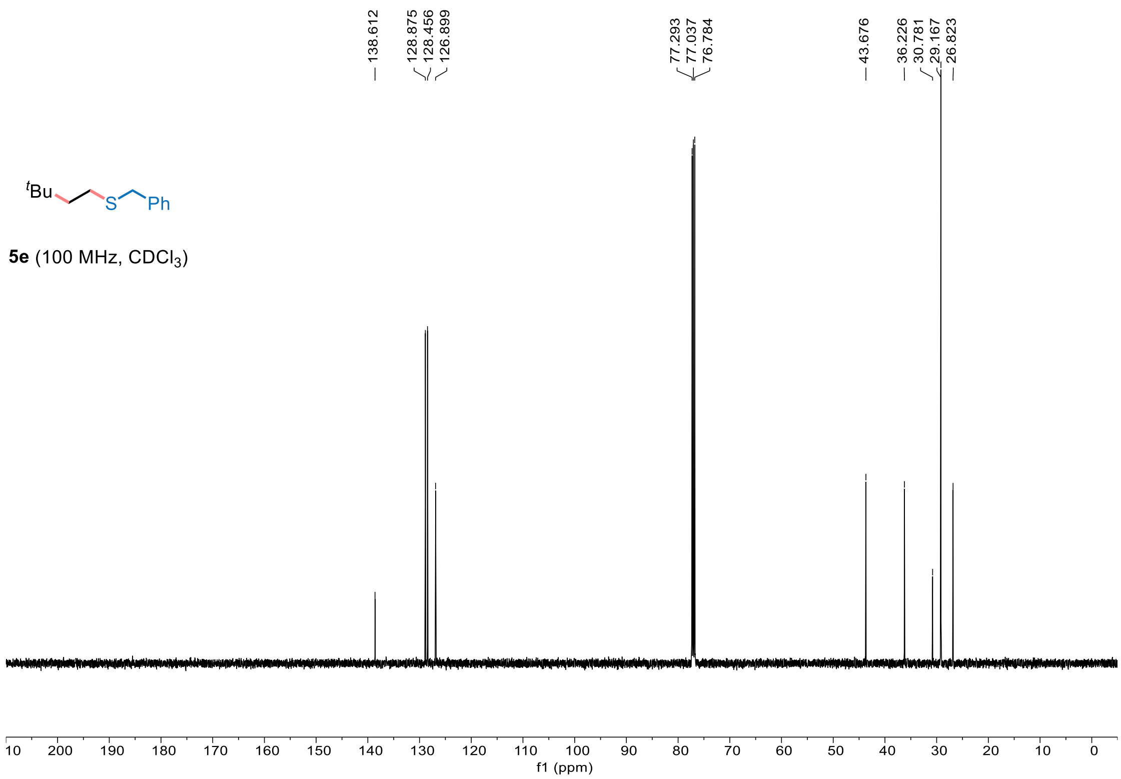

5f (400 MHz, $CDCl_3$)

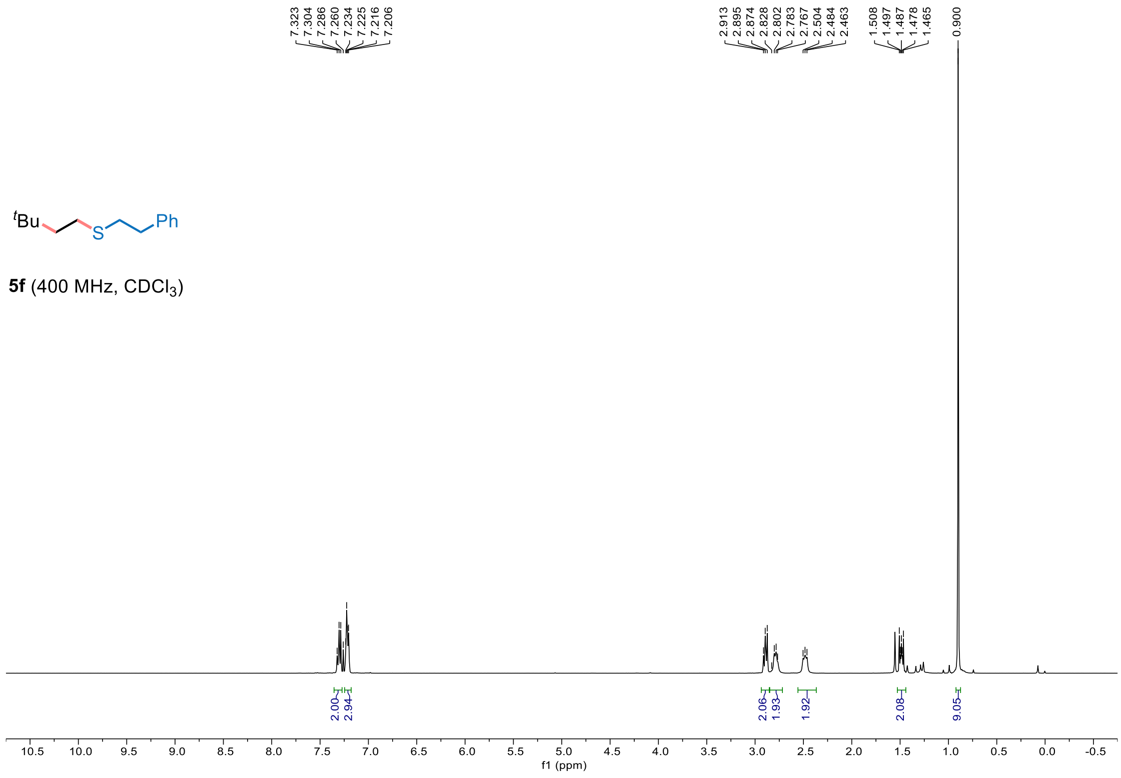

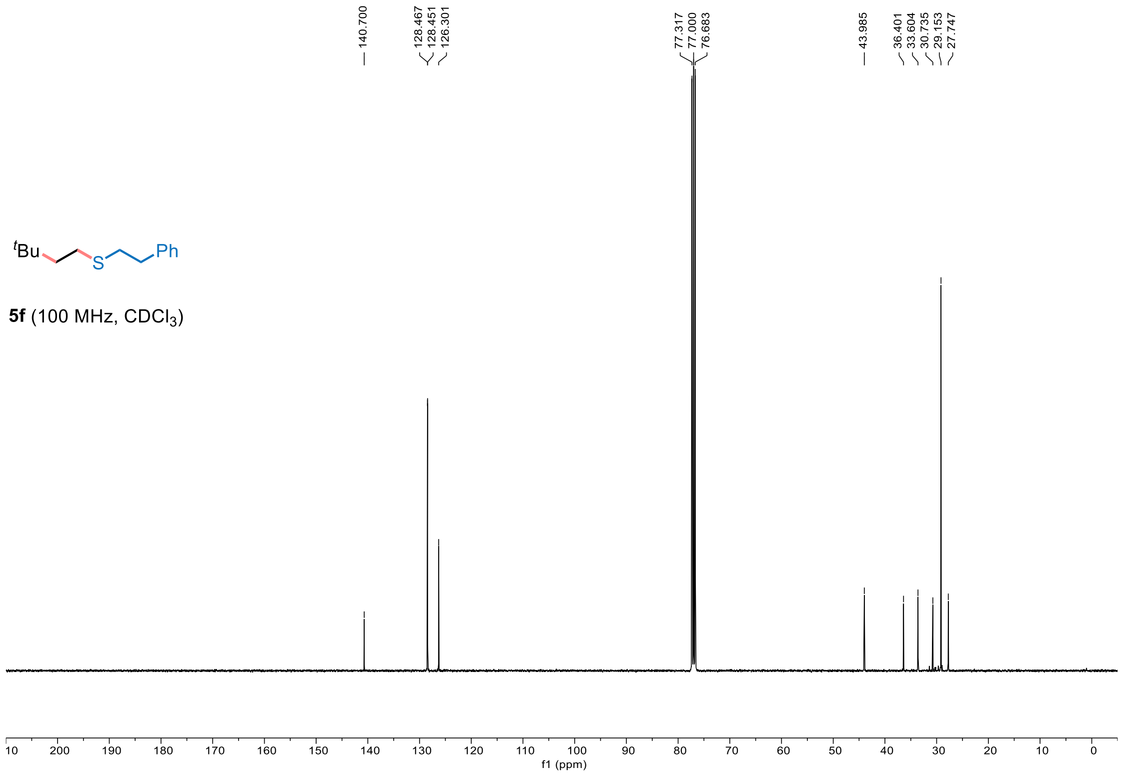

5f (100 MHz, $CDCl_3$)

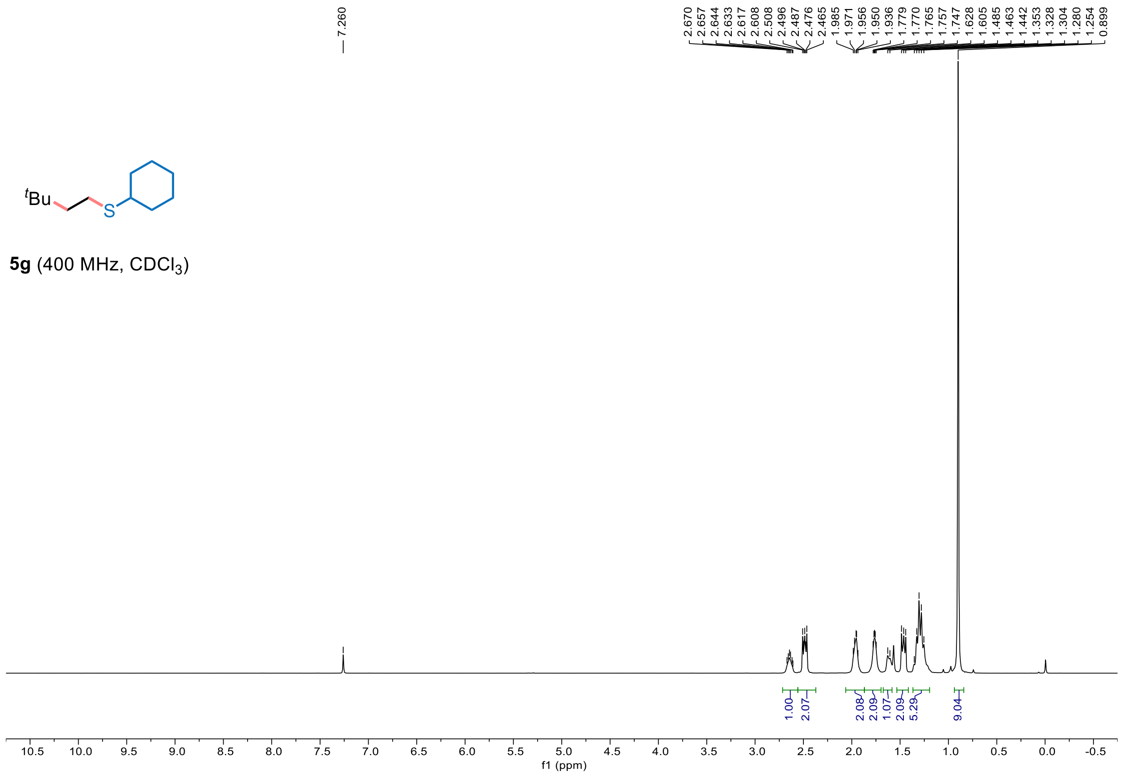

5g (400 MHz, $CDCl_3$)

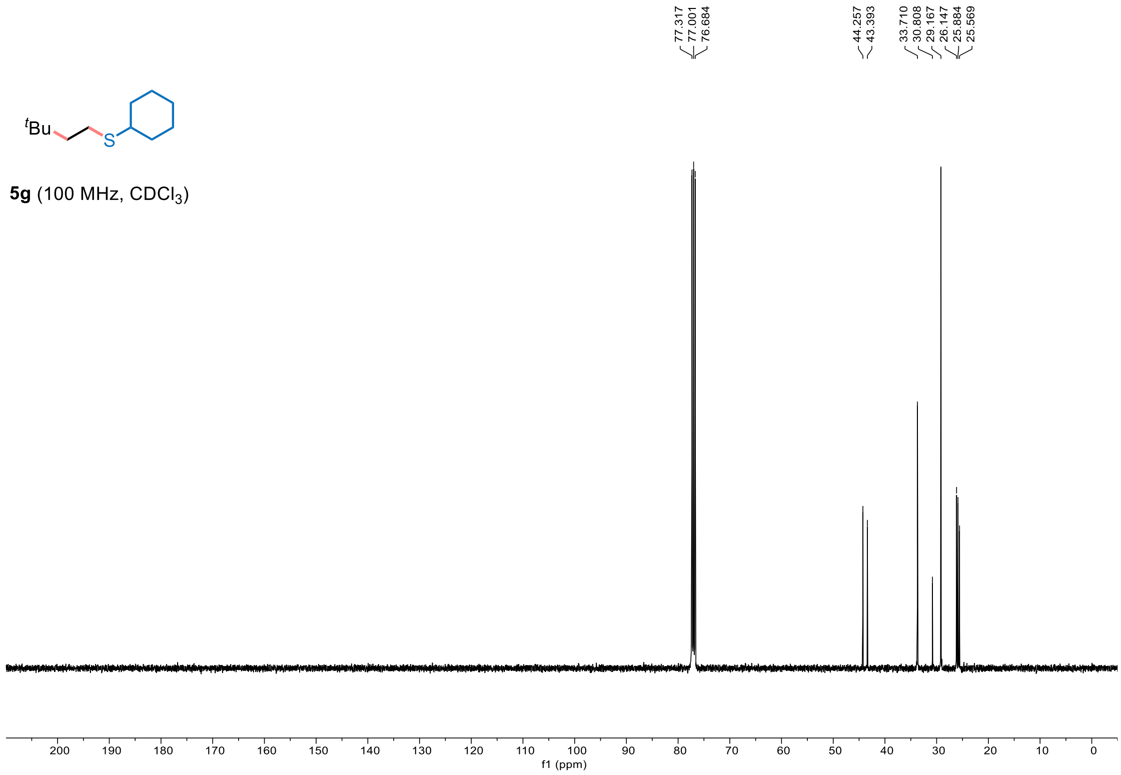

5g (100 MHz, $CDCl_3$)

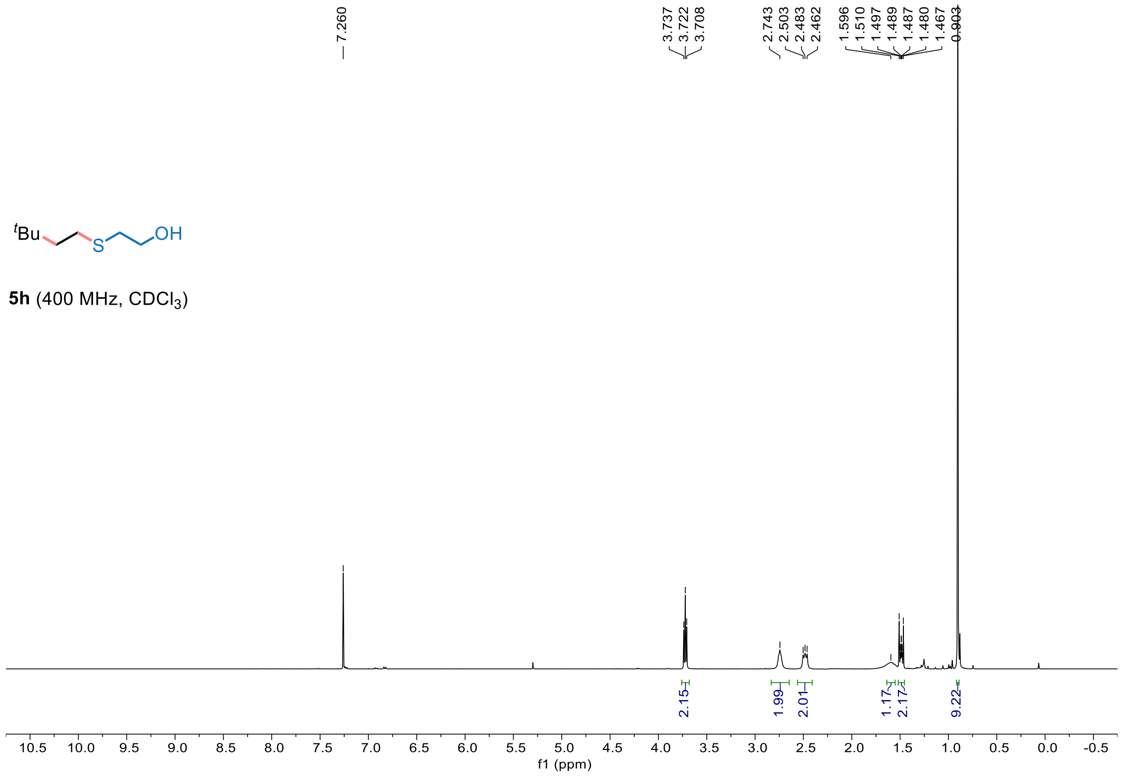

5h (400 MHz, $CDCl_3$)

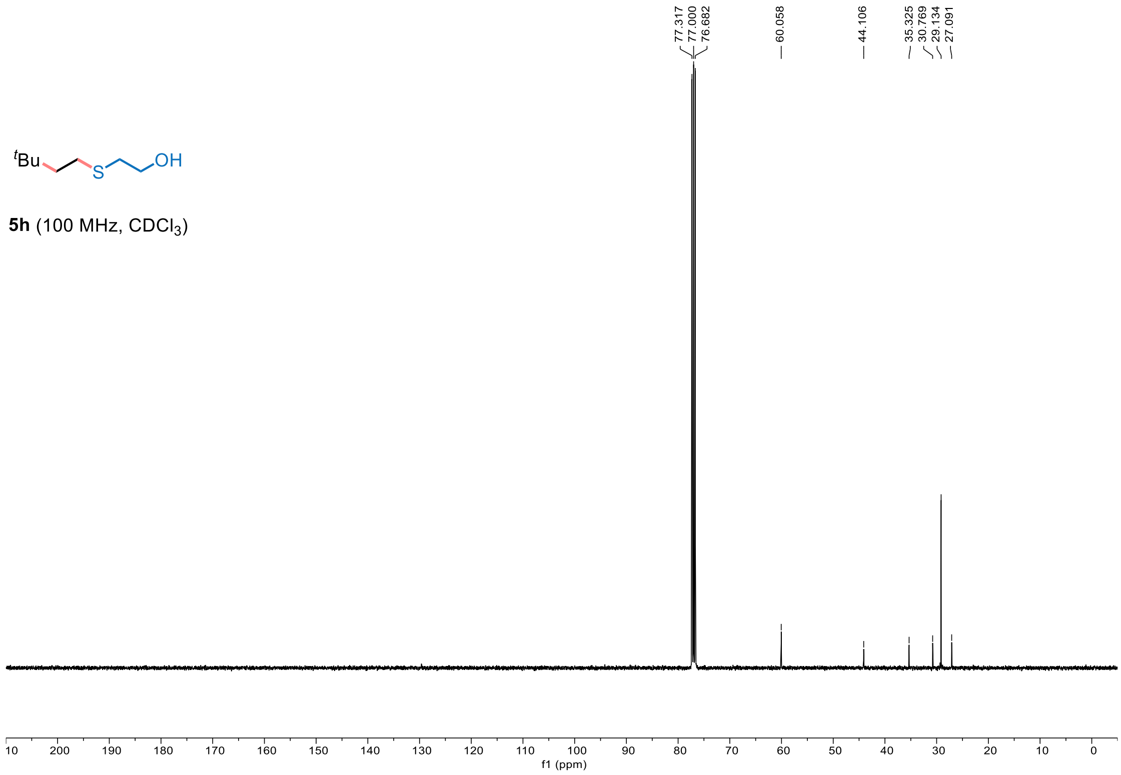

5h (100 MHz, $CDCl_3$)

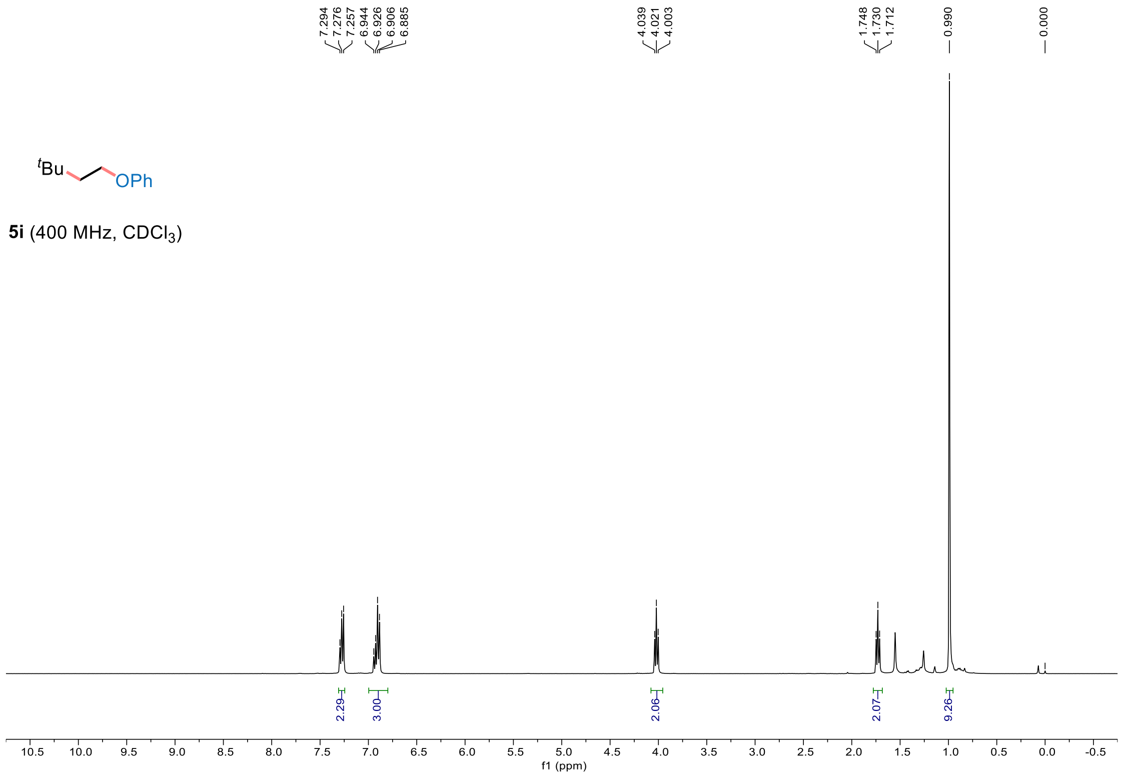

5i (400 MHz, $CDCl_3$)

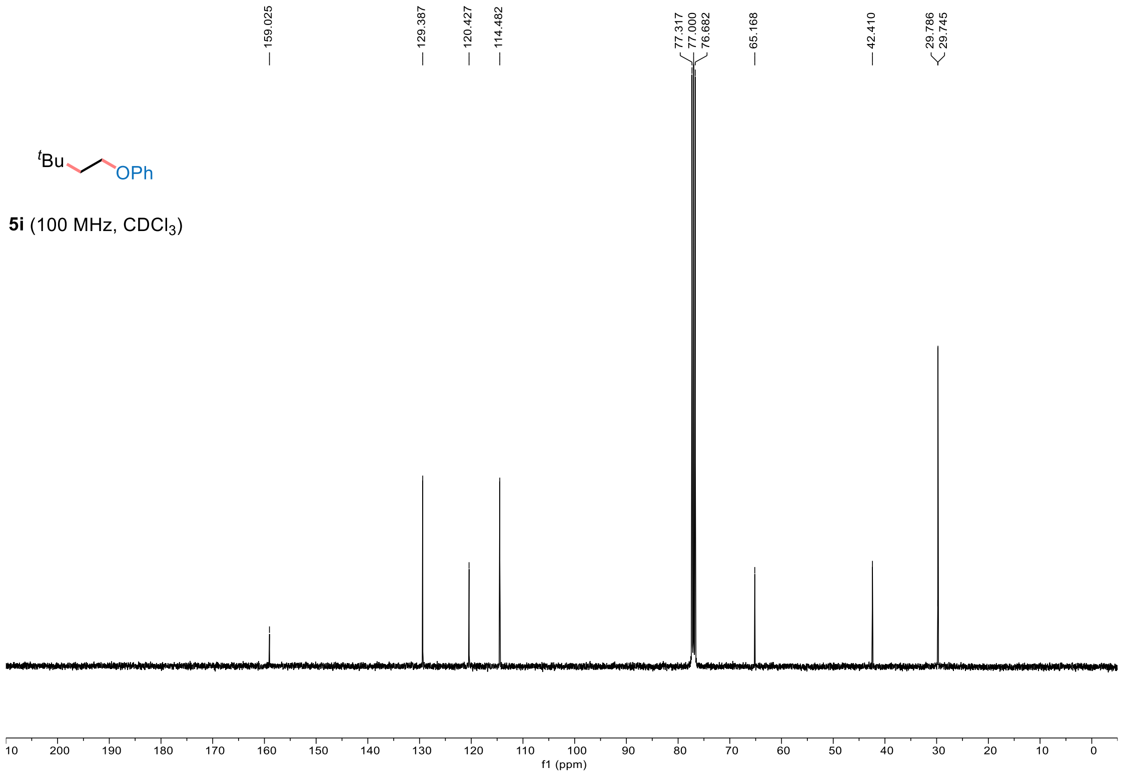

159.025
129.387
120.427
114.482
77.317
77.000
76.682
65.168
42.410
29.786
29.745
tBu OPh
5i (100 MHz, CDCl3)
f1 (ppm)

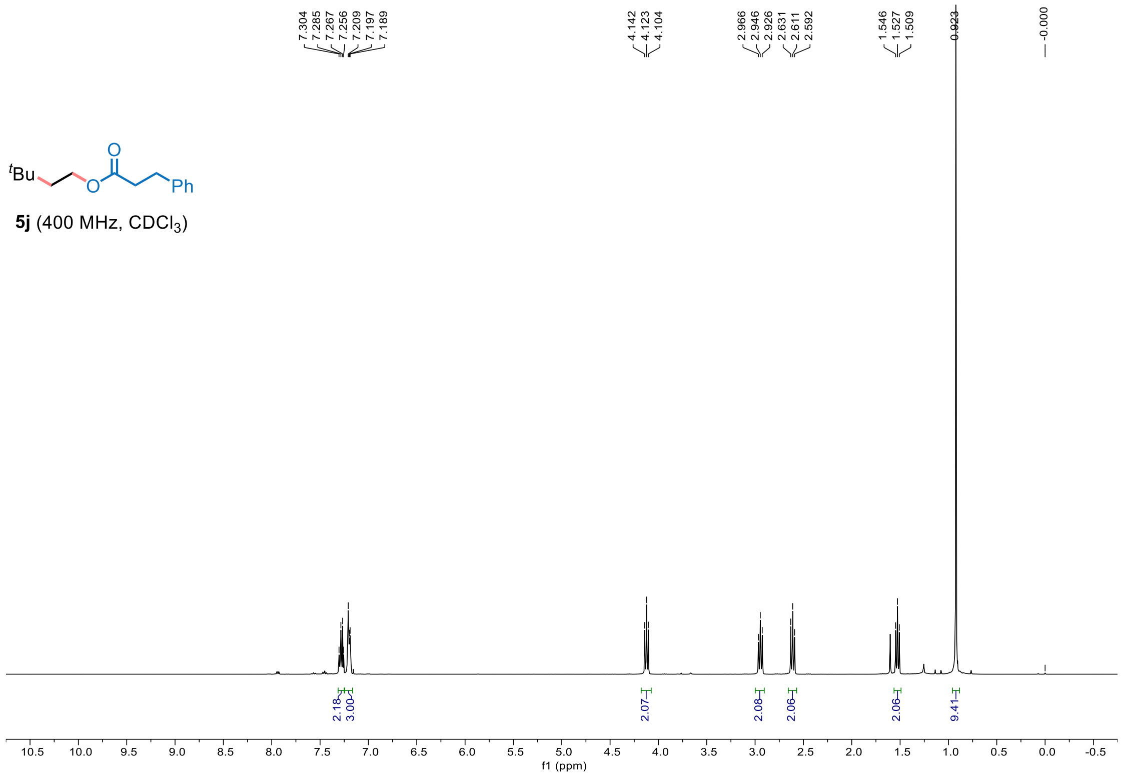

7.304
7.285
7.267
7.256
7.209
7.197
7.189
4.142
4.123
4.104
2.966
2.946
2.926
2.631
2.611
2.592
1.546
1.527
1.509
0.923
-0.000
tBu O O Ph
5j (400 MHz, CDCl3)
2.18
3.00
2.07
2.08
2.06
2.06
9.41
f1 (ppm)

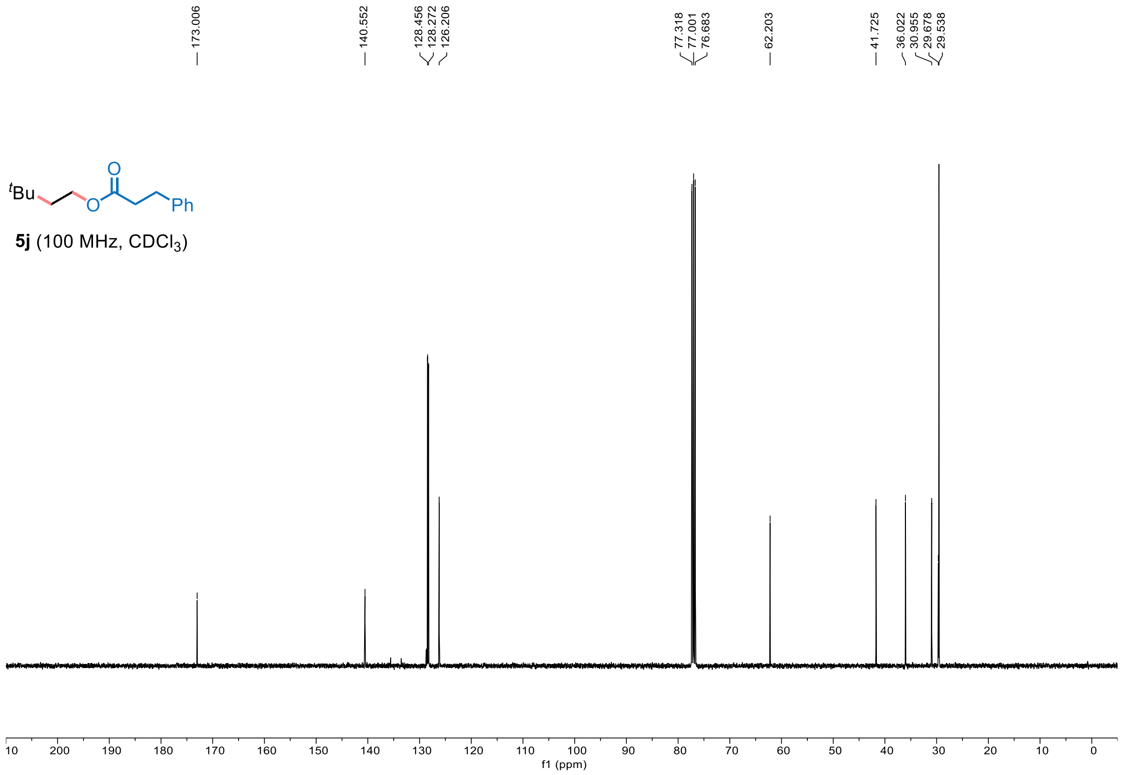
173.006
140.552
128.456
128.272
126.206
77.318
77.001
76.683
62.203
41.725
36.022
30.955
29.678
29.538
tBu
O
O
Ph
5j (100 MHz, CDCl3)
f1 (ppm)

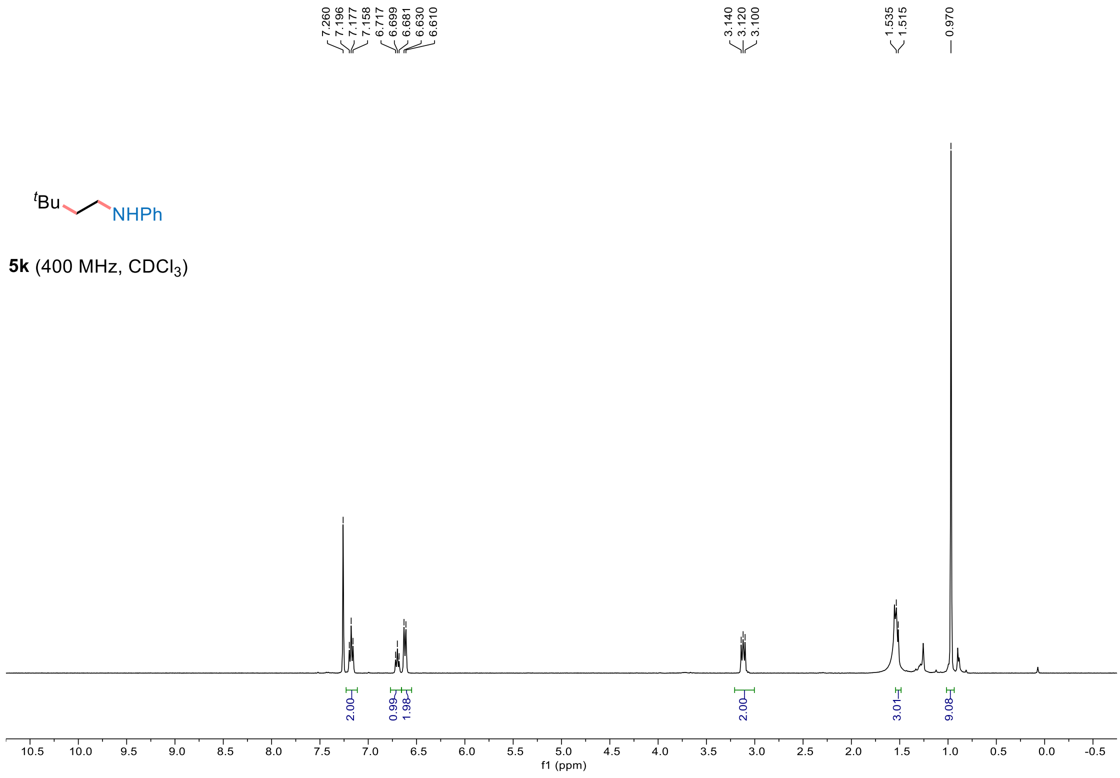
7.260
7.196
7.177
7.158
6.717
6.699
6.681
6.630
6.610
3.140
3.120
3.100
1.535
1.515
0.970
tBu
NHPh
5k (400 MHz, CDCl3)
2.00
0.99
1.98
2.00
3.01
9.08
f1 (ppm)

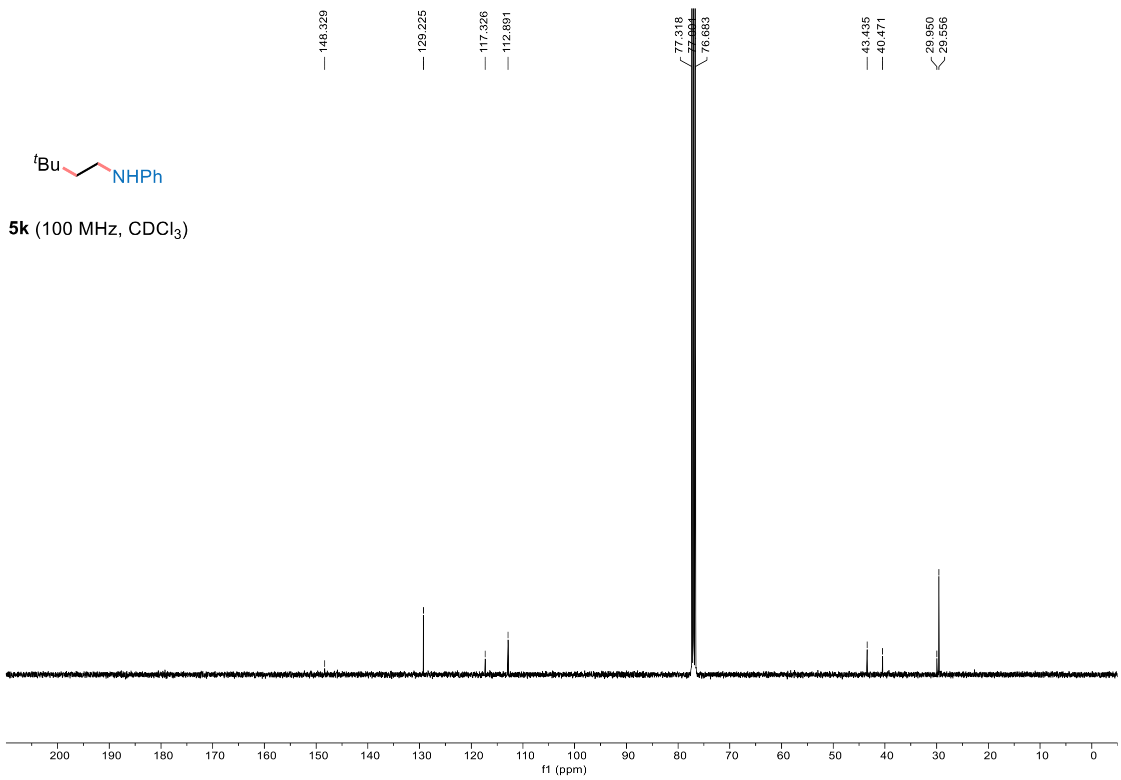

5k (100 MHz, $CDCl_3$)

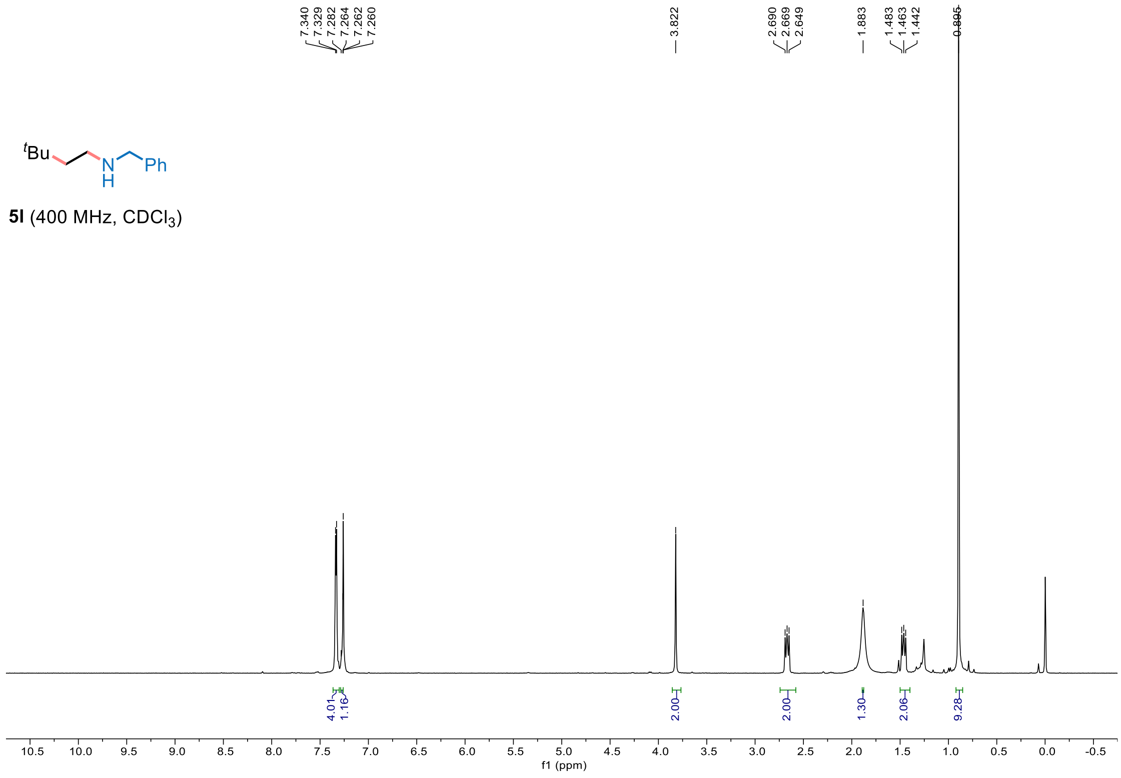

5l (400 MHz, $CDCl_3$)

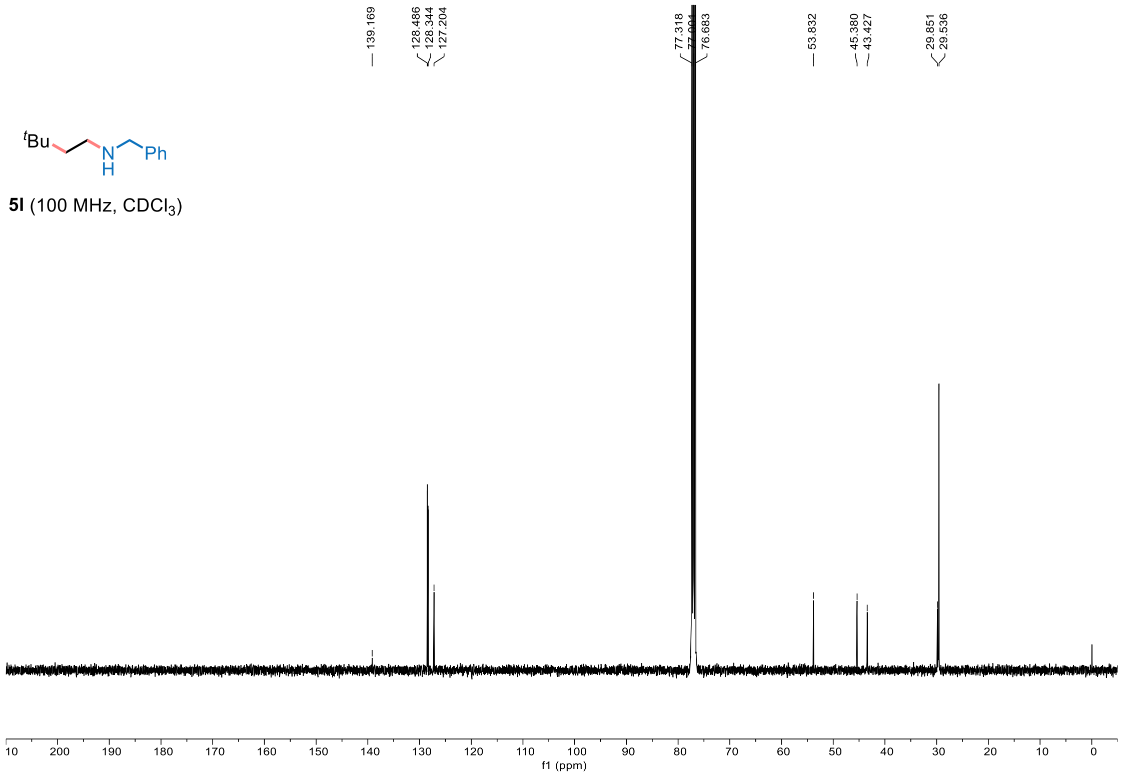

5l (100 MHz, $CDCl_3$)

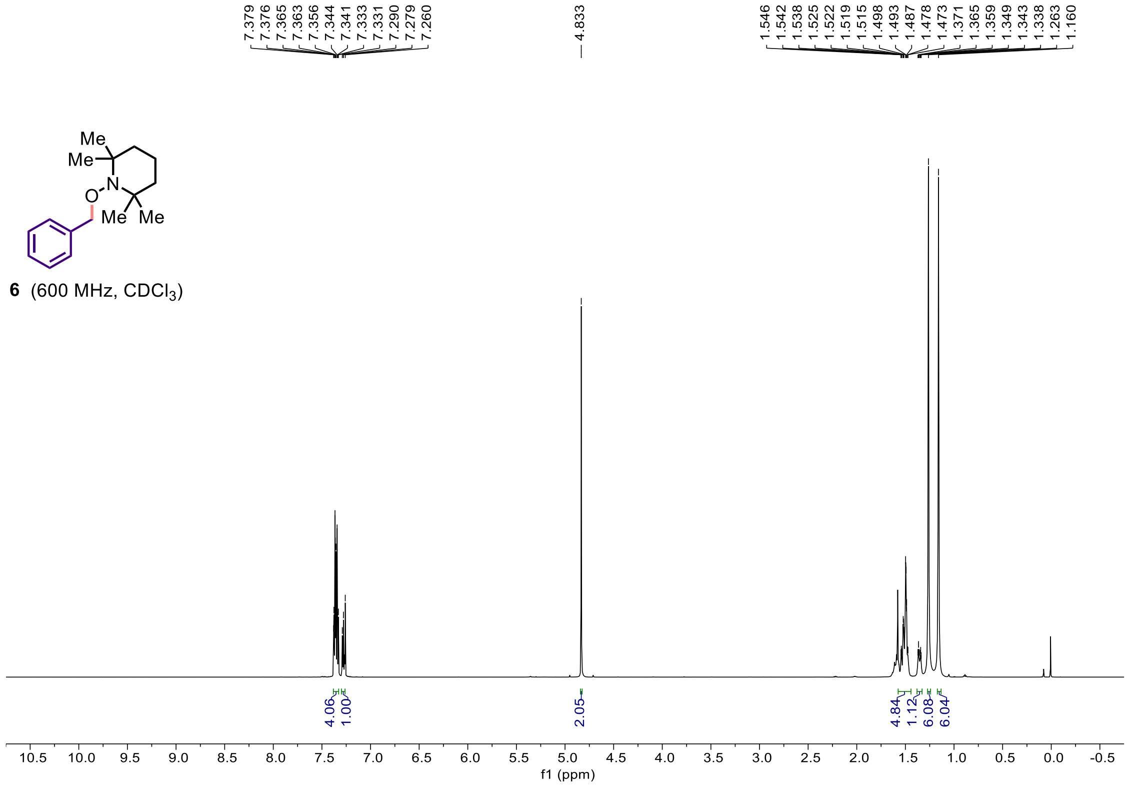

6 (600 MHz, $CDCl_3$)
